# Supplementary material for: Associations between SNPs and vegetation indices: unraveling molecular insights for enhanced cultivation of tea plant (Camellia sinensis (L.) O. Kuntze)
Source: PeerJ. 2024 Jul 18;12:e17689. doi: 10.7717/peerj.17689 (PMC11636977; doi:10.7717/peerj.17689)
Supplement: Figure S1 — Vegetation indices (VIs) with R2 > 0.5 and p value < 0.0001 are shown. Green - tolerant genotypes; blue - susceptible genotypes; gray - non-responsive [file peerj-12-17689-s007.pdf]

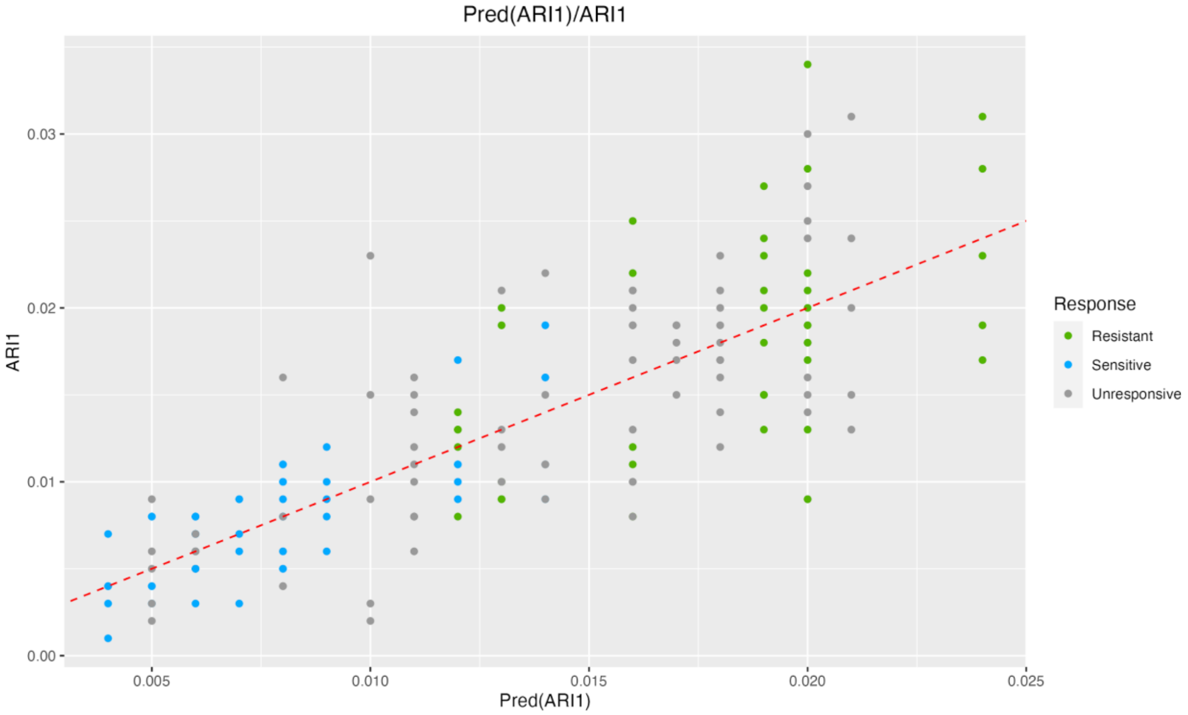
Pred(ARI1)/ARI1
ARI1
Pred(ARI1)
0.00
0.01
0.02
0.03
0.005
0.010
0.015
0.020
0.025
Response
Resistant
Sensitive
Unresponsive

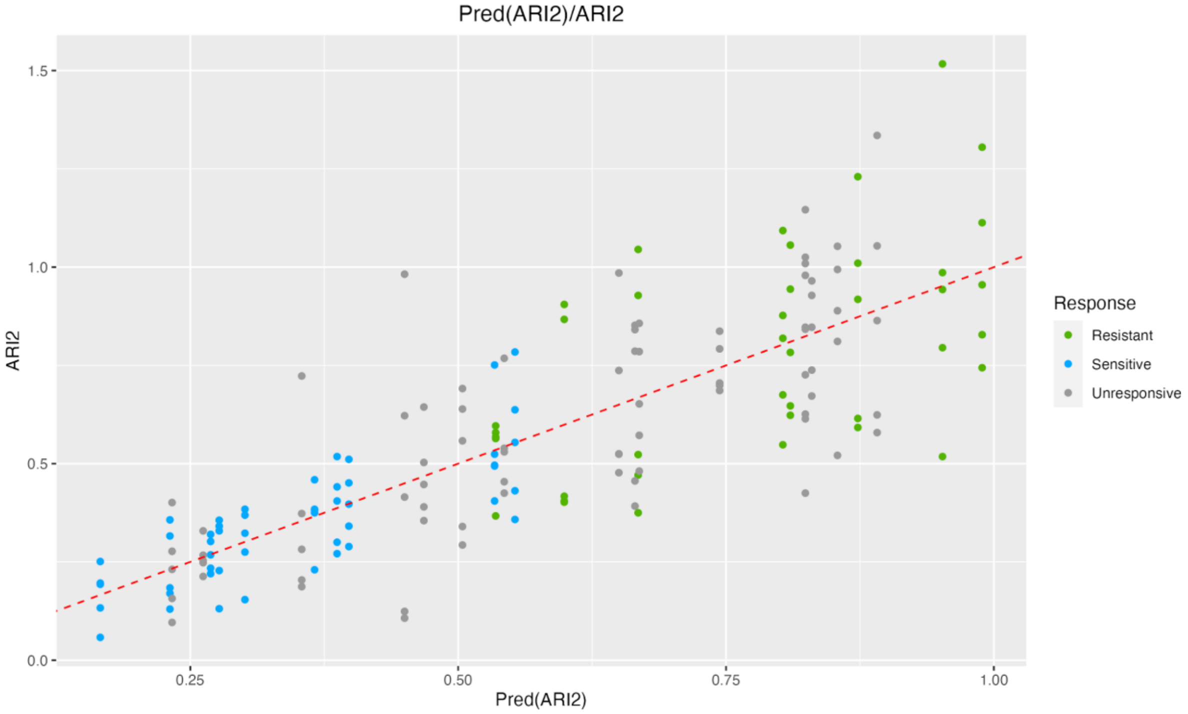
Pred(ARI2)/ARI2
ARI2
Pred(ARI2)
0.0
0.5
1.0
1.5
0.25
0.50
0.75
1.00
Response
Resistant
Sensitive
Unresponsive

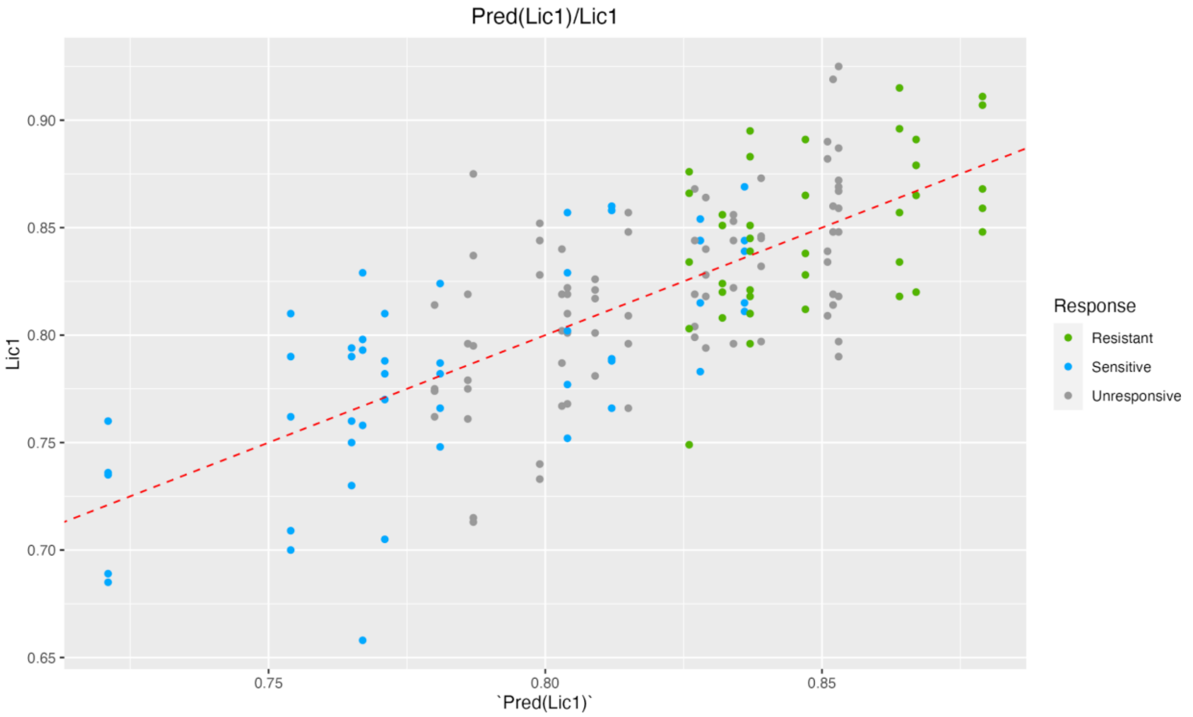
Pred(Lic1)/Lic1
Lic1
0.90
0.85
0.80
0.75
0.70
0.65
0.75
0.80
0.85
`Pred(Lic1)`
Response
Resistant
Sensitive
Unresponsive

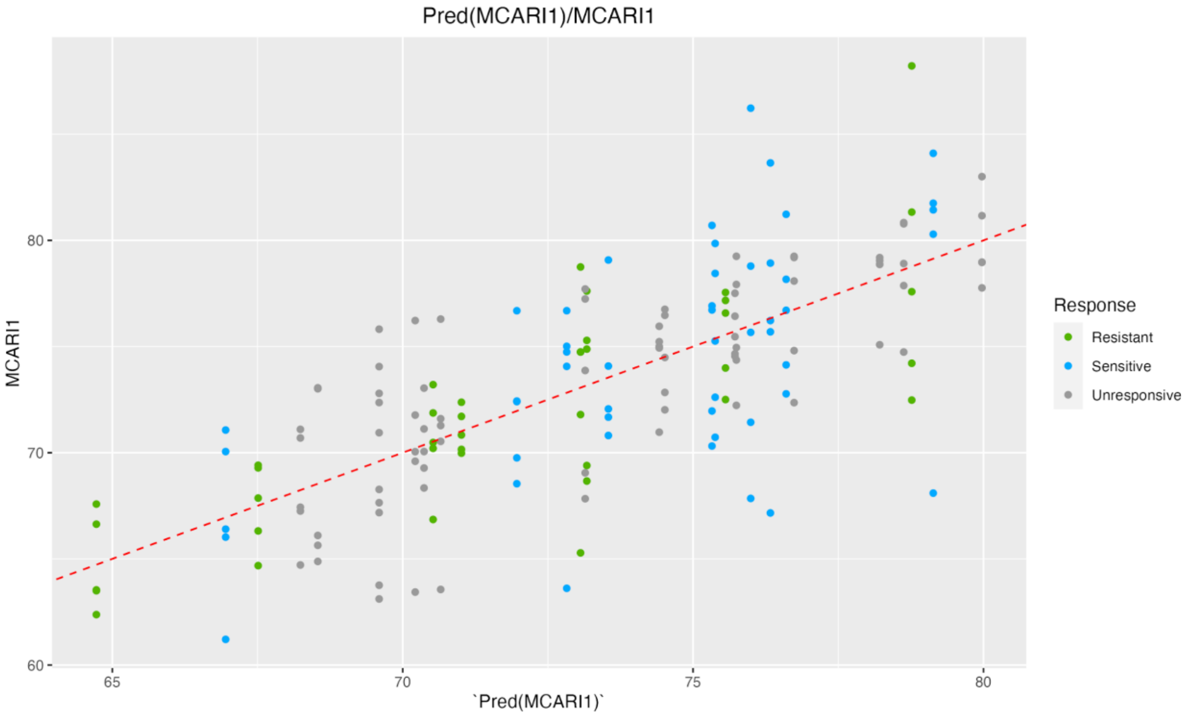
Pred(MCARI1)/MCARI1
MCARI1
80
70
60
65
70
75
80
`Pred(MCARI1)`
Response
Resistant
Sensitive
Unresponsive

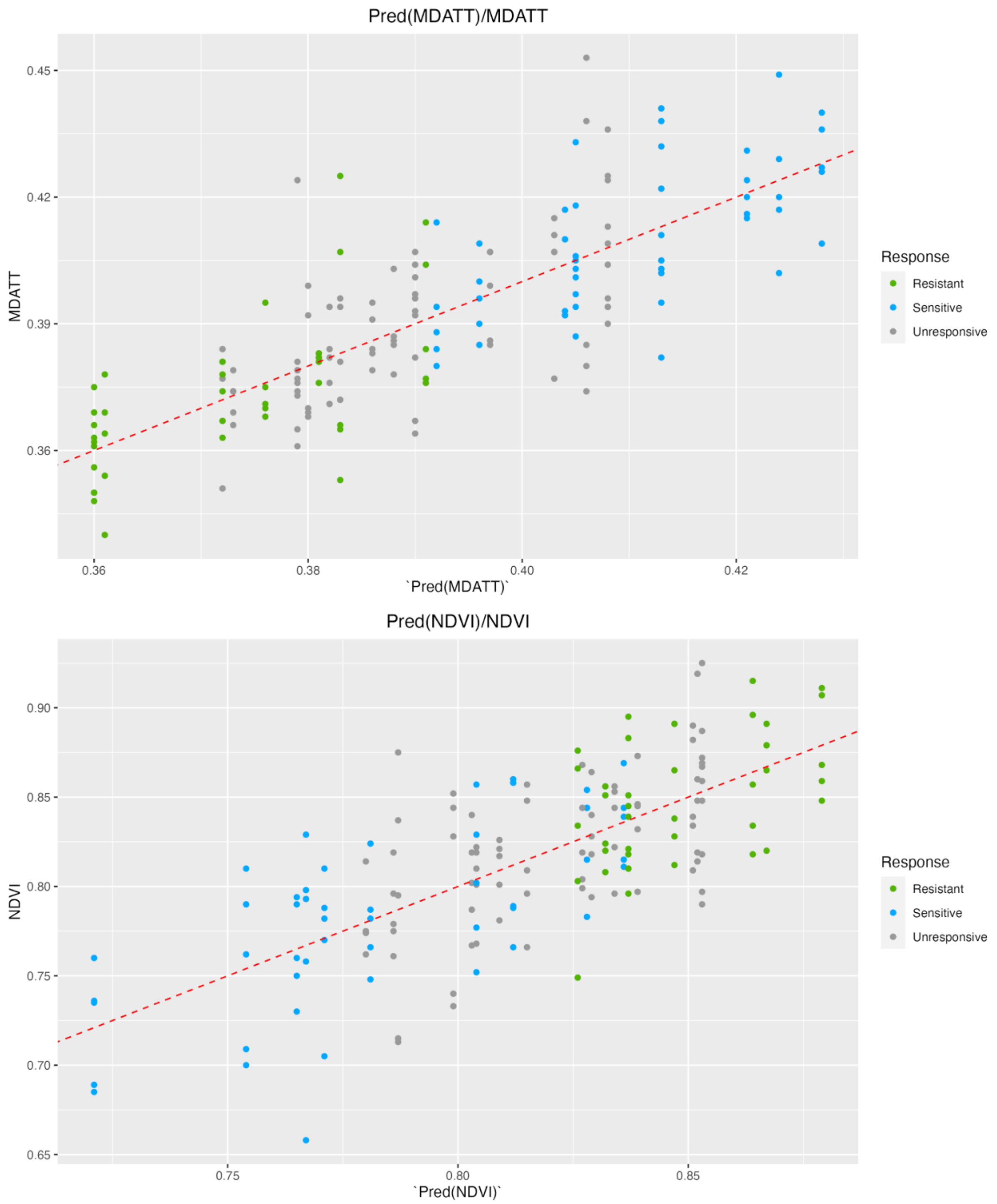
Pred(MDATT)/MDATT
MDATT
0.45
0.42
0.39
0.36
0.36
0.38
0.40
0.42
`Pred(MDATT)`
Response
Resistant
Sensitive
Unresponsive
Pred(NDVI)/NDVI
NDVI
0.90
0.85
0.80
0.75
0.70
0.65
0.75
0.80
0.85
`Pred(NDVI)`
Response
Resistant
Sensitive
Unresponsive

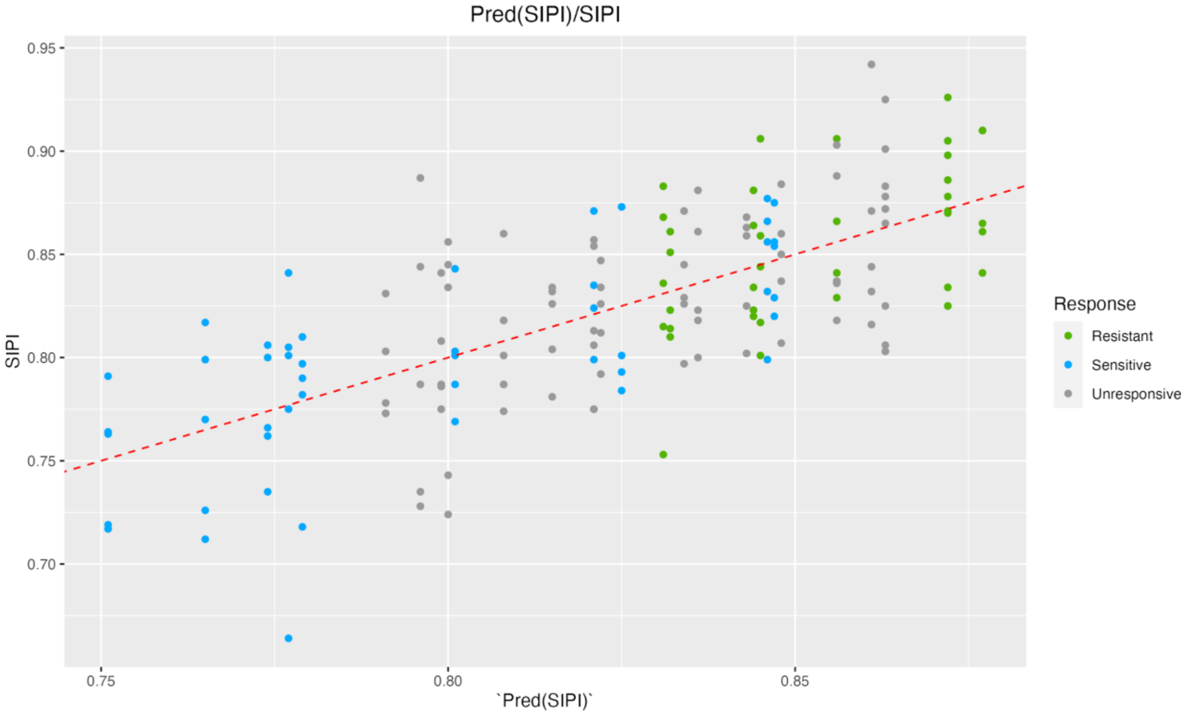

Pred(SIPI)/SIPI
0.95
0.90
0.85
0.80
0.75
0.70
SIPI
0.75
0.80
0.85
`Pred(SIPI)`
Response
Resistant
Sensitive
Unresponsive

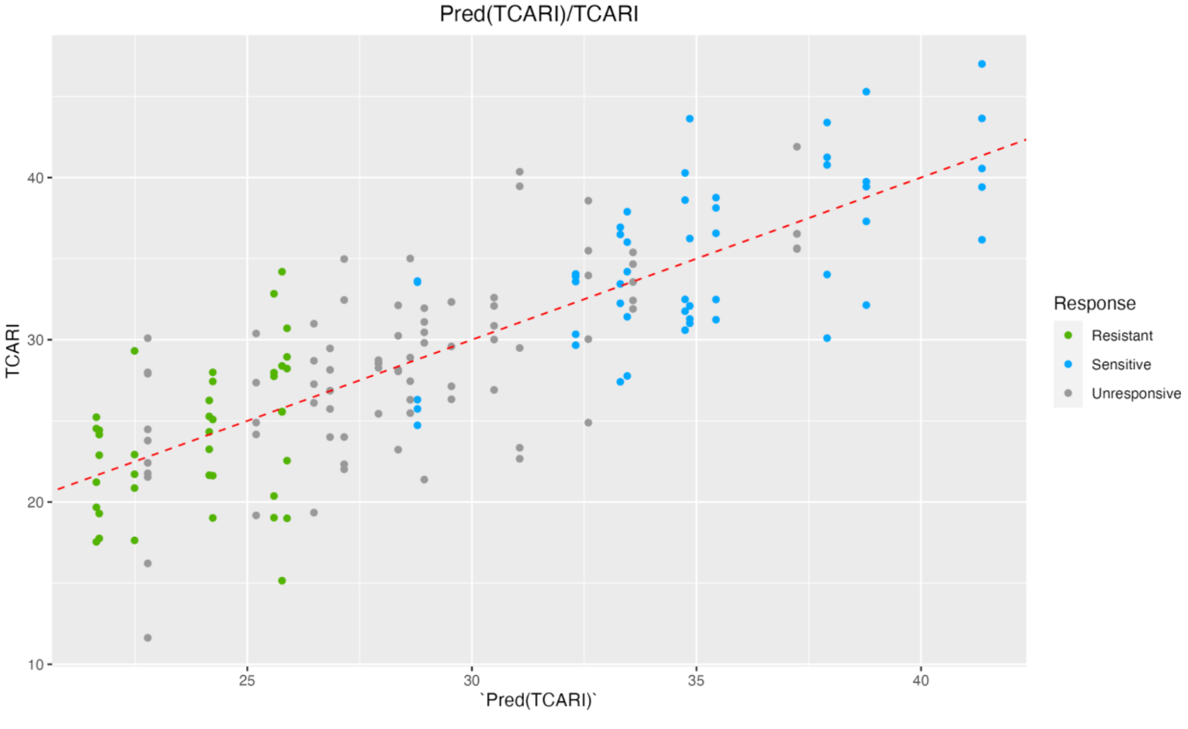

Pred(TCARI)/TCARI
40
30
20
10
TCARI
25
30
35
40
`Pred(TCARI)`
Response
Resistant
Sensitive
Unresponsive

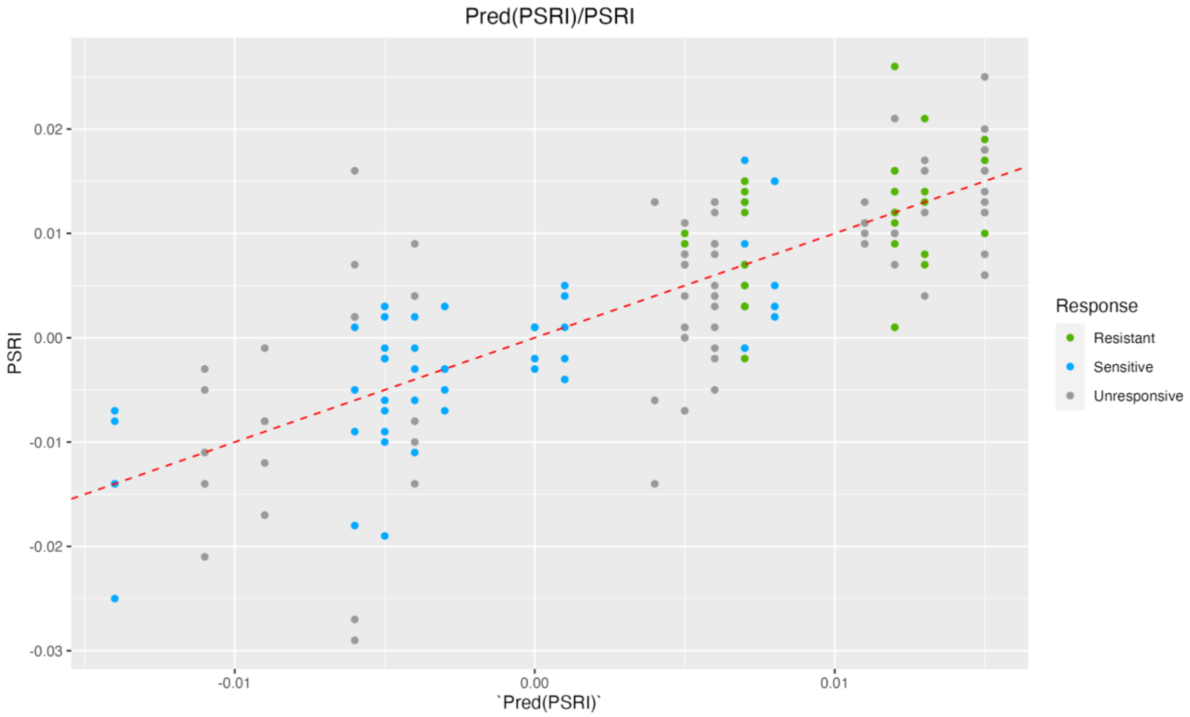

Data points distributions and prediction in the tea genotypes with different responses to nitrogen deficit. VIs with R2 > 0.5 and p value < 0.0001 are shown. Green - tolerant genotypes; blue - susceptible genotypes; gray - non-responsive
