# Supplementary material for: Associations between SNPs and vegetation indices: unraveling molecular insights for enhanced cultivation of tea plant (Camellia sinensis (L.) O. Kuntze)
Source: PeerJ. 2024 Jul 18;12:e17689. doi: 10.7717/peerj.17689 (PMC11636977; doi:10.7717/peerj.17689)
Supplement: Supplemental Information 8 [file peerj-12-17689-s008.docx]

>LOC114302347|CSS0007238.1 glutamate dehydrogenase A

ATGGCTATGAACGCTCTTGCCGCCACAAACCGCAACTTTCGTCGTGCGGCTCGCATTCTTGGATTGGACTCTAGGATTGAGAAGAGTCTGTTGATTCCATTCAGAGAAATCAAGGTTAAGATTGTTCTGCAATTTTAGTTTCTTGGGTTGTTTTTATTTTTTTGAGAATTGTGGAAAATTTTTGATCTTGTTGGTTTTGGGGTTTTGATCAGGTAGAATGCACAATTCCTAAGGATGATGGAACTCTGGTGTCCTACGTTGGATTCAGAGTCCAACATGATAATTCTCGAGGCCCCATGAAAGGAGGGATCAGATACCATCCTGAGGTGAATGTTTTATTTTTTCTGTTTTTTGTGCTTCTTTTGATGATTTTGTTGCTTCTAGGTTGAAATTGATTTTTTTTTTTGCTTTGATTTTGGGTTTTAGGTCCTTGAGATTCTTGGTTGATGTAATTTAGTTTGGGTTGAATTAAAGGTTTGTTTGGATGTCTATAGAAACAGAACGATTTTATTTTAATCCATCCATTCTTCTCTGTCAAGGGAAATTATACTGACAAGTGATAGAGCAAACATAAGAGTGAAAATCAAAATCACTTCAGATGCATGCCAGTTTTGATGAATGATTTCATTTTATTGGGATATTCAAACCTTGCTTCGAAGAGGATGATTAGTGTTGTGTTTCGTGTTTTTATACAGCTGGTTTCAAGCCCTGATAAAAGAGGAGGGTTATGTGTTAGACAGCCGGACAGCCAGCACGTAAAATCTCTGTCTGGTCGCTTATGAACACGAATCCTAATATGATAATCTTGAATTTCATGGTCTTCAAAATTGTTTTTAGGGTTGGTTTGGGATGTCCTAAAAAAGTGTACTTTTTGACTTATTGGTGGGTTTGGATTGGGTTTGGGTTGGGTTTGATGTGATGTGTTAGAGAGGTGATGTGATTTAAATATGATGATGTGACTTAAATAAGATAGTGACTTTTTGACTTTTTGACTTATTTAGTACATGCCAAACTAGCCCTTAGTATTTACTTTTTAGTTCTTAGCTTGGTTTAGCTAAACTATGTGATAAAAATGAATTTGAGATGCATACAATTGTATGGTCAGAGGAAAATCAGAGTTATTTGATTGAAGGCTTGTCATATGTTGGAGTCAGTAAACAATTAAGTATTGATTGAAGGCATGTCATATGTTGTTTGATTATTTTATTTAAACTGCATTAGAGGCTTTAAGTTATTTGATTATTTTATTTGAACCGTTGAATTGTAGGTGTTTACAACGAGTGACCCAACTGAGCTGTTCACTACATAGGGCTCTTACCAAATTGATCAGTATTCAGTATAGATAGCACCATAAGAGTATTATTGAAGAATTGATGGGGCTTTTTTATTCTAGTTTCAAGTGTATGCCTCTCCTTCCGAGTCAAAGCACTCCTTTCTAAATCTCCTATAAGGCCTTAATAATTGGTCTTTCAAGAAATTTCTTCTAATCAATCCTGAAATACTATTAAGTAATGTATATGTGAACCTTCTCTTTTTTTCTTTTTTTTTTCAAGTATAAAAGTTTTTCAAAGGAATTTGTTGCCAGTTATTCCTCAAAGTCCGTTTTTTTAGTTTAATGGGTATTGTACAAGTATCTTCTGAATATTTGTGCCAAATTTCTGGGAGCTTCACTTCTTTTTACTAATCCTAAATGAAAAAAGTAAAAATACACGATTTCTTCATTTAAATTTTGTGCTGAGATGTTTCTTAATTTTTTTCTACAAGGTTGATCCTGATGAGGTAAATGCTCTAGCTCAATTGATGACATGGAAGACTGCTGTAGCAGACATTCCTTATGGTGGAGCTAAGGGTGGAATTGGATGCAAGCCAAAGGATTTAAGTAATAGTGAATTGGAACGTCTCACTCGGGTCTTCACTCAAAAAATTCATGATCTAATTGGGGTTAATAGGGATGTCCCTGCGCCGGACATGGGGACTAATGCACAGACCATGGCTTGGATTTTGGATGAATACTCGAAGTTTCATGGTCATTCACCAGCTGTTGTGACCGGGAAGCCCATAGTAAGTACTGAAGACAAAAATCCAATGACATGTTTCTTGCTTTGAAATAAGATTTAATATTTATCTAGTTAAACAGAAAGTAATTTCTATCAAACTCTCACATGAACCTCACAATTTTAACTTTGCATTTTAGGATCTTGGTGGGTCCCTGGGTAGGGAGGCTGCAACTGGACGTGGTGTTATTTATGCCACAGAAGCTTTACTCGCTGAATATGGAAAATCAATCAAGGATTTGACATTTGCCATACAGGTATTTTTGTCATGGATTTTGTTAAATTAAGGCACATGTTAAGAAGTTTGAAAAGGTTGTGCATTTTGTATAAGTCTCTGCATTAAAAAATGTAGAATCTTTTTAAGCTTTCTTATCATGTGCCTCAAGGACACACGTTAACAAGACCCTTTTGTCATTGATACTTCAATGTCAATTTATTGTGAGAACTAATTCTTTTCTAAGTTATTAATAAAGAAATTCTTTTTCAGGGTTTTGGCAATGTGGGGTCTTGGGCAGCAAGGCTTATTCATGAGAGAGGTGGTAAGGTCATTGCAGTGAGTGACATCACCGGAGCAGTTAAGAACCCAAACGGGATTGATATTCCAATTTTGCTTAATCATAAGGAAGCAACGGGGAGCTTGAAAAATTTTGATGGTGGAGATGCCATGCATCCAAATGAATTGCTCCTACATAAATGTGATGTCCTGATTCCATGTGCCTTGGGAGGAGTTATCAACAGGTTTATCTCTTGTTTTGCCTAGAAACTTGTGACTTTCTTTATACTTTTGTGTGAACATATTGCCTATTGCACACATAATCGTGAATGATTTGTACATCTCACAAATATGGGTGGTGTTTGGGAAGTTTGGGACTTGGGTTTTTTTTTTGATTTTTTTTACAAAAAGATAAACTTTTTGCAAAATAAAAAAAAACACATCCAAACAAAATATCACATCTAACCCCATAATCATTTCACTTTTTCTTTCATAAATATCACATCCAACCACAAAAAAATCCAACTATCTTTCCAAACACAAAAAAAAATTTCTCCAATAATATCTTACAACTAAAAAAACCTACATAAAAACCCACTTCCTAAACACTCACATGAACACCACTCCCTCTCCGTTGCATGGCAATGTTCGAGGGCACAAAATGACAAATCAAATTTTAACCGGCGTCATAATATCATCCTATCAAAATTAATATTGGGGTTTGTGATAGGAGTTAGGTTGATGGCATATCGTAAGTATATAATGATGCTGATCAAAAACCAAACCAAACCAAACCAAACCAAACCAAACTAAACTAAGCCTTAAGTCTCAACAAATGGGGTCGGTTACATAAATTCTTCTTCTCCATTCGACTTGATTCAAGGCTATATCGTTAGTCAAATTAAGGAGATTCATATCTCTATTTATTACACTAGCCCATGTCAATCTAAGTCTTCCTATTTCCTTAACATTTTCATCTACCGTCACTGTATCGCATCTCTTCACTACTGCCTTTGTTGGTCTCCGTTGTATATGACCAAACTATCTCAATCTATTCTCCCTCAACTTATCTTCTATCGGTACTATTCCTATCCATTCTCGAATATACTCATTCCTAACTTTATTTTTTCTAATCTCGCCACACATTTACTTTAGCATCTTCATATCAGCAACACTCATCTTATGTACATGTTGTTTCTTAATTGCCCAACATTTCATCCCATACAACATTGCTGGTCTAATAGTCGTTCTATAAGACTTCCCTTTTAGTCTAGCGGGTACTCGTTTATCGGACAGTATCTCAGACGCGCTCCTCTACTTAAGCCACCCCGCCTTAATTCTATGGATCGGATTTACTCCAATCTCTTTATCTGTTGTGATAATCGATCTTAAATAACGAAAATGTTCACTTTTAGGTAGTACTTGGTTCTTAATCTTCAATTCTCCTCTACTTCTATTATTACTGTTACTAAAATTGCACTCCATGTTCTTAGTCTTAGTTTTGCTTATGGTAAATCCTTTAGATTCAAGTGCTTCTCTCTAACTCTCTAACTTAGTGTTAACTCATTCTCTAGTCTCATCTACTAACACGATATCATTTACAAAAAATATATACAATGGCACATCATCTTAAATATTTCTCGTCAACTCATCCATAACTAAAGTAAAAAGATAAGGACTTAAGGTTGATTCTTGGTGTAACCCTACAGTAATTGAAAATTCATTTGTCTCTCCAGCTGGCGGTCTAATAGTGGTCACCACACCTTCATACATATCCCTAATCACATTAATGTAACATTTTGTCACACATTTCTTCTCTAACACCCTTTAGATTATATCATGAGGAACTCTGTCATATGCTTTCTCTAGATCGATAAAAATTATATAAATGTCTCTTTTCTTTTCTCTATAGTTTTCTATTACTCTCCTCAAAAGATAGATCCCTTCTATTGTTGACTTACCAGGCATAAATCCAAATTGATTCTCTGACACCTTTGTTATATCTTGCAATCGACGTTCCATCGTTCTCTCTCACACCTTCATTTTATGACACATTAGCTTGATTCCTCTATAATTATTGTAGTTCTGGACATCTCCTTTATTCTTATAGATTGGCACTAAGATATTCTTCCTCCACTCCTTCGGCATTTTCCTAGTCAACAAAATCTTGCTAAATAACCTAGTCAACCAAGCCACCCCTTTTTTACCCAAATATTTTCAAGCTTCAATAGGGATATCATCGAGTCCTAAAGCCTTATTCGATTTCATATTCTTAAATGCAATCTCCACTTCAAAGTTCCTAATCCTATGATAGAATATGTAGTCATGGTTTTCCTTATTACTGCCTATTTCCTCCCCTTTAGACCTGCCTCCATATTCCTCATTTAACAACTGCTCAAAATAGTTCTTCCACCTCTTCTTAATCTCTTCATCCTTCACCAACACCTTAGAGTCCTCACTATTGATGCACTTTACTTGATCAAAGTCTCTTGTTTTCCTCTCTGTTATCTTAGTTGGTTTGTATATCCTCTTCTCTTCCTCCTTACTATCTAGTCTAGTTTATAGGTCATCATAGGCTTTCAACTTAGTCTCTCTCACTATTTTTTTTGACCTCCTTATTAGCCAATCTGTAGCTCTTAAGGTCTTTCTCAGTTCTATCGTTTTGCCACTTTTTAAAGTACTCTTTTTTTGTCTTTACTACTGTTTGAACCTCCTCCCATCAGCACAAAGTTTCCCTAGAGTTGGGTCCTTTTCCTTTCGACTCTCCAAGAATTTCTCTTGAGATTCTCCTCATACAATTGGCTATCAAATTTCACATAGTGTTAGCATCATCTTCCAAGTCCCAAGTACCCTCTTTATCCATTCTATTATTAAATATTTATGGTTTAGCCTCAAGTCCCACCATCTCGTCATACGGTGCCCACTATCCTTCCTAACTTGGTATTGCCTTTTAAAGTATAAATCTAGTATCAAAATTCTATGTTGAGATGCCAGGCTCTGTCCAGATATTACCTTACAGTCGCTGCATTTTGGACTTAAATTATTTATCCATATTTTCGGTTTGGTTGAGATCTATGAAGCACGGGTACTCCAAAAATAGTGCCGTACGAGTACCGGGTACTTACCGGGTGCGGGTACGCGTACCGGTACGGCGGTACGCGTACCGGGTACGGCAAAAGAGGGTCCGGTACGTTTGGCTTATTCGGGTACGGCATGGGTACGCCACGGGTACGCCATGGGTACGGCGTGGGTACGCCAAGGCCAATTTTTTTTTTTTTTTTTTTTTGCCCTTGCAAATTAGCAATTATTATTATTATTTTTTATTTTTTTTGGTGCCCTTGCAGATCAGTAATTTTGATGTTTTTCTGTTGTCCTGTTTGTCTGTATAAATTGTAGAAGCATATCAACAAATGATTTTAATATTTTTAAAAAGCAATTTGAACCAAAAAAAATAACAAGGCCTATAAAGGGTAATTCTGTGTATGACTATGCATTATGCTTTTGCTTTAGATCCTCCTAGAATTTCTTTATAAAATCTTTAATCATAACAAACTTTTAAAGTTTATGCACAAAACATTAAAAATTGATACTGTATTAGTTGATTAGTAGTTGTTGGGAATGATGCAATTTTATGTATTATGCAAATTGCAATTGACAACCTGCACCAATTGAGAATTTTTTTCCTTTATTGTTGGTAATTAACTTTAAATTTTTCTTTGGTCTCCTGTTATTTCTTTGGTCTCACCCTATAAAAATAGCAAATTGATGAGCAAAAAGAAAGATTTTAAAAATAAATTATGATCACAAATTCACAGTGCATAACATATCAAAGTGAGAGATTTCACAAATATTATTTTTAACGCTTAATGGTTTTAAATTATAGGAAAATGAGTAGTTCTTTTTTTAGTTTTGATATTTAATTTTAATTTGTAGGTTGAATCCTTTAATCAAAATTATATTTAAAAAAAAATAAAAAAATATCGGCGTACCGTAGCCGTACCCGTATCGTACTTTTTTTGAGTTTTGCCGAATCGGCGTACCCGCACTCGTACCCGTACCGTACTCGTACCCGCACCCGTGCTTCCTTGGTTGAGATGCTATTTGAAGTTGCTAGCAGGGTGGTGGATCATTAGATGCTCAAGTTGATTTTAAAAATGGATATATTTTGAACTCTTCTTATGCAAAATATCCTGATTTTCTTGACTTCTTGATATTCACATTTGAAGAGAAAACGCTGATAATGTGAGGGCCAAGTTCATTGTAGAAGCGGCAAATCATCCTACTGATCCAGAGGCAGATGAGGTATGACATTGACTTTAAACTTTTTAACTTCTCTTCTGCTTGGGACATATGTTCATCATCTGAAAAACTGAGTCATTGTTTCTGCAGATTCTATCTAAGAAAGGAGTTATAATACTACCTGATATATATGCAAATTCTGGAGGTGTGACGGTCAGCTATTTTGAGTGGGTCCAGGTAACAAAAATACTCGATATTCCTTCTAATTTCCAACATGGTGAGCAGTATGTGAAATTGTGTGAATTAAATTTTATTTTTTAATTAACTAATATATGCCTCCAAAAGATCTATCAATATTCTTACACACTCTACTTTTTTTTCCTAGAATTTGACTTGCAATTGAAAGATGTAGACCCTAAAACAAGTTGATTCTTCCTTAAATGTGAACTTGGTGAAACTATATGCAGGAGGACTCTTTATTTTTCTCATGTTTTTCAAAACTATTTATTACTTTGAAGAAAATTTTCTCACATATATATTTCACACGCTCAAAATATATAATTTTTTTTATATTTATTGTACGTCGATAAGAAAAAATGTGTTATATATATAAATATTTTATTAAAAGATAATAACAAAATATAATATATAAAAAATCAAATAAGTATAAAATTTACGTAGTTTTTCTTTTATCATTGAATTTCAAGTTATTTTTTTTGAAACTTGTAATGTGTTTTATGTGTCTCTAACCAATCCACGTGTCGCAAAGTGTCCGATTAGTGTTGTGTCATGTTAGTGTCTATGCTGCTTAGGTGTCAGTTATGCATCTTGTCTGTGGAAACTATAGGTAAAAACAACAATAGTGCGTAGATAGCAGATTTCGAGCGGAAACAATTGTAATGTGCCACAATGAGTTTTGTTCTTGCAGAATATTCAAGGTTTCATGTGGGACGAAGAGAAGGTGAACAAAGAGCTTCATAGGTACATGACAAAAGCTTTCGGTAACATCAAAAGCATGTGTCAGACACACAATTGCAACCTCCGAATGGGTGCGTTCACGCTGGGAGTGAATCGTGTTGCACGGGCAACTGTATTAAGGGGTTGGGAAGCGTGA

>LOC114264550|CSS0010852.1 lysine histidine transporter-like 8

ATGGAGGAGAGGCCGGAAACAGAGCTGATTTCGATACCGGCGACGCCGCGAGCATCGACTCCGGAGATTCAGACGCCGTCGGGACAGAGGTCGCCGAGGCCTCCGCATGCGGCGGCGTCGAAAGAAGCGAAGTCGTGGACTCCGACGTCATTCATATCGCCTCGGTTCCTGAGCCCGATTGGGACTCCGATGAAGAGAGTGTTGATTAACATGAAGGGTTATTTGGAGGAGGTTGGGCATCTCACGAAGCTCAATCCTCAGGACGCTTGGCTTCCCATTACCGAGTCTCGCAACGGCAACGCCCACTACGCCGCCTTCCACAACCTCAACGCCGGCGTCGGATTCCAGGCCCTTGTCTTGCCCGTCGCCTTCTCTTTCCTCGGCTGGTACTCTCTCTCTCTCTCTCTGCGAATCCCCATTTCATTATTGGGGATTTGAAATGAAATGGGTATTTGTTTGTTTGATTAATGCTCTATGCAAATTAACAAATCTACAATTTTTTCATACTGAGAGTCCTAAGTTTGTTTAATGTGTGGCATATTCGTTAGTAAAAAAGAGAGTTCGTAAGATTTGTTAGTTTTGAGTATCATTTTCCTATTTGTTTTTGTGAAGTTTATGTTTTGATTGTTTGTAGCAATTCTTTTTTTTTTTTTGGAATTTTAGTTTCAAGCTAAAGTGAACTCTGTTTGCATCAAGAGAATACTTAGAACCCAAAATCTCTTCAAATATTAAGTTAGAATTTCTTGAAGCCATCCACCAAAACCTTTTATTTATTTATTTTTATTTTTTATATGACTTGGGTATCCAGTCCAATGGACTGAGTAATCCGGGACCAATCCCACTGTTCAGTTGAGGCGTTCCAATTAAAGCCATGACAAAAGCCCGTATAGACTGGACCCAAGAAATTGTTTACAGCCTACAGGTTTCGAACGTAATACCTTGGAGGTAGCACACCCCAATCCCAAGCCTTGACCACCACGCCAACCGCTCGGGGTTATCCAACCAAAACCAACGGGCTAGATAGATGGAGGGATCCTTAGATTATACGAAGCAAATTTAAATAATTCAGAGATGCGATGTGAAATATGTCTTTGCTTTCTCTCTGTATGTTTAACTGTATAAAGTTGATTTTTCTTTTTCTTTTGGTGTTTAATTGATTAAAGGAGATGGGTTGTGGGTCTAACTGGAGATCCTTCTTGTTTTGCTCTTTTTCGAATTGCATAATGGATTTGATTTGAATGGAATGGAAGAAAGAGACATCCTTTGAGGCATCTCTTTTCGTTCCTTTCATCTTTTGCTTTTTCTCACAATCTAAAATTATTTTACAGACAACTACACCACGTTACTGGGCCCTCTGTTTTTGCTTTTTTTCATGTCACATTATCCATTTTTAATTACTATGAGAAGCAATTTTTTTAAGACCAATCTCTGCTTTCATTGAATTCCCCACCTACAGTTTTCCCTTCAAAATTTTTATTTTTTATTTTTCATTTATGTTCAGAATATTGGACTGGTTCAAATATGATTTTTGCAAATTTTTCTTTCAAAAATTTTGGGCTCTATTAGAACGCAGATGAAAATAATTTTGTTCACTCATTTTTATGATCTTTGAACATTTAGCCACTTCATCGTTGGTTCTTCCATGTTTGTTCATGGCATTGAAAGAACCAAAAGGGGAGGCTTGTAATGTTCACTCGTCATAAAGGTGAGAAACAAAATAAGTCTTGAACCCCACGTTTTATTGCCAAAACTTTTAGAAGATCATAGTTCAATTAAGTATTCTAGTAAGATATAGTGTTTTAGGAATTCGCAAACAATGATGTGTATATATATATATTTTTATATATATCATAGCATAATTGTCCTTGTGACATGACGTTATAATCACTCTTTAACATTTCTATTTGAAATGGGACCTCTATTATGGTGTTAAGTAGGAATGGGCATGCCAATCAGATTTTTTATTTGGTTTATTGTAGTTTTGTACCCATATCAAAGACTACATTTCAGGTTCGTTGCTTGATATCCTAATTTACGGTTCACTCTGCTTTCTGTGATCGTAAATCCAAAGCAATCATTCTTCACTTCAAAGTAAAATATCATACGAAATTTGGTTCTTCATCCATATAGGGAAGGTACCTTTTTCCTAAAATCTGCTACGCTATCAAGTTTGAGCTGCTTATGTGATAATTGAATGGTTCCAATCCACAAAACAGTGAAGAAAATAAATCAAAGACAAGACATACGTGGTTTAGTGCCTAACGGCCTAAACCCTACACCCTTCCATAAAAGTCTCGTTACACTAATAATACAAGACACCTCACAACAGCTTAGTATCTACATCCCTTCTAGTATTAGATGCTAATGCATTGAATTGATTGTACATTCCAAAGTTTGTAGTCCTGTAATATAAATGTGCTTCTTATGTAAGGAGTTCAAATTTTTATAATAATTTGTCGCTTTGATAAATATCTTGTTCATAGTTTGTTTTGACGTTGGTGGAGTTTTTCGTTAGTTATGGTTATGGGCCCTCCTCCATATATGGAGTGGTCAAGCTATGTCGAAACCTTTGCTAACATCTCAATCTCTAATATATTGTGCAACTTTTTCATTAGATTAGATTTAAATGGAAAAGAGACATCCTTGTGGCAAGTCATTTCATCTCTTCAATCTTCTATTTTTTGGTTACCATGACTGCAATTATTTCATAGTTATGTCATCCTTCTAGGGGCCACTCGTAAAGATTATCTTCTCATCGAAAAAATAATTGTGTTTTTCTTTTGTAGAAAGAGGAATGGGGCCTACATTTCATGCTTGAGTGTTGCAACAGAACTTCTCAATAATACCCATTTCTAATCATTTGTAATAGTAACTCAAAGCACTTTGCCAGAAAAAGAATTTGATGCTAATTAAGAACTTGTGCTTTACATGGATATTTTATATATTTTCACTAGAAATGATAATTGTGATAATAATAAGTGACAGAGCATGCTAAATCAAACCGAGTAATATTTAGGCTCCGTTTGGTGGAGCTTTTAAAATATAGTTCCACACTCATTTTTGAGTGTGGTGTTTGTTTAGCATTTTCAAAAATTAATTCCACATTCACTTTTGAAACTCTTTTCAAGGCAGGAAAATTTAGACTAACTGGTCGTCCCACCGGTCGTCCCACCGGTTGATTGGAACAGTGTTCGGTTTTTGTTTTTGTTTTTTTTTGTTTTTGCACCTGTTATTTTTATATTACAAAAAAAAATTATTTGTGAATTTTTTTTAAAATTTTTTATGAGCTTTGAATAGATTTTTGGATTGTGTGATTGGTTATGAGTTGTAATTTTTTGTATCAAATATCATATATTACATCACTCAATTCAAACTCAATTTAAAACTCTTTTTAAAATTCATAACCATACAAATTTTTCACATTCAAATCATAATTTCAAAACTCACAAACTCAAAATACAACTCTATTCTTAAATAATAATTCTAAAATTCTACCAAACACTACCTTAGTTTTGTTTTTGGATACTTGTTTGGTTTTGGAAAGAACCTGGATGAGGGTATGGGTAACAAAAAAACCACTTTTATTATGTTTGTTGCATTTGCAAATGGGTCCCCTCTATACTCATGTGCGCATACGGGTGGTAGGTCAGTCAGGTTTTTCATTTTATTCATCTAGGTGTAATACTTGCTGATAAATGAATCCATTTTGACCTTTTATTCTGTGTGGTATCTCAACTACAACGTAAAAAAAAATAAAAAAAAGAAAGCAAGAATGAGTACAACCATTCCTATACTTGAAAATATTTCCATTTTATATAATATTATTACCAATATAGAAAATTTGGAGAAAAGAAAGAAATAAATAAAACAACTTGTTGGCCAATTTTTATGTTTGTGAGCCTTTTAGTCATGGCACTAAGGAAAAGAACAAACGGAAAACATTGGTTAATCGCCTTGGCTTTCCTATATCAATTATTTTTCTAGCTTACTTGGGATTTTGGGGTACATTTCTAAACATGTACTTTTCTATTGGTGTTCTACAGGAGTTGGGGAATACTTTCTTTGACGATAGCCTACTTCTGGCAACTTTACACCCTCTGGATTCTGGTTCAGCTACACGAAGCAGTGCCCGGGAAGAGGTACAACAGATATGTGGAGCTTGCACAAGCTGCATTTGGTGAGATCAAATTCCATGCCCATCTTTTAGTTACAATCAATTCATCACCTAACCGGTATTTTCAAACTATTAACAAATTGCTTATAGCCATGAATACAATGGGGGGACTAAACTGGATGTGCTTTTTAAAAAAAAAAACAAACTGCTTATTGTAATCTCAACATATTTTTGCATGTTGATCTAATAAAATAAAAAACTTGAGAATGATCGGTCATTAAACAAGATACTCCCTCCGTCCCAAAATATTAGTCCCTTATTCCATTTTTCATTGTCCCAAAATATTAGTCCCTCTTTATAAATCAAGTACAAAAATAATGTAAATTTCCTATTTTGCCCTTCTTTTAAATTAATAAACAATTTAAAAAGTACAATTATGACATTGATATTCCCAAAAAATGTACTATCAAAAGGGTAATTTTGGAAAACCAACTTTTTTTAAATGCAATAAGTGTACTACCAAAAAAAGTTGGATTCCCCAAAAGGGACCAATATTTTGGGACGGAGGGAGTACTTATAATTCTTTACATCCTATTTGTCTATGGTTCATTTGCATTACTTTTGCTTTTTCAGGGGAAAGATTAGGTGTCTGGCTTGCTCTCTTCCCTACAGTTTATTTGTCAGCGGGGACGGCAACAGCTTTGATTCTTATAGGAGGGGAGACGATGAAACTGTTCTTCCAGATAGTTTGTGGGCCCCTATGTTCATCAAACCCTCTATCGACAGTGGAGTGGTATCTGGTCTTCACGTCCCTGTGCATCGTATTGTCCCAACTCCCGAACCTCAACTCAATTTCAGGACTCTCTCTCATTGGGGCAATCACAGCCATTACTTACTCAACTATGGTGTGGGTTCTTTCAGTGAGTCAACCAAGGCCGCCCTCAATCTCTTACGAACCCCTTTCATTACCTTCCTTTTCAGCCTCTGTCTTTTCATTTTTTAATGCACTTGGAATTGTCGCGTTTGCATTCAGGGGACACAATCTAGCCTTGGAGATTCAGGTATGCACTCTCAAGATCTAATTTGGTAAAATTTAATCCCTCTCGTGAAACAATTCCCAGAATATTTTTATAGATTGAAATAAGCATTTTTGTCATTGAAAGAGTTTCATGTGGTATTTTTCAGGCAACAATGCCATCCACTTTCAAACACCCGGCTCACGTGCCCATGTGGAGAGGAGCCAAAGTTGCCTATTTCCTTATCGCGATGTGTGTGTTCCCTATTGCTATTGGAGGTTTTTGGGCCTATGGAAACCTTGTAAGTGTACTGTCATTCTTTTCTCTCATGTCTACACATTAAAAGCCCGAATAAATGTGACCACCACCTAATAGCATGTTGATGCCAAAAGCAAAACGCCATAACTCCATCCTTTTGAAGATTTTACTTAAGAGAACTAATGATGTGTACAATACTTGAATTCTAGTATACAACACTTCAAAGACTCATTTAACATTATAGGTGTCATTAAGTTCTTGTCTAGTCTCAACCACATAAATGTGTGTTGCATTTGTTGATGATTAAATTCAGCATTTTAACTGTTTCTTTCTTGTAGATGCCTTCGGGAGGCATTCTCAACGCCTTGTTTGCGTTTCACAGTCACGACATCCCAAGAGGACTTCTTGCCATGACATTCCTTCTAGTTGTGTTCAACTGTCTGTGTAGCTTCCAAATATATTCAATGCCTGTTTTCGACAGTTTTGAAGCCGGTTACACCAGCAGAACCAACCGCCCCTGCTCAATCTGGGTCAGGTCTGGCTTCCGGGTTTTTTATGGATTCATCTCTTTCTTCATTGGAGTGGCGCTTCCATTCCTGTCTAGTATTGCCGGTTTGTTAGGTGGACTCACTCTTCCGGTCACATTCGCGTACCCTTGCTTCATGTGGGTTCTGATAAAGAAGCCAACAAAGTTCAGCTTCAACTGGTATTTTAATTGGATTCTGGGATGGTTGGGGATTGCATTCAGCTTGGCCTTTTCAATTGGAGGAATTTGGAGTATGGTAAACAGTGGCCTCAAGCTCAAATTCTTTAAGCCCAATTAA

>LOC114266906|CSS0009063.1 leucoanthocyanidin reductase-like

ATGACTGTGTTGGAATCTGTGTCCGCAGCCGGGGGCGGAGTCCTCATCGTTGGAGCCTCCGGTTTCATTGGCCAGTTCATCGCCGAAGCTAGCCTCCATGCTGATCGGCCTACGTACCTTCTCGTCCGATCGGTTGGCTCTAAAACCAACAAAACTCTTCAAGACAAAGGCGCTAAAGTTATTCATGTACTCTCTCTCTCCCTCCATTGGCAAAAATATTGAGGCTGTATTTGATTTATGAATTTAAAAATTTTGAAAAATAAAAATAAAAAATTATGTAAAATATAATTATATTTCATTCATAATTTACTTATTTTTATTCATTTTATTTCTAAATTCATAAATTAAATGCAGCCCAAGAGACGAGGGAATGTAGTTGCCCCATATAGTTAATTTTTGAAAAATAATTTTTTCTATCTATAATTTTATTTTTTAAAATTTTGTCGATGTTTCAAGAAGTGTTTTTGAATTTATCTTTCCATTGTTTTTTTTTTTTTTGTACGGGCCTCTTTGTTGTTTTATTGTCGTGGTTTTGTTTTAAATATTGGTTTTTGTTTTTTCTCATTTATCTCTTTCTCCATCTTCTTATGAATAGTAAAAAAAATTATATTTATGATTTTTGGTGAACGTTCAATCTTACAAAACAAAAATTTTGATTCCGTCCTTATATTTGTACACAAAAATACAAACATGATAAAACCCGTTTTCAAATATATACATATATTTATTGAAAATGACACTTTTTAAGTAACATTAACAAAATGTTTTTCAAAACTGTTTCAGGATATAACATGAAAACATTATGAAAATACCATGATTTTTTTTTTTTTCTGAGAAATTTTTTTCATTGTTTTTGTTTTCACAAACACACTAATTTGGATGTGATGTGATATGTAACAGGGTGTTGTCAAGGATCAAGCATTCATGGAGAAGATACTAAAAGAGCATAAGATAGATATAGTAATATCAGCTATTGGTGGTGCTAATATACTAGACCAACTCACCCTAGTCCATGCCATTAAAGCTGTCGGAACCATCAAGGTAAAAACATTTTCGCTTACGTCGTGCTTACGTCGTGTTTGGTTGGAGGATTTGAAATGAAATTTAAAAATAAAAATAAAAAAATAAGTGAAATATGAATAAAATATACCTATATTTCATTTAGTTTCACAAACTTTTTTCCTTTCTTTTTTCAAATCCCTCTCTAAATGTCTCAACCAAACACAGTATTACGGTGTGTTTAGTTAAAAGATTTAAGAAGAGATTTAAAAAATAAAAAATAAAATTATATAAAATTAAGTAATAAAAAAATTATAAAATTATATTTTTTATTCATATTTTACTTAATTTTTTTCCTTTCCTTTTTTAGATCTCTTCTCAAAACTAAACACAAGGTTAAGAAATAAAAAAATAGAAAGCAACAAAAATAGTTCTTCCAACCTTTCCATATTTGCCAGCATTCATTCAAATTCATCGAAGAGGTCATATTTTGAATAACAAATACACAACTGTGATATACTCTTCTCTTTGACGTACTTGCAAAGCTCACAAAGTCTTTTATCGAGGTTGACACGAATACTTTGGTACGTACTTGTTGCAGAGGTTTTTGCCATCGGAGTTTGGGCATGACGTTGATAGGGCTAATCCGGTGGAACCAGGGCTCACCATGTACAACGAAAAGAGGAGGGTGCGGCGATTGATAGAAGAGTGTGGGGTGCCCTACACTTATATCTGCTGCAACTCTATTGCTTCTTGGCCATACTATGACAATACTCACCCATCGGAAGTAATTCCCCCTTTGGATGAGTTCCAAATTTATGGTGATGGCAGTGTCAAAGGTATGGCTATTGTTACGTAGTTATAGCTCAAAATTAAAAAGTAGAAGCCAACTTCACAATGAACCACCTTTGACTCAGCCTTTGTTTGGCAAGTTTTGGCCCAGGCTTTTAGTTACAATATTTACAAAAAAAAAATAAATAAAAATAAAATAGATTTTTTAATTTAATAAGATAAAACATCACATCTAACTTATAAATATTATATTTTTTTTCTTACAAAAAATCAAAAAATTCATTCCTAACGTGACTTTTTAACACCAAACTAAGATAACTAAAAAACCAACTCACTTTTCAAACCAACACTTTTTCACATTGTAATCTTATTTTTTAATTTTTTAATTATTTCTTCTTCAGTAAGGGAAAAAAAATAGAAAGGTAACCTATAATGCATGACAACTTTTTCTCATCGCGATTATTAGAGCCTTGATTCATTAGTCATTTTTAACGATTTTAGTGATTTAGTGGGCGTTTAGAAAGCAACAGTGCTGCAGACCCCCCCCACCACACGCGTGGGGGCAGGGAGAGGATGAAAAAAAAAAATTTTGGGCCACTTGGTGCTGGGGTTCTCCCCATTTTCTTTTTTAAAATTTTTAATTATTTAAATTTTTATATTTGAAAAGATAAATTTAATTTTTTGTTTAATTATTGTATTAAAAAATGTATTTAAAATAATATTAAAATAAAAAAATAATTAAATTATTCCTAAAAAGTTATGCCTGACGTGAAGTTGTGCAAGTACATGATGATTGATTTGATGGCATATGAAATGATTAAATTGCTCATGTGTGTTGAAATGGTAAATTGGTGGGTTAGTCAAATATTAAAAAGGCAAAATTGTAATTTTAAATCTATCTGCGTCAGTTTCTAATAAAAAAAAAAAAAAAAAAAAAACAAAAACTAAGCTATAGTTGGACTTTTTGGCTATTTATTTGAATGGGACTAAAATAGCCTCTCAACCTCTTTCCTAAAACTGCCTTAATAAGCCTTCCTTTGGCAATTTGGATTGGATTGCGTTGGACTGTGGATGGAATTATTTTAGTTATAAAAAAATAAAATAAATTTTTTAACCTAATAAGATAAAATGTCACATCTAATTTATAATCATTACATTCTTTCTCTCACAAACAGTAAAAAAAAAGTCAATCTCAAACGAGACTTTTTAACTCCAAACTAAAATAGTCAAAAAGTCAAAAAGGCTTGTTTTGGCAACGTGGTTAAAAGCCGCGTTTGGGCGTTTGATGAAAATGCTGAACGTGGCTTTTTAGTTCGAAAACTCATTTGGGAAGCTGGATAAACGCATGAGGGGGTATTTACGAATATACCCTCCATTCACTTTATGTGACAGTGTGACAGTGTACAAGGAGCTAAAGCATGTGAAATTCTGTAATTTGCAAATGAGAAGGGGGTATTTTGGTCATTTTACACTATGCCATGATGATATGACAACATAAAAAATTATTAACTAAAAAATTCAAAAAGTTACTTTTTACCAACTTCTCAAATCATTTGTCTAAAAAATCCAAAAGCTTGGTTAAAAGCCACAAAGTCACTTTTTACCATCTTCTCAAAACAACTCAAAGCCAACCTTCTTCTCAAACGAACTCTAAATGTGCATAACTTGAATTTACAAACCAATTTTATTGATAAAACATATAAAAGTCTAGAGTGTTTTTTTTTTTTTTTTTTTTGACCGTAGTGACATTTCTTTGATAGAACTCTAAGGTACTATGTTAATCCTGTTCTGGTTGGTGGTTGTGAATTTGTAACTAAGACCCGTGAATTCATAATGACCAACCACACAAGTAATTAAAATGCAGCAATATTAGAGATTTACATTTTTAAATTTACAAATATATTTATAATGATATGGTAAAAAAGTTAAGTTTTATATCATTGAGTATGTAATAAATCAAGAATTTATATATATCTTGCAATATCACTGTGAGTACAATTTTTTTAAAGTCTCAAGTATTTTCCTTTAAAAAGATACCCATTAAAATAAAATAACCTTGAAAAACTTTATTGGTCAGCTGAAACTGAAAGAAACAAACAAACAAACAAAATAAAGAAAAAAAGGAGAAAGAGATAAAGATTGATAGAGAGAAAATCCATTAAGAGTAGTTTAACTATTATACTAAGGAAAAGAAAGAAACCAAAAAAAAAAATAATAATAATAATAATTTTATTTGGATGTATATGTATGTACACATGCTAATGTACACTACAACAAATTTCAGAAACAAAAATTTAACTATAAAATCACATTTTAGGAGTGAAAGACTTTTGTTGTCAAAAGTGTTATTATTATATTTTTAGAGACGAAATTAGAGATTTAGTAATAAATTATTATCCTTTTTAAGACCAGTTAATTTTTTCTCTAAAAGTATATTTATTAGTGACATTTAAAAAAAAAATTTACTAAAAGACAATTTTTTTTAATACTTTTTGAGACGACTTTTTGACGAATATTCTTGTGACGATGGGAATTGATTCCTAAATATTTTTTGCAACAAAATTTTTCCTTTCGAAAAAAAAAAAAAATTGATCACTAAAACTTTTATATGTTGTAATGGCATTATTGCATACATATATAATTATTTTTTGTCATTGCATATATGCCATGCATGAATTTCTTTCTAGTTAGCATAGTTATAGAAAATATTAAATATAATGGTGTATGATTTTATTTGGTATTATTGACTAATAAAAGATTAAAAATAAAATTAGGTAAACAACCTTTGTCTTGGACAAACTTAACCATGTTGTCTCGACTGTTACGATGATATTATCATATGATACCAAATATGTTTCATTTTGTTTGACTGTATCAACATTTTTTTAGAACTCTACAAACATATAATCGACTACCAAAAAATCACATAAAATATAAATAGAATAATTTGAATGAAATTGTTTATCTTATTATGAGCGTCATCATGAGATATGATGGTTAAAATTGTTTTTAATAGTTGATTGTGTTTTTGATTCTGTGTGTTCCTAGTATTCTTGATAACATCTCATAACACATGCAGCAGAATTTAAAGTCAATATTAATCAAATTACAACTTGGCATAATTTGACTTTTATTTTTGATTAAAACCAATCCAAGGCTATCAACAAGATAGAAGTGAGCTAAAGTCACTCCCAATGAAAAATCATTATGTTCTAAAAATAAAAAAAGAAAAAAAGAAAAATCATTATGTGTGTAGATGTGTATATATATATATATATAGCCTAAGAGTATCTCCAATGATAAATAGGTAAAATAAATAGTTAAAATTATAATTTAGTAATGAACAGTGTAAAATAGTAATAATATAACAATGTTGCTCTAATGGTCAAATTGTTATTTTTTAGCTTGACCAACTTTCGGATGGTCATTCGGGCCGTCCTCCAAATTGGCAACTTTAACAAACATTTGGTGGTTGTTTGGGCAGTTGTCCAAAATTTGTTGTACTGACAATTTTAGCTATTAGTAAAACTTATGTTAAATTTGATACAAAATATAACAATTGTTATTTTTAACAGTTTCAATAGCAAACCGTTACAATGCTATATTTTTTCACACTTATTATATTTTGCATTTAACAATCCATTCAATAGACTGTTAGAGATGTAACTTGCTTAAGCTTGAATGTAGGTTGCTGATTTACATCTTTGTTTTTTTTTTTGAATAATACTATATATACTAATTAGTGTATCAATTGATAATGTGACATAATTTAAGAGATTTTTTATATTCATAATTTGTATAATCAGTAAAATTAATAATAATATCATAATTAATATTAATATATTAATTAAAATCTTAATTAATGCATATAATATCACAGTTGTTTTTTTTTAAGGTATTTACATCTTTGTTTAAAATCCACTTCTTCGAGGTAATTATTCTGTACTATAAAACTACCAAATTAATATGATGAGGTCGTACTCCCAAATTCAATAAAGCAATAATGAAATATTTGATGGCTTTGTTCTGAGTGGGTATACTTTTCAACCCGCCATAAAAGAGATTGCGAGTTCAAGGCTCGTGTAATTGTTGCGTTTATTTCTTTTTCCAAAGATTTTGATTAGAGTGAGTTTGGCAATAACTTAAATAAGTTGTTTATTAATTTATTTATTTTTTTAAATTATATTATTTATTTTTATTTTTTTTAATATGTTTTTGTGTAAAAAATTAAAAAGATAAAAAATAAGCTATATCCAAATTAAAAAATAAGCTATATCAAATGGGCGAAAAAAAAAAATGTAAAATTGTCCACTTGATTGGCTGAGTGGGGGAGAGCGCCTTTTCCATTTTAATTTAATTAATTAATTTATTATTTATATGTGAACATGCCATGTCATGGTTATGTTAGGAGCATGGATGTAATAATGCAAAAACATAGGAAATAAATAAATAATAAAAAAATCTCACATATAATTTGTTTTACTTAGTTGATACTGGTGCAATATTACATCTTAATTTAATGTAAAATTAATGTTTTGATATAATGAAACAAGTTAACATAAGAACATAGAAATATACATATCTTATATTTAGATAAATTAAATTGACTGATAAGGTTAAAATAACCAAAACTATTTCAGATAGTGTGCGTATACAAGTTTTTTTTTTTTTTTTTTTTTTGCGTTTTTTGGCTGAACATAGACACGAGATTTAACGAAGTAAACATACATTGAAGTTTAAAAATTATGAAAACATTTATTTTTCTATTCACGTGATTAAAGAATTATTAATGAAAAGAGAGTTACATATTATATAAGATGAAGTTTGTAGAAACTATACCCATGGAATTTTATCAAATTTGTGAAGTTGAATGCATTTTCTGCAAATTTGAAGAGAGAAATCTGTTATTTAAACCAACCTCTTCATATTTCATTTTCTATTGTCCTTGGTAAGACATTACCACTCATAAATTTTTTCACACCAAGCACACATGATAGACTGTAATGATAATCAACATTATTTCCCATATCTATTCGCACACTTCATTTAAAATTTTATGTTCTAATTCAAATTTCATGATTCTGTGCTATGCAGCATATTTTGTTGCAGGCTCTGATATAGGAAAATTCACCATCAAAACCGTCGACGACATTCGTACGCTGAACAAATCGGTTCATTTCCGACCATCCTGCAATTTTCTCAACATAAATGAGCTTGCATCTTTGTGGGAGAAGAAGATTGGACGCACTCTCCCCCGAGTCACTGTCTCAGAAAATGACCTACTAGCAGCAGCAGCAGGTTAAATATATATATTTTTACCCATTTCAATATTGAAATATTAATGTTTGTGTTTATTTATTTATTTATTTTGTGCTGCAGTGAATATAATCCCACAAAGTGTCGTTGCATCGTTCACACACGACATTTTCATTAAGGGATGCCAGATTAATTTTTCAATTGAAGGTCCTAATGACGTTGAAGTATGCAGCCTCTACCCTGATGAATCGTTCAGGACCGTTGATGAATGTTTCGATGATTTTGTTGTGAAGATGAATGGGAAGAATTTTACAGATGAGACGGATGGAAACACTGCCCAGAATCATGTTGTTGAAGTACTTCCCATCACAATGTGTGCTTGA

>LOC114306385|CSS0016543.1  dihydroflavonol 4-reductase-like

ATGGAAGTAGTGGAAGTGAAGAATGGTTATGGTGGTGGCGGTGGAACCACCGTGTGCGTGACCGGAGCTTCAGGTTTCATCGGTTCATGGCTCGTTATGCGTCTTCTTCAACGTGGCTACTATGTCCGAGCCACAGTTCGCGATCCTGGTCTCCAATATCGCCTTCTGAAATTCCTCTCTCTGTTTCTTCCTCCTTTTTCTTAATTGTTATGAAATGGTGGTTGAATTGCAGATAACACAGACAAGGTTAAGCATCTCTTGGACTTGCCCAATGCTACCACACACCTCAGCTTGTGGAAGGCAGATCTCGATGAAGATGGAAGCTTTGACGATGCCATTCAAGGCTGTCATGGTGTCTTCCACGTTGCCACTCCCATGAATTTCGTTTTTGTCATGGACCCTGAGGTAGATGTATTTTTTTAAATTGCTCACCACTTGCATGAAAAAACTCCTCACCTGCTGCATATTTATATGAGCAACTATTTTACTCTCAAGCGCCCGTCCGAGTTAGTTAATGGATATGCTTTTGGATTTATTTTCTAGTGTGACCCAATAGTCTATATATATCTACATATTTTAATTTATTTGGATTCCACAAATTAGATAAAGCCAAGATTTGATATAAAGTTCACTAATTAGAATTTTAATATAATTACTCTTTTAAGATATAGAGGAAACAATGGTTAAAAATTTTTCTGATTGAGTTTATCTGATCATTTTCTCTCTTCTCATATGAGGATGGGTGACTTCCTTGTCATATCATGAATATTTTTCCATCCCTCTAAATTTTATAGTCATCCTAATTAAGCGAGACGTAGAGAAGAAAGGGAGCTAAGAATTAAAGGTTCAACTATTAGGGGTATTTTCTCAACTTTTCTAATTCAATTTTTCTAATTCAATGGCCGACAAGGGTAGGTTCGTTTTATTTTTGAATTGTATTGAATTCAAGATTCTGTAAAGTTTAATTCCGCGTTATTATTATGGTTACATGTATTATTAAATCTTACATATGCTTCCTTGTTATTAATCAGACTTTAAATTACTCCCACTAATTACATTGTTTATGTGCTACTTTCAACCAATAGCTTAGCGGAAAATTTTACACCGAAAACAATACTGGACGCATGCGCAAAAGCAAAATACATAAATTTAACCGTTCCAGTTAAAATGAAATAAAATGACATTAAGCCACACATATATATTAAAACAACCTAATTTTGTGTTTACTTTAAACATAAGCAGTTCAATTACCACAACAAAATCATTGCGAAAAATTCATCACATAGAATCCTAATCCCATAAACAACGCCACATGGTAGGACTAACTTCATATATCCAAAAAATGACCAATGAGTGAAGGCCTTTGTGCTCATACCTAATCAGATTATTTCTATTGCTAGTCTGTTTCAGTACCACGACTGAAATTGAAAATTCACAATGACTTGAAAATTGAGATTATTGTTGTTGTTGTTGTGGGCAGAATGAGTATATTAAACCGACGGTAGATGGGGTTTTGAACGTAATGAGATCATGCTGCAAGGCCAAGACTGTGAAGAGGATCATATACACATCAACCATGGCAACTATTGAATACCAACAGAAACCCCCTTCCCAATATGATGAGAGTATTTGGACCGATGTGGATTTCTGCAGGGCTCGCAAGATGTTTGCATGGGTAAGATAAGACACAAACCCTCTCACATGAGAAGGTTGATCTTAATTTTTTTGAGTCCTTCACTCATGTGAGAAGTTTGTGTGCAATTTTAGGATTGGTCATGTTCCTAGAAGAACTGTTAGTTGGTAAGCATTTCAAAGGAGAAAAGTTATGGAAACAGAAAATGTGCATAGAATAGTACATAGAAACTCTAGCGACCGTCTTCAAAGTTTCGAAGACACTTTTATTTTAAGTATTAGTTAAGAATATTTTTAAGTTATGTAAGTTTGTCTCTCTAAATTTTTTTGAAAAATTTAATACCCATTCCATTCAATTTAATATCCCCATAAATTAGCATCCAGGGCCGGCCCAGAGATTTAGGGGCCCAAAGGTAAACTTTGGAAATGGAGCCCTCTTGCATTGAAAAAAAAGAAAAAAGTTCATGGATAAAACGATAAGTTATAAATTTTTTATTTGTTTGTAGCCACAAAAAAATTTACTTGCACATATAATCATTAAAAGTATAAATAAAAATTTAATTTCATATATTAAAATAGGAAACAATCATTACAAATACTTAAAAAAAATAAAAATCTAGTCTTGTTGCTTTTTTTGGGGCCACATGTTCAATTGGGCCTATAGTATTTGGACTTTTTTTTTTAATTTCTAAAACACAAAATTATATTTGGAGGCCTCTATTTTCAGTCCCAATTTGGAGGCCCAAGACGTAGACTTCTCTTGCCTACCCTCACGGCCGGCCTGTTAACATCCAAACTACTGCTACCAAAAAGAATGACAACTCCAGCAATACTTTCCGACAGGGTACAAACATTTGACTATATGAACCCAGGTTGTTTTAAACAAGAAAACTTCATTGTTTGCGGGTCAACTTGTGTAGAACATTAGTGAATTGGAAGAAAAATCATACCCTGCTTGTTGATGATAAAAACTCAATGTAGAAGAAGACCGGACCCATTCCACATACGAGATCTACAAAAGCAACCATCATTAGTGTTTAGTCCACCATTATAAAGAGAGTATCCACGGAGAGGTTGTGTAATGAATGGTGGCATACCTTAACCTTTGGTGTCTAGGTATATAATATAGATGAGTTTATCTTGGTGAGCAAGCCATTCTAAAAGCTCCCATCCCATGGATATGATGTGGAATATTTCCTTCAGGCCCTCCAATCGAGCTAGATATTTGATAGAAAATACCTAGTCGAGTAACGTCCCATATCCCAAGGATATGCTATGGAATATTCCTTCTAGAGGTCTTCCAAACAATCTAGCTAGGTATTTCATAGAGAATGGCTAGGTAAGTTAGGACTTATTCCAATAATGATTTTCTCATCTAAGGGAAACTAATCTATATACATCTAGACTCCCAAAGGTTGAGGTATGTAATTATTCACTACTCAAACTCTATTGGTATCTCATCTTTGATGGTGGATTAACTGAAGTGTGTGAGTTCTCCCGACCGAGTTCTAATTACTTTTGTGCATCTCACACTCGAGACTTTTCCATTGGGTTCACATCATCAAACAAGTAAGTGTGATTTTTCATCCCCTACAATTAGCTACAAGGCATCAACTTTTCCGTCTCTGACTACAATTCAATAATCTTACTTCTGATTTCGTTGTCGAGTAGATGTATCTTGTGGCAAAAACAGAGGCTGAGAAAGCTGCATGGAAATTCGCAGAAGATAACGGCCCTGATCTCATCACCGTCCACCCCTGTAGTGTGATTGGACCATTCATTACACCATACAAGCCTCCCTGCACTTCAATGGCACTTGCCTTAATCACTAGTATGTTTTATTCTCAACCCTATATTTTGGGTGCCACCACAGCTTACCTGTTAATAACTTACATACCTTACGGTACATGTTGTGTAGAAAATAAAGCTTTTTACCCAATGCTGACCCAAGGACATGCCGTACACGTGGATGATGTGTGCAACGCTCACATCTACTTGTTCGAGCATCCACAAGCTAAAGGAAGATACATTTGCTCATCTCACTCTTTTACCATATTCGATCTTGCTAACTCATTGAGCAAGAAATACCCGGAGTACAACATTCAGACCAAGTATGTGAAAGAGAAAGATAGTGGTTTGTTTTGTTTTGTTCGTTTTCATTTACAATTCTATGTTTTCAAAAACAAAATAGTAAAAGTTCAAAAACACCCATAATATTCATATCGTTTTGCTTTTGTGCAAAATTAATTTTGGTATTCCTAAAAAGTGGGTTATTTTTAGAAAATTTTCAAAACAGAAAGAAAAAAAAAACGAATAGGCCAAAAAATAATATGAAAATGAAGTGTGTCGTGTAGATTTCAAAGTTGGTTTGGTGTGAGTCACATCATTTGGTCCGAATGTTTCTTTTTTATTTTATTTTATTTTTCAAGAGTGCATAGCAATAGTATTGCTTGTGCTAATTTTTTTTATCATTAGTTCATATCTAACTTCATGTTTTGAGCTTTGCACAGATTTGAGGACATTGATGAATCATTGAAGCCTATCCCTTGCCCATCTAAGAAACTGTTGGACCTTGGATTCAAATTCAAATACAACTCTGATGAGTGTGATGCAGGGGATTTGTGTGCTGAAGCTATTGAAGCATGCAAAGAAAAGGGGCAGATGCCATCTCTATAG

>LOC114308069|CSS0022726.1 lysine histidine transporter 1-like

ATGGGAACTCAAGCTCCAAATGATCAGAACTATAACCCCAACAATGTCAGCCTCTCTCTCTCCCTCTCTATATATAGCTATGTCTCTCTGCTTTTTGTGTATGTATGTTGATGTGTGATTTGATAGTGTTCTGCTTCTGATAATGAAGTCAAGTTAAGATTTGTTATTGTTGCTTCGTTTGTTGTTGTTGTTGTAAGTTTTGGGATTTAAATCTTCGATTTTGATTTTGGTTGACTTGAAAAGAGTTGAAATTTTAACTGTGTGTGTGAAGTTCCGGTCATATTTGACAAGATTAATTAATTTCTTCAGTTGTTTAAAAGGTAGATACAAAATTATAAATATTGTTCTCATTTTTGATATTTTCTTGGGAAGACGTAAAAATAAATTAAATGGTCTAATTAGAGGAATTCAACATTTACTAGTAATTGTCAAAAGGATTTTTTTTTATATATAAAAAATCATATACCAAGAAAATTTATTTAAAAAAACAAATATTACAATTTTCCAAAAATTATAATGGTCCTGATTCCAAACTATAATTTTTTTAAAAAAAATTATTTTTCTGAGTAATTTTTTTTGAAATTTTTTTTTTTTTTGGATAGATTCATAAGTTATATACCAAAAAAATAAGTATTACAGTGAAACTCGTTACACGCCCAGAGGCAATACACCGTTCATGAAGGAAACAAACATGCCTAACTTTTATACAAAATCCAACAAGCAGATTAATATACAAGGAGAAATCAAAGTTGGGCCAAGCAATAATGAACGGACACACTTACGATTTCAAAGGAGTCTTACAATTTGATTTTTAGAGTTGATAGAGAGGCTACCCTCTTGAGTTAGGTTAAGATGGCAATTCTCTCAAGGAATTTTTTTTTGTTTTCTACCTTTGAAGATGAATTGTCTATGAAATAACGAATGTTCCCTCTAAAATGGATTGAGGAGCTCTTTTAACATGTGAAAATCGTGGACTGACCCATTTTTATTTTTTTTGAATCCTAACACGGGCTTGCTATCAAAAGTCTAAAGAGGTTCATTAGTGTGGGCCCTTTTTGTTGGGAAAGACGTTGAACCCACGGCAATTTCGTTTTCTATTCCTGATGTATCACAACCACACAAAAAAATTTGGATCCAAGCTTATCTTATAGGCAATAAATAGTAGCCAAGTTGTCAAGTGGGTGTTTGAGAAGTTGCTATTTTAGTAGGTTATTTAAGTTTTTGACTATTTTAGTAAGTTAAAATTTTTGTGTTTAGAAAGATATATTTTTTTATAGTTGGATATGATGTTTGTGAGAGTAAAAGTAAAATGATTATGGATTTGATGTATTATTTTATCTCATTAACTATTTTAGTTTATTTTGTCTTTTTATGAATTTTAGCCAAAAATTAGCTAAAATCTCACCTGCCAAACACCACCTACTAACATAACTGTGACTAAAAATCAAGCTACTATGATTTCGTTTTTATTAAAATTTAAATAAAGATTAATTTAACACAACCGTTCTGGACTTCACCTGATTGTCTTAATTAAGGGGTACTAGGCTTTCTTTTTTTTTTTACCATAATATTTTCAAAAAATAAAAAATTAGAGAAACAGACTCTGCACATGCCTTGTGCTTGGCAGTGAAATTTTCCACTTATTATGTAATGTCTAATATACTCGAAAGATTAATACACACCGTAAAAATAATAAATAATAAATAATAAAAAAAAGATTATTATACATCTAAATGTCTATTGTGACTGGTTTATGGTTACCCAGATTATATTAATGATGTTTTTTATTAAAACTGCGTTTTCTTTTTTTTTAATCTTTTTCTTTTTTGGTTGAATTTATTTGAAACAAACATGCTACAGGTTGACACACAGTCTGAGAAGGAGAAGGCGATCAATGATTGGCTTCCAATCACTTCATCAAGAAATGCAAAGTGGTGGTACTCTGCTTTCCACAATGTCACCGCCATGGTTGGAGCTGGCGTCCTCAGTCTCCCTTATGCCATGTCAGAGCTCGGATGGTATGCACTATGCATAATCATGAATCATGATCACCAAACAATTTACTTTAATTCATTAATAATTTTAACTTAAGACAAGCATTTTTAATTAGTTGAAAATAATTTCAATGAAAATCAATGTATTGTGGGGTCTCACTGAATATGTTTGATAAAGCGCAATGTTATTAAAATGTTCATTTTAGACCATTCATTCTTGTAAAGGGCTACTTTTTCAAAAAAAAATTTACCATAGAAGTTTTTATTTTCTGAAACTTTTTTAAGATTTTCGAATAATTGAGAGTCTGAGACATATGAATTTCTTTAAATTAACCATTTTCATTTTACTTCTCAATCAAAAAAGAAATTTTTATTTTTATTTTGGAAATTTTACAGTCAAGTAACTGAGTTATACTCTTTTGATTTTCAGGGGTCCTGGTGTGGCTGTACTGGTTATATCATGGGTTGTCACTTTTTACACCTTGTGGCAAATGGTTGAAATGCATGAGATGGTACCTGGAAAACGTTTCGACAGGTATCATGAGCTTGGCCAGCATGCATTTGGTGAAAAGCTTGGTCTATACATTGTGGTGCCCCAACAGCTTGTCTGTGAGGTTGGTGTGGACATAGTCTATATGGTCACTGGAGGGAAGTCACTCAAGAAATTCCATGATTTGGTGTGCAAAGATTGCCAAAATATTAGGCTCACTTTCTTCATCATGATTTTTGCCTCTGTCCACTTCGTGCTCTCCCATCTTCCCAACTTCAACTCCATCTCCGGTGTGTCTTTGGCTGCAGCAGTCATGTCCTTGAGGTACAGTCCCTCTTGCTTTTCTATTCCATTTTATGTTACATTGACTCAGGTACACACAGGTATGTGTGTGAGTGTCTAATTCATCTAATTATCATGTCAAAGGACACGAGAGTCCATGAAACATAGTTTTAATGTCCAAAATTCATCTTCTCATTGATGCCATTTCATCATTGTTTCCCAATAATTTTATGTTTGCATGAAAAAATATAATTAAACCTAGCAGTCACATGAAAATTCGTGGAAAAAAAAGGCAACCCCTAATTTTTATTAGTGCTCCAATGCTTAATATTGATCGGCAAGCAAGTACAGCAATATCTTTGATTTCGATGTCCTGCAGTTACTCTACCATTGCATGGGCTGCTTCGCTTCACAAGGGCGTGCAACCGAATGTCCAATATTCATATACAGAGAAGAGCACAGCGGGAAAAGTCTTCAACTTCTTCAGTGCCTTGGGTGATGTGGCTTTTGCCTATGCAGGCCACAACGTGGTCTTGGAGATTCAAGCAACAATCCCTTCTACACCCGAGAAGCCTTCCAAGGGCCCCATGTGGAAGGGAGTGATAGTTGCCTATATCGTCGTGGCCTTGTGCTACTTCCCTGTGGCACTGATCGGTTATTGGACATTTGGGAACAATGTTCAAGACAATATCCTCATCTCATTACAGAAACCCACATGGCTCATTGCAATGGCTAACATGTTTGTTGTTATTCATGTTATTGGAAGCTATCAGGTGCAAGCCATAATTTCTTGATTTGATTAAGTTGATAAACTTTCTTTGGTGGATCTCTATCTGATTTTTTTTTTTTTTTTGTTGATTTAGATTTATGCAATGCCGGTGTTTGACATGATAGAAACGACTTTGGTAAAAAAATTGAATTTCAAGCCCACTTTAATGCTTCGCTTTGTTTCTCGAAATATATATGTGGGTAAGTGAATTGTACTCCTCATCTTCATCCAATTACTGCATTATTTTACATGAATTTGTGATTTTGTCAAAAGCAAAAAGTTACGTTTTTCCAAAGTTAATATAATTTCTTTTTCTTGCAGCATTCACAATGTTTGTTGGCATTACGTTTCCTTTCTTTGGTGGTCTTCTTGGATTCTTTGGAGGATTTGCATTTGCCCCTACAACATACTTTGTAAGCAACTCTCTGATCTCTTTTTTATTGAAACCATATAGAATGAAGAAACAAACATCGTATGATTGCTTACTCTCATTCTTGCTTTTTCTACAGTTACCATGCATCATTTGGCTTGCTATCTATAAGCCGAGGAGGTTCAGCTTGTCTTGGGTTACTAACTGGGTATGACTGAAACTTAAAATTAATTTCACATTGCTTTCATTTTTTTTCCTTGTGGTTTGAAATTTAAAGCTTAATTAATTTTGATTTGGCTTCATCAATTTCAGATCTGCATTATACTGGGTGTTCTTTTGATGATCTTGTCGCCTATTGGAGGGCTAAGGCAAATTATAAAAGCTGCAAAGGACTATCAATTCTACTCATAA

>LOC114309343|CSS0022212.1 flavonoid 3',5'-hydroxylase 2-like

ATGGCCCTAGACACAGTCTTCCTGCTTCGTGAGCTCTCTTTTGCAACTCTCGTCATCCTCATCACTCACATTTTCATGCGCTCCATCCTCACTAAACCCCTCCGTATGCTCCCCCCCGGCCCAACAGGGTTGCCACTCATTGGTGCCATTCCCCACCTAGGTTCCATGCCACATGTTGCTCTAGCTAAAATGGCTAAAATATACGGACCCATAGTCTACCTCAAAATGGGCACTTGTGGGATGGTGGTTGCATCGACTCCAGACTCAGCTCGAGCCTTTCTCAAAACCCTAGACTTGAATTTCTCAAACCGCCCACCTAATGCCGGTGCAACCCACTTAGCCTACGGTGCTCAGGACATGGTTTTTGCAGATTATGGACCTAGATGGAAGTTACTGAGGAAACTAAGTAACTTGCACATGCTTGGTGGCAAGGCTCTTGAGAATTGGGCTCAGGTCCGATCCTCTGAGCTTGGTCACATGTTAAAAGCTATGTACGATTTGAGTTGTCGCCGTGAGCCAGTGGTGGTAGCGGAGATGTTGTGTTATGCAATGGCAAATATGATCGGACAAGTGATACTTAGCCGACGAGTGTTTGAAACGAAGGGATCGGAGTCAAATGAGTTCAAGGACATGGTGGTGGAGCTCATGACCACCGCAGGCTATTTTAACATTGGAGATTTCATACCATCGATTGCGTGGATGGACTTGCAGGGAATAGAGCGTGGGATGAAGAGGTTGCACAAGAAGTTTGATGTGTTGATAACAAAGATGATAGAGGAACACAAGGCTTCGGCTTATCAGCGCAAGGAAACGCCAGATTTTCTTGATGTTTTCATGGCTCAGCAGGAAAATCCTGGTGAAGAGAAGCTCAACACGACCAACATCAAGGCACTACTCTTGGTATATGCCTCTTTAGCTCTCCTTCAATTCAACACACACTTATATAGTTATGGATATCTATATATTAATATTATAATGCTGGCTCAAGACTTTTCGTACCTTCGACGAATTTTTAAAAGGTTCTTTTTATATTTTAAAAATATTAATTATTTTTTCATAGATAATTATTATTTGAAATTTATTTTTTTCTTCTTGTTAATAAAAATTTAATTTGGATCAAATCGATAAGGGTGATTCGATAGGAAAAATTAATGTTCACAAATAGCAAATTAATCAAGGCAATGTGGTTGGTGTCCTATGTTGTAACATATTTAAAGATTTCTTTAGAGGTTGTAATGTGAAGAAATAAGTACAAACAAACTGAAAATTTGATTAATAACACCTAAACTTGTCACTTAATTATAAGAATTAAATTACAAATTTCATAACATTATAATCAATAGGTCCAAATTAAATTACAGACCCTGAATTACTGATAATCTAGTGTTAAGTACAAGAGCTACTCGTGGATCCACCACAGGCTTAGAGGAGTACTTTAATGACCTAAACGAAGGGGCCGCACTTACATTATCATGGAAAAAACCTCTCTGTGCACCTTAAAAAAGTCGTATATAATATACACTTTATACATAATTTTATGGGTATATTATGTATGAAAATTTAAATTGATTTGATATTCATAATTTTTTTATTGTATTGTGTTTGTGTCCAAAAATTAGATGATTTATTTTTTAAAAATATTATTATCGTCTATTTTTTTGAAAACAAAATGAAAATATTACTATAATGGGAATGACTAAATTTTTTTCATTAATAATATACACTGAGGATCCTACTAAATGAACAACTCGAATGTTAAAACGGAACTCATGATATGTTCCATGTGCCCTTCAAATTTCCTCAAATTTATATGAATTTTAATAAAAAAGATTAATACAAAACGCATCAAAGTAATACTAAACGATGTTAATTAGATTGACACTTAAAAAATAAAAAAATAAAAAAAATAAAAAAAAAGCTAGTAAGTTACTAACTAGAAAACCAAATACAAAAATTAGAAGATTTATTTTTAAAAATATTATTATCATCAAATTTTTTTTAAAAAACAAAATGAAAATATTACGACTATCGGAATGATTAAATTTTTTTTCATTGATGATCCTACTAAATGAACTACTCGAATTATTCAAACGGAATTCATGATATGTTCCATGTGCCCTTCAAATTTTCTCAAATTTATATGAATTTTAATAAAAACACATCAAAGTAATATTAAACGATGTTAATTAGATTGACACTAAAAAAAAAAAAAAACTAATCATCTTGTAGCACTAAACCCTATGAAGCACGTATACTTCAAATAGAGTGACATATTCGTATCGTATCCGTATCGGATACCGATACTCTTTTTTTTTTTTTTTTGATCGGCAAAGAAAATCTTTACCAATACTCCTTGCATACTCACGGACACGTATCGGATACTTATTAAAAGTATAAGATATTTCTTAATTATTTTTTCATTAATTTTGGAGTATACGATACTTAATTTTGAAGTATCAGATACTCCAAGTCATGTTTACTTGTTTTTTGAAATTAGAATTGTAACATTTCTTGCATAGTAATATAATATATTATAATATAATTGCAATTATAAATATAAGAATATTTTTCTAATGCTTTGTTAAATTCTTCGATAAGAAGAGCATAGTAAGTTAAATAATAGAATTCATCAAATTGATATATCTTAATTATTTCATGCATATCCCCTATAACTACATAGAAAGGAAGAATTAACAAGAGAATATAATAATGGTAAAATTAAATATCAATAAATAAATAAGCAAGTAAACTTGAATATAATATTTTTCACTAGTGTCAAAGGAAAAAATATAAGGACAAGTTGTGAATTTTTCTATTCTTATAATATAAATATATTAAACTTGATATTGAAATGAAATATTTGCAAGATTTATTAAAAATTTAAAAGTAAATAAGCTACTAACTTAAATTAATACAAAATTCTTAAAGGTTTGTATTTTACATTATAAAATTATATTGTGATTTCTCAACGTTGTTTAGCTTATGTGTAACTCACTATTAATAATTTTAAAAAATATATTTTTCACAAACGTATCATTACCGTACCGGTATCTTATTTTTTAAATTTTATCGTATTATCGTATTTGTATCGTATCATATTCATATCTCGTGTCTATATCTATGCTTCGTAGACTCAACCAAAGCTAGTAAGTTTCTAAGTAGAAAACCAAATACAAATCACAGATCACTATCTAACACAACTCCATTCACCACACATATCTTCACCTGAGAAGCAAAGACCATGAAACCACTAAGCAAACCTTGACAACAGTAGCCTTTAATGAGCCCAAAAGCTCATTGGATAACAACCTTAGCATCCATACACGATAACTAACAATTATTATTATTATTTTTTTTTTTTTTTGTAAATTATAAATATATCCTTTGAGATTTATTTATATTATAGAATACTTCTATAGTTTGAAAAATTATAAATGTCCCTTTGCTAGAATATTTAGAATTAACTGAAAAAATAAAATTTTTAATTGTTCTCCTATACAATGATAAAATTACCTGAATTATTAATTGAAATAAGTTTTTAACGTTATTAAAAGCTGTTAATATTTACTTGATGATTTATTAATAAAATGTTCATTTGTTATGAAATTAAAAATTTTGAGGAGCAGGATGTAATTTATAGAATCACAGAAGACAAATCATATTTTAAACAAATCTCAGGAGATGTATTGTAATTTTACCCTTATTTTTTTGACCAAACGTCCTTTGTCTCTAAATCATTCTCTTCTTACCTCAAAAAAAACTCACAAATTATTGAATCATGTAACAATTTTGTAGTGTGAAAATCTGGAGAAGTAAAATTAAAAAAAAAAATTAAAAATACACCATCTATTTATTTTTCTATCTCCTAGTGTAAATTTCCATGATTTTGACTATTTTTACATTTTTCCTCGTTCATCGTTTGGCTAGAACTTATTCACTGCTGGCACCGACACATCATCAAGCATAATAGAGTGGGCGCTAGCCGAAATGTTGAAAGACCCAAAAATCCTCAACCGAGCACACGACGAGATGGATAGGGTCATCGGTCGAAACCGACGGCTACAAGAATCCGACCTACCCAAACTCCCATACTTACAAGCCATTTGCAAAGAAACATTTCGAATGCACCCTTCAACTCCTCTCAATCTTCCCAGAGTCTCTGCCCAAGCTTGCCGAGTGAATGGTTATTACATTCCAAAAAACACTAGACTCAGTGTCAACATTTGGGCCATAGGCCGAGACCCTGATGTGTGGGAAAGGCCATTAGAGTTCATTCCTGATAGATTTTTGAGTGGAAAGAATGCAAAGACTGATCCGCGTGGGAACGATTTTGAGCTGATTCCATTTGGAGCTGGAAGGAGGATCTGTGCCGGTACTAGAATGGGGATTGTGCTTGTTGAGTACATTTTGGGCTCTTTGGTTCACTCTTTTGATTGGAAATTGGCTGATGATGGAGTTGAGCTGAACATGGATGAGAGTTTCGGACTCGCTCTGCAGAAGGCTGTGCCTCTTTCGGCTATGGTGTCTCCTAGGTTGCCTCCAAATGCTTATGCTGGTTAA

>LOC114310255|CSS0015313.1 glutamine synthetase nodule isozyme-like

ATGGCTCAGCTTTCAGATCTCATCAACATGAACCTCTCCGACTCTACGGAGAAGATCATTGCCGAGTACATATGGTGAGTCTGTGTTCCCCGTCTGTTCTGTTCTGTTTTTTTTTTTTTTTTTTTGGTCTGGGTTGTCGTTATTGTTGTTGGGTTTGCTTTGTTTTTCCACCCATGTTCTGTTTCTTCACTATTGGTGGGTTTGCCCTGTTTTCTTTGCTTTTTTTTGGGCTTTGCTTTTCGATCCAATGTTGCTGTCACAAATGTGTCGTTCTCATGGCATGCGGTTTCTATTCCTCGCGATCCACCAATTTTTTTTTGCCATACATCAGATTTATTTTATTTTATTTATTTATTATTACTATTATTTTTTTTTTTAAGTTTCCACTGCCATTGATCTTTGTTTTTAGTCTTGGTTATTTTTGTTTTGTTTGTTTAGCGATCTGGGTTCATACCGTTCTGGTAGCAAATGGGTATGCTCTGTTTTTTATTTGTTTCCCCTTAGATCAGTCTTGTTGCAAGATCTCACCTTTTTGTTATCAATCATTGTTTTTTCATTTTCATCTTCATTGTTGTTCTGTTGTTAGACCATCTCCAACCCAGCAACTTTTCTTTCATCAAATTTAAATTTGATGAAAAAAGTAACTTTTTGTTGCTCCGATGTTTCATCACACCAATATCAAATCCAGGATATGTTAGAGCATCTCCAATGGTGTACACAATTCTTTTTAATGCCAAATTTAAGTAAAAACTCTATTTGATATCAAGTGCTTCAAGGGAAGATTTTGAGATTTCAGACCGGCCAGATGTCCGACTGGTGGACTGGCCAGTGTATTTTTTTCAAAAATTTTTTGCCTAGAGGCGGTCATTCTCTAGTAGGACATCCAGTGACGAAAATTCAAAAGCTCTGAACAGTGATTTGATGAAAGTTCCCAGCGTCCTCAATTTGGTGAAGGAATTTTTATATCTTCAATTTGATACTCATTTGTTGAAACTATAAAATAGATGTTTTTCCATCAAATTTAAATTTGACGACAGAATTAATGAACCATTGGAGATGGTCTTACAGTTTTTATTACAGTTTTTATCAAATCGAGGATGGCTGTGGTTTTCACCAATTTACTGTTCAAAGTCGTTGGAGTTGCAGGTAGTACAACACCATCGGATGTCCAGCTGGTTGTCCTTCCGGATGACTACTTCGGTGAAAAATTGCTCACAACATATTTCAGATTTGATTGAATATTATACTGGCCGGTCGACTGTATGCCTGGTCGTCCTCCTGGTCGATCAATGATTTGATGACAGTCCAAAATTTTCCGATGGAGCGCGCGATTTTTATTCAATTTTTTAATGAGCGATTTTTATTCAATTTCTTAATGAAAAAAATGAGTATACGCTGGAGATGCCCTTAGTCCATCTAATTTTCATATGATAACTCCAATTTTTGTACTTTTGGGTTATATTTTCATTGAGTAACCTTTTCATTAGCAATAATTCTCTGTTCTTCTGTGTACAGTGCCCTGTTTGGACATAATCAATTTACTTGTACTTTTCTTTTAATCAAATGATTCATGAGATTTTTATATATCTAAGGTTGTTTTAATCAGTTGATTCATTACATTTATATATCTAATGTCGGCATGTGGATATTTCACTAATATTGTTTTAAGGAGAAATCTACAACGTCCCCCCCCTTTTTTAAAAATTTGCTCATGTTCAAAAAATGTTAATATTTTTTATTACTTCCATGGAAGTCATTAATGTTTGTGGTGTGTTATGATATTTGCCATAAAATTACAAATAGTGAATTTATGGTTTGTACCCTAAGGTCATGTTTGTTTGGGTGGATATGAGCTTGGGATTGGATTAATAGTCCAATAAAATTATTAATCCGATGTTTGGTTAAACAATTGAAGTGCGGACTAATTATTTTAAACTTGACTATTAGTTAAAATAAGATACATAATCCAACGGATTCGTCGTCCTAGGACTATTAGTCATGAGAATGCTAATCCAGTCCGTCCAAACAAACATGGCCTAAAGTTATGAATGGTGAAGTTATGGCATGTTATATTTTGCCGTGATACCTCGTTATTGTTGCCACATTTTGTGGTGTTGAGGTGAGAAAAGTTATGAAGTACACACCATATACACCCTTTTGGGGTGTACATTGTAGCATCTCTTTCATTTTAATCATCTCTTTAATTGTATTGCTGTGTTATAGTCATCAGAATGTTTGCACAATTGGATACAAATTTCAGTTATTTCATTTGGTAATCGAGTGTTCATCTTGTTTGTAGATGAAACTTTGTCATAGTCTTCTCTCAATATAGTATGCTTCATGACATTTTTTCTTTATTTGTGGTTGCCTTTTTTTTTTTTTTTGATTGTTAAAATCTATAATTATACAACTTTGATTGTTAATTTTGAATCGTTGAATAGGATTGGTGGTACTGGCTTGGACCTCAGAAGCAAAGCAAGGGTAATTGCTTTTTACTTATTTAAAATTTGTTTGTCAAGTTGTTCTTGTAGTTATTGTTCATCTTCTTGATTGGTTTTAGTGATTTGTAATATATATGCTGTTGTTTATTCAGACTCTTTCTGGACCCGTTAATGATCCCAAAAAGCTTCCCAAGTGGAATTATGATGGATCTAGCACAGGTCAAGCTCCTGGAGAAGACAGTGAAGTGATCATATAGTATGTTATCTTGTTTTCCCAATTTTCATATCTTCACTTTAATTTGCGCTTGCTTTTATGGTCTTATTTAGTCTCTTTCTAAATTTAAGTTTTACCCGATAAATGTTACGAGTGCTTACCTTTGTAATTTGGTGATTATGTATCAAAACAGCCCTCAAGCAATTTTTAAAGATCCATTCAGGAAGGGAAACAATATATTGGTGAGTTAGTGCATTCATGCAGTTCATTGGAATGGAGTAGTCATTCCATCAGAATAATCAGAATAATCATCTCATGTTTGGTTCGGTAAAAAGGAAAGGAATTTAACATTGGAAATAAAAGCACCATTCACCAATTTAGGAGAATTGTCATTCCGTTGGAAGGCCAAGGAATAGTCATCCCATTCCTTCCTTTAATCGTTCCAATGAACAAAACATTTCCTCAATTTCATTTGGGTTTTGGGGCACTTTCATTGTGAAATCTCAAACGATGTGTTAAAAAAGTTTTGTTGTGCTATTTTTCTCATTAATCATTATTGTCTTAGGTCATGTGTGATGCTTACACGCCTGCTGGTGAACCAATTCCAACCAACAAGAGGTACAATGCTGAGAAGATTTTCAGCCACACTGATGTTGTTGCTGAAGAACCTTGGTAAAGGATTTCAAGCTTTTCACAATATTTTGTCAACACTTAATTGTCACTGTTGTTTGAGTATCTTTGTCCATTTAACTTTTGGAGACTGTAATTTATTTTGAATTTTAATTTTAAATTAGGTATGGTATTGAGCAAGAGTACACCTTGTTGCAAAAAGATGTTAAGTGGCCTATCGGGTGGCCAAAGGGAGGTTATCCTGGACCTCAGGTAATGAAATTGATACTTTTTCCGAATATCAACAATTTCATTTGTCTATTCTGTCTAATATTTAGTGATCATGTCCTCCAATTTTAGAATTTTCTTGAACCTGGTAAATTAATTTTGTCTGCTGGTTTAGCCCTTGTGAACTTTTGGATACTGACATGCTTTGCAACCCCTTGTGTGTGTCAAAAAGGATAAAATTACATAATATTATGTTAGCTTTTAATCTTTTTGAGCCACTTGTGCAATGCTATTAAAAAACATATATAAAGAAGTCCTTATTATGCACCTTTTTAATTTGCTGTTTATTGTGCAGAATTCAATTGCATATAATAGCAATTCTTGTAGATGCTCTGGTGAATATTTTTGTGGCTATTTCTTTTTGCCTAAGACTTATAGTTGAACCTTTTGTTATCGCAACATAGGGACCATACTACTGTGGCGCTGGTGCCGACAAAGCTTTTGGACGTGATATTGTTGATTCACACTACAAGGCTTGCCTTTATGCCGGGATTAACATAAGTGGAATCAATGGTGAGGTGATGCCGGGCCAGGTAAAATTAATAATTTTCAATTGTCTCCTTATGTTTTTTTTCTCCTAATCTGAATTCCTAGTATTCATCAGTGCAATAGATAGTTAAAACAAATATTTCTTTGTACTATACAGTGGGAGTTTCAAGTTGGACCTGCTGTTGGCATCTCAGCCGGGGATGAATTGTGGGTGGCTCGTTACATTCTAGAGGTAGAGTACCTAATTGAAATACGTACTACTTCTAACCACTAATACTTTTTTAATGGTGTATGAATATGAGTAATACATGTGTTATCTAAACTGCTTTTATTTTCTGTTATTAACAGAGGATCACTGAGATCGCTGGGGTTGTGGTATCTTTTGACCCCAAACCTATCGAGGTTAGATTTTTCATGTTTACTTTGCTCTTCATCATTCCCAAGCATCTTAATTTTCTCATTGTTAAATTTACTGAAGGAGGTCGGAACATCTGGTTTTGACACTTCTTGAGCACAATGAAATTGCATTTACCTTAATATCCCTATAGCTATTGCTATTATTGCCATCTTAAGTCGAAATCATGGTTTTTTGCACACCAGGGTGATTGGAATGGAGCCGGTGCTCATACAAATTACAGGTAATGGATGAAATTTCATATAAGGGACTAATGGCACTTTACATATTTGGAATATTTGATGAATTTTAATCCTAATTTCAGCACCAAGTCTATGAGGAAAGATGGAGGTTATCAGGTCATCAAAAAGGCTATTGAGAAGCTTGGACTGAAGCACAAAGAACATATTGCTGCATATGGAGAAGGCAACGAGCGTCGTCTCACTGGACGACACGAGACAGCTGACATCAACACCTTCTTATGGGTAAATAATTATTTTTCCGCTTCAAATATTCCAAATTTGTTATTGTTTTATATATTATTTAGCTTGCATCATTTAGATCAATTATGAAACAATGCTTAGCGTTCACAACAAACATTCCAAACATTCCTTGATTCATTCAATTGCAGTCATTTTGATTTTCTTTTGGTCCCCCATTGTAATTTATGCAATAATAAATATGCCTTTAGTCTGAATTGTAAAATTTTCTTTTTTCTATGAAAATTCAAGAGCACATTTTTTGCTTTTGCATCCAATCTCTATGATTGATTGATATACCCTTTCAAGCCCGACGGACCACATGAGGATATGATTCTCGTGATTTTTGTTATCATGGTCTAGTTTTTATGACTAGTACTCCCTCCGTCCAAAATTGTTTCATTATTCCAGATATTACACAAAACAAAAGTGATTTATATTTTGTAATTTATTATTTTTCAATGGTTTTAAAGACTTATCTACAAGAATTGAGATCTTTTAATTTGTGGAAATTATTTTTTTAACAAGTCATTATTTATTGACCCACCAAAAATGGACAAATAATTTGAGACTGAGGGAATAACTATTGCAATGGACCTAGCTCATGTTCGTAATACAAAGCAAACCTGAGAGACTTTACTTGTGCTCATGCTTTATTAATTTTTTTCCCACCAGGGGGTTGCAAACCGTGGTGCATCTGTTCGTGTTGGCCGAGACACTGAAAAGGCAGGGAAAGGGTATTTTGAGGACAGGAGGCCTGCTTCGAACATGGATCCATACGTGGTTACCTCCATGATTGCAGCTACCACCATCCTGTGGAAACCATGA

>LOC114311274|CSS0033978.1 transcription factor MYB4-like

ATGAGGGTTCCATGCTGTGAGGAGATGGGGATGAAGAAGGGACCATGGACCCCTGAAGAAGATCAGATATTGATTTCTTACATCCAAAGTTATGGCCATGGCAATTGGAGGGCACTCCCTAAACAAGCTGGTAATAACTTTTTTAATTACACAAAAACCCCTCCTCCTCCTCCTCCTACTTCTTAATTTGATACAACTTGTAGTAATTGCTAGCTCTGGCTTTGGTTGGCACACACCATGGAAATGGAATTCAACTTCTTGAGAAATTTTATAACACACCCCTCAAAAAAAAGTAAAATAGATGACTCAGTTATGATTTTTATTTCAAAAATAATTTAGACCTCTAAAGGAAGAGCTCTAATAGAGCACTAATTTGTCTTTTTCTTTTCTCTTTTTTATCTTTTACTTCATTGTTTTTATTTTTTATATTTTTTTTATTGATTGACAGCTCATTAGAGCTATCCTTTTGCAGCTCTAATTAGAGCTCCGGAGCATTACCCTTTTTATTTTTACTTATTTTAATATTGTTTTTATAGCTTATTATAGTGTTGATAAGGATTAATTATAATCTATTGTGATTGTTATTTTTAACTATGATTGTAAGGTTTTTGGCTATGCTTTATTTCTTTGGTTATGATTGAGAGTAACTTAATTGTAACACAATTAAGCAATGTTAAAACTATAAAATGTTATAACTTGTATATGATGTATGTTGTAGAATCGCTTTTCAATTTGCTTTTTGAAGCAATCTTTTGCAAATTAATGTTCAGATAAATTTTGAAGGTTCTTAGAAATATTCTTAATCCAAAATCAAAATAAATTCTTTGAAAATCTAATATATATATATATATATTTGTTACAGGTCTACTTAGATGTGGTAAGAGTTGCAGACTTCGATGGACTAATTACTTGAGGCCTGATATTAAGCGAGGAAACTTCACTAGGGAAGAAGAAGAAACCATTATCAAGCTACACCAAATCATTGGAAATCGGTATGTACTCATTGAATCATTAAATGTATATTTGGATCATGAATTTGAATAATAATTTTAGAAAGATAAAAGATAAGGGAAAAGAAATGGAAAAAAAAAAAAAAATTCTAATATTTCATTTACATATCTTCAACTTTTTTTCCATCTTTTAAAATTTTTGTTCAAATCTAAGATGTGTGGGTAAGGCGTACGTCCCCGGTTCGATTCCCTACTAGGTGAGTGTTTGCAACAGTTTGAAAAGGGTACACGGCTTTCCACCAGCTACTTACCCCTTAGGATTAGTGACTTCCTTTACAATCAGAGGATTGAGAATACCTGAGTTATCAAAAAAAAAAAAAAAAAAGGGCCTAACTGAGAATAATCATTATCTAGCCTAACCTATATTTTGAATATTTTTACGTTGTTGTATTTTTATCATTGTTTCTTATAAGGATCCTTATCAAAAAATGTGTACCATGTCCTATTCATGAAGATTTGTGATCTTATTCGATTATGATATTTCTTTGTTTTTTTATTAGCGAAATAAAATTGTTGATGTGAAAAGATGAGTTATAGAATAAACAACCGAGGAGACAACACCTATCTAATTTATATCTGAGAAAATTTCATTTTATTTTCAAAATTACATCTTGATAATTAGTTTAACCTCGGTACTTTATAAAATTTTAATTTTATATCTAAATTATTTAATTTTTTTATCAATTTAACTCCTGCTATCAGTTACCGTCTATAATTAGGCGGCATTTGTTACTATTTTCATTTGAGATTTCATTTTATTCCTTAAATTATTTCCAAAATATCAATTAGACTCCTAAACAATATGAAGTTTGGGGTTTAATTGATATTTTTAAGTTAATTTTTTTAGGGGGTGGGGGAAATAAAATTTTAAAATTTCAGTGTTGAAATTATAAGAAATGTATAAATTAAATTAATCATCATCTAGATATAAATTGGAAGGTAAAATTATATCTACACATACGTAGTTCCTGAATACTTTTGCCCGATTACGAACACTTACTAAAAGATAAGCGAAGAAAACAAACAGTAAAAAAAAAAAGAAAAGAATGAGAGAATATGGGATGTGAAATAAATATTAAATATAACAGTGACTAACTAATAAAAAATCTCACAATCCAGTAATAGAATTGTAAGGTTGGATTAATGGATTCTATTTACAATTTTGAATTGGTTAGTGACACCAACCTGAAATGGTGTGATTCTCACACTCAATATTTATTCTATATTGGTTAACAAAAGGATATTTATGGTGTCATATGGTCTCCCACTTTTTTCAAATCCAATAGAATACGCTGATGAATACAAATTAGTGTGTATTTAAAAAAATATTTACAAATTCTAAGTCAGTGTGTATTTAAAAAAATATATTTACAAATTAGTTTCTATTAATTTTGACGTAAAATATTTTTGTCAAAAATATTTTATTCACTTTTAGTTATTTAGTTAAACAGAAAGTAAGTAAAATATTTTTTAAGAAATATATTTTTACATCATTTTTTCAAAAATGATTTACCTTTAAAAATTTCATAAGTCAAATTTCACAAAGTTGACTCTCACACTACTTTTTCAACACATTTAACATTTTCAAATAAATATCTAAACTCACTAAAAAGTCAAAAATAACATTTTATTAAATTAAAATTTCTTTTTTAGGCAATAAAATATTTTTATTTCAAGTATCAACCAAATGTTAAAAAAAAAAACAAAAGCATTTTTTCAGTCAAAAATCAATTTATAAGAAAATTATTTTTTTAAAAAAATATTTTACATCGAAACAAGCGGAGCCTTAATGATAAATTTTATAAATATTAAAGTATAAAGTGATAAATTTTTTCATTAACTAGTAATTATTTTTAAAAAAAGCACTGAGGAGTAGTTTCCCCATAGAAGTTCATCAAGAATACATCAATGAAAATATGTGTCAACTTAATTCAATTAATAACGTTACTTTAAAAAATTATTTTAAAATTAATAGATAAAATAGTTCATTTATTCTTGTCAAAATAATTGATGGGCTAGCATCCAATTTGAAAAAGCCTATTCCCTTTTGAAGGAAACATTACGCCTTAGATTTCTATCCAGTTTTTTAACAAATGTTATTAATTAGATTAAATTAATATAGAGTAAAAATTAATACAATTATAATTATATAGATCAAATTGACTTGAAAAACAAATTACATAGACTAAGAATATAATTGGGTTAATTATACTAGCACCCCTGCACTTTTATCAAAATTTCACTTTAGTTCCTTCACTTTAATTGTAATAGTTAACTCTCTGCACTTTAAATTAAGTTCTGGTCTTACCCTTTCATTCATTTACCGTTAACAAAATTGACAGAACTTGTTTGAAATAACATGTGTAACGCTTTTGTTGTCTCATTAATAGATTTACACTTTTATTAAAATCTCATTGTAGTCCTTAAACTATCATCAAAATCTCACTTTAGTCCCCACACTTTCAATTTTCATTAGTTTTATTAATGGTAGATGGCTGTAAGGGTGAGGTTGGAACTTGATTTAAAGTGTAGGATAGGAATATAAGAAAAACCAAAAAGACTGACTAAATCGGTTGAACTGAATCAAATCGGCTATATTTAGTTGATTTTTTTAATATGAATCAGTTTGTGTGATTTTTAAAATTAATTTTTTTCTATTTTCGGTTCGGTACCAGTTTAGGCATGCACCTTGTGCATCAAATTAAACTAAAACTGATCGAAAGTTTCCCATATTAAACATCATATATATTAAATATATAATGTAATTGGTTGAGTACGAACTTCAGTTGATTTAATTAATTGTTTTTTTTCTTCTTCTTAAACACTCCATATTTGACAATAGACATTGGTAATGTGATAAGTTTTTAGTTTACTACTTATATGTGCTTGAGTCAGTTTTGCTACTATTATACCTATATAGCTATAGCAGATGTTTATGTCATTTTTATTTTTTTATTTTAACTTTACTTGATAGATTTGTAATTTAGCCTTATGGGATGATGGTTTTAAACTTTGGTGTCAAGACTTTCGATTTTCAGATTATAGTTTGCTTCTAATTTTACTTTAGTAAAAGCTTTAAGACTTTAGTATTTATTAACTCTTTTATTTGTATTTTTGTGAATGGTTGGATTGGATATTGAAGTAATGTACAAATGACTAATTCAATATTTTTATATAAATTATTTACTATACAACAAATTTAACAAGCAACTCAAATTTGAAATGCCCTAAATCGAAAACCGCACTGAGCTAAACCGTTTGAATTGGTGCAGTTTAGTAAAAATTCGATCTATATCGGTTCTCAATATAATAAGAACCGACATTATTAATTCGGTGTAAAATTTCTCCCAAACACCGACCATTCCAAACCGATTATTGACTATTACAACTGAAAGTAAATAGATCGAATTAAGATTTTAGTGAAAATACATGATATTATAGTTTACCCAATATAATTTATCCTCAAATTTTTGCTCTCCTTTTCAACATATACTATAGTCATATGCTTGAATGGGAAATTGACAATTTGAATGTAAAATTTTTCCCAGATGGTCAACCATTGCATCAAGATTGCCTGGTCGAACCGACAACGAGATCAAAAATTTCTGGCACACTCATTTGAAGAAGAGGCTTAAACAAAGTCATCATATGGTAATTCAAAGCTTGACATATGCACCAATTTCTACACAATTTTCCGAGTTTATTTCAAGTTCAGAGTCAGACTCCCCTGTGGAGAAAGTTACAGGACCTGCCAATGAAGAGGAGTACACGATTAATGCCAACGTCAATGATGACATGGCTTTTTGGCACAATCTCTTCATGAAAGCAGGAGAGTAA

>LOC114311452|CSS0006819.1 4-coumarate--CoA ligase-like 9

ATGGGGGAGGACATGCCACCAAAGCCCACGATGATCGACCCACGCAATGGCTTTTGCTCAGAGACCAAGACCTTCCACAGCATGAGGCCAACCGTCTCTCTCCCTCCTCCAACCCAACCCCTCTCTCTCATCCACTACACACTCTCTCTTCTCTACCACTCCTCCACCCCCACCACCACCCCTTTCCTCATCCACTCCTCCACCGGTCTCCACCTCACCTACTCCGACTTCCTCCGCCAAACCAAATCCCTTTCCCTCTCTCTCCTCAATTCTCTCCCCTCTCTCTCTAATCAAAACCACGTCGCCTTCATCATCGCTCCTCCCTCTCTTCAAATCCCCGTTATCTACTTCTCTCTCCTCTCCCTAGGCGTCACTCTCTCTCCCGCCAACCCTCTCGCCTCCCAGTCCGAGATCACTCACATGATTGGTCTTTGCAAACCGGTCATCGCCTTCACTACCTCCTCAATCGCTCACAAACTCCCCTCTTTCCCACTCGGCACAATTCTAATCGATTCGCTCGACTTTCAAACTATGATGATGATGAACCCTACTACTGAAAACAATCACCAACCGAATCCAGAAATCAAAGTGGAGCAGTCAGATTCGGCGGCAATACTGTATTCGTCTGGTACGACGGGCCGAGTCAAGGGCGTTGAGTTGACTCACCGGAACTTCATTGCCTTGATTGCCGGATTCTACTATATCCGGCGAGAAACCGACGACGACAACGCTCCGCATCCGGTGTCTCTGTTCACGCTGCCGTTGTTCCATGTGTTTGGATTCTTCATGCTAATTAGGGCGGTTGCGTTGGGGGAGACTCTGGTTTTGATGGATAGATTCGATTTCGAGGGGATGTTGAGGGCGGTGGAGAAGTACAAGGTCACTTACATGCCGGTGTCACCGCCGCTGGTGGTGGCGCTGGCGAAATCGGAGCTGGTGGCGAAATACGATCTCAGCTCTCTTCAGTTGCTTGGATGCGGTGGTGCGCCGCTCGGGAAGGAGGTTTCCGAGAGGTTCCGGGATAGGTTCCCCAGAGTTGAGATAGTGCAGGTATATTAGGATAATGGCGTGAAGTCCAATTCTCTTTAAAAGCGCGTAGTATAAACTTTATTGTCTCCAACAAAAAGGTCATTATCTATGTTAATAGTTTATAAGCATGTGTCATTATTTATGTTAATAGTTGCAACAATGATTCATTCCATTAAAAACACCACCACTAATAGCACTAATGTATTAAAAAAATAAAAAAAATAAAAAAAAATTACAACTCTAAGACCAAAACATCACATCATTCATGACGGGACCTAATTAGTGAGAACACTAATTAGTGCTCCCAAACATTTTTCTAATTATTGCTGGGTTAGTTTCTTATACGTGACCATGTGAGTGAGGTGAGGGTATATTGTTAGGGACGGCAATTTGAACCCGACCTGGCGGGACCGGACTGCTCTATTCTGATAAGTTGGATTTTTCTCGAAAATTATTTCTTATAAGGCCGGAGCTGATATTGAAAAATAGATTCCGATGATTTTTGGGCTGAGGACGGGAATAGATTCCCTGGTCCCTCCATGTATATATATACACCTATTCAGTCACTCTAAGTCTTATGTGTTCTCAATGTAGGAGAAAATTAGATTGTTGGCATTTTTGGGGATCAGTATTCCAGTTTTTTTTTTTATTATTATTATTTTTTATTTTAAATTTTTAGCAATTGGAAGTCCGTGTCGTGTGGAGAGTACTGTTTGGTTGGGAGATTTAGAAAGGGATTTGAAAAGGGAAAGGGAAAGGGAAAGAGAAAAAGTGTGCAAAATTAGTTAAAATATAAGTATGTTTTATTCACATTTTATCTACCTTTTCTATTTCCCTTTCCAAATTCCTCTTCAAATCCTCTAACCAAACACAAAATTAGGCTTTTTTTTGGTTTCTATTTTGAGTACTTCTTGTCTTTATATATCTTTTTTTCTTCGTCTTTAAAGTTTTCTTTTGTTGCTGATCCCAAAAAAAAAAAAAGGAAGAAGTTATTCTTCTTTGATACTGTGGGCATGCAGCATGGTAGGACGGTAGGATGATTTTCACTTTGCCTATTAGAATTTTTACATAGTAGCTAGCCGCATAATGTTTTAACAACAATTTTACTGTTTGAGGCTTTCACTATTTGTTCACTAGTGTTAGTCTTTGTATTACACTGTACGTGATACTGAAAATTTATACAATTAATCAAAACTTGAACAATTGAATTGTTGAAATTCATGGCATAAGAACCAATTGTTATATTTTTGTGAACTTACATGATTGTAATACGGCTGGGACGTTCCTTTAACTGCATGTGATTCTGCTTTTGGCTGCTAATTTACTTTGCGATTCTGCCTTTATTAGGGATATGGTCTAACTGAGACTGGTGGAGGGGCAACAAGGATGTTAGATCTGGATGAATCTAGACGCTATGGATCTGTAGGTCGTCTAGCCGAGAACATTGAAGCCAAGATAGTTGATCCTGCAACTGGAGAGGCCTTACCTCCTGGACAACAAGGGGAGCTATGGTTACGAGGACCTACAATAATGAAAGGTGATCCCAATAGGACCATCATTTATTTGTTTTTGTAGGCAAACTTGTAATATTTTATTCCTTCAAGATAAACTACTGCAAAGCATTCTTATGGTTTTTGCTTGGTGCTAACAATTCCTTGGGGCCATCAGACTGGGGGAAAACCCTTAAATTAATTGTGGTGCACTTGCGGAAAATTCCTTGCAAAGGGTCTATGCACTTCTGGAGATTAGTCGGGTTTTGATCCAGACACCCCAGTGTCAATAAAAAAAAAAATAAAAATAAATATTTACTTAATTTTGAGGCTTGAGCAATGGAAATTGCATGTTGTACTCTAGCTTCTTTATAGTTGATTATATTTACTGCATCTGATCCGGTTTCATGACAGAGTAATTGAATCAACTAGAAGAATAAGTTAAAGCTCATTCTTATGCAATATTAACTGCCTTTTAGAGTTTTTCTGAATTGAAGAGGTTTTTAAAAAGAAAGAACCTCAAAGGAAAGAATTAAGATCTTGATCAGAATTTTATGGAATGACTCATTTACTTGTTCACTACTACCGGGTTATATCAGTTTACAATTGTTAATTTTTATTTTTTAAAACTTTGGCCTTAAAATACCGTTATGATGATTCTTGTTTGATAAGGAGAATGATGGGAAATTATAGGTTACGTTGGAGATGATGAAGCAACTGCGGCAACATTGGATTCAGAAGGCTGGTTAAAGACTGGTGATCTCTGTTATTTTGACTCTGAAGGATGCCTCTACATTGTTGATAGATTAAAGGAATTGATAAAATATAAGGCCTATCAGGTGCTAAAATACTCTTTTACACAGTTTTCAACCTCTTAACACGTGGTTGGAACTGCGTTTTACTGGTATTGAGTTGCCATCTGCTAAAATCTTGAATAAATTGCAGGTTCCTCCAGCTGAGTTGGAACATTTGCTTCAATCAAATCCTGAAATTGCTGATGCTGCTGTAATTCCGTATGTATCTCATATTGTGATTTTTATCCATTTTCAGAAAAATCATGTGTGCTTACGCACGTTTGAGTTTGTGCACCTACAGAGCTTTTAACATTGTAGTGATTATGACAGCTAAATGCATGCACGGTTAGCTCAAAAAATTAGCATGTACTGGCTTCTCTTTTTGATTGTCTTGTTGATTCCCTAGAACACAGTTGGTCATCATATTCTGGAATCCGCACGTGTTTTTTGTTTTTTGATTCAACATCCTCTAAAATTAAAGTTCGTACTAAAATTGTTAGTTTCAAGTCTGCAGTTCCTTGCATTCAGATGAAACATGATTCCAAGTATTCATGAGCCAAATTTTGTGAACTCTTTTTTGTTTTTCTTCTGCTTTTTGTCAAACCCACTTAACCAGTAAGTTAGTAATCTGGTTCCTACACTTCGTGTCAACATCTTTACAGGATGGGCAAGCTTTTGAGAGATTTCATCCATCTCTAATATCCTTCTCTTATGAAGTTGAAACTCATCTTTCACCATGTATATTCTACAATATACCTCATGATTGGCTAGCATTTTTGGACTCAGTAATGTGGATTCTTATTCAATCTAGGTTTTCTATTGTATTTATGTCTGCTTTCCTTAATCCATGACTCGCCAACACAAATCCATCTTTCAATCTAGGTTTTTCATTGTTTCCTTTGTTTGGTAACAAATTATTCTCGTACTTGTATGACGTTTTCAAGGTCCCGCTTTAAAAATCAATTGCAGAATATTCAACTTTTAGTCGATAACCTTTTATAGGTTCACCTTGCTTTCAATGTGATGATTAATGGAAGTCATATAAAGGAAGATTTTTCTCAAAACAATTGGGTTGGAAAGTTCTTCCCTCCTTTTGAACACTGAATGTACATGTTTAACGTTTCACCATTAGTTTTTTTGTCTGAATCCTATATAGTTTGTAATCTTTCTTTCTGCTTCAACATACTTAACCTGTGGGTTTGCTTTCTTGATATGCTTACTTCTTAAGCCCTAGGAATATTTTTCATTACCTACAAAAGAAAAAAGAAAAAAGAAAAGTGTGGAATTAATTAATTAGTAAATCCCTATTTAACATTTTTGGTTTGAGGTCAACTTAAGGTGCTAATATAATATTTTATGGTCCGTGATGTAAATTAGCTTGCTTCAACAACCAAACTAGCAAACAATATCAAATAAATTGTGTCATCTGAAAGCAGATATCCTGATGAAGAAGCAGGGCAGATTCCCATGGCCTTTGTAGTGAGGAAATCTGGGAGCAATGTCACTGAAGCCCAAATTATGGATTTCATTGCAAGACAGGCAAGTTAACTTCCAAATATTATTTTAACATTTGATTGATTACATTTAGGCTCCTTTTGTTTTGACATAAAATTTTTTTGCCAAATATTTTTTACTCATTTTCTGTTGTTTGGTTGAACAAAAAGCAAGTAAAACATTTTTTAAGGAAAATATTTTTGCATCATTTTTCCAAAAATGACTTACCTTTAAAAATTCCGTAAGTCAATTTTTACAAAGTTGATTCTCATTCACTACCTTTTCAAATAAATATGTTAGCTCGCTAAAAAATCATAAATGACACTTTTATTGAATCAAAATTTCTCTTTTAGGCAATAATTTCAAGTGTCAACCAAACACTCCAAAAAAAAAAAAAAACAAAAACATTGTTTTTTTGTAAAATATTTTATGTCGAAACAAACGGAGCCTTAGTATTACATATGTGTCATTTGATGCTAGCACTTCTTGCATTCTGCTTAAACAATCTGTGTACATACTAGCTTTCAAGCTTTACAAAAGGAGGAAACAAAACATAACAATTTAGTAGTCATGAAAACCCATATATTAAGAAAATATAGATGCGGGGAATTGATGATTGTCTTTGACATTTTTGGGATACCTTTGTCATTTTCTCGACCCATCACCCCTGTGGATGCTCTGGGAATGACAAACATACTTGGGAAAGTTCAAATCACCAAGGAATAAGAATTTAAAATGTTCATCCCTACCTAATGAGATTGGTTTTTCTAATTTTATTTTTATTTTTAAGTAAGCGGTTTATAAAGGAAAGAAAATATACAAGGGGCATACCCTAGGTGCATAAATATAATATAATAATAATTAAAAAAAAAGTGAAATCCATAAGATAAGATCTACAAACTAACCTGGTTTGTAACCAAGGAGGATACATCCCTCTAAACAATACCCAATCCCAAACCCCAAAACAAAAGTCGCCCAGCTGCTACTACCATAGAATGAAAATCAGGCTATGGCAAAAGCAGCTGAGGGATCATTCCTTCAAAACCTCCCTATATAAGAACAAAAACCTCTAAACCATAAACCTACTTAACAGACCCAATTATTAAAAAAAAAAAAAAAAAAAAGCACAGCCATCTTCAACCAAAATGCATGGTATAGAGCAGCCGTAGTGTCAAAAGAAAGCTATGTGTCTTTGATCTGGCATAGTCTTCAAAAATCTTCTAAACCCAAACCATCTTCCTGAGCCTCCTATGCGGTTGAAGCACCTATTCAGCTAGTATGAATGGTTTGAAAGCAGCCATATCAACTTGAGGAACTATGGGATTTCGAACATGGAAGTGCACACAAACCAAAATTTTAGCAATACCAACACAGCCCTATTGAGGACTGCAGCAGCCCACTTTTAAGGGACCCATGGGCATAGCAACACGGATAGAACTCGTAAATATCATCAACACAAGGTTACACTATTGCAACAGCCAACGCAAAGCATCAAAATTAGCTTCAGCAGGAAACACAACCATATTCTCACATGTTACCTCACCCTATCTTACTCACTCACTATACACTACTTTTTTCTCCTGTCTAACTCTGTACGTCCTCATTTCCCCCCCTTATTCTAACTCTACCAGCCCACTCTTTCTTTCTCATTTTTTTTTTTAAAACTCTCCATATGGTAAAATTCCAAACCAACCCATGTTTACGTTTCACACTACATAACATCTAAACAGCTAACAGAACATCCCACAGAAGGAACAAACAGCCACTGCCCTAGCCTGCTATTTCAGCAGCAGCAACAGCAGAGTAGCAAGCAGCAATCAGATTCCTACAGAGGCAAACAGACCCATATTCACCAGCATCAGTCACTGCTAGGATCAGTTTCTGTGTCACTCACTCTTCACCACTCTATATAGAAGTGCAACAATACTAAAAAAACAGTAAATCTACAAACCATCAGACAAAGCAGAACAGGATACCTCAGCTAACATCAAATAAACAACTAACCAAGTACCCAACTACCTTTTCGCCCCTATATATACTGTTAAGTGTTAATTATTTTTTGTATTGTTGTATGACAGTGCTTGTTACCATCTAAATGTCCGCCCCTACTTTTGTTTTAGTAGTTTTTATGGTAGCACTGGTTCGTTTCAACTTGCAGGTTGCTCCTTACAAGAAGATTCGGCGTGTTGGATTTGTCAATGCTATTCCAAAATCTCCGGCAGGGAAGATCTTAAGAAGAGAACTGATCAATCATGCTCTCTCTGCTGCTTCATCTAAATTGTGA

>LOC114314253|CSS0033075.1 flavonol synthase/flavanone 3-hydroxylase-like

ATGGAGGTAGAGAGAGTGCAAGCCCTGTCCCATGTAACTCTCCATGAGCTCCCTGCAAAATTTATCCGACCGGCCCACGAGCAACCGGAGAACAGCAAGGCTATCGAAGGTGTCACCGTCCCCGTGATCTCCCTCTCTCAACCACACGATGTGGTGGTCGATGCATTATCAAAGGCTTGTAGTGAATGGGGATTTTTCCTCATCACAGATCACGGTGTCGAGCCCTCGTTGATCGGACGGCTAAAAGAGGTTGGGGAGGAGTTCTTTAAGCTCCCACAGAAGGAGAAAGAGAGCTATGCAAATGATCCTTCAAGTGGGAGTTTTGAAGGGTATGGAACAAAGATGACTAAAAATTTTGATGAGAAAGTTGAGTGGATTGATTATTATTTTCACGTCATGCACCCTCCTAAGAAGCTCAATCTTGACATGTGGCCTAAGAACCCTTCTTCATACAGGTATGTATGATTCACATAATCTTATATAAATTCTATCATGATTTTTCAAGTTTTAGTAATATTGCATATTCAGGGGAAAAAAATTTGAAGAACAATTTTTATTTATTTATTTATTTTTAAAATTGAGTCAATATAGTTAAAGTTTCATGAGGTAACAGAGAAGCTGTTACAAAATTATTAAAAGGTCATTAAAGTTAGCTTAGCCAAAATTTGAAAAGATTTCTTGGAAATATATGGAAAGAAAAATAATATGCTATTTTTCTACGAGAGGGACTACCTTTGAATGGAAGAGCCACATTGACTTCAAGAAATGCCTTAGAGATCAGTGGAGGTGACCCACCACCCCACTATTTTTTGATTAGTGAAATTTTTTTTTTTTTTTTTTCTTTTTTTCTTTTTCTAACCTTGCCTCCCAGCCCCCACGTGGGGTTCTGCCCCGCTGGGGTCTCCAGCAAGGTCCTATTCCTAAACCCACTAATAATTTCTCTCCTCTCACACATAAATAAATATCCACACGTCATATCAAAAGCGACTCAGGACAGGTCCATCTTACCTGTCCAAAAATAATTCTTTAATTATTTTTGCGATGGCTCATTTATGAGATCTCGGCAATTGAGCCCACCCCTGATTGATGAGTGATCAGCCCATTCGTACTAAGCTGTTTTTTGTGCTTGACAAAGCGGCCTACATTTTATCATTATCTACTGTCCTGCCACATTCAATTAAAATAAAAAAGACTGGTAGTTGTGTTTGAAGTGACATTAATAATTCCTATTTAAATTAACTTAATAATTTTTTAATTATTTAGTTAAAATATAAATTAAAAAAAATTAGTATGTTTAAAATAATTTTTTAACTTTTTACGAAAAGATGTGCAAAAAGTAAAGAGAAAGGAAAATCTTTGACTTTTTGATATAAATTTTTGATTTTTTGATTTTTTAATAACTTTATGACTTTTAAGCTAAAAGAGACCGTTCCCAAACGAGGGCTTAATAACGTTAATTTCACATTTTTATTATTATTTATATTTACAGAATTTTTAAAATTATAAATAAAATTTTATTTGATTTATGTTTTAAGTTTTAAAAGGTTGTAAACAATTTCTAAAATTTATTAAATTTTATAAATACAAAAAAATTGATTTTAATTTTAATTTTTTTTTTTTCATTTCTTCTTTCTTGTAATCCTTTCTTTTACTCTTTTGAATCACATACCATTGTTCATCATTACTAGGAGTATAAAAATGGCCTGCGGCTTGAAATACACCCTCGATCTAGGATAAGCCTCCTGTTTAGGCCCGTTTGAGCGGGGCCCCGAGCCCAGCCTTAGACCCTAGACCTGTGCCATCATTTTGAAGTCCGACCCATCGGCCCCCTTAAGGCCCACTCAAGGCCCGGTCCGAGGTCCGGCCTACCGAGGCCCAGTGTTTTTCTAGTGGAAAATAATAAAATTTTGGTAGAAAAAAATAAAACAAAAAATAAAAAGATTTGTTCAAGCCTGCCCAAGCCCGAGCTCAAGCCTAGAACCACCGGCCCGCCCTGAGACCCGGATACCCCCCGGGCCTAGGCCTAATTTTCTGGTCCGTGGGACGGCCCGACTCGGCTTGAATTTTGATAAGAACCCACAAGACCTGACATAGGTCAAGCCAGGCCAGGCCAAATTTACACCTCTAATCATTATTGGCTATCATTGCTAATTACATCTTGCACAAACCATCTTTAACCAGTATCTCCTCAATTGCCAACCACCTTGTTTGGAATGTGGGTTGACTTTTTAATTTTTTGACTTTTTGGCTATTTTAGTTAGGGGCTAAAAGCTAAAATAGCTTCTTTTAGGATAGATTTTTTGATTTTTTTGACTTTTGTAAGAGAAAAAATAAAATGATTATCGGTTGGATATGACATGGGGGTGTTTAGGAAGAAAAAAAATAGCCTAGTTTAGGCTAAAAATCACAAAAAGATAATATAGGCTAAAATATTATAAGACAAAATATCACATCAAACCCATAATTATTTCACTTTTTCTCTCACAAACATCACATCTAACTACAAAAAAATCCAATCATTTTTCCAAACACAAAAAATTTAGCCTATTAAAATAGCCAAAAACTAAAATAGCCAACTAAAATAGCCACTTCCCAAACAAGGCCTTTGTCTTATTTGGCTAAAATAACATATTTTGTCCTATATGACTATTTTAGCCCCAACCTTTATTCTAAATAGGGCCTATATGTCATAGCCACCAACCACCTCTCACCAATAGTAACCACGTTTTGTTAAAAAACACCGACAACTACCATCCTAGGAGATCGACTAGCCCTTATCTTTGTCCATAGACAATCACTTTCGACCACTACTCCCCGTGGCCATTAACTACCTTTGGTTATTGCCAACCACCTGACACCAATAATAACCACTTTTGGCCACTACCTTTCGTGACTGACAATCACATTCGATCACTACTCCAATAATTGACAACCACCTATCGCAATTACTCATTTTCACTTGTTTTTAAAATAATTTTTTTAAAAAAGTTGCGTACCAAAAACTCTTTTTGTTTTCATTTTTATCCAAAATAAACATAAACATATTTAATATTTTCTGTAAACAAAAGATAGTAAACTGTAAACATTGTCAAAATACCTAAAATGTGCCAATCAATTAATTCTGGAGACACCATTTAAATATTTCTAATACTCTATTTACGATGACTGTCTATTTTATAAAACGCAATTGTCGTTTATTTTTTGGAAATAAATATGATTAAAAAAATTATAATTATTAGATCAATTTATTTTTTTTCATAAATGATATACACAGAAAAAAATTTAAATTTTTTTTTCAAATCAACAGAATCCAAGGTAAACTTTGCCACTCGAACAAACCGAAAAGAAGCCCAACCCTTTTAGTTTCTATTTGCAAGAATAATAGACACCATCATTATGAGTAGAAAGTAAAAAATAAACAATTGAGCATGTTTGGTAATTTTTGTTCAGTTGTAATCATAATAAATAAATAAATATGTTAGTTTCACTTTTTAAAATTTGTACAATCTAAACCTATCAATTATAAGTAAACTAATTGTAATTTCTCCATTTTCACTGTTTAAGTTAGAATCCAAATTTAACAAATCAACATTTGACATTATTATACTTTAATGTAACCACCACTTCGAAATCATAATTAGCACGACTTTAATTCATAGTCACGACCACTTTAGAAGAAAAAGCAAAACAACTTTAATTCATAGTCACGACCACTTTAATTCATAGTCGCGACTTTAATTCATAGTTAGAATCCAAATTTTCTTTTCTTTTTTTTTTTTTTTTTTTTGATGGGAAGAGATAGAGAGAGATTGAAAAGTAACTTTCAGATCTTATTTTTCCAAATAATTTTTCAACATAACTTTTGTCACTTAAATTCAGATGATGAAATTAACATGTAAATATGCAGGGGAGTGACAGAGGAATACAATGTGGAAATAATGAGAACAACCAACAAGTTATTTGAACTTCTCTCAGAGGGACTAGGTTTGGATGGGAAGGTTTTGAATTCTTCTTTGGGTGGTGATGAAATTGAATTTGAAATGAAAATCAACATGTACCCACCATGCCCACAACCTCAGCTCGCCCTCGGAGTTGAACCTCACACTGACATGTCTGCTCTCACTTTACTTGTCCCCAATGACGTTCCCGGTCTTCAAGTTTGGAAAGACGGTAATTGGGTAGCTGTCAATTACTTGCCAAATGCACTCTTCGTCCATGTTGGTGATCAACTTGAGGTAAAAATACTCTTCTCTTGCTCACCTAGAAATATCAAACAAATGAACAAAAAGTCTAGTAAAAGGAAAAAAAAAAAAAAAAAACACCATACTTGGAGAAAATTGCATTGAATATGACAGTAATGATTCAAATTTAGCATATATATGTGAAAACATAGAGTTCTTCAAGCAATGGGTCTATTTGACAGTTTTTTGTAAGTTGAAAGCTAATAATTAGAAACAGAAAGCCAAAAGCACTCATTCGTGTACTTCTCAACAATCAGTTATTTATTTTATTTTACATTTTATAAATATTTTTGCCTCATAAGTTAAAAGCAGAAACTGCATCAAACATTGGCACAAATTTTCAAAAGAAAAATGCTGTAAACATCTGCTGCACTAGACAGGGCCAATATCTAGCCTGACTGTAGTGAAAAAAATCAAGATTCAGAAAGCGTTTCAGTGCTGCAACTTACAAAACATTTTGTTTCAGGTACTAAGCAATGGTAAGTACAAGAGTGTTCTTCACAGGAGTTTGGTGAACAAAGAAAGGACAAGAATGTCTTGGGCTGTGTTTGTCGTGCCTCCTCATGAAGCAGTGATTGGACCTCTTCCAGAGCTCATTGATGAGAAAAACCCAGCAAAATATTCAACCAAAACATATGCTGAGTACCGTTATCGCAAATTCAATAAGATTCCACAATAA

>LOC114314260|CSS0046767.1 glutamate dehydrogenase 2-like

ATGAATGCTCTTGCAGCCACCAAACGCAACTTCCGCCTCGCCTCTCACATTCTCGGCTTGGATTCCAAGCTCGAGAAGAGCCTCCTCATCCCCTTCAGAGAAATCAAAGTTAAAAAAAAAAAACCCTCTTTCTTCAATCCTCTGTTTTCATCTAAATTTTCAAACTTTTTTCACTTGTGTTTTGTCTCTTGTTTTTCAGGTCGAGTGTACGATCCCAAAAGACGATGGAACTCTAGTGTCCTATGTCGGATTCAGAATTCAACATGACAATGCTCGAGGTCCAATGAAAGGCGGCATTCGTTACCACCCTGAGGTAATTATTGTTGGTGCTAGTCTGTCCTAATTTCATTGAATTGGGTTTTGGGTTTTGGTGTTTTTGCACTGTTATAGCTGTGTTTTTATAGTGTGGACTCTTTCTATTTGATGTTTTGCATGTTTGAGAATATTTGTTGATGGAATTTATTTTGATATTTACTTGGGTTTTTTAGTTAGTGTTTTGAATATGGATCAGACTGGTGAATCTTGATTTGGGCATAATTCTGATTTTACTTTGGAGTTGGTAATCAAATTTTGTTTTGGCATGCAATTGCCTCTGTATATATTTTTGCATTTTTGTTTATAGATCATCAACCTTGATGATGATGCTAAGGACAACTGGTTGATCATTAAATTTATGTTCATGTGGATCAGAGTTGTGGTTATGATTTTAAGTACAGTTTTTTTTTTTTTTTTCCCTTTTCTTTTTCCCTTTTTCTTCTGGGATTTTTTATTTTATTTTATTTTATTATAATGATTCCCAAAGATTAATCTTGATATTTACTATAATCGTGACAATCCCTTGTTTATTCCAAATCAATGTTGTCAATATTCCTAAAATGTTTGCCAAACACGGGTTTAATGACAGATCTAGTAATTTTGAGAGCAATTTTTTTTTTCTTGGGACTACTTTGCAAAAAAGGGATGAAGCCAGGACTTGAACAGTTTCTAAATCTTTCACTCATTTAATGGAAGCTCAATTTTCGTCAAATTTTACTAATTCATAGTGTGATTATGTGTTAACCAGCCATATATGTATCAACTGTACATAACTGGCAGTTTAAATGTATAAATGCTATTTGTATGTCATTATTTGGGAAAGTCATCAAGCATATCTATTTATAATGGCGTCTTTTGTTGCGACATGTGTTAGGTCCCTTTACTGTCAAATCCGGATACAAAACTTTGTGTTGAACAAGTTGTAACTTTCTCTATGTTTTTGTCTTTCCCGCTCGAGCGGGACCATTGGCGCTCAAGCGGAAATTAGGGTCTCTCAGTATTTCCTAGATTTTATTCAAGAGTCCTAAGGTTTTGTTTGAAGCTTTTCTTCTGAGGTTTTTGTGAGTTTTCCTTGATGTTTTTGAGAGAGTTCTAAACTTATGATTTGAGATTCATACATCAAGGAGTTTGTTACTTTATATTCAAGAGAGTTCTCTTCGGTGGATTCCGAAGATTCAAGCGTAGAGTTTTCAAAAGTCGTTTGATTCTGTACGCAGTCGTTAATTCGACAAGTGAAGTTGTGGATTCAACTTCAGGATTGATCAGTTCCGAGACGAAGGTGTGGTGAAAGAGTTACAGAAGATTCGAAAACAAATTGACTGAGTTGGGTAATTTGTGTATTATTTTTGTAATCTTTCTTTCTTCAGTAGTGGATTGATTAGTGGCGTTTCGCCCGTGGTTTTTGATTACAATTGAAAGTTCTTCAATTGTAGTTTTTTTCAAGTAAAAATTGTTTGTGTCGATTTATTGTTTATTCCGAAAAACAAACAATCTAAATCACTTTCGTAAGTGCTTGTTGATTTAGATTGTGTGTTTTGGATAATTGGGATTTGGAATATTTTCATCTTGATCTAATCACAAATCACTGAGGATTTTCAACATGGTTTCGTTGTACATCCCTTTTTTTCTTTAAGTGGATGGACTTTTTATTACAAAATAGAATTCTGAAACTTATTGTCGATTACTCTTTATGATCAAAACATATGATCATAACGTGTTTTTGTCGTCTTGCATCCTTGTATCCTTTAATGGATAATAAGATACAATATTGAATCTCTGAGGGAATACTTGGCATGCTTATAACATATCATTTACAATGTCTTATTTTATCGTTTTCTGATTTTGTTATTTGTTTCAATTTATGGTTTTGCCATCTTTCAATTAATTTGTAATAGCATGAAATGTAATTTGGAAGAATCAATAAAAAACGAAATGAAAAAAAATTCAGATTTATTTTAATTTACATACATCAATTATGGGAAGGAAACATAGCACTAATGGGTCAATGGATCTTGTTAATCAATTCATTGGACCTTTTGGTCTAACCCATTGAGCTTCGCAAATTCTTATAATCAATCTATATTGATAATTATTTAAATTGTTCTTTAAGACAGAAAAAGTTCTATGATGTTGCGGTCCTGATCTCGTGTTTACATGGTAGAATTCCCAACAAAATTCGTCTTCCTGTTCAAAAATCAATATTTGTCTTGCTTATATATAATATCCTACAATAGTTATTACATTGGAATGAAAGTATATTACCTCCTTTTACATGCTTATCCACTAGAAGTTAATAATCATGCATAAAGGTGACGATGAAAGCTAAAATATTTAAGGTTTTCTTTGTTTCATACTTGCTTAAAATTTTTCAAAACATATGTTGCAAGGTTGACCCAGATGAAGTGAATGCTCTGGCTCAACTAATGACATGGAAAACAGCAGTAGCTGATATTCCATATGGTGGAGCAAAAGGCGGTATTGGTTGCAACCCAATGGACTTGACAAAGAGTGAGCTGGAACGTCTTACTCGAGTGTTCACCCAAAAGATCCATGATCTTATAGGAATACACACTGATGTTCCTGCTCCTGACATGGGAACTAATGCACAAGTATCACCCCCTTCTTGTCACCCTTAACACTTCAAAAATCACATATATATATATGTATATTTCAAATGCAATTGTTGTTACTTTATGCTTTATTTGGACAGACAATGGCTTGGATTCTAGATGAGTACTCAAAATTTCATGGGCATTCACCTGCAGTTGTTACAGGAAAGCCTATAGTAAGTAGGAGAATCAATGTTGAAAAGGCAACAAAATAGATATAGAAGTTGTGATCGATATGATGTAAGTGAAAAACGTTTACTAGTGCTTGTTTGGCATTTTAGGATCTCGGAGGATCGCTTGGCAGGGAGGCTGCAACTGGACTCGGAGTGGTTTTTGCAACTGAGGCTCTACTCGCTGATTATGGGGTGTCAATTTCAAAAATGAAGTTTGCTATCCAGGTACTTTTAACATCATGAAGTTTCATTCATCTATGTGCTTAATATCCTAATTATTAATTTTGTTACATTGATTAATTTCTTAGGGATTTGGAAATGTGGGCTCATGGGCAGCCAAGTTTATTCATGATCGAGGTGGAAAGATTGTGGCAGTAAGTGACATTACTGGGGCAGTTGAGAACCCCAACGGGATTGATATTCCGGCTCTCCTGAAACACAAAGATAGCACAAACAGTTTGAAGGGGTTCAATGGTGGAGATGCTATGGATCCAAACAACTTGCTAATTCATGAATGTGATGTTCTCATTCCATGTGCTCTAGGTGGTGTTCTCAACAAGTATGTGCTCTGCCAATCTATGAAACCCGTATTCTCTGCTTATTCCTTCCTTGTTGCATAGGTGCATTAGTGCTTTTTTAGCTTTACATAATCCATCAAAAATAAATTATCACAAGTCAAACATATCCTATGGCCTGTCCAACGAGAAAAATGATTTAGTATATCCTTGTCAGCTATACATGTTTTGAAGTTCTCATTTACTAGAATAGAAGAATGTCTCGTAGATATTAGTTTTGACTTGTTTGTTAAGCACTAATGACACAAGTTTTGTTGTTCAAAGGGACAATGCTGCTGATGTGAAAGCAAAATTTGTTATAGAAGCTGCAAATCATCCTACTGACCCTGAAGCAGATGAGGTAATGAGGCATCCATGGTTCTTTCTAGTACATACTTCTTGAAGAGGCACAACATGTCAAGCAAATAAATACTATCTCATAATACATCTCCTATGCAGATTTTATCCAAGAAAGGAGTTGTTGTACTTCCCGACATCTATGCAAATGCTGGAGGTGTGACTGTGAGTTACTTCGAGTGGGTTCAGGTAGCACAAACAACGAGAAAATCTACTCGTGTGTTTTAAAATATGCAAATATTAAGTCCATGACATAATTTGATATGTGTGTGTGTGTGTGTGTACTGCAGAACATTCAAGGGTTCATGTGGGATGAAGAGAAAGTGAACCATGAGCTTGAGAAGTACATGAGGAGAGCTTTTCATGAAATCAAGGCGATGTGCAGTACCCATGGATGCAACCTTCGTATGGGTGCGTTCACTTTGGGAGTGAGCCGAGTTGCACGTGCCACCCTCTTGAGGGGTTGGGAAGCATGA

>LOC114314776|CSS0014373.1 WD40 repeat-containing protein HOS15-like

ATGACCAACATCACTTCTGTCGAATTGAATTATCTCGTCTTTCGTTACCTACAAGAATCTGGTCTCTCTCTCTCTCTCTCTCTCTCTGTTGTATATTTAGGGTTTATGTGGAATTTTAATATTCGTCGTATTTGCGGCAATTAGGGTATCTGAGTTTGCGTTTTGTGCGTTTTTGGTTTAGGGTTTGGTTGAGGATATGGAAATTTAACATATTGGGAATAAATTCATGAAGTAAGTGCGTGTAGATCTGTGGAGATTTATGGCCTTTAGTGTTGATGACAACTTAGTTTACCGTAGATTGTGGAAGAAACTGGTAGGGTTTGCTTCTTCTAACTGCGAATATTCAAGATTCCAATTTTATTTTATTTTTTTTTATTAATAAGAATGCTGAGGGCGTTTCTGCAGCAGAGGTTTGGAAGGATTATTAGTAATTTTGTATAGCAAATTCACCTCGAGGGAAGGTGTTTCCATTTGGGTGATGTTGTGGAATTTGTTGAGGATATCTCCAAATGGACTTTTTTTTTGGTTTAAAAAAAAAGAAAAGAAATTTCATATGAAGGCACTGCGATTCTTGTGAGTTTTGGTGGGGGGTTGTTCTTTTAGTAACTTCCGTAGTCTTTTGGCTAGAAGGTTAGCTTCCTTAGAAACAGCAATGAAAGAGAGATCCGGCTGTTTAGCTCTATCCTTAAGACTTTTGACAATGTGTACTTGTCCATTTGACTCTTGTATATTTATTATGTTTACTTTAATTCACGGTAGGCTATGAAGCACCGGTACTCCTAAAATGTTGCCGTACAAGTACCGGGTACGTACTTGGTACGGGTACGTGTACCGGTATGGCTGCACGCGTATCGGGTACGGCCAAAAGGGGTCTGGTACGTCTGGTACATTTGGGTACGGCATGGGTGCGGAGGGGGTACGCCATGGGTGCGGAGGGGGCAAAAAATACTCAAAAACTACATGTATACATGTATACATACCATAAACATACCATAAACACATCTATAGAGGGAATCGAGAGAGAGACAGAGGAGATGGAAGAGATCGAACCTGATAGTGGAGAGAGGCGTCGCCTTTGTGTGCTAGGGCAATGAAGGATTTTAGGGTTTTCTCTTCGATCGATCGATCGATCAATCGTCTCTCTCTTTGACCGATCTTCTGCTCACTGTTGATCAATCGTCTTCGTCTCTCTCCTTTCTCTCTCTCTCCGTCTCTCGATCGCCTCTGTTCTCTTGTTCTCTCTCTTCGTCTCTCGATCGCCTCTGCTCCTCTTCGCCTCTGCTCTCTCGCCTCCTCTCAGATATAGTATAAGACATATGATACATATAATATAATATAAATAATATAAGATATATAATATAATATATATAATATAAGATATAATATATAATATAAGATATATAATATAAGATATATAATATAATTAGTGATACATATAATATATAATAATATAATAAATTTTTATATATAATGTAATTGATCATGTATAATATATAATATAATTGATCATATATAATATATATAATAAATTTTATAAATTATAATTTTTATCATATATAATAAAAATATATATATTTATTGGCATACCGCAGCCGTACCCGTATCATACTTTTTTGAATTTTGACGTATCGACGTACCCGCACTCGTACCCGTACCGTACTCGTACCCGTACCTGTGCTTTATAGACGGTAGGTTTACTATTGAAATGAGCCCTGTTTGCTTCTAAAAGATTTAGTTAGACAAACCAACCTTTTACTTAATAGTTAATAGTTTGAGGAACCAAGGAGGCAAGAGAGGAAATTTTTGGATGGCTGTACGTTAAATTTTCCTTCTAAATTCTAGGGGAAGAGTAGCGTTTGATGTTTGATGTGTTTTTTATTATACACATTTGCAGGTTTTACACATTCAGCTTTTGCTTTGGGATATGAGGCAGGTATTAACAAATGCCCTATTGATGGAAATTTAGTTCCACCTGGAGCTCTTATCACATTTGTGCAGAAAGGACTTCAGTATCTAGAGATGGAAGCAAACTTGAGTAATGTTGCCATACTTCATTCTGTTGCATAACTATAATCATATCTACTTCCAATCAGTTTATGTTATTCTATTTTATTTTTAAATACTTTGTGTACTTATGTTTGTCAATTCTTATTTCAAGCTTGCCAGAGCGAGACAGATATGGATGAAGATTTTTCATTTTTACAACCTTTGGATCTCATTACAAAGGATGTCTATGCTCTGCAACAAATGATAAAGGACAAAAAGAAAAGTCAACAGAAGGATATGGATAAAGAATTTGACAGGGAACATGATGAAGGTGAACGTAAGCATGTAAGAGAGAGGGAAAAGCATGATAGGGAGAAAGAACGAGAAAAGGACAAAGAGAGAACAGAGAAGGATAAGGAGAAAAAGAAGCAGCATGAAGATCACACTTTGAGAGAAATGGTTACAGATCAAGAAGATAAGGTTGATGTAATAAAACAAGAAGAGAATGGAATTTCTGGAGGTAAATCTATTTAGTTATTTTCACCTTTGCTTTTCAACTCTTTTTTTTTTTAGTATATAGTTGACATTTTGATGAATGCTTTGCATTTAAAGTAAGAAGGGTACTAGAGTACACATCTTTCTAAAGGCTGTGGGCCACTACCAACCAATATTGTCCTGGGACACTTCAGTTTGGTAACAATTGTTTTTCATATCATGGTTTTGAAAACCAAACTGGACTGACTAGTCCAATTGGTTGAACTGGCAATCGGGCTTGGTTCTAGTCCTGTCAACCCATCTAAACCGTTTTAGATTAATAACCACCTTATTTTGGGTAAACCATTTGAACTGGTGGTTGGACCGGTAGCCTGTCCAAACTGGATCCAATTCAACTGGACCAGATATCAAGGCGCAGCCTGCTTAAACTATGTTGGGGAAGGCGTTTGAACCCATAACCAATCATAGGATGACAACATCTGCTACAACTGATCAGCGCGTTTGCAAACTTTTAAGTCGTATATTTTATTTATTCAATGCTTGTTGAAACTAGTTTCTGCAAGAAACCAATTAAAAATAATTTTAAGTAAATATATTTGTTTAAATATAAACGAGACATCAAATTAATTTTGAGGAAATTTTAGGTTTTTGTAAGTAATTGTTTTTGTAATTTTAATACCTATATTTATACATATATATATAAATTTTAACATATATATGTCATAATTATGATGTCATGATTCAATTTTGGTTGACCCGACGGTTGAACTAGTGATCCTTAACCTAATTCCTTTTATTGTTTGGTCAATGGTCCAGGTTTCAAAACGTTGTTCCAGTTTGTTAATGTGTTGCTGTTTGTGCTTAACCACTATGTTTTAGGTTCAATTATTCACATTGCTAATGGAACGAG

AlaAT

AlaDC

ANR1

ANS3

AQP

GOGAT_a

GS2

NPF2.11

TSI

WDR

WRKY57-like

βG

FGS

LDOX

>TEA032217.1 locus=Scaffold110:833307:837259:- glutamine synthetase cytosolic isozyme 1

ATGCTCTGTTTCTTCAGTATTCTGGGATCTTCTTTGTCTTCCCATGTTCT

TGGTTCTGCTACTATGTTTTCTCTTCATGGAGATCAACCTATCGTTTCTG

TTTATAGTACTTACCCTTTTCAGAATTTCTTGCAATTGTGTGTTTTTCAC

TATTATATCATCGCTCGTTCACTATCAATGTTTTAGATGTATGTATATAT

TTATTTTGTGTGTGTGAATCTGTTGCTTTGTTTTCGATCTATTTCTTATG

AAAAACCCTTTTTTTACAAGATCTAAACTTTACGTTATTTATTTATTTTA

ATTGTGAATGGAGTTTCATTAGCAATTCTCTGTTCTGATGAGAATCATGC

CTTTTTTCTTTGTTTGTAGGTGGAAATTTGATGCTTTGTTTAAGGTTTTT

TATTTATTTTTTATTTTTAGAATCAATATATTTTCTAAGATTTCATACAT

TTGTTATCACTTGGCTTTAATCTTCAGTTTAGTTTTGTAACCACTCTTGT

TTCTATGGAGATCTGGTATACTATGTTGTTGTGATTGATATGGGCTAATC

TTTCCATGGTTTTTAAAACTAGGCTGTGTGTTTGGTTCATGAATTTGGTG

AGAGATTTGGAAATGAAAAAAAGAAAAGGTGTATAAAATTAAATGAAATA

ACATTTTACCTACCTTTTCTATTTTCACTTTCCTAACACAGCTTATGTTG

TAGTTTTATTGTTGATGAGTATGTTCTGTGACTTTTTTTCAGTGCTATTA

ATTATTTCCCTTGTTTATCTAGAATTTTTCAACTTTGATATTTAATTTTG

AATCTGTGAATAGGATTGGTGGTTCTGGCATGGACCTCAGAAGCAAAGCA

AGGGTAATAGCTTTTCTCCCTAATAAAAAATTGTTTCTGGTTATTGTGTA

TGGTATCTAATGTAATCGATTGTGCTATATAGACTCTTTCTGGACCTGTG

AGTGACCCCAAAAAGCTTCCAAAGTGGAATTATGACGGATCTAGCACAGG

ACAAGCTCCTGGTGAAGATAGTGAAGTCATTATCTAGTATGTGATCTTGC

TTTTCAATTTCTTTTACCATTTTTTACACAATTTTCCCTTACATAGTGTT

GTTTGTATATTTGGGTTCACTTAACAATAACTGAAATTAACTTTTGTAAA

TTGGTGATGTAACAAACACAACAGCCCTCAAGCAATTTTTAAGGACCCAT

TCAGGAGGGGAAACAATATATTGGTGAGTTAAACTTGTATTTTTGCATTC

AGTTCTTTTGGGTTTGGGGGGCACTTCATTGCGATGTCTTTAACAAAATA

CTAAACAAGTTCGTTCATCTCTCTTTGTTGTTATCTTAGGTCATGTGTGA

TACTTACACACCTGGTGGTGAGCCAATTCCAACAAACAAGAGGTTCAACG

CTGCAAAGATTTTCAGCCACCCTGATGTTGTAGCTGAAGAACCTTGGTAA

CTATTATTTTATACCTCTCATAGTGTTGTCAAATTTTATTCTCATTGTTG

TATTTTACGTGCATCTTTATCCCCCAATATTTTTGGGATGTGGTAATTTA

CTGGTAATTCATGTTGTAAAATAGGTATGGTATTGAGCAAGAGTACACCT

TGCTCCAAAAACAAGTTAAGTGGCCGCTTGGCTGGCCGCTTGGAGGTTTT

CCAGGACCTCAGGTAAATGAAACCGATCCTCTTCTAAATACCAACAATTT

GCAATTTTCTATCTTGTCTTAATTGCAGTATTTTTTTTCTTGTTTGATGT

ACTGATTATTGTAATTTACTTTTGGTTGTAAATTGTTTGTTGTTTGATGT

ATTTTTAGATTATGATTTCTAGTTATTAACTTTTGGTTTGCAAAAGACTA

CACTGCACCAAACAAGGAAATTTGATCTGATTTTTGTCGTGTATAATTCA

ATAGCGTTCAATGCCAACTTGTCTGGTAATTTAAGCAACTGCAAAAAGGG

ATTATGCTTTTTAATGCCTACATTAAATGCTTCTCCCAATTTAAATAGAT

CCCGAGTTTAGCAGCTGATAATTCTTGTGAGCAACAATGTTTCTATCTTT

TGTGATATGTTATGGTGAATCCTTTTTGCCTGAAACAACTGTAATTGAAC

CTCTTGTTGTTGCAACATAGGGACCATACTATTGTGGTATTGGTGTTGAC

AAAGCTTATGGACGCGATATTGTGGATTCGCATTACAAGGCTTGCCTCTA

TGCTGGTGTAAACATAAGTGGAATAAATGGTGAAGTGATGCCTGGCCAGG

TAAAAGGAGTTACTTTTGAGTTATTTCTTTCATTTTTCTCCTCCAAATCT

GAATCACTGTTGTTCTATTCAGTTGAAACTCAAATATTTCGTTGTTTTAT

AATACAGTGGGAGTTTCAAGTTGGACCTTCTGTTGGTATCTCAGCCGGAG

ATGAATTGTGGGTGGCTCGTTACATCCTTGAGGTAAAGTTCCTAGTTGAA

ACTACTTACTACTATGCTAACTATTAATTTTATTTAATTTTATTATGTAG

GATTTAATGATACAAAGTTTGTTCTAAACTGCTTTTTATTTTCCTTTATT

GACAGAGGATCACGGAGATTGCTGGGGTCGTGGTTTCCTTTGATCCCAAA

CCTATCGAGGTTAGATTTTTCATAATTTACTTTGTTCTTCCTTCATTATC

CCCCGAGGATCTTGATTTTCTTTCAACACTTGGTTATATACAATAGTTTT

GCATTTACTTCAATATCGCTATTATATTCGTTCTAACTCTCACTGCGGGC

TTCTTGTATATCAGGGTGATTGGAATGGCGCTGGAGCTCATACCAATTAC

AGGTAATAGATCAAACTTAAAATAGGGACTGACGGCATAACATGAAATTT

GTTCTGCTTTGTGCTAATGATTGAATGTTAATCCAAATTTCAGCACCAAG

TCTACGAGGAGTGATGGGGGTTTTGAGGTCATCAAAAAGGCCATTGAGAA

GCTTGGCCTAAGGCACAAAGAACATATTGCTGCTTATGGTGAAGGCAACG

AGCGTCGTCTCACTGGACGACATGAGACCGCCGACATCAACACCTTCTTA

TGGGTAAATAATTTGTTTTACAATTTTTTTGATGGTTGAAATCTTGCACT

GAGAGATCACCAAAAAATATTTTCTCTATATATGAGTGCTTGTGGATATT

ATTTCATTCGTTCTTGCATTTAGTGCCCAAAAAAAAACAAAAAGTCTGAA

TTGGGAAATTTTTATTTTCTATGAAAATAGAAGAAATCCATTTTACTTAA

TTTATATCCAATTTTTATAACAGCTAGTGCATGGACCTAGCTCATGTTCA

TAATACAATGCCATCCTCATGGTAATTTTTTGTTTTTCTTCCAACAGGGA

GTTGCAAACCGTGGTGCATCGATCAGGGTCGGGAGGGACACAGAGAAAGC

CGGAAAAGGGTATTTTGAGGACAGGAGGCCTGCTTCGAATATGGATCCAT

ATGTGGTTACTTCCATGATTGCAGAGACCACTCTGCTGTGGAAGCCATGA

GTACGAGCGTTGTTCACTTATGCTATGTTTGGATGGGGATTTAAGGATGG

ATTTGGAAAAGAAAATAGGAAAGGTATGTGAAATGTGAAAAAAATATATT

TATATTTCACGTAGTTTCACACATTTTTTTCCTTTCCTTTTCCAAATCTC

TTTCAAAATCTCCCAACCAAATACAGCGTTAGCGACGGCTACATTTGAAT

TTAATATTGTGCTCTGCTCTGTCTGTTTCATTGTTTAGTGATTTCAAAGA

GGAAAAGAAATTGTATTTCATAATCCCAACCCAACCTTTGTGTCTATTTT

TCCAAATTGAAGTAAGTGGAGAGTATGTCTTGTTATTTTCTTGGTACTAG

GCTCTGTTTGTGTCTCGAGTAGCAAACATGATTTCCAGTCTCTGTCTTTG

AATAAAAACAGTAACATTTTACATTAATAACAATATGAAGGAGGGGTTGA

ATT

>TEA016839.1 locus=Scaffold180:570205:573569:- probable aquaporin TIP-type RB7-18C

CAAAAGTACAAATTTTTTTAGTGAATTAGTTCACTTGAGAGCTTAATTAG

TTCAATCTCTGTGGGTTTTTGAGTGATAAAAAAAATGGTGAAGTTGGCTT

TTGGTAGCATTGGTGACTCATTCAGTGTTGGGTCACTCAAGTCTTATCTA

TCTGAGTTCATTGCAACCCTTCTCTTTGTTTTTGCTGGTGTTGGATCTGC

AATTGCTTACAGTCAGTGCAAATATTTGTCATGATTTTTTTTTAATTAAA

AATAATAATAATATTTCACTAAACAAATTTTACCATCTTTTTTCGACTCT

TATGTGAAACACGGGTACAAATAGGAATACTTTTTTACTAAATAATAATA

CTTTTTTCAATTATTATTGGATATCATTTCTTTTTACGCTACCTCTCAGT

TATTACTAGCATTTTATACAGTTTTAACCCCTCAATCCAACCAAAAAAAA

AAAAAAAAAAAAAAAAAACAGCTTCAATTGTAAAGGGAAAAAAAAAAAAA

GAAAAAGAAAAAAAAGAGAAGGGGGAATAGCAAATCAGTGACAACTTCAA

AAATAAATATCGATTTTATTGTAACAAAGTTTTTCATTAATTTATAAATA

TTTTCCGTATTTCACTGAAATGATATTTTCCTCCCCCCCAAAAAAATGGC

AAACTATAATTTTTGGCTTAACAATAGGTCTTAAATTGAATTTCATTTGT

CAAAAGATATATATAAATAAATAGTTATATATAATATGATGATGTGGCAG

GTAAGCTGACATCAGGAGCAGCATTGGACCCAGCAGGGCTTTTATTGTAA

CAAAGTTTTTCATTAATTTGTAAATATTTTCCGTATTTCACTGAAATGAT

ATTTTCCTCCCCCCAAAAAAATGGCAAACTATAATTTTTGGCTTAACAAT

AGGTCTTAAATTGAATTTCATTTGTCAAAAGATATATATAAATAAATAGT

TATATATAATATGATGATGTGGCAGGTAAGCTGACATCAGGAGCAGCATT

GGACCCAGCAGGGCTAGTAGCAGTGGCCGTAGCCCATGCATTTGCACTCT

TTGTTGGAGTGTCCATGGCAGCCAACATCTCAGGTGGCCATTTGAATCCA

GCTGTCACCTTCGGATTGGCCATCGGAGGCAACATCACCATCCTAACTGG

CATCTTCTATTGGATTGCCCAAGTTCTTGGCTCCATCGTGGCTTGCCTCC

TCCTCAAATTCGTTACCGACGGCATGGTATCTCTATCTCTCTTAAGAAAA

TTGTTAAAATAAACAAGTATCATAACATGAATGCTGTAAAACAATACTCC

AAGACTTACTCCTATATAATTAGATGATTAAAAGGGCAAATGTTTGAGAG

TTTGCGTTGAAACTCAGGACAAGATTCTTAGTTATTAATTTTAAATCATT

AATATTTTGTTAAACAAGTATCATAACATGAGCGGTGTGAAACAATACTT

TAAAACTTACTCCTATATAATTAGATGATTGAAAGGACAAAGGTTTGAGA

GTCTACGTTGAGACTCGGGGACAAGACTCCCGGTTATTAATTTTAAATCA

TTAATTTTTGGTCATTATATCTATAATGAGACTCGGGTAAGTTATGGTTT

AAGTCAATTTTGAGAAAGTAAGTGCTTACAGATTCCCTACATTTAGGGAT

GGTAACAGAGATCCTACTCCATGGATACCCATGAAGATGTGTTGTTGCAC

ATCTTTGTCATTAGACTTGTTTAAAGCCCTCATGTCCAACTTTCAGCTAG

TAGAGACTCCTATAGTCATAAGCAAAGAGAGTTGCCACACGTTGATTGCT

CCCTCCTACACCCCTTATTTAATGAGAAATAAAAGAAAACTACCAAAAAA

AAAAAAAAAAAGGGGCCGAAATTGATTTCACTAATTTTTTCTTCTCTTCA

TAAATTCACACACAGTTAATATCACACCATATTAAAAAAATTAATGGTGA

ATTCCCTATACTTTTTTGTAAAACAAAAATAATAGACTTCCAATTAGGCA

ATAGAACTAGTTGTGCTTTCCTTCATTACCAGTTATTTTATATATACAAC

AGTTACAGACTCAACCAATGATGAAATAAAAAATAAGATAATTACCTAAC

CTAGTATCGAAGACTTCTACCACTACTTAATTAATTAAAAGATACATAAC

ATTATACCAGTGAAATTATCTTTAAATCCAATTTTCCCACGTAACAATTG

TGTGTGGCATTTCACAGGCAATCCCAACCCATGCAGTCTCATCCGGGATG

AATGCCTTCGAAGGAGTGGTGATGGAGATAGTCATAACCTTTGCCCTAGT

TTATACTGTCTATGCCACCGCCGCAGACCCCAAGAAGGGCTCCGTCGGCA

TAGTCGCACCCATCGCAATTGGGTTCATCGTTGGTGCCAACATCTTAGCT

GCTGGTCCATTCAGCGGTGGCTCGATGAACCCAGCTCGCTCATCTGGGCC

AGCTGTGGTCAGCGGAGACTTCTCTGAGATTTGGATCTACTGGGTCGGCC

CACTTATTGGTGGAGGCTTGGCTGGCTTGATCTATGGTGATATCTTTATT

GGGTCATATGGTGCACTCCCAGCCTCTGACCAGTTATTTTATATATACAA

CAGTTACAGACTCAATCAATGATGACATAAAAAATAAGATAATTACCTAA

CCTAGTATCGAAGACTTCTAGTACCACTAACTAATTAATTAAAAGATACA

TAGCATTATATGAAATTATTTTTAAATCCAATTTTCCCACGTAACAATTG

TGTGTGGCATTTCACAGGCAATCCCAACCCATGCAGTCTCATCCGGGATG

AATGCATTCGAAGGAGTGGTGATGGAGATAGTCATAACCTTTGCCCTAGT

TTATACCGTCTATGCCACCGCCGCAGACCCCAAGAAGGGCTCCGTCGGCA

TAGTCGCACCCATCGCAATTGGGTTCATCGTTGGTGCCAACATCTTAGCT

GCTGGTCCATTCAGCGGTGGCTCGATGAACCCAGCTCGCTCATCTGGGCC

AGCTGTGGTCAGCGGAGACTTCTCTGAGATTTGGATCTACTGGGTCGGCC

CACTTATTGGTGGAGGCTTGGCTGGCTTGATCTATGGTGATATCTTTATT

GGGTCATATGGTGCACTCCCAGCCTCTGAAGATTATGCTTAAATAGCCGA

AAAAATGCAGAAATTATCACGTGGATCGGGAACACTGGCGTGATCATGTG

CCTGTTTAATTACAAGCATTCAAGTGTTGGGCATAAAGCGACGTGGTGTT

TTGGAAACACCAGATAACTTCTCCGAAAAATGGGGACTATCTCCTTGTGC

TGTGCTGTGTGTGTCGATTGTCAAATGTTTGTGGTGTCTTTCTTTTGGTG

TTGAGGTGTGCTTGT

>TEA009357.1 locus=Scaffold237:1334740:1337641:- Plasma membrane intrinsic protein PIP2;3

ATGGTGTCTGCAAATAGAGAAATGGTGGTGTATTGCTTCGACACCCTCGT

CGCTCGCTACAACAACAACCAACCCCCTCCGCTAGCTTTCGACGATGGTC

AACAATACATTTTATGCTTTCTCTCTCTCTAAAACCCTATTCTCTCTCTC

TCTCTCTCTCTCTCTCTCTCTCTCTGTAGAAAGTTGTATTATATAGATTA

TGGCGAAGGATATGGGGGTGGCGGAGCAGGGGTCTTACTCCGCCAAGGAC

TACCAGGATCCGCCACCGGCACCGCTAATCGACGCGGAGGAGCTGACCAA

GTGGTCGTTTTACAGGGCTCTGATTGCGGAGTTCATTGCCACGCTTTTGT

TCCTTTACATTACGATTTTGACGGTGATTGGTTACAAGAGCCAGGTTGAC

CCGGTCAAGAATGCTGACCAGTGTGGCGGCGTTGGGATTCTCGGCATAGC

TTGGGCTTTTGGTGGCATGATCTTTGTGCTTGTTTACTGCACTGCTGGAA

TTTCTGGTGGGCAAAAATAAATACTAAAATTACTAAAATACCCATGTTTA

TTTGCACACACTCCGTAAATATGCATTTTACAATGCTATAGTGTTATGAA

AATTTATTTTTTATTTGCAAAAGCCACTTAGGATGAAAAAAAGTCAAACC

CTATCATGTTTTGTTGTCAATGCTGTTCTCGTTTGGCAATAGATTTTTTT

AATTAGTTTTTTTTTTTTTTTTTTTAACGTGAGATTAAAGAGTTTGTTTG

TGTTGATTTTTTAATTTTTGTGAGAGGAAAGTAAAATAATTATAGAAAAA

AATTAAATAATTATGATTTAAATGTGACGTTTTATTTTATTGATTTTTTA

ACATATTTTTATCATTCAATTTTTTGTTTGCCAAACAAAGCCATTGTGTT

GTTATACATTTTCTTATAAAAAAAAATACATTTTCTTAATAAATTACTTC

CTTATCACATAAATCAATTATTATTATTATTATTAGGGTAAATTATACTG

ACATCCCTGGAGGTTTATATTTATTACAGTTCATCCCCTCACATTTGAAA

AATTATACTGACCCTCTTGTTAATTTAATTTTATGTATGATGATTATTAC

GGCGAAGGAAAGAGCTGTTATAAAACTTTAAACCTTAATGAAACTTTAAA

CCTCAGGAAGGAAAATGTAATTTTCAAAAGTTAATAAATATAAACCTCAG

GGGTGTCAATATAATTTACCCTTGTTATTATTATTATTATTATTATTATT

ATTATTGGTCTAATGGACAATAGATCACTTCCTTCTTAAAGGAGTGACAT

AACATTCAATTGCTTTGCTTAATTTCATTTTCAGAAATTTTCAATTTTTT

TGAGGCAAAAAAAAAAAAAAAAAAAAAAAAAACTTATCATTTTCATATAT

GTGTATATACATATTATTTTTTGGGATAAAATAATTTTTAAAATTTTAAA

AATATTTTTTTAATTCAATTATAAAAAAAAAAAAAAAAAAAACCAAACAA

GATAGGATGAGTATTAAAACTTTATATTAAACATAAATTATTATTCAAAA

TTAATTCCTAAATTGAGCACAAAACAAAGAGAAGGGTGTGCACCTTTATG

TGCACATAATAGGCTTTTTGTGAAGGAAGGGGGATGCAATGAGTTATTTC

AACTCAAATTCTCTCTTTTTTGTTATTGTTTTTTTTTAAATTTATGATTT

TATTAGATGCATTTAGAAAGAAAGAGAATGGGGACATATTAATGTAATTT

ATTTTTTTAAAAAAATAATAATAATTGTGAAATTAATGTGGGGAAACAGG

AGGACACATAAACCCAGCAGTGACATTTGGGCTGTTCTTGGCTCGAAAAG

TGTCGCTGATTCGAGCAGTGATGTACATAGTGGCTCAGTGCTTGGGTGCC

ATATGCGGAGTTGCGCTTGTGAAGGCCTTCCAGAGCTCCTACTACGAAGT

CTACGGCGGCGGCGCTAACGAGCTCTCCCCCGGCTACAGCAAGGGCACCG

GCTTGGGCGCCGAGATCATCGGAACATTCGTACTCGTCTACACGGTCTTC

TCCGCCACCGATCCGAAGAGGAGCGCCAGAGACTCCCATGTCCCTGTAAG

CAATCCCCCCACCACCCTCCTCTACACAAATTATATGTTCATTTTTTAAA

TTTCTAAGTTAATAGATTTAAGTTTGAAATATCTATTTAAAAATTTACAT

ATTTGCCCATACAAATGTCATCTATCTATATACGTATTATCTATTTATCC

AATTATCCGTCCAAATGTCACAAGGCGCACACCGGTCACAAGCCAATTTG

TTAGCGACCACAAAATACATAGGAGTGACTTGTCACCAATTAACTTAAAT

TTAAGCCATGAATTTAATGTTGTTTTAATAATGTGTGTTGATTTGTGAAT

TGAAAATGAAGGTATTGGCACCACTTCCAATTGGGTTTGCTGTGTTTATG

GTTCACCTAGCCACCATTCCGGTCACCGGCACTGGCATCAACCCTGCTAG

GAGCCTTGGAGCTGCTGTTATTTACAACCAAGAAAAGGCTTGGCATGACC

ATGTCAGTAAATATATATCTTCCCTAAAACCCATTTAAATTACATTATTA

CTTCTCTATATTGTTTTGCTTATCAAATATTCTCTGTTTATTTATTTATT

TATTTTTTTGCAGTGGATGTTTTGGGTAGGACCCTTCATTGGTGCAGCCA

TTGCAGCCTTCTACCACCAGTTCATCTTGAGAGCTGGAGCCATTAAAGCT

CTCGGGTCATTCAGGAGCAGCTCCCATGTCTGATTTTTCAGAAAGTTAAA

AAAAGAGAATAATATTGGAGGGGTGCTTGAGTCTGTGATCTGGAAGTCTC

TCTCTCTCTCTCTCTCTCTCTCACTACATCACTATTCAAGATTTAAGGGT

AG

>TEA032123.1 locus=Scaffold507:1538684:1544380:- Glutamine synthetase

GTAATACTAATCAACAATCACAAACACACAGATAATATAAACGCAGAAGA

GATAAGAGAGAGGGAGAGAGGGAGAGAGAGAACCAGAGCAATTTGTGATA

GTGTGAAAACATGGCTCAGCTTTCAGATCTCATCAACATGAACCTCTCCG

ACTCTACGGAGAAGATCATTGCCGAGTACATATGGTGAGTCTGTGTTCCC

CGTCTGTTCTGTTCTGTTTTTTTTTTTTTTTTTTGGTCTGGGTTGTCGTT

ATTGTTGTTGGGTTTGCTTTGTTTTTCCACCCATGTTCTGTTTCTTCACT

ATTGGTGGGTTTGCCCTGTTTTCTTTGCTTTTTTTTGGGCTTTGCTTTTC

GATCCAATGTTGCTGTCACAAATGTGTCGTTCTCATGGCATGCGGTTTCT

ATTCCTCGCGATCCACCAATTTTTTTTTGCCATACATCAGATTTATTTTA

TTTTATTTATTTATTATTACTATTATTTTTTTTTTTAAGTTTCCACTGCC

ATTGATCTTTGTTTTTAGTCTTGGTTATTTTTGTTTTGTTTGTTTAGCGA

TCTGGGTTCATACCGTTCTGGTAGCAAATGGGTATGCTCTGTTTTTTATT

TGTTTCCCCTTAGATCAGTCTTGTTGCAAGATCTCACCTTTTTGTTATCA

ATCATTGTTTTTTCATTTTCATCTTCATTGTTGTTCTGTTGTTAGACCAT

CTCCAACCCAGCAACTTTTCTTTCATCAAATTTAAATTTGATGAAAAAAG

TAACTTTTTGTTGCTCCGATGTTTCATCACACCAATATCAAATCCAGGAT

ATGTTAGAGCATCTCCAATGGTGTGCACAATTTTTTTTAATACCAAATTT

AAGTAATAACTCTATTTGATATTAAGTGTTTCAAGGGAAGATTTTGAGAT

TTCAGACCGGCCAGATGTCCGACTGGTTGACTGGCCGGTGTATTTTTTTC

AAAAATTTTTTGCCTAGAGGCGGTCATTCTCTAGTAGGACATCCCGGGAT

GAAAATTCAAAAGCTCTGAACAGTGATTTGATGAAAGTTCCCAGCGTCCT

CAATTTGGTGAAGGAATTTTTATATCTTCGATTTGATACTCATTTGTTGA

AACTATAAAATAGATGTTTTTCAATCAAATTTAAATTTGACGACAGAATT

AATGAACCATTGGAGATGGTCTTACAGTTTTTATCAAATCGAGGATGGCT

GTGGTTTTCACCAATTTACTGTTCAAAGTCGTTGGAGTTGCAGGTAGTAC

AACACCACCGGATGTCCTGGTTGTCCTTCCGGATGACTACTTCGGTGAAA

AATTGGTTTTCACCAATTTACTGTTCAAAGTCGTTGGAGTTGCAGGTAGT

ACAACACCACCGGATGTCCTGGTTGTCCTTCCGGATGACTACTTCGGTGA

AAAATTGCCCACAACATATTTCAGATTTGATTGAAAATTATACTGGCCGG

TCGACTGTATGCCCGGTCGTCCTCCCGGTCGATCAATGATTTGATGACAG

TCCAAAATTTTCCGATGGAGCGCGCGATTTTTATTCAATTTTTTAATGAA

AAAAATGAGTATACGCTGGAGATGCCCTTAGTCCATCTAATTTTCATATG

ATAACTCCAATTTTTGTACTTTTGGGTTATATTTTCATTGAGTAACCTTT

TCATTAGCAATAATTCTCTGTTCTTCTGTGTACAGTGCCCTGTTTGGACA

TAATCAATTTACTTGTACTTTTCTTTTAATCAAATGATTCATGAGATTTT

TACATATCTAAGGTTGTTTTAATCAGTTGATTCATTAGATTTATATATCT

AATGTCGTCATGTGGAGATTTCACTAATATTGTTTTAAGGAGAAATCTAC

AACGTCCCCCCCTTTTTTTAAAAATTTGCTCATGTTCAAAAAATGTTAAT

ATTTTGTATCACTTCCATGGAAGTTATTAATGTTTGTGGTGTGTTATGAT

ATTTGCCATAAAATTACAAATAGTGAATTTATGGTTTGTACCCTAAAGTT

ATGAATGGTGAAGTTATGGCATGTTATATTTTTGCCGTGATACCTCGTTA

TTGTTGCGATATTTTGTGGTGTTGAGGTGAGAAAAGTTATGAAGTACACA

CCATATACACCCTTTTGGGGTGTACATTGTAGCATCTCTTTTATTTTAAT

CATCTCTTTAATTGTATTGCTGTGTTATAGTCATCAGAATGTTTGCACAA

TTGGATACAAATTTCAGTTATTTCATTTGGTAATCGAGTGTTCATCTTGT

TTGTAGATGAAACTGTCATAGTCTTCTCTCAATATAGTATGCTTCATGAC

ATTTTTTTCTTTATTTGTGGTTGCCCTTTTTTTTTTTTTGATTGTTAAAA

TCTATAATTATACAACTTTGATTGTTAATTTTGAATCGTTGAATAGGATT

GGTGGTACTGGCTTGGACCTCAGAAGCAAAGCAAGGGTAATTGCTTTTTA

CTTATTTAAAATTTGTTTGTCAAGTTGTTCTTGTAGTTATTGTTCATCTT

CTTGATTGGTTTTAGTGATTTGTAATATATATGTTGTTGTTTATTCAGAC

TCTTTCTGGACCCGTTAATGATCCCAAAAAGCTTCCCAAGTGGAATTATG

ATGGATCTAGCACAGGTCAAGCTCCTGGGGAAGACAGTGAAGTGATCATA

TAGTATGTTATCTTGTTTTCCCAATTTTCATATCTTCACTTTAATTTGCG

CTTGCTTTTATGGTCTTATTTAGTCTCTTTCTAAATTTAAGTTTTACCCG

ATAAATGTTACGAGTGCTTACCTTTGTAATTTGGTGATTATGTATCAAAA

CAGCCCTCAAGCAATTTTTAAAGATCCATTCAGGAAGGGAAACAATATAT

TGGTGAGTTAGTGCATTCATGCAGTTCATTGGAATGGAGTAGTCATTCCA

TCAGAATAATCAGAATAATCATCTCATGTTTGGTTCGGTAAAAAGGAAAG

GAATTTAACATTGGAAATAAAAGCACCATTCACCAATTTAGGAGAATTGT

CATTCCGTTGGAAGGCCAAGGAATAGTCATCCCATTCCTTCCTTTAATCG

TTCCAATGAACAAAACATTTCCTCAATTTCATTTGGGTTTTGGGGCACTT

TCATTGTGAAATCTCAAACGATGTGTTAAAAAAGTTTTGTTGTGCTATTT

TTCTCATTAATCATTATTGTCTTAGGTCATGTGTGATGCTTACACGCCTG

CTGGTGAACCAATTCCAACCAACAAGAGGTACAATGCTGAGAAGATTTTC

AGCCACACTGATGTTGTTGCTGAAGAACCTTGGTAAAGGATTTCAAGCTT

TTCACAATATTTTGTCAACACTTAATTGTCACTGTTGTTTGAGTATCTTT

GTCCATTTAACTTTTGGAGACTGTAATTTATTTTGAATTTTAATTTTAAA

TTAGGTATGGTATTGAGCAAGAGTACACCTTGTTGCAAAAAGATGTTAAG

TGGCCTATCGGGTGGCCAAAGGGAGGTTATCCTGGACCTCAGGTAATGAA

ATTGATACTTTTTCCCAATATCAACAATTTCATTTGTCTATTCTGTCTAA

TATTTAGTGATCATGTCTTCCAATTTTAGAATTTTCTTGAACCTGGTAAA

TTAATTTTGTCTGCTGGTTTAGCCCTTGTGAACTTTTGGATACTGACATG

CTTTGCAACCCCTTGTGTGTGTCAAAAAGGATAAAATTACATAATATTAT

GTTAGCTTTTAATCTTTTTGAGCCACTTGTGCAATGCTATTAAAAAACAT

ATATAAAGAAGTCCTTATTATGCACCTTTTTAATTTGCTGTTTATTGTGC

AGAATTCAATTGCATATAATAGCAATTCTTGTAGATGCTCTGGTGAATAT

TTTTGTGGCTATTTCTTTTTGCCTAAGACTTATAGTTGAACCTTTTGTTA

TTGCAACATAGGGACCATACTACTGTGGGGCTGGTGCCGACAAAGCTTTT

GGACGTGATATTGTTGATTCACACTACAAGGCTTGCCTTTATGCCGGGAT

TAACATAAGTGGAATCAATGGTGAGGTGATGCCGGGCCAGGTAAAATTAA

TAATTTTCAATTGTCTCCTTATGTTTTTGTTCTCCTGATCTGAATTCCTA

GTATTCATCAGTGCAATCGATAGTTAAAACAAATATTTCTTTGTACTATA

CAGTGGGAGTTTCAAGTTGGACCTGCTGTTGGCATCTCAGCCGGGGATGA

ATTGTGGGTGGCTCGTTACATTCTAGAGGTAGAGTACCTAATTGAAATAC

GTACTACTTCTAACCACTAATACTTTTTTAATGGTGTATGAATATGAGTA

ATACATGTGTTATCTAAACTGCTTTTATTTTCTGTTATTAACAGAGGATC

ACTGAGATCGCTGGGGTTGTGGTATCTTTTGACCCCAAACCTATCGAGGT

TAGATTTTTCATGTTTACTTTGCTCTTCATCATTCCCAAGCATCTTAATT

TTCTCATTGTTAAATTTACTGAAGGAGGTCGGAACATCTGGTTTTGACAC

TTCTTGAGCACAATGAAATTGCATTTACCTTAATATCCCTATAGCTATTG

CTATTATTGCCATCTTAAGTCGAAATCATGGTTTTTTGCACACCAGGGTG

ATTGGAATGGAGCCGGTGCTCATACAAATTACAGGTAATGGATGAAATTT

CATATAAGGGACTAATGGCACTTTACATATTTGGAATATTTGATGAATTT

TAATCCTAATTTCAGCACCAAGTCTATGAGGAAAGATGGAGGTTATCAGG

TCATCAAAAAGGCTATTGAGAAGCTTGGACTGAAGCACAAAGAACATATT

GCTGCATATGGAGAAGGCAACGAGCGTCGTCTCACTGGACGACACGAGAC

AGCTGACATCAACACCTTCTTATGGGTAAATAATTATTTTTCCGCTTCAA

ATATTCCAAATTTGTTATTGTTTTATATATTATTTAGCTTGCATCATTTA

GATCAATTATGAAACAATGCTTAGCGTTCACAACAAACATTCCAAACATT

CCTTGATTCATTCAATTGCAGTCATTTTGATTTTCTTTTGGTCCCCCATT

GTAATTTATGCAATAATAAATATGCCTTTAGTCTGAATTGTAAAATTTTC

TTTTTTCTATGAAAATTCAAGAGCACATTTTTTGCTTTTACATCCAATCT

CTATGATTGATTGATATACCCTTTCAAGCCCGACAAACCACTTGAGGATA

TGATTCTAGTGATTTTTGTTGTCATGGTCTAGTTTTTATGACTAGTACTC

CCTCCGTCCAAAATTGTTTCATTATTCCAGATATTACACAAAACAAAAGT

GATTTATATTTTGTAATTTATTATTTTTCAATGGTTTTAAAGACTTATCT

ACAAGAATTGAGATCTTTTAATTTGTGGAAATTATTTTTTTAACAAGTCA

TTATTTATTGACCCACCAAAAATGGACAAATAATTTGAGACTGAGGGAAT

AACTATTGCAATGGACCTAGCTCATGTTCGTAATACAAAGCAAACCTGAG

AGACTTTACTTGTGCTCATGCTTTATTAATTTTTTTCCCACCAGGGGGTT

GCAAACCGTGGTGCATCTGTTCGTGTTGGCCGAGACACTGAAAAGGCAGG

GAAAGGGTATTTTGAGGACAGGAGGCCTGCTTCGAACATGGATCCATACG

TGGTTACCTCCATGATTGCAGCTACCACCCTCCTGTGGAAACCATGA

>TEA026779.1 locus=Scaffold529:1104335:1116450:+ Glutamine oxoglutarate aminotransferase

ATGTCGGTAGGTTCGACTTCTGCTATTAATCATACTCCAAACAACTCAGT

GACACTTTCTTTCGTTAAGCCTGTGAATCATCAGTTGAATATTGTGCCTC

TCAGTCGAGTTGGTGTATCCAGAGCATACTCTCTTGCCTCTTCTTCCTCT

TCTCTTGCTAGAAGATCGAAAGTTGTTTTGCAGAATAAATTCTATGGAAC

ACGACTACAAACATCTGGTAGTGAAAGACTTAATGTTCGGCAATCGAATC

CAAGAGTGGTGGTGCGATCGGCATTGTCGCAAGTGCCCGAAAAGCCTCTC

GGTCTCTACGATCCATCGTTTGATAAGGATTCGTGCGGGGTTGGATTCGT

CGCTGAATTATCCGGAGAAAGCAGTCGAAAAACGGTATGCATTAGATCAG

ATAGATACATAGACAGATATAAGCAAGAAAAAGAAGGTTTTTTTTTTTTT

TGAGTGTTTTTGGTTTGGTTTGATTTACAGGTGACAGATGCGGTGGAGAT

GTTGGTGCGGATGTCACACAGAGGTGCTTGTGGTTGTGAGACGAACACTG

GTGATGGAGCTGGCATTCTCGTCGCTCTTCCTCACGAGTTCTACAGGGAG

GTACGTATGATAATATACATCAATCTTTTAATATCTACTTTTTTTATTTT

TAAACGATTCATTAGCTTTTCTTTTCATTTATAGTTTTGGTGATTTTCAT

ACCTCATTTCAGAACATAATAGTTAGATTATCTCCAATTCCGTACTTTTT

TATTCTTCTTGTTATTATTATTATTATTATGAATAAAATTTGAGTGTAGA

TTTAAAAGCTTGATCGGGTTCGGATCAAATTCTTAGTTTTTTATTATTTT

TTTATCAATTTTTTTTTTCAAAAAAATTAATTTTTCAAAAAAAAAAAAAA

ATAATTATAAACTTGATTAACAAATTTTTATGAAAGAAAATCATTTCCAA

TTCTTACTGTTTTTGAAATTTTCATCAAATTTGACTAAAAATATGACTAT

TAGAAATTTGATTCAACTTTGATTTGGTGAAAATTGTGTCTCTGTTGATT

TGAAACAATTTTGATGAAATGATTGGGGCTCACTTGCCTTTATTCATCAA

CTTTGAATTAAACGAATGAATAGGTGAAAGATTGGAGATGTTATGGAATG

AGGTATGATTTTTTTTTTTTTTTAATTGTAATAATTATGATTCCAGGCTT

AATTCTATTGATTTTATTTGGTTATCTTGTGTCAGGTCGCCAAAGATGAA

GGTTTTGAGCTACCACAGCCAGGGGAATACGCTGTGGGAATGTTCTTCTT

GCCCACAGCTGAAAAACGAAGGGCACATAGCAAAAACGTATTTACAAAGG

TAAATTTAAGATCAAAACTTGTTCAGTTTATATTACTTTCTCTTCTCTTC

ACTTGAATGTGTAAATGAAGTGCATATGTAATTGCTGAAGTGAATTTTGG

TTTCTTATCCTGGACTTGTATGTTTTGTTGTTCTTGGCAATGTATTGTCT

CCTAAGAAAATTCATGATTGTATTTCTAGGTTGCGGAATCACTTGGGCAT

ACTGTTCTTGGCTGGCGTTCTGTCCCAACAGATAACTCTGGATTGGGTAA

TTCTGCTTTGCAAACAGAACCTGTTATTGAACAAGTGTTTCTTACACCAA

ATCCCAGGTCAAAAGCTAATTTCGAGCAACAGGTACTAGTCTATTTCTAT

TTAGCTATAATCCATTTATCTTTAGGGGCAAAACTGGGAAATTCCGTGTC

TTTTGGAGGTCAACATAAAGCTAATCCATTTATCTTTAGGGGCAAAACTA

GGAAATTTAGTGTCTTTTGGAGGTCAATTTAAGGTTTATGGTCCAGTGTT

ACAAGAAAGAAACATCAGTTGGCTTTATAAGAATCAAGATGCATCAGTGG

ATGAGCCTAATTAAATGATATGTCATAGCATCGACACAAACATGTCATAG

CATCGACACAAATTGACGCATCACAAGTTCTAATTGCTTCTAATACAATT

TTTTCCCCCCCTTTCAAATTCATTGACACATCACAAGTTCTAATTGCTTC

TCAATACAATTTTTTTTTCCCTTTCAAATTCATTAAAATTTTAAAGCGGT

ACTGCAGAACATTTATGAGCCCCTTCTAAAAGAAAATTAAATTTCAATAA

AAATTCAGTTCATTCGACTTGTACACTTCTTGTGCAACCTGCTTTTCTAT

GCTCTATAGCTTTGTACGTCACAGTATCTGATCTGTTGTAGTGCAGTTAA

TTTGCTTGATCAACTTGGGTGCAGGACTTTGAGAATTAGCCTTATTATAC

TCTCTCTCTCTCTCTCTCTCTCTCTCCTCCTTTTATTTAAAAAATAATAG

TAATAATAAAATAAACTTTTAGTCCATGAATACAGCAATTTTATGGAAGT

CTGCCTTTTTTCCTTTTATTTTGTTGCAGTTATATATATTAAGGAGGCTT

TCAATGGTAGCAATCCGAGCTGCTTTGAGCCTTCAACATGGCGGAGTTAA

AGACTTCTATATTTGTTCTCTCTCATCAAGGTTTGTTTTTCAGTAAGCCT

TTTTCTTTCAAAGAAAGTATAAAATCTTGGTTGAATGCTACTCTGCAGCC

TGATTATAAGACTTGTTCTGGTGTTGTTTCAGGACTGTCGTCTACAAAGG

TCAGTTAAAGCCCGACCAATTGAAGGAATACTATTATGCAGATCTTGGCA

ATGAAAGGTTTACGAGCTACATGGCCCTGGTAAATATATATTTCATTCAC

TTTTCTTCTTTGTTCACTGACTCCATACTTCAACAACGAACAGCATGGGA

TCAACCAAGGGATGCAAGGTTGAGGAATGACTTCTGAGGCATTGAAGTAT

GAATGATCTCTTAAGTTCTTCCACTCCCTTGATTACTGTTTGAATTTGGA

TGCCGGATTGGATTCTTTTGGTGAGTTGGTAAGGAACCTCATTATCAAGT

AAATCGTGTTTGCAGAAACTTGGTGCAGTGTTCTCTATGATCAGTTGAGG

ATAACTTCCATTTTAGTGGTGGAAAAGAAATTGTGTTTGGTCAATGGTAG

TTTAGGTTTTCCATGTTTTCATGTACTGGCGCTGCGGTATATTGTTTACT

GAAGTGCCCTGAACACAATTTCATGCTTACACTAGTAAAGTTAAATCCCA

TATCGTCAATAATGTGTAGGAAATTGGGGTTGAAAGTTTTGCATCTCTTT

TTTCCTGCAGATACACTCGAGATTCTCAACAAACACATTTCCTAGCTGGG

ATCGTGCTCAGCCTATGCGTGTCTTAGGCCATAATGGGGAAATTAACACA

CTTCGGGGCAACGTAAATTGGTAAGTTGTCAAGCAAGTAGTGGATTAATT

GTTCCTGAATAATGGAGATTTTGATGCCATTGTTAGTGGAACTTGAATAC

TTCTAATAATAACCTTCACACCTTAACAAATAGGATGACGGCACGTGAGG

GTCTTCTGAAGTGCAAGGAGCTTGGTCTGTCAAAGGATGAGATGAAGAAG

CTTCTACCCATTGTAGATGCCAGTTCATCTGACTCAGGTTTGATTCTAAT

CTAACTCTTATGTTTTTGTGTGCTACCATACATCCATATACACATGCATC

CCGCTTAATTTGAATAGTGGTGCAAAAATATTCATAAGCAGCATGCAGAC

ACTTAAATTGCCAGTGTAAGGAGGAGAAATAGTGCCACTTCTCGTGCTTG

AGCCTTCATTAATTACCTTATATCTGAATTTCTCTTTTCTCTTAGGTGCT

TTCGATGGTGTTCTTGAGCTTTTAGTTCGAGCTGGTAGAAGTCTTCCTGA

AGCGATCATGATGATGATTCCTGAAGCCTGGCAGAATGATAAGAATATGG

ATCCTCATCGGAAAGCCTTTTATGAATACTTTTCAGCCCTCATGGAACCA

TGGGATGGGCCTGCTCTGATATCATGTAAGAGTTCTACAAACTCTTTGAT

TTCACATTGTCTTACTTCCTTGCAGCATTTGGTTTGTGCTTACCAAAAGC

AATATGCAGTTACTGATGGTCGCTATCTTGGAGCTACACTGGACCGAAAT

GGATTACGTCCAGGTCGATTTTATGTCACACACAGTGGACGAGTTATAAT

GGCAAGCGAAGTTGGTGTAGTTGATATTCCACCTGAGGATGTGTGTAGGA

AAGGAAGACTTAATCCTGGCATGATGCTTCTCGTGGATTTTGATAAGCAT

GTCGTTGTAGATGATGAAGAACTGAAGCAACAGTATTCGCTTGCAAGACC

TTATGGAGAGTGGCTTCAAAGACAAAAGATAGAGCTAAGAGACATAATAG

AATCTGTTAGTGAATCTGAAAGGGTTCCTCCAGCTATAGCAGGAGTTGTG

CCAGTGAGTATAGTTTCTAATTATTATGCTTATTCTTCTTTCCGCACATT

GAGGTTGCAGTCCTTTTTTGTCCTGCCCCTCTTCATGGCAGTTCTGAATG

TGGATTGTATTTATGTTATGTAACCAGGCATCTAATGATGATGACAACAT

GGAAAATATGGGCATTCATGGATTGTTGGCTCCATTAAAGGCTTTTGGGT

ATTTATCTCTTTTGCCTGCTCTTGCACTTTAAGTTCTTTTGACTGTTGCA

ATTCTGCAGAGAAGTAAATTCAAATACTGGCCTCTTGTGCAGTTACACTG

TTGAATCCTTGGAGATGCTACTACTACCAATGGCAAAGGATGGTATTGAG

GCCCTTGGTTCAATGGGAAACGATGCTCCATTGGCTGTGATGTCAAACAG

AGAGAAACTCACATTTGAGTATTTCAAGCAGATGTTTGCTCAGGTTACAA

ACCCTCCTATTGATCCTATCCGGGAGAAGATAGTCACGTCCATGGAGTGC

ATGATTGGTCCAGAAGGTGATCTTACTGAGACCACTGAAGAACAATGTCA

TCGCCTCTCACTGAAAGGTCCCCTTTTATCCATTGAACAAATGGAAGCAA

TTAAAAAGATGAACTATAGAGGCTGGCGCAGCAAAATTCTTGATATAACA

TATTCTAAGGACCGTGGTAGGAAGGGTTTGGAGGAGACCCTGGATAGGAT

CTGTTCTGAAGCGCATGATGCAATTAACGAAGGTTATACGACACTGGTGC

TTTCTGACAGAGGTACTACATATCTTAAACTAGTGTTGTTCCTGAAAAAT

ATATATTTCTGAATGTTCTTTATTCTTTAATAAAAATTATGTTATTATTA

TATGAAATAACATATTTGTAATTTGTTTAATTGTGTAGCATTTTCATCAA

AGCGTGTTGCTGTAAGCTCCCTTTTGGCTGTTGGTGCTGTCCATCATCAT

CTAGTTAAAAATCTTGAAAGGACTCAAATTGGGTTAGTTGTTGAATCTGC

TGAGCCTCGCGAAGTGCACCATTTTTGTACACTGGTTGGATTTGGTGCCG

ATGCTATATGCCCTTATTTGGCCATAGATGCCATTTGGAGACTGCAGGTT

GATGGAAAGATTCCACCCAAAGCAAGTGGTGAGTTCCACTCAAAAGATGA

GCTTGTCAAAAAGTACTTCAAAGCAAGCCACTATGGAATGATGAAGGTTC

TTGCCAAGATGGGGATATCTACTTTGGCCTCGTACAAGGGTGCTCAGATT

TTTGAAGCAGTGGGCCTTTCATCCGAAGTGATGGAGAGGTGCTTTGCTGG

AACTCCGAGCAGAGTTGAGGGGTCAACATTTGAAGTGCTTGCCAGTGATG

CCCTTCAGTTGCATGAGCTGGCATTTCCTACACGGGGCTTCCCTCCTGGA

AGTGCAGAGGCTATATCATTGCCCAATCCTGGTGATTATCACTGGAGGAA

AGGTGGCGAGGTCCACCTGAACGACCCCCTTGCTATAGCTAAGCTGCAAG

AGGCTACCAGAGGGAATAGTGTTGCTGCCTATAAAGAATACTCAAAGCGC

ATACATGAGTTAAATAAAAACTGCAATTTGCGGGGACTATTAAAATTTAA

AGAGGCAGACGTGAAGGTTCCTCTAGATGAAGTGGAACCAGCCAGTGAGA

TTGTAAAACGGTTTTGTACTGGGGCCATGAGTTATGGATCAATATCCTTG

GAGGCACACACCACCCTTGCCATTGCTATGAACACAATTGGGGGAAAGTC

AAACACAGGTATGCCCTTAATAAGATTCTCCCATAGTTATTTGTGTGAAG

TAATGTAATCTGAGCATGTTCTATCAGTGTTGAATAGTCTGAATGTTTGG

GAAGAATTTGGTGGTGTTTGGTTCAGGTGTACATCATGTTGTCGTCCTTT

ATGTTGCTTAACTTGTGGAAAGCATAGAATAATCATTCTTCTGTTATTGA

TGACTTTTCTTTTTTAATTTTTAATGTCAAAAAGTTATTTTGATAAAAAT

AATTATTCAACTCCCATGGAGCGTGCTTTTTGAACTCATTGTTTTGCATT

TCCCCCCCTCCTTGAATGCTCCTACAATATCCATTTATTTGTTCACTTCT

TCATTTAGTTTTGTAACCTTGTTTATCCATAAAATTGTCAGTTTTTTTTG

TAACTTTCCTTCTATTAGCGCCAACTTAAGTTGCTTCTCAAGGGTATTAG

CTTTTTCACATGCTTCTTTCCTTGAACTTACTTTATGCGACTTCTTTCTA

GGTGAGGGAGGTGAGAACCCATCCCGTATGGAACCTCTTCCAGATGGTTC

AAGGAATCCAAAAAGGAGTGCAATTAAGCAGGTTGCAAGTGGAAGATTCG

GTGTTTCAAGTTATTACCTTACTAATGCTGATGAGCTACAGATAAAAATG

GCTCAGGTATATTTTGATGACATACATTTGAACTTCAAATTTTCTTTCTT

GGTTTATGTTTCTGTTGTTATTGTTCATCCATCCCTGTTAGCCCCTGTAT

TCCTATTGGAATAGAGGTTCCTCTTGTTTATGAATTTATGTTCATGGACA

GATGATGCTGTGTCTGCGAAGCACCTCTGTGTGTCTTTGCATCTTTAACT

GATACCTACCTTTGGCATTTGGTGAGGATATGAACTGAACTTGTTTATCC

TTTGTTCATTGGATTTATTATTAATTTTTGACTTCCATAATGCTCAGGGG

GCCAAGCCTGGTGAAGGTGGTGAACTTCCTGGCCACAAGGTTATTGGAGA

CATTGCTGTCACTAGGAATTCTACTGCTGGGGTGGGACTAATCAGCCCAC

CTCCACATCATGATATTTATTCAATTGAAGATCTGGCTCAATTGATTCAC

GATCTTAAGGTAAGTACATGCATTGATGCTTCTACAAATTTCTATGGAGT

GCATGATGATTTTTATCTCATTGCGTGCCTTATAAGCAGCTTCTATGCGT

GCTCCAATATTGTCAGTTGCATAGTATAGTATACCTGACTTCTTTATCCA

TAATTTGTAGAATGCCAATCCAGGGGCTCGAATTAGTGTGAAGTTGGTGT

CTGAAGCTGGTGTGGGAGTAATTGCTAGTGGGGTTGTGAAGGGTCATGCT

GATCATGTCTTGATCTCTGGTCATGATGGAGGTACAGGTGCCTCCCGATG

GACTGGCATCAAGAGTGCTGGCCTCCCATGGGAACTTGGTCTTGCCGAAA

CTCATCAAACTCTAGTTGCTAATGACCTTCGTGGCCGAACAGTTCTCCAG

ACAGATGGCCAACTGAAAACTGGCAGAGATGTGGCCATTGCTGCACTACT

TGGTGCAGAGGAGTTTGGTTTCAGCACAGCTCCACTCATTACGCTCGGAT

GCATCATGATGCGGAAGTGCCACAAAAACACTTGTCCAGTTGGCATTGCT

ACCCAAGATCCAGTGCTTCGAGAGAAGTTTGCTGGAGAACCTGAACATGT

GATAAACTTTTTCTTCATGCTGGCAGAGGAGGTTAGAGAAATCATGTCTC

AGCTTGGGTTTCGGACAATCAATGAAATGGTTGGTCGATCAGATATGCTC

GAACTGGATAAAGAAGTGACAAAGACCAATGAAAAGCTAAATAATATTGA

TCTCTCCCTATTACTCAGGCCTGCTGCTGACATCCGGCCAGAAGCTGCCC

AGTATTGTGTACAGAAACAAGATCATGGTTTGGACATGGCTTTGGATAAT

GAACTTATAACTCTGTCCAAAGCTGCTTTAGAAAAAGGTCTTCCTGTGTA

CATCGAAACACCAATTTGCAATATAAATCGTGCCGTTGGAACAATGCTTA

GCCATGAAGTGACAAAGCGTTATCACATGGTGGGACTTCCAGCTGATACC

ATCCATATCAAGCTCCATGGAAGTGCGGGCCAGAGCCTTGGTGCTTTTCT

CTGCCCTGGCATCACACTGGAGCTTGAAGGTGATAGCAATGACTACGTTG

GTAAAGGACTATCAGGTGGCAAGATTGTTGTTTATCCTCCAAAAGGAAGC

AGATTTGATCCAAAGGAAAACATTGTGATCGGTAACGTGGCTCTCTATGG

AGCAACTGTTGGGGAGGCATATTTTAATGGAATGGCAGCAGAAAGATTTT

GTGTCCGTAATTCTGGGGTTAAAGCAGTTGTAGAAGGTGTTGGTGATCAT

GGATGCGAGTACATGACTGGTGGGACTGTTGTTGTGCTTGGAAAAACTGG

GAGGAATTTTGCTGCTGGCATGAGTGGTGGCATTGCGTATGTTCTTGATG

TGGACGCCAAGTTTCATTTGCGATGCAATCATGAGCTGGTAGATCTTGAT

AGAGTTGAAGAAGAAGAGGATATTATGACACTGAGAATGATGATACAGCA

ACATCAGCGTCACACAAACAGCCAACTAGCCAAGGAAGTGCTTGCTGATT

TTGATAATCTTTTGCCCAAATTTGTTAAGGTCTTCCCTAGGGATTATAAG

CGGATTCTTGCAAGAAAAAAAGAAGGGGAAATTTCAAAGGCAGCAGGGGC

TGCTGAAGAAGCTGATGAGCAAGAAGACGGAGAAGTGATGGAGAAAGATG

CTTTTGAAGAGCTTAAGAAGTTGGCAGCTTTGTCCTTGAATGAGAAAGGC

AATGAGGTCCATTGATTTGTTCATTTATTAATCTTTCTCTGAATCCTTCT

TCTATACTTGTAGTATTGTATGATTTTAATGATTGATGAGATTTGAGGTC

GTTGTCATAAAAAAAATGGATATAATTTGAAAGAAATTCCAGGTGAGCAT

TTTTTTTATTATTATTAGTAAATGCTTGAACTAGACTTGACATTTGGTTT

TGACTGGAATCTGCAGAAAGTTGAAGAGGCTGAACTGTTGAAAAGGCCAT

CTCGAGTTCCAGATGCTATAAAAAATAGGGGATTTATTGTTTATGAGCGT

GAAAGCATCTCGTACAGGGATCCCAATGTTCGGATGAATGATTGGAATGA

GGTCATGGAAAAAGCCAAGCCCAGCGCACTTCTAAAGACACAATCTGCTC

GCTGTATGGACTGTGGTACTCCTTTCTGCCATCAGGTGAGATAATTGCTC

TCTCTTACTTCTCATTTCAAAATTTCTTAAGCTTCTGCCCAATTATTTCA

GTTTTAGCCTTAATTTCTCTTTTGACTAAATTGTAGGAGAATTCGGGATG

TCCTCTGGGCAATAAAATACCTGAATTCAACGAGTTAGTGTACCAAAATA

GATGGCGTGAAGCATTAGATCGGCTTCTAGAGACTAATAACTTCCCAGAG

TTTACTGGTCGGGTGTGCCCTGCACCTTGTGAGGGTTCTTGCGTTCTTGG

TATTATTGAAAATCCTGTATCTATAAAAAGCATTGAATGCTCTATCATAG

ATAAAGCTTTTGAGGAAGGATGGATGGTTCCACGACCTCCCCTCAGAAGG

ACCGGGTATGCTTCTTAGCTCTTCCATACTTTCTGAGCAGATCGTTATTT

CTGTTTAACTTACCGGGAAAATAGATTCTAAATATCCTTGCTTTTACAGG

AAAAGAGTTGCTGTAGTTGGGAGTGGACCTGCTGGCTTGGCTGCTGCTGA

TCAGTTGAATAGAATGGGTCATTTCGTGACTGTGTTTGAGCGTGCCGATC

GTATTGGGGGCCTTATGATGTACGGAGTTCCTAACATGAAGGCTGATAAA

GTTGATATAGTTCAACGACGGGTTGACCTTATGGCAAAGGAGGGAATCAA

TTTTGTGGTTAATGCCAATGTTGGAAAAGATCCCTTGTATTGCCTAGATT

GGCTTCAAAAGGAAAACGATGCAATTGTTTTAGCTGTAGGTGCCACAAAA

CCAAGGTATGTTTGTCTTTTAATTTATTTATGAATAAAAAATCTCATTAC

AGTTTTCTTTAGAACATTGTAACAAATTGTGAACAAAATATTGGTTACAG

GGACCTACCTGTACCTGGACGGGAGCTATCAGGGGTCCATTTTGCTATGG

AGTTTCTTCACGCAAATACCAAAAGTTTGCTTGATAGCAATCTCGAGGAT

GGTAAATACATATCTGCCAAGGGTAAGAAGGTAGTGGTAATTGGTGGAGG

TGACACTGGTACAGATTGCATAGGAACATCTATCCGCCATGGTTGCAGTA

GCATTGTAAATTTGGAGCTTCTCCCTGAGCCGCCTCTAACCAGGGCTCCA

GGCAACCCTTGGCCTCAGGTTTGTAGACTGCATAATATGACAACTGAAAT

CAAGCAAAATGTAGATGTTTATTTTATTTTATTATTATTATTATGACCAA

GAACTCGGCCTAGGCAGGGTCATTTGGTACCACCCCAATGAAACAGTGAA

CATATATGTGAATGGGTAATCCATTAACAAAATGGTTTAAAAAATGGTAA

ATATTCAATTATGTTTTGATTGGCGCAAGTAAAAGCAACATGTCATAATA

AGAGTGCCTAAGATTTTAGAAAATAATCTATGATGCAGGTTGTTTTAATT

GGTGGATATTTTGGAATACATGTTGTGTAATTTAAGAAATGCAAAAATTG

TGAAAATCTCATCCCATTTGATGACTTCTTGTAGTCATTCAATGATTTAT

TCCTTTCAAATTTCTTGTTTTGCAGTGGCCTCGCATATTCCGTGTAGATT

ATGGGCACCAGGAAGCTGCTTCCAAGTTTGGCAAAGACCCAAGGTCTTAT

GAGGTATTGACTAAGCGGTTTGTGGGAGATGAAAATGGGAATGTGAAAGG

ACTTGAGGTGGTACGTGTCAAATGGGAGAAGGATGCTGGTGGCAAGTTCC

AATTTAAGGAAATTGAAGGCAGTGAGGAGATGATTGAGGCAGACCTGGTC

CTACTGGCTATGGGCTTCCTTGGTCCTGAGTCGGTTAGTATTCTCATCTA

TGACTCTAGTTTAATTTCTCTATGTTGAACCAATAACCATGTTCAGTTTC

TGATCAAGAGTAATTGTTGGAAGTGAGCTGGACAACTAGGATAGTTTCTC

TTACAACTTGGAAAATCTAAACTATTGGTCAGAATAGCGCAAGTTAAATT

TAGTTCCAGTCATTGATATATATTGTCTTGGTTCCTTTACAAAATTGAAC

TTATGCAATAATGAAAATTAATGGTAATTTTGTCATTTTTTTAAATAATG

TTCACCTAAATTTTTTCCTTAACCCCCAAATGGTCTTTTCGACTTCTTCC

AAAAGCCTGATTCAAATATGTTTGGTTTTGTTTTTAGAATCCCAAAAAGA

TGCTTTTGAATTTTCAAAAACTATTTTTGCACCTAAAAAAGAGATAGAGA

GAGAGAGAGAGAGAGAGAGAGAGAGAGAATAATATGAAAAAGGCAAGTGC

TTTTAAAATTTTCATTATTTTTTTTTATCAAATTAGTTTTAGTGTTTTCA

AAAAATAGATTTAGAAAACAAAATTTACTTTGGATGCTCCATGAAAATGA

ATCTATTCCTTCTCAATACATCACCAATATTCACATTCATATACATGTTT

ATGATAGAATTCTATGCAAACTGATATTCTACTATTTTTGTGATGCTCAA

TTCTTCAGACACTACCAGAGAAACTGGGCTTGGAGAGAGACAATAGATCA

AACTTCAAGGCAGACTATGGCCGCTTCTCAACCAATGTGGAAGGTGTGTT

TGCAGCGGGGGATTGTCGGCGTGGCCAGTCTCTGGTAGTTTGGGCCATCT

CAGAAGGTCGGCAAGCTGCTTCACAAGTTGACAAGTTTCTCATGAGGGAA

GAAAAAGACGGAACCATTGATCTCAAATGGCAGCAAGATAGCAACAAACA

AACAGTAATGACGTAG

>TEA006643.1 locus=Scaffold670:913595:918611:+ Flavone synthase

AAACAAGTTCCAAGCACATTCTAAAGGAACCACCCAAAAAATAGAAACAA

TTAAACAAACATGGAGGTAGAGAGAGTGCAAGCCCTGTCCCATGTAACTC

TCCATGAGCTCCCTGCAAAATTTATCCGACCGGCCCACGAGCAACCGGAG

AACAGCAAGGCTATCGAAGGTGTCACCGTCCCCGTGATCTCCCTCTCTCA

ACCACACGATGTGGTGGTCGATGCATTATCAAAGGCTTGTAGTGAATGGG

GATTTTTCCTCATCACAGATCACGGTGTCGAGCCCTCGTTGATCGGACGG

CTAAAAGAGGTTGGGGAGGAGTTCTTTAAGCTCCCACAGAAGGAGAAAGA

GAGCTATGCAAATGATCCTTCAAGTGGGAGTTTTGAAGGGTATGGAACAA

AGATGACTAAAAATTTTGATGAGAAAGTTGAGTGGATTGATTATTATTTT

CACGTCATGCACCCTCCTAAGAAGCTCAATCTTGACATGTGGCCTAAGAA

CCCTTCTTCATACAGGTATGTATGATTCACATAATCTTATATAAATTCTA

TCATGATTTTTCAAGTTTTAGTAATATTGCATATTCAGGGGAAAAAAATT

TGAAGAACAATTTTTATTTATTTATTTATTTTTAAAATTGAGTCAATATA

GTTAAAGTTTCATGAGGTAACAGAGAAGCTGTTACAAAATTATTAAAAGG

TCATTAAAGTTAGCTTAGCCAAAATTTGAAAAGGTTTCTTGGAAATATAT

GGAAAGAAAAATAATATGCTATTTTTCTACGAGAGGGACTACCTTTGAAT

GGAAGAGCCACATTGACTTCAAGAAATGCCTTAGAGATCAGTGGAGGTGA

CCCACCACCCCACTATTTTTTGATTAGTGAAATTTTTTTTTTTTTTATTT

ATTTTTTTTTTTTTTTCTTTTTTTCTTTTTCTAACCTTGCCTCCCAGCCC

CCACGTGGGGTTCTGCCCCGCTGGGGTCTCCAGCAAGGTCCTATTCCTAA

ACCCACTAATAATTTCTCTCCTCTCACACATAAATAAATATCCACACGTC

ATATCAAAAGCGACTCATGACAGGTCCATCTTACCTGTCCAAAAATAATT

CTTTAATTATTTTTGCGATGGCTCATTCATGAGATCTCGGCAATTGAGCC

CACCCCTGATTGATGAGTGATCAGCCCATTCGTACTAAGCTGTTTTTTGT

GCTTGACAAAGCGGCCTACATTTTATCATTATCTACTGTCCTGCCACATT

CAATTAAAATAAAAAAGACTGGTAGTTGTGTTTGAAGTGACATTAATAAT

TCCTATTTAAATTAACTTAATAATTTTTTAATTATTTAGTTAAAATATAA

ATTAAAAAAAATTAGTATGTTTAAAATAATTTTTTAACTTTTTACGAAAA

GATGTGCAAAAAGTAAAGAGAAAGGAAAATCTTTGACTTTTTGATATAAA

TTTTTGATTTTTTGATTTTTTTAATAACTTTATGACTTTTAAGCTAAAAG

AGACCGTTCCCAAACGAGGGCTTAATAACGTTAATTTCACATTTTTATTA

TTATTTATATTTACAGAATTTTTAAAATTATAAATAAAATTTTATTTGAT

TTATGTTTTAAGTTTTAAAAGGTTGTAAACAATTTCTAAAATTTATTAAA

TTTTATAAATACAAAAAAATTGATTTTAATTTTAATTTATTTTTTTTTCA

TTTCTTCTTTCTTGTAATCCTTTCTTTTACTCTTTTGAATCACAGACCAT

TGTTCATTATTACTAGGAGTATAAAAATGGCCTGCGGCTTGAAATACACC

CTCGATCTAGGATAAGCCTCCTGTTTAGGCCCGTTTGAGCGGGGCCCCGA

GCCCAGCCTTAGACCCTAGACCTGTGCCATCATTTTGAAGTCCGACCCAT

CGGCCCCCTTAAGGCCCACTCAAGGCCCGGTCCGAGGTCCGGCCTACCGA

GGCCCAGTGTTTTTCTAGTGGAAAATAATAAAATTTTGGTAGAAAAAAAT

AAAACAAAAAATAAAAAGATTTGTTCAAGCCTGCCCAAGCCCGAGCTCAA

GCCTAGAACCACCGGCCCGCCCTGAGACCCGGATACCCCTCGGGCCTAGG

CCTAGGCCTAATTTTCTGGTCCGTGGAACGGCCCGACTCGGCTTGAATTT

TGATAAGAACCCACAAGACCTGACATAGGTCAAGCCAGGCCAGGCCAGGC

CAAATTTACACCTCTAATCATTATTGGCTATCATTGCTAATTACATCTTG

CACAAACCATCTCTAACCAGTATCTCCTCAATTGCCAACCACCTTGTTTG

GAATGTGGGTTGACTTTTTAATTTTTTGACTTTTTGGCTATTTTAGTTAG

GGGCTAAAAGCTAAAATAGCTTCTTTTAGGATAGACTTTTTGATTTTTTT

GACTTTTGTAAGAGAAAAAATAAAATGATTATCGGTTGGATATGACATGG

GGGTGTTTAGGAAGAAAAAAAATAGCCTAGTTTAGACTAAAAATCACAAA

AAGACAAAATATCACATCAAACCCATAATTATTTCACTTTTTCTCTCACA

AACATCACAAAAAAATCCAATCATTTTTTCAAACACAAAAATTTTAGCCT

ACTAAAATAGCCAAAAACTAAAATAGTCAACTAAAATAGCCACTTCCCAA

ACAAGGCCTTTGTCTTATTTGGCTAAAATAACATATTTTGTCCTATATGA

CTATTTTAGCCCCAACCTTTATTCTAAATAGGGCCTATATGTCATAGCCA

CCAACCACCTCTCACCAATAGTAACCACGTTTTGTTAAAAAACACCGACA

ACTACCATCCTAGGAGATCGACTAGCCCTTATCTTTGTCCATAGACAATC

ACTTTCGACCACTACTCCCCGTGGCCATTAACTACCTTTGGTTATTGCCA

ACCACCTGACACCAATAATAACCACTTTTGGCCACTACCTTTCGTGACTA

ACAATCACATTCGATCACTACTCCAATAATTGACAACCACCTATCGCAAT

TACTCATTTTCACTTGTTTTTAAAATAATTTTTTTTAAAAAGTTGCGTAC

CAAAAACTTTTTTTGTTTTCATTTTTATCCAAAATAAACATAAACATATT

TAATATTTTCTGTAAACAAAAGATAGTAAACTGTAAACATTGTCAAAATA

CCTAAAATGTGCCAATCAATTAATTCTGGAGACACCATTTAAATATTTCT

AATACTCTATTTACGATGACTGTCTATTTTATAAAACGCAATTGTCGTTT

ATTTTTTGGAAATAAATATGATTAAAAAAATTATAATTATTAGATCAATT

TATTTTTTTTCATGAATGATATACACAGAAAAAAATTTAAATTTTTTTTT

CAAATCAACAGAATCCAAGGTAAACTTTGCCACTCGAACAAACCGAAAAG

AAGCCCAACCCTTTTAGTTTCTATTTGCAAGAATAATAGACACCATCATT

ATGAGTAGAAAGTAAAAAATAAACAATTGAGCATGTTTGGTAATTTTTGT

TCAGTTGTAATCATAATAAATAAATAAATATGTTAGTTTCACTTTTTAAA

ATTTGTACAATCTAAACCTATCAATTATAAGTAAACTAATTGTAATTTCT

CCATTTTCACTGTTTAAGTTAGAATCCAAATTTAACAAATCAACATTTGA

CATTATTATACTTTAATGTAACCACCACTTCGAAATCATAATTAGCACGA

CTTTAATTCATAGTCACGACCACTTTAGAAGAAAAAACAAAACAACACAA

AACAACACAAACATATGATCCACTTTTAAGATGTGTGTGGACAATAGAAC

TACCCTTCATAATCCCAACACGCTCGCGTGCATGCACAAACAGAGAGACA

GAGAGGTTTTTCTTTTCTTTTTTTTTTTTTTTTTTTGATGGGAAGAGATA

GAGAGAGATTGAAAAGTAACTTTCAGATCTTATTTTTCCAAATAATTTTT

CAACATAACTTTTGTCACTTAAATTCAGATGATGAAATTAACATGTAAAT

ATGCAGGGGAGTGACAGAGGAATACAATGTGGAAATAATGAGAACAACCA

ACAAGTTATTTGAACTTCTCTCAGAGGGACTAGGTTTGGATGGGAAGGTT

TTGAATTCTTCTTTGGGTGGTGATGAAATTGAATTTGAAATGAAAATCAA

CATGTACCCACCATGCCCACAACCTCAGCTCGCCCTCGGAGTTGAACCTC

ACACTGACATGTCTGCTCTCACTTTACTTGTCCCCAATGACGTTCCCGGT

CTTCAAGTTTGGAAAGACGGTAATTGGGTAGCTGTCAATTACTTGCCAAA

TGCACTCTTCGTCCATGTTGGTGATCAACTTGAGGTAAAAATACTCTTCT

CTTGCTCACCTAGAAATATCAAACAAATGAACAAAAAGTCTAGTAAAAGG

AAAAAAAAAAAAAAAACACCATACTTGGAGAAAATTGCATTGAATATGAC

AGTAATGATTCAAATTTAGCATATATATGTGAAAACATAGAGTTCTTCAA

GCAATGGGTCTATTTGACAGTTTTTTGTAAGTTGAAAGCTAATAATTAGA

AACAGAAAGCCAAAAGCACTCATTCGTGTACTTCTCAACAATCAGTTATT

TATTTTATTTTACATTTTATAAATATTTTTGCCTCATAAGTTAAAAGCAG

AAACTGCATCAAACATTGGCACAAATTTTCAAAAGAAAAATGCTGTAAAC

ATCTGCTGCACTAGACAGGGCCAATATCTAGCCTGACTTTAGTGAAAAAA

TTCAAGATTCAGAAAGCGTTTCAGTGCTGCAACTTACAAAACATTTTGTT

TCAGGTACTAAGCAATGGTAAGTACAAGAGTGTTCTTCACAGGAGTTTGG

TGAACAAAGAAAGGACAAGAATGTCTTGGGCTGTGTTTGTCGTGCCTCCT

CATGAAGCAGTGATTGGACCTCTTCCAGAGCTCATTGATGAGAAAAACCC

AGCAAAATATTCAACCAAAACATATGCTGAGTACCGTTATCGCAAATTCA

ATAAGATTCCACAATAA

>TEA028194.1 locus=Scaffold793:1051608:1055662:- glutamine synthetase leaf isozyme, chloroplastic

ATGGCACAGATTTTGGCTCCTTCCCCACAATGGCAGATGAGAGTTACGAA

GAACTCAACAAATGCGAGTCCAATGACATCAACAATGTGGAGTTCTCTAT

TTTTGAAACAGAACAAGAAAGCAACAGCTAAAACCTTTGCCAAATTTAGG

GTGTTTGCTCTACAGTCTGAAAACAGCACAATAAACAGGATGGAGGATCT

GCTAAGCATGGATGTAACACCATACACTGATAAGATCATTGCTGAGTATA

TATGGTACATTTCCTTTGCTGAACGTCCCATTCTCATAAGCAGGAAACAT

CCTCATAGTAGCAATTTTTGGTTCACTAATCCTATAAATCAACTGGGAAT

TGTTTTTTCAGTTATGCCACTATTTATATTTATTGTTGGTTATACAGTTA

ACTATCTTGACAATCTATGCTATTGCCTTTTGAAACAGGATTGGAGGATC

CGGTATTGATCTCCGTAGTAAATCAAAGGTATTAATATGTTTGATGCTGT

TAATATTATCTACATTGAATTTTAGTTTTTTTTTTTTTAACGAATCTTTG

AAATTCAGGTAACTTTTTTTCGAAGTCTTATGATCTTGTATTTGCAGACA

ATCTCGAAGCCAGTTAAACACCCATCTGAGCTTCCAAAGTGGAACTATGA

TGGATCAAGTACTGGACAAGCACCAGGAGAAGATAGTGAAGTAATTTTAT

AGTAAGATTTTATGAGCTTGAACTATTTTTTTTTGTATGTCTTTGCATTT

TCATTTTCTTTCTTTTTTGTTTTTGCCCACTATTACCTTGATTATTTTGG

TTTTGATTCTTCAGCCCTCAAGCAATATTCAAGGACCCTTTTCGTGGAGG

TAACAACATCCTGGTGAGTGTTGTAAGATAATTCAAGAAATTCATTATCG

ATGAATATATACTTGTATGATTTGCAAACAATAATTCTTTGTACAATTAT

ATTTCAATCAAAAGATGAAGCATATTAATGTAAAGACTAGCTCTTATATT

TGATGTGATTTAAAAAGGTGATTTGTGACACTTACACGCCACAAGGCGAG

CCGATCCCCACAAATAAACGCTACAAGGCTGCTGAAATTTTCAGTAACAA

GAAGGTTGTGGATGAAATACCATGGTAAGTTTGTCTGGATTGGCATTTCG

CCAAAATTATTCCATTAGACAAGCATGGATTTTGTTGAAAATGAAAATGA

AAAATAGTCCAGTTTTTTATTGTGTGAAAACAAATAAATCATGAAAAACA

TTTTCGATGTTTCTTTGGTTCTCTTTGAAGCAGAATCGCTAATATTTTTC

ATAATCAAATCAATCCAACCTAGGTATGGCATAGAGCAAGAGTACACCTT

ACTCCAAACAAACGTGAAATGGCCCTTGGGTTGGCCCGTTGGAGGCTATC

CTGGTCCTCAGGTAATTACATTCTTTATCAGTGAAAATAATTTTGCTACT

CTGATCATCTCAATTTATGCGTACTAAATTGATAGATATTGAAATTTTCG

TGGCCTAATAGGATTATATCACTTATTTGAATTTAGGGTCCTTATTATTG

TGCTGCCGGAGCGGATAAGTCATTTGGGCGTGACATATCAGACGCTCATT

ATAAGGCTTGCTTGTATGCCGGAATTAACATTAGTGGCACCAACGGAGAA

GTCATGCCTGGCCAGGTTTTCTTCTCTTCCTTTGCTTCTCTTTATCTCAT

GTATGAGAAGGAATATCACTCGCACAATGCATTCCTAGATTATTTTTTCT

AATTTGATGATTTCCCCCCCGGTAGTGGGAATTTCAAGTAGGTCCAAGTG

TGGGAATTGAAGCTGGAGATCACATGTGGTGCGCTAGATACATCCTTGAG

GTAATTTGTCTTCATTTGTTACTGGGTTTGTAGATAAATCAATTTAAAGT

AATAATCTGTTGGCGGACAAAATGAAGTCCATTAAATGGTAGTTGGGTTA

TTCAGTTAGCTACAAGAACATGTCTTAGGGTCTCACCCAAACATTAAGGT

TGTATATTAGGATCTTAGATTCGTGGATAGAAAAGAAAAGGGAAGGAAAA

AGTGAATGATATGAAAGTTAAATTTTTTCAATTTCCCTTTACCTCCACAA

ATCCCTCAGCAAATCTAGGACCCAAACACAGCATATGAGGTCCCTTTCAA

CTCACCCTTGAAGTTAGGTGGCAATCTAGCTATGCTTGTTGAATTAACTA

GTTCATTAGGTAAGTGCTTGAACTTAAATAAATAGAACACGCCGATGGTT

TTTTAGGGTATCATCTTCCACTTAATTTATAACGGTTTATGTGTGTTTTG

CCATGTACTATATGGCTATGCTTGATTTGCGGATTGGTTTAGGGGAGGAT

TGGATATCATGGGATTATAGGCAGGATTAAAGGTGGTAAGGACAATGGAT

ACATAGCATAAGATTAGTTGTACTAAATTTAGTTAGGATGGGATAAGGGT

ATGTTACTCTTGGATTTTGACCCGAAATTGTTTTTATTTTTGGAAAACTA

GTTGAATAGGTAATCAATATGGGAAAAGATTATTTCTGAATGTTGGATGG

ATGGAGGGACATAGTCCCATGTCTTGAAATTAGTTATCCCCCCCAATTAA

TCCCCTGATTACCAAGCTTGAGGGCTCAATCCCGGGACCCAAAAAAAAAA

AAAAAAGAAGAAAAGAAACAAACAATGGACTAATATTTTTAATCCCAATC

TCCCCACTCTGTCAAACAAACAAGCCTGCATATCTAACACAGAATAACTT

GGTTTTTCTTTGTTGGTTGTCACAGAGAATTACTGAACAAGCTGGTGTTG

TTCTCTCACTCGATCCGAAACCAATAGAGGTAATCTAGATACCATTTGTA

TCAAACTTTTCTACCATAAAATGAATAATTATTGAATGACTGTCTTCGTC

GTTGAGTCAATAGGGTGACTGGAACGGTGCAGGATGCCACACCAATTACA

GGTACTAAACTACTTTTATGATAATTAGTTTGTTATGTACCTGAAAAACA

ATGTTGATATTTTGGATCAATATTTAGTAATTGCTTAAATAATCGTCGGT

TTGACAGTACAAAGAGTATGAGAGAGGATGGCGGGTTTGAAGTAATTAAG

AAAGCAATTCTGAATCTTTCGCTTCGTCACAAAGAACACATCAGTGCTTA

TGGAGAAGGAAATGAAAGAAGGTTGACGGGAAAGCATGAAACTGCCAGCA

TCAACACATTCTCTTGGGTATGCTCCAAAACTATTTGAGACTCTTATTTT

GGGAAAAAAGTTTTAAAAAATAAAATAAAATTCAAGGTTGGGTATGGAAG

AGTTTTTAAAAACAGTTTTCTAACTCAAAATCATGTAAAACAGAAATTAA

GATTGAATTTTCATCTGGTTTGTTTTTGTTTGTTGTTTCTGAAAACTGTT

TCAAAGGGCTAAGTTAAAAAGAACCAATTTTCACACCAGAATATTGTTTA

TTTATTTGTTTTTTATTTCTCCTTTTGTCTTGTAGTTAGGAAAACTAAAA

AGACAAAGCATTGTGTTCTTATAATCTTTACAGCTTTCAGAAACATTATT

TTCTGAAAATTGTTTAGAAAAAATGTTTCTTCCTAGTTTTCTAACTAAAT

CACTTGCCCTCTATTTGCAGGGAGTCGCTAATCGCGGTTGTTCAATCCGC

GTGGGGCGTGAAACTGAGAAGCAAGGCAAAGGTATGCTAAAGCTATATTC

ATGAATTTTCTCTATTTGATTCGAGGAAAACTAGAAAATGGTTTTGCAAA

CTTTTACCATTTTTACAACAGATTTCACAAATCCCACATTTATAACTGAC

TCGCTCAACGCCATTGATATGGTTCTTCATTAGATCATTTTAAACTGACC

ACAGTAAGCATTGCCTATGTTAATTTTCTGGTTAATGTTGTCCCTCATTT

TTCCTTCCTTGTTAGGTTACTTGGAAGATAGGCGTCCGGCTTCAAACATG

GACCCATATATTGTGACGGCCTTATTGGCTGAAACTACCTTACTGTGGGA

GCCTACGCTCGAGGCTGAAGCTCTTGCTGCTCAGAAACTGGCAATGAATG

TCTAA

>TEA003869.1 locus=Scaffold854:422845:427376:+ aquaporin PIP2-7-like

CCTCTCTCTCTCCCCTCTCCATAAATACCTACCCCTCTCTTCCTCACTTT

CCACATCCTACACTCTCTCTCTCTCTCTCTCTCTCTCTCTACACACACAG

GCTCTTTTGTTTTGAGGTTAGAATGGCAAAGGAAGTTGAGGAAACAGTAG

TAACAGAGCAGGAGTTCTCAGCCAAGGACTACCACGACCCACCTCCAGCT

CCGCTAGTGGACGTGGTGGAGCTGACAAAATGGTCCTTCTACAGAGCTAT

CATTGCAGAGTTCATAGCCACACTTCTCTTCCTCTACATCACCGTCCTCA

CCGTCATCGGCTACAAGAGCCAGACTGACCCCATCAAGAACGCTGACCAG

TGTGGCGGTGTCGGCATCCTCGGCATCGCATGGGCCTTCGGCGGCATGAT

CTTCGTCCTTGTTTACTGCACCGCCGGTATTTCTGGTTAGTATAACCTAA

CTCTTACTATAATATTTTAGAAGCCGCCTGTAACACACGTCCTTTCTAAA

ACCATTATTTACCTTTTTACTGATTTAAACCCTAATAATTATACATGTTT

TTTTTAGTTAAGATTGTCACTGACCTTTGTAAACAACAATTATTCTACTT

GCACAAAAATATATGGCTGAATTTATGCGGGATTAATAAAAAAATAAGAT

TCATCTCATGTAAAAGGGTTTTGTCTAATTTTGTAATCAATTTTTATGTT

TCTAATTATTTAGTTTGGGAAAAAATTGAATTGTCAAATATTTTGATGTG

GACTCAAAATAAGATGACTCATCCTACTTATGTTTTTTTTTAAAATTTGA

ATCATTTATAATCATATCATCTCGCCTAATTATATTATATCGTCATTTAT

ATTTATCGTTTATTTATTATTTTACATTTTTTTTAAATGTGTCTTAAGAA

TACATATTAATAGAAAAAATTTACAAAACAAAAATTATGCAATCATACAA

TTACACAATTACAATTATTGAATTAACTTTTTCTTATAAATGATCTATTA

AAAAAACTTTAAAAAATAAAATTATCAAAATAGGTCCAATAAGTAGCACG

CATGCGATAAAGAATTGGGCAATCGAGTCTTTTGCAAGAAATACTAGTAG

TAAGTTTACAAAATAAGAAAAGTATTTTATAAAAAAACAATGATAAAGAG

TTCTGATTTTGAAATTTAAAAAAAAAATTTTAGTGATATGGTAAGAGAAA

AAATATATATATATAGACACACACATTACATCATATGTGGCTTCACTCCT

TACTACATCACTAGAGTTTTTTTTAGAGTTTCAAAAATTAGATCTCTCAA

TTTTTATTCTTAAACTTATAATTATTCCTTCAACAAGGATAGAGACTCCC

TTTTTGGAGAACTTCCATAACTCCCAAAGTGATCCACATCACTCATAATG

TGAGCCCCACACATCATGAGTGGTATGGATCACTTTGAGAATTGTGAGGG

TCTTCCAAATATGAGGTCTTACTCATTTTCCTTATTCCTTTTACAATATA

TGAAATAATAATGATAGAGACGTTTTTTTTTAAAATCCCTACAATTCTTT

TTGAATCTCAACCATTAATAGTGTGTGTCCACACATACACCAATAATTGA

GATCGAAAGAGGATTGTGGAGTCAAAAAAGAATTGGAGTCCAAGCATTTT

TCTATATGAAAAGTTGGTAATCAGCGGATGTTTTTCTGACGTGCGCAGGA

GGGCACATAAACCCAGCAGTAACGTTCGGGCTCTTCTTGGCACGTAAGGT

GTCACTCATCCGAGCGTTGTTGTACATGGTAGCACAGTGCTCGGGTGCTA

TCTGCGGTGTTGGGTTGGTGAAGGCTTTCCAGAGAGCATACTACGATAGG

TACGGTGGTGGGGCCAACGAGGTCGCACCAGGATACAACAAGGGGACCGC

TTTGGGTGCAGAGATCATCGGGACCTTTGTTTTGGTATACACTGTGTTCT

CAGCCACTGACCCTAAGAGGAGCGCACGAGACTCCCATGTTCCTGTAAGC

ACCTTATAATTATCATACTACTAGTTGGTTTAAATATGTAGGAACAAAAA

AAGCCTCATAAAACTGTTTTGACAAATATTTTTTTAAATTTTTTTTAGGG

CCTTCAGATTAAAAAGGAAAAATCAAATCTTAAAAAAAAAATTAAAAAGG

AAAAGTCAGAAAATGAATTTTCTGGGAAATTAGTTGTGTGGCAAAGAGTA

AAATTTATATAGTGTGAAATCTATATGTAGAATAAATTTAAATTATAATA

TCATAATTCTGAAAAATTGTTAAGTAGTTAAGTTTGAATTTGTAATATTA

TTAAATTAAAAAATATGTAAAAATTATAAAAAAAAAATTAAAAGTACCAT

GGCCCATTTTGACAACTTTGACTTTTTAGTTTTTTTTTTTTTTGACTTTT

TTGAGTTTTATCATCTCTCTATTTTTTTATATGTTTTTTTAAAAAAAAAT

AAAAAAATAAAAAAAATTAACCTTTAACCTACTCTCCAAAACATCCCAAC

AGAAACTAAAGTGTTTTGAGAAGATAGTTAAAAGTCGACTTTTGACTTTT

AATCAAGTTTTTTGACTTTTATAGAAAGTCATTTGGAAAGTTGATAAAAA

ACTATTTTTTTACTTTTTTTGTTAATATTTGTTTATACGCCACGTCACAT

TTAACTGATCAAAATACTTTTACTCAAAGGATTTTGCCCTAGCCCACAAA

TGTAAATATGAAAGGTATAATTGTAATTTCACTAGTAACTTTTAATTACC

TTTCCAAATAATTTTGATGCAAAAAAAGTCAACTTTTTATTTTTTTTTGA

CAAGCTGCAAAACACATCTTTTATTTTTCTTTCCAAATGGCCCACTATGA

AAAATATGATTTTAACATTTGCCAAACTGATGAAATGCTCTAGCTCACCG

AAAACAACATTTCACTAGCTCATTGACTATAATAATAATAATTTAAATAA

AATAAAAAAAATTCATTTCCTAAAGACCTATGTATGATTTTGGCTACTTT

GGCTATTTTAAATTTTTTGACTTTTTCAAATCACATAATTTCTTTATTTT

TCTATACGTTTTTTAAAAAAAAAAAAAAAAAAAAGTTAAAAAATTAACTT

TTAACCTATTTTCTAAAACAATCCATATATATTGGTGAATCAATTTACTT

AGAATTTTTATTTGTTAGGTAGTTATATAGTGAGTTTCAATATTTTTAAA

AAGAAATACTATATATTATTAACATTTTTTGTCTCCATTTTATATCACAT

ACTACATAGATACTTATAATTAGTTTACTTATTTGGGGATGATTAATAAT

AAAAAAAAATCTTATCAAGTAATCCGGTAATATAAAAAGATTTTTTTTTA

AAAAAAACAAATAATATGTAATAATCATTACATTATTTTTAAATATAACA

ACCGTGACACACACGACTTTCATTACATAACTATACATAAAAAGTAAAAC

AAGATGGCAAAAGAAATTCAATGATAAATAACTTCTTTTTTTTCTAATGC

TCATCACATCACACTCCTATAAAAAAGATTGTAATTTTTTTTTTATTAAT

TAGAGAGAGGTGTATGTTTGTCTCGTACCCAAATTGTCCTAACAAAATAA

TGATAAAAAATGGATGATATATTAGATTTTTATTGTACTTGTGAATCTTT

ACAAAATGTACATAATAATTGCAACAATGTTAGGTGGCCCGAAAAATCAT

GACTCGAATGGCTCAAAACACCTAAAACGGCGTCGTTTTGACACATTCGA

GCAACAAAAATTCGAGTCACCAAACATTTTCCATATAATTAAGCATATAA

ATAACTGAGTAGTAATGTTGATGATGAAGGTGTTGGCCCCACTCCCCATT

GGATTCGCGGTATTCATGGTCCACCTAGCCACCATCCCAGTCACTGGTAC

CGGTATCAACCCAGCTAGAAGTTTCGGAGCTGCTGTCATTTACAACCAGA

AAAAGGCCTGGGATGATCATGTACATCCCCTTATACACTTTCACTTTTTT

TTTCTTTTTTCTTTTTTTTCTTTGATAATTGGATTTGATTAATCATAAAC

ATATACAAATGTGTGTGTATGTAAATTTGTTTTTTTTTTTTTTTGGCAGT

GGATATTCTGGGTCGGTCCATTCGTTGGGGCAGCCATTGCTGCATTCTAC

CACCAATTCATTCTGAGAGCAGCAGCTATTAAAGCTCTGGGATCCTTCAG

AAGCAATGCCTGAGGAGTTTGAATAACAATGGAAGGACAAGAGAAGGTTC

CTTTGAGCAAGACAAAAGTGATTTGATAATGAAGAGAAAAAAGGGTCTTG

GAATTGTAGATAATATTTCCTTCTGGTGTCTTTTGTATTTCCTGTTTGTT

TCAACTTTGTCTTTATCTATCTCCTTTCTTGATTTTCTTGTCATCATCAT

CATCATCATCATCATCATCCAATTTTCTTTTTTCTTTTACCCCTTTTTAT

CTTTTTATATTTTCTTAGAGTGTTGTGTATTCATGATGTTTCCCCTTAAA

TAAATAATCAATTAGTAAATGGGTTGTACCTT

>TEA023090.1 locus=Scaffold947:529897:538441:- Alanine transaminase

GGTTACAAAAGTCAAAATCCATGGGCGTTGAAGCCAAACAGAGTTGGTAA

GAGACTGACTGACTGACTCACGCGCATGCCCACGGGAATTTTTCTGAGAA

ACAAATGTTGAGGGCGGCTTCGTAAATGGAATCGCCCAAAACCAAACCCA

AATCACATCCATTAAATTGCAGGTAGAAAAAAAGTGCAAAATGTGGAAAT

TCGTAGCCGACAAAGCCAAACATCTCATCAACAAAATCCCCACCGCCTCT

TCTCCTATTTTCACTCTTCGTCTCTCTCCTCTTTCTTCTTCTCCAAAATC

TCAATCTGCTATTCGTTTCTTGACCTCTCTACACTCTCCTTCTTCTGATT

CAATGGCTTCTTCCGATGCATCTCTTCTTGTCACTCTTCAATCCATCAAT

CCCAGGGTATCACCCACTTCCGATACATTTCTATACATACATGCTTTCAA

TTTTTTTTTTTTAGTTCCACACTAGTTTTCACTGTTTCATCGTTTCTATT

CATAGCTACTACTCTCTCTCTCTCTGTCTCTCTCTCTCTCTTTAAATTTT

ATTTAATCGTGTTCTAGGGTTTATTCTAACTGCGCACACACTCATTTCTA

TTTAGATCATCAATTTTTTTTTTTAGTTCCTCACTACTTTTCAATGTCTC

ATCGGTTCTGTGCATAAGTGATTTCTCTTTAAATCTAATCGTGTTCTAGG

GTTTATCTTCATCGCGCGCCGCGCGCGCGCGCGCGCACACACACACATAT

ATATATATTGTATATTATCGGTGGGTGCTATGCTAACCCATCACGTTTGA

AGTATCATATTTAAATTTTTGGATGGTGTAATGATTGAGGAACGGTTTGA

TGAATGAGAAAAACCATTTAGCAATTTTGGGTAATTTTATGATATTCGTT

CGAGCAATGTTAGGAACACAATCTATGTCTCATTTTGTGTTGGTCCTCTT

TGCACTTAGATTTTGTATTCATTCAGCAGTTATTATGTTGATATGAAGGA

TATTTTTGGAAAAAGAATAAAAAAAAGAAAAAGTATAGTGTTCCCTCCAA

TTGGAGAGTACCCTTTATATAATGGAATAGATATACTAGAAGACTAAGAA

CATGGTGCAATTGAAACTGTCAATGCATGGAGATTGTGTATGAGTGGAGA

GAGGGTTCGTGTGTTGTGGGCAGTAGTTTTTGATTTGTTGATTTTTGTTT

TTGGTATTTACGTTAATTGGTAGGCAAAGGTGAATATTTGGAGAACATAG

AGTGTCGATGTGATTTTTTTTGGGATTCAAAATTGTGTGACAAAGCTTTT

CTGAAGTGAGTTGTGTTGTATGCTTATGGATAATAGAATAGATAGATGGA

TAGAATGGATATTGAATGTTATGTTTTATTGTGTTTTTCCACCCCTGATT

TTGTAAATTTGTTTATGCATCAATATGTATTTTCTTATGTCATGTCATTG

GGGAAACCCAAATCGCTCTCTCTCAAATAAGCTTTATATTTTTGCTTGTT

ATGTATGCTTAATTTGTATCCATCTTTAGTTGGATATATTATTTTATTTT

TTCCTGTTCTGTTGCTTTGGGTATTGGCTTCTGTTTTTGTGGTTGTTTGT

GTTGTGTTTAATACTTTGGCATGTAGGTTCTCAACTTTTTTGCTTTGCTA

TATTCTATGTTTTCTAGATCGAGCATTTGATGGATTTAGACATCTGAGTA

TTTCGATTATCACCACTACTGTCTTAAACATATCAATGTTGCTATATAGC

TCATGATTTGCTTCAACTGCTAAATAAGATCAGGTGGCTGTATTGCAGAA

CCCCTGCTGAATTCAATTGAGTAGGTGATTTATATGGAGATAGTGATGGA

CCCATGAACCCGTTTGTGTCATAGAACTAAAATTACTCTTCCTCTAGCAA

TTCAAAAATTGGAAAAGTTCAACTTCTCGTTTGAGAACGGGCTATTTTAG

TAATTTTTTAGCTTTTTATAAAAAGATGTGCAGAAAAGTAGAGAGAGTGA

TGTAACAACTTATAGTGACTTTTTGGCTAAAATATGTAGTTCCCAAGTGA

TAGAGTCAATAATTTGTTGAATTAAATTTAAGTCGTGTTAATAGTCATAA

CTACAATTTTTTGGTTTTTGATAATTTTTTGGAAACTCAACCATAATAAT

AATGCCTTAAACATCCTAGTGTATATTTTTAAGCTTGATGAATAAGAAAT

GAGATATCTGCTTGTCTTTGCATTTTTCACTTGTAGATTTTCTTCTGGAT

TTACATGTTGGTCCTCATTTTAACCTCCAAGTTTTCTATTCTGCCAGGTT

CTGAAATGTGAGTATGCTGTCCGTGGTGAAATTGTTACCCTGGCCCAGGT

AACATTGAGATTTGTGCTATGCTCTTCGATGAGATATTAATTAAAATTTC

AACATTTGATAGAGAGATGCATAGCATTGCAATGGTAGAATGGCTATCCA

AAAAAAAAAAAAACCTGAATTTTGTGTTAAACTGAGGAAAATTTATTTTA

ATCAAGAAGAATGGAATGTGAGGTGAAAGGAAGAAAACAGACTAATTCTT

GAAGATGTGTCACGATATTCCCTGCCAAATTCATGGAATTTAGGCCTATT

TATGCCCATTGCATACTTTGCAGTTGCTCTATGAGCATTACCTAAACTAA

AGTATGCAAAATTATTTTTTGTGGATTGTAATTCTATTTGATGACTGACA

TGTTCTCTCACAGAGCTTGGCCCCTTGAAGAATTATCATGAAAGTAAATA

AACTTGTAAATTGGCAATTTGGTTAAGCAAGATAACTATTTGAATGTGCT

GGTTCTAATTCTTTTAATTTCTACAATCTACTTATCTCTTTGAGAATTGT

TTGTGTTTTTTTTTTTTGTTGCAGCAATTGCAGCATGAATTACAAACAAG

TCCAGGTGCTCATCCGTTTGATGAGGTACCTTTTATTTGTTTATTTCTTA

ATATATTGTACTTGCATATATTTTTCCTTACAAATGCATACTGCAAATGC

ATTTTAGGCCTACTTAAAATGCATCTTGGTATTCATCTAGGTGTTGTTAT

GTTTCTTGACTCAATTCACTAATGACTCGGTGACCATAATCCTTTGTGAA

CTGTTTTATGACCTCTTTTCATGTAACTAATTGAAAAACCTAATTTCAAT

GTTACACACAAGATAAAACATTGAATGAACCTAGGATTCCATTCCATCAA

CCTCACTTTGACTAATTCAGAGATTTTGCTGATTGGATTTTATTGCTCAC

TTCTTTAAATAATGTTTCTGTGTGAAAAGTAAAACAATGAATCTCTTTGT

AGAAAGCTTCTTCTCTTATTTAGTGTTGCTACCTCATGCAAGAATTACAA

GTCTTCAAAGCACGATTTACGAAGAGGTTTTCTGCTTCTGCACTTATTTT

CCCCTTATTGTGACAGATACTTTACTGCAACATTGGAAATCCTCAATCTC

TTGGCCAGCAGCCAATTACTTTTTTCCGGGAGGTTTGTTTGCTGTAACTT

TGTCCTATAAAGTATTATATCAACGTCAGCTCAAGCAATATTTGATCATT

ACCATCCTTATGTTAGGTTCTTGCACTGTGTGACCATCCAGCGATATTGG

ACAAAAGTGAAACACAGGGTTTATTTAGGTATGGAAACTTATACTTGGTT

ATGTGCTTTGTACATTGTTGATTTTGATTCACACTGCTGGAAAATTTTGC

TGTTTTCGCAGTTAAAATCTCATCTAACTCATAAACACATGCAATTCCAT

CTCCAATTGAGACCTCTTGACATCAGTTTGCTAGTGGTGTAACTTGTATA

TTACCTCTGCTGGATTTCCTTGTCCCTTGTTTGGATATTTGCCTGCTAAT

TGATTGTTAGTGCTAAGTATCTCACATAATTTTCTAAGGTTTATGTAACC

CTAGGACTGCAGTAGTTCTGAAAATTTTAAAAATGAACTCATAATGTGGT

GAACTTCTAATTGTTTGAAATTTAAAGTAAATGGGAATTTGAAATGTCAA

TTTAAGTTTCCTATGGAGATATTGATTAATCTTGCCATTTATGTTTCTCC

ACCCATAACATAAATAAAAATTCTGTAGTGTTCTCTTTTGAGCGGATAGT

ATTCAGTATTCAGTGGAGGCTTCTATACCCAACAGCAGGGAGTTCAACAC

ACCATGGTTTCTCAAAAAATTAGTGATGTAATGATGTATTTAAAAAGTGC

CAAGGAGAGAGAAAAGGAGGAAAATTGAGTCATTCAGGAACAAAGAGCAT

CCTATGTATATCACCTAAGCCTTGCAGTGTAGTGAAACTGGTTTTGGATT

CTGTTGATAATCAGTTAAGTTCTTATTCCTTTTCTTCCTTTTGCTAGTGC

GGATTCCATAGAGCGAGCTTGGCAGATCCTGGAGCAAATTCCTGGGAGGG

CAACTGGTGCATATAGTCACAGCCAGGTTACCATGTCCTCTCCTGTGAAC

ATGGAAAATTTGAGCTAGTACATTGCTCGTGTATTTATGTTGTATGCAAT

GTTTTGTGTATACATGTATATTCACAGGGAATCAAGGGTTTAAGGGATAC

AATTGCTGCTGGCATTGAAGAACGAGATGGTTTTCCAGCTGATCCTAATG

ATATTTTCTTGACAGATGGTGCAAGTCCAGCGGTAAACTTTAATGTCACT

CCCTTCTGTCTGCATACTTCATAACTTTTCAGAACTGGCGGCACTCGAAT

CTCAAGAATGGGTAACAGATTTTTACTCTCATGTCAGGTCCATATGATGA

TGCAGTTACTGATAAAATCGGAGGAGGATGGGATTCTATGTCCCATTCCT

CAGTACCCTCTGTACTCTGCTTCAATTGCCCTCCATGGTGGCACTCTTGT

ATGTTCATGCAATATAAAACCAAAATTTAGGTCAAATAAGTCGTTGTCTT

GTGGGTTTTCATTGCCCATGAGAGTATTTGACTTTGAATTATAAGCTGTT

TTTTTGCAGGTTCCTTACTATCTTGATGAAGCAACAGGGTGGGGATTGGA

GATTTCTGAGGTTAAGAAACAATTGGAAGCTGCCAAGTCCAAGGGCATCA

CTGTTAGGGCCCTGGTTGTAATAAATCCAGGCAATCCAACAGGACAGGTA

AAGCAACAGTCAGATTCTGGAAGATTAAGTAAAATCACAAATATAACTTG

CATTTTAATTCAATGATCTGAGGGTGATTGGATGGGGGCAATTATTGGGT

GTTATGAGTTATGAGTCCTGTGACCTTGTCATGTTTCATAGTCTTGGTAA

TCTGTACCTACAGGTTCTTGCTGAGGACAACCAGCGCGACATTGCAAAAT

TCTGCAAGGAAGAAGGTCTTGTTCTTCTGGCAGATGAAGTGAGTAGATTC

TTCAGTGTTTGGAGATTTGACTAGTTCCTTTTAACTTCAAGTTGTGCTTA

GGAGCCTCAGACTTGGCAATTTATTGGCAAAACTTAAAACTCACCTTTGC

TTTGATGGCTTTGAATTTATACAGGTATACCAGGAAAATATTTATGTTCC

TGACAAGAAGTTTCACTCATTCAAGAAAATCTCCCGGTCTATGGGGTATG

GTGAGAAAGACATCTCATTAGTATCTTTTCAGTCAGTCTCTAAAGGTATG

TGGGATTCATAAATCAAATTAATATATGAATTATGGAACATATTGAGTTT

AGATTTGATGTGCTGATGTTTTCTTGCTGCTTTCAGGGTATTATGGGGAG

TGTGGGAAAAGAGGAGGTTACATGGAGGTCACTGGTTTTTCTCCTGAGAT

AAGGGAACAAATCTACAAAGTAGCATCTGTGAATCTTTGTTCAAATATCT

CTGGTCAAATTCTTGCAAGCCTGGTCATGAACCCTCCCAAGGTTAGGTTT

TCCCTCTAGATTAGTATTTTTGCATAAAGTGTCATGACTAAATGGGCCTA

AACTTCAAGTTTATTGAATTCAGTTAGATCTGTTTAAAATCAGAAAAATC

CAGAGGAAACGGGTAAGATTTCTGATTTAATACTAGGGATGACAATCTCG

CCCAACCCAACTCTGCCCTGGCCTCGATCCCAATCCCGGTCCGGGCCCAT

ACAGGTTTCTCCCACCTCAAGGAGGAAGCAAGGCGGGGATGGGTTTTCCT

GCCCCTCCCCATTCCATCCCTCCCCTGCCTTGATTATATTTGTTAAATAT

ATATATAATTTTTTTTTTTTAATATACTAAGACTAAAACACTATATTACC

CTTAACACTTATTTTCACGTATTTCTTATCTTACATTTTTTCAACTCTTA

CTACTTTTAAATCATTATACATAATATACCATATCGTCTCTCATTTTTAT

TAGTACCGACATTTCTAAGGTTAAAAAATTTGCCCCGCCCCCAATATGAT

TTGGAGGGGGGGCAGGGGCAGGGCACTACAAACCTGCCCCTTCACCGCCC

TGTTGCCATCCCTATTTAACACTAGTCCACTGCTAACATGTTCTCATGTG

CAGCTAAGCTTGAGTTCCTTTGATCACCTGTTCTGTCAGGTGCTCAACTT

TCCTGTGTCAACATTGCTGTAGTATGATTAAGACTTTTGGATTTGCAGCA

TACAGATTGATTTTTAAGTGCTAGTTTTGTTGACCTTAGGTACATGAACA

AGATCTTGATTGTGCATTATCAAGCTGCTCTAAGGTTTCATTGAAAGCAT

AACTATATTCTCTTAGTATGAACAAAACTAGTTATTTCTACCCATAATTC

CCTCTATGTATGTGTCATTGTTAGATAAATGAAATTATATGGATGGTTTT

CAGGTTGGAGACGAGTCCTACGAGTCTTATTATGCAGAGAAAGATGGTAT

CCTTTCATCTTTAGCAAGGCGTGCAAAGGTAGGTTAAAATCAACAATCTC

ATTGTTTGGGTTCAAAATGAGAAATTAATTAAATAGTAGAAAGAAATGTT

TTATTGAATTTTGAAATATAGTAAATGCATATCAGTCAAGCTTCTGAAGG

AAAATCCATAAATTATCTCTTATGCTAGTAATAGGAGCATATTTCTCGAA

CAATTTGGGTATGTTCAATTCACATTGAACTTTTGATTGTTTTGCCAAAA

CAGTATAATTCATCATGTGTTCTTTAGTTGGAAATTATCTTAGTCTTCGC

CGTATATATTTATTTTTTTGGTAAAACAGACATTGGAAGATGTATTCAAT

AGCTTAGAGGGTATAACATGCAACAAAGCAGAAGGAGCAATGTATCTCTT

CCCCCGTCTTCACCTGCCCAAGAAAGCAATAGAGGCAGCAGAAGCAGCAA

AAAAAGCCCCAGATGCATTCTATTGTCAACGCCTCCTCAATGCCACTGGA

ATTGTTGTTGTCCCAGGTTCTGGCTTTCAACAGGTGAGTGTTGTTGCTAA

TATTGCTTTTTGATTCACACATTACTGTTAGCATTCAAATCTGATTATTC

ATATTGAACTGCAATTTTCCCTATTCAAATGTCAGCAAATAGATGGTCGT

ATGCGTTGAATTTCTTCCAAATTCCAATATCTGAAAATATCCTGACCTTA

GATTTGACCATCGAAAATATCTTTCTGGAGTTTTTTTTTTTTTTTTCCCG

GCAAGTAATATATATTTCTTTAGGGTAAATTACACTTTAAACCCCCCTAT

GGGGTAAATGTATTTCTTTAATTGCTCTCAAGTATTTAGTGTTACGCTTA

TTCCGTGTATTTATATTAGGTGTCTGTTGTACTTTGATTGGCCTTCTCTG

TATTTCTGAATAATGATTATTGATTGTGATGTTATGTTTTGTCGCAAAGG

TTCCTGGAACGTGGCATTTTAGGTGCACAATACTGCCTCAAGAGGATAAG

ATTCCAGCCATCGTCACCCGCCTGACAGACTTCCACAAGGGATTCATTGA

TGAATTTCGTGATTGATTGATCCAAATCCAACCTTGCCCCTTGTGTTGAA

GGATGTTTCTTGTTCCTTTCCAGTTTTCACCTTTAACAGGGAGGGCATTT

TACGGTTAAGTCTTGTTTTGAGTTTTCATATCTTGAATAAGTTTGCAAGC

TGCAAAGGATCTCATCTTGTTACAACCACAGGGGTTTAATTTTCCAGTTT

TTGTTACAGAGAATGTTGAAAAAAGAACTGCTTATAGAGGCACTGCACAT

GATCTTTTTTTTGGATATTTATTGGAAGCCTTGAATCCTAGCTGGGATGT

TGTAAAAGCTGCGAATGCCCAAGGAGTTTTTGTTTTTGTTTTTGTTTTTC

TTATTTTGTATGATGTTTACTTTTTCCTTTAATATAATTGTGTTTTAACT

TTTCTAAAAAAAATAAAAAAAAGAACTGGGGCCATTTTAAAATGTCATAT

AGAGCAACCGAAACAAGCAATGAATTATTGAGTTATGCTACTGATCATGC

ATTGCTTGTCACACCAAGCTAAAATTTTAAGAAGTTTAGGTACCGTTTGG

TAACACTTACATTTTCAGTTTTTTATTTATATTTAGTTTATGAATTTGGT

TAAAAAATGAAAACACAAAATGTATTTTCATTTTGAGTTGAGTTTTTTTT

TTTTTTACCACCCAACATTAGCCACCACCCGTCATCATTTTCCAC

>TEA024296.1 locus=Scaffold988:896692:899019:+ Putative tonoplast intrinsic protein

ATATAAATGGCCCTATATCACAAGCTGCCAAACCAAAAGCAAACCAAAGC

CAATCTTTTGGCCTTTTCCCATTTTTTTGAGAGTAGAAAACCTCCGGTGA

TCGGTTTTCCGATCAGCTAGACTCATAAATGGTGAAGATCGCGTTAGGAA

CCGGCCGAGAGGCCACACAGATTGACTGCATCCATGCCCTCATTGTTGAG

TTCATTTGCACTTTCCTATTTGTGTTTGCCGGCGTTGGTTCTGCCATGGC

TGCCGGTAATTCATTCCATACCATACAATTTTTATTTACATATTTTTTTT

AATGAGAAAATGATTGTTGATTTTTAAATCAAAATTCAATCCAAGATCAG

TTGATAGTATATTTGGTTTGGACTAAGAGTGTGCTACCACTCTCTAACGT

GAATTTCGACTTACAAATCATTTTGATGAATATGTTATAATTTTTTAACC

ATTTTTTTTTAGATATCAATAGATACAAACCCACCACTTTGTGTCTAGTG

ATTAAAAAATTAAATGAAAATTTTATTTATGTACAACATTACACCAATGT

TCAGGGATTATGCTTTTTGGGTTGGCTATGAAAATTGTTTTGCTTTTTTT

TTTTTTTTTTTAATAGATAAGCTAGGTGGAGATTCTCTAGTGGGCCTGTT

TTTCGTGGCCATGGCTCATGCACTGGTGGTTGCGGTGATGATATCGGCGG

GTTTCCGTATCTCCGGTGGTCATCTTAATCCGGCGGTAACGCTCGGCCTC

TGTGTTGGCGGTCACATCACCGTATTCAGGTCGATCCTCTATTGGATCGA

TCAACTCTTAGCTTCTGCAGCTGCTTGTGCTTTACTTAAGTATCTTACTG

GAGGATTGGTAAGTCAAATAATTATAAAAATATGAGAATTTGTAAGGTGT

AACTTTTGCTGTGTTTTTTACTCAGAAAAAACATTTGTAATTATTTTAGT

CCGGTTCCTAATTAGGTTTTTGTTTTTTTTAATGTTTGCAGACTACTCCT

GTACATACACTAGCCAGTGGAATGGACTACTCCCAAGGAGTGATAATGGA

GATTATCTTGACATTCTCATTGTTGTTCTCAGTATATGCCCTTTGTGTGG

ACCCAAAGAAGGGCTTTCTTGATGGGCTAGGTCCATTACTAGTTGGGCTT

GTAGTTGGGGCCAACATCATGGCCGGTGGGCCTTTCTCAGGTGCTTCCAT

GAACCCAGCAAGATCTTTTGGGCCAGCCTTGGTGAGTGGAAACTGGACTG

ATCACTGGGTTTACTGGGTCGGACCGCTCATCGGTGGTGGGCTTGCTGGA

TTAATCTATGAGAATTTCTTCATTGTTAGGTCTCATGTTCCACTTCCAAG

AGAGGATGAAACTTTCTAGGGTTGTGCCAAGTGCTCATAATGTCATTATG

TAGTGTTGCCTTTGGCTTTTGATGTATGGTACTCTGTTTATTGCCTCTTT

TTTATTGTAAGTTTCCTGATGATGGAGTCTTGATTGGTATTTACTCTCTC

TCTCTCTCTCTCTGTCTCTCAAAGCTGGGTATGTTGTGATAGTAATTATA

TTATATTAAATGGGTATTGTTATGCTTTATTTGTTTAGAAGAAAAACATC

TTTTGTAAACAGCCTAATGAGTTTTAATACGAACCTGAATCCCAGCCTAA

CATGACTAATTTTTGCTGAATGGTCGGTTTGGTTGGGATTAGACTATAAC

CCATCAATTAATCAAACAACATTGAGTTGATACTCTTAATTGATTTTGAC

TGGCCACTCAATCGAAACCATCCGTTACAATGTGTTTGGTTGAGAATTTA

AGGAAAGATTTAGAAAGAGAAAAGGAAAATGTGGGTAAAATTAGGTGAAA

TGGTATATTTTATTCGTATTTCACTTACCTTTTTCATTTTTCTTTTTAAG

TTCCTACTCAAACCCCCCAAACAAACACAGTATTAATGTTAATTAGTTTG

GCAATTGGATTTCATCATTTGGGCCATATAGCACTTTTGGGCCGCTTTAG

TTTTGGCCCAAAACTACTTTGTACCAGGGCCACTTTCAACTTTTAAAAAG

AGGTCATCTCAGTACCAGGGCCAATCTAGTACTTGTCAATTTTAACAAAA

GACAACCCATTTTGCATTTTATGGGCACCACAAAAAAGAAAAGAAAAAAA

AGAAGTGTAATTAGTGGAACTTCTTGCTGCCTATCTCAAATTTTCAAGCC

TCTTTTTCATTTTTTCCGTACTAATGTCAAGTGGTCCCAGTTGATGAAGC

CTATGAACAAGCTAGTAAGGCTTATGAGGTGACGAGACTCACTAATAGAT

TGCTTGGTTGTCTATTTATGAGTGATGC

>TEA008965.1 locus=Scaffold1085:1932110:1936285:- Tonoplast intrinsic protein

TATATATATAGCAAGTTCCACACTTTCACTTGATCAAGGAAGCTAAACAT

AGAGACAGAGAGAAGAGCAAGCAAAAAAAGGGCATTTTGCAAATAGTTCT

CTATTAGCTAGAGAAGGGTAAAGGAGAGTAGAGTGTTAAAAAAAAAAAGA

AAAAAATGCCAATTTACAGAATAGCTGTGGGGACGCCGAGAGAGGCGAGC

CACCCCGACGCTCTCAAGGCGGCGTTGGCGGAGTTCATCTCCATGCTCAT

TTTTGTTTTTGCAGGAGAAGGGTCCGGCATGGCTTTCAGTAAGTTTTCGT

AACTAATCTCAATTTAACTTTATTTTTGAAAAATAATTTTTTGATTTTTT

AACTAAAATATAAAATAAAAAAAATTAGTACGTTTGAAATATTTTTTAAT

TTTTTATAAAAATATATAAACAAATAAAATAAAATAAAATTTGATTTTTT

AATTTTTTAATAATTTTTTAATTTTTAAGTTTAAATAGATATGTCAAACA

AAGCCTAATTTTTGACTTTTTTTAATATACCAATTGCAACTATTTTTATA

ACGATATAAATTTGTCTCTAAATGTCTTTTTGCAACAAAATTTTCACTTT

TAGAATTCAAAATATTTAGTTTCTAAAAAAATAGTATGTTTCACTCGAGG

CCCAGCATCATTTGAAACTACCTTTTTTATTACTTTTCAATTGTCTTTTT

AAAAAAAAAAAAAATTAAAGAACTTTTTAATGAAAAAAATACTTCTAAAA

AGTAAAATTATGAATATTAATTGTTGATGTCAAACTTAACCTGAAATACA

AAAGGAAATACAAATGGTGTGTGTGGCTCTCTCCAATATAAATCCTCCCA

AGCTTGAATAAAGGGCAATAGGAGAAAGTTCTCTAGTATGAACCTCAATA

TTCTTATGTAAAAATTTATTTTGTTGTTTAATGTCTATAATGATAAATAT

TTTGATTTGAAATGAATTAAATAAACACTATCACGCTCCCTGGAATGGGG

TTACTGCTGTGTAAAGGCTTGACACAGTAGTAACTGTTGTGCGTTAATTT

GAGCTCCCTGGAATGGGGTTACTGCTGTGTAAAGGCTTGACACAGTAGTA

ACTGTTGTGCGTTAATTTGAGGTTTATTATGGGCTTAATTTTATAATTTG

AATTGTTCATTTTATAAGATTTATTGAGTACTTCATACTTATTAAAAATC

ATATTAATCGTTTATCAATAATTATGGGTATGTTTGGTTTGTATTTGAGA

GATGAGTTTTAGAAAAAAATTTCTAAAAAATTATTTAGTTTGAATTTTTT

TCAAAAAATTTTCAAAATCACTTTCTCCCTCTTGGAGCGGGGTCACTGCT

GTGCCCCTCCCTGCTGTGTTTTGCTGTGCCGTGAAACGGCACAGTTTCAT

CCCCTTCTTTCTTTTTTCTTTTTTTCTTTTGCAAAGTTTTATCTTCTTTT

GTTTTTTTTAATTTTTATTTTTTTATTTTAGAGTTCAGAGTTTAGGATTT

AGGATTTAATTTTTAAAATTTATTTTTTTATTTTAGAGTTTAGGGATAAA

TTTTTTTTTATTTTAGGGTTTAGGTTTAATTCTTAATTTTTTTTTTATTT

CACGGTTTAGGGTTTAGGGTTTACAGTTTAATTCTTAAATTTTGTTTTTT

ATTTTATTTTAAGGTTTAGGGTTTAATTCTTAAATATTTTTTTTATTTTA

ACTGTTGTGCCCCCTCCCTACTGTGTTTTCTTTTTTTGTCATGCTCTCAC

ACAGTTTGATCTTCTTCTCTTTTTTTTTTTTAATTTTATTTTAGAGTTTA

GCGTTTAGGATTTAGGGTTTAATTTCACTTTCTTTCTTTTTTTTCATGTT

CTCACTATGAATTAAATCTAAACCCCAAACCTAAAATAAAATAAAAAATT

AAACCCTAAATCCTAAAATAAAAATAAATAAATTTTAAGAATTAAACCCT

AAACCTTAAACCCTAAAATAAAAAACAAAATTTAAGAATTAAACCCTAAA

CCCTAAATCATAAAATAAATAAAAAAAAATTTTAAGAATTAAACCTAAAC

CCTAAACCCTAAAATAAAAAAATTAAATTTTAAAAATTAAATCCTAAATC

CTAAATCTTAAATCCTAAAACAAAAAATAAAACTTAAGAATTAAATGCTA

AACCTTAAACCCTAAAATAAAAAAATAATAAAAAAAAATTAAGAATTAAA

CCCTAAACCCTAAAATAAAAAAATAAAAAAAAATTTATCCCTAAACTCTA

AAATAAAAAAATAAATTTTAAAAATTAAACCTTAAATCCTAAACTCTAAA

CTCTAAAATAAAAAAATAAAAATTAAAAAAAAAAGAAGAAGATGAAATTG

TGTGAAAGAAAAAAAAGAAAAAAAAAAAAGAAAGAAAGAAGGGATGAAAC

TGTGTCGTTTCACGGCACAGCAAAACACAGCAAGGAGGGGGCACAGCAGT

GACCCCGCTCCCATCTTTCTTACACATCAATTAATTACTTTTTCTCTCTC

CACTCTTCTTCTCAATCACTTTTTCCCTTACGCACATCTACTCAAATTCT

TTCAAATTATCAAATCATCACAAACTCCCAAAAAAATAACATCTACTCAA

ATTCTTTCAAATTATCAAATCATCACAAACTCCCAAAAAAATAACTCTCC

CAAAATATACAACCAAATAAACTAAAAAAATTTCTAAACTCTCTCTCCAA

ATTTTTTTTCAAAAACAACTCTTCCAAAAACAAACCAAACAATAATCCAA

TTATATTTATCATAAAAAAATAAACAATAATTTTTTTTTTTTTGAGAAAT

AATATAAAAAATATGTAATCCGATCGTATAATTATTGATATCCGATGAAT

ATAATTTTTGGTAAGCATAAAATACTCGACGAATCTTATAAAATGAACGA

TTTAAATCATAAAAATAAATTTATAATAAAACTCAAATTAAGGCACAATA

GTTATTACTGTGCCAAAGACACATCCGTAACCCTCTTTGCACCCTAGGGC

ATTACAAAGTCACGACTAATAGAATAATTAATTTGCATTTTTTTGCTCAA

AACCTTTTATTAATGGATTCAACTACAATACAAAACTAGTGGTTTTTATA

ATCTAAATCTAATTGTTAGAAATGATTTTTGGATACATTTTTTTTTTTTT

TTTTGAACCTTTAAAAAAATTCTTTACCTATTTATGATGGGCCCAAGAGG

ACTAAATTAGTGATGGATGGTCTTGGGTTTTGACAGATAAGCTGACGGAT

AACGGGTCGACCACTCCGGCTGGGCTTGTAGCTGCAGCATTGGCTCACGC

GTTCGCTCTTTTTGTGGCGGTTTCGGTAGGTGCAAACATTTCAGGGGGTC

ATGTGAACCCTGCTGTGACATTCGGTGCCTTCCTCGGTGGCAACATAACA

CTGTTGAGAGGCATTCTGTATTGGATTGCACAGTGTTTGGGTTCAATTGT

CGCTTGCTTGCTTCTCAAATTTGCCACTGGTGGATTGGTAAGCAACTCAT

ATATATATATATATAACACTATATGTGAATTTCAAACTAATCTTTTGATA

CAAATTTTCATCAATATATCTATGGGTTTGATCAACTTTGAGGGTCGGGG

GGCCATTACGAGTGATTTTGATTAGATAATCGTAACATTTTTATTTTATT

ATATTTGTATTAAAAAAAATGAATGATTTTTGAAAAATTTCACACTCGAT

CCCTTGCAGGAAACATCTGCATTTTCCCTCTCGATTGGCGTCTCCGTATG

GAACGCATTAGTCTTCGAGATCGTGATGACATTCGGCCTAGTCTATACTG

TCTACGCCACGGCAGTTGATCCGAAGAAGGGCAATGTGGGGATTATAGCA

CCAATCGCAATTGGTTTCATTGTGGGTGCAAACATTTTAGCAGGTGGTGC

CTTTGATGGTGGGTCCATGAACCCAGCAGTGTCTTTTGGTCCAGCCGTGG

TCAGCTGGACATGGGCCAACCACTGGGTCTATTGGCTCGGACCACTCATT

GGAGCTGCCATTGCAGCCCTTGTCTATGATAACATCTTCATCGGTGACAA

TGCACATGAAGCACTACCCACCGCGGATTATTAGAAAAGTTTCTAGGTGC

TCTTTGATTTCTTGATGCAAAATCATGTTTTAGTTGTTTTGTGTTGTAAT

TTTCTCTTCCTTTTTTATTTAAGGAG

>TEA015198.1 locus=Scaffold1167:1184291:1200743:- protein fluG

AATGAGATTTGTAATTCTATATAGAGAGGGGAAGTCAGAGAGTGATTTGT

AGAGGGATAGAGTTTTGATCGTAGAGAGAGAGAGAGAGATGGAGAAATTT

GCAGAGCTGAGAGAAGTAGTGGAAGGTGTGGAGGTAGTAGACGCACACGC

TCACAATCTGGTGGCTCTGGACTCCACTCTTCCTTTCCTCCAATGCTTTT

CTGAAGCCTACGGCGATGCTTTATTGCTCGCACCCCACGCTCTCAACTTC

AAGGTACATACACCAAACACCAATCAATATCTTCTTCTTTTTGTCTCCTA

TTTCTCAGTCTCTACAAGTTTCAACCTCAATTTATCAATTTTGTACAACA

ATCGGTTTTGATTCGTTGTCTTTTCCTTATGGAGCAATTTGCAAACTTGG

TAAAAAAATCAAATTTTTAATTTTTACCAAGTTCTTTAAAAAAAAAAAAT

AAGTTACAAAAAAATCACTAAAATAGTCAATTTTTTTGCCCAAAAAAACC

AAAAAAAAAAAAAAAAAATCAATTTTTTTATCAACTTCTCAAAATATTCC

AATGTTTTGAAAAAATGATGAAAAATTAATTTTTTTTGATTTTTTTTATA

AAAAAATAAAGGTGATATGACTAAATTTTTTTTTAAAAAAAACTAAAAGT

ATCTTTTTAAAGGCGTTGGCTCTGGACTGTGCTGTTGGGTCCGTGGGGGT

CTACCACGGATTTTCTTTTGATAATCTGAACCGTTAATGTGCAGAACTCG

TTGAGTACTATAAGTTTGTAAAAAATCACGTTAATCGGATATCGGTAGCT

ATGTGATCAAATCAGATTTTTGTCAACCATTAATTTATTTCCCATCTATT

TTTTTTATACAAAACTAATTTGATCACGTAACTACCGATATCCGATTAAC

GGGATTTTTCATAGGCTTATAGTACTCGACGAGTTCGACACATTACATGA

TTCAGATTATTAAAACAGAACCCGTGATAAACCTCCACGAGCCCAACAAC

ACAGTCCAATCCAATCCGCTTAGGGACTGGACAGAAGCAAGCCCTTAAAT

ATATGTTTGGTAAAAAGGGTTACGGTGCTTTTGGCCTGTCAGTACAAATC

CAGAATTTTTAAAAGTTGCCCAAAAAAAAATCATTGTGCCATACAGATTC

ATTTTACCAAAAATGCGCCGGACCCAGTAAAACCTACCCCACCGACCCCC

CAGTGCCATGTGGCACTGCAGGATTGAAAGAAAAGAAAAAAAAAAAACAA

AACAAAAAATTGGCCAATGGAGACCTGCCATCTAATTACAAATATTACAT

CTAATCACAAAAAAAACTCAACTACCTTCCTAAACACTAAATTTTTTTTT

TCCAATAATATCCTCTAACTAACTAAATTTTCTTTTTCCAATAATATCCT

GTAAGTAAAAAAAAAACTTACATAAAAACTCACCTCCCAAACACCCACTA

ATTATTCAAGAATTTATATTTATTTCATAAATATTCAAAAGGGTATGAAA

CTCCACATTTGATTCAGTCAAAATAATAAGTCTCAATGTCTCATATTCAA

TAGAGTTCAGCCATGGGGACACCAATTTTTACTGCATATTTTAAAGGTAA

TTACAAATACATTCTTACAGTTTATTTAAAATATAAATTATTAGAGTTCT

TTCCAATTATATAACAAATGATCTATTTTATTAATAAATTATTAAATTAA

GTAACAGTTTTAATAATGTCATAAATATATTTTAATTAATTGAGGTGATT

TTATTGTTTTATAAAGGAGTTGTTAGAAATTTTATTTTTTCTATTAATCT

TGTTAAATAAATTTTTTTAATAAGGAATGTTTATAATTTTTAAAATTATA

AGAACAATTCATATAAATTTTGTGTTTGTATTTTACCCAATTCTACCAGT

TAAGATGGTTACTTATGACCCACTCTCAACTATAAGACCATCTCCAATCT

TTTTTCTATTTTTTTTTATTTATCTAATTTAATTTGATATGAATTATTTT

AATCATTTCGTCAAATAGAATTGTGTGGTTTAATTGACTTTCGTAGGATT

GTATCTATATTTAGGAGGAGGGATTTGCTGTCTTTGTTGTGGGGCCCATG

TTGGATCACACTTAAACAGGTCAAATGAAAAATCAAATTGAACGAATTTT

ATTGTTATATGATTAAATTGATTTATTTAAAAGAAAAATAAGTCATCCAT

TTTTATCAAGATAAATATTAAATCTGATCAACTTGAATTTTTGGATATAT

TCTCGATGAACTATATAAGATAACAGTTTGGATCTATGGGGTGGATATAC

AACTGAGGGGCATATCAGGACTTCTCATCCAATTACAAGAGGTTTCAAGA

AAATGACCAAGATTTTGTGATAAAGACTTAAATTATTTTATGATAAAGAC

TTAAATTCTATAAACTTGATTTTTAGTTTTTTTTTTTAATTTAAAAAATA

ATAAATTTCAATTTTTTGATGTTTAAACCACCATGTGTGGGCGAAAAGAG

ACATATTTGAATGACTTCATGAATTTTGTTAAGTTGTAATAATGCCTTTT

GACAGAGGCTAAAAATTGACTATTTGACAGCAATAACTAATGGCTTCATA

AAACTTTTGATACCATGTATGGAATTTTTTGATTCTCAATTTAAATTGCT

TCATCAATTATTATGATCTTTGTTGTTTTCCAATCTGTCATCACCTCTTC

AGTGGTTCCCCTGTGGGACATTTCACTTATTGTTCCTCTGTTATTTGCTC

AAAAGGACATGTTTGTAGACATTCTGACTGAAGTTCTTGGACTCACTGTT

TTGAGTGGCCTTTTGAAATTAATGATATAATGGTAATTAGTTAATCATCT

TTATTTTTTGGTTTTTTTTAATTTTTTTAACTATAGACTTTTTTTTTTTT

TTTTTCGCCAGATAGTCACTAGAAAATTATAGTTAGTTCTAGTTAGAAAT

TAAAATGACAATATTCTTTTTAAATGTTCGATAATGGCATCCAGAAGGAT

ATGTTTTAAATTTTAATTGCATTTGGATACGAGTATTTGGAGCCAGTGTA

TGTCCAAGAGGCAAATCTTATTAAATCCTAAATCTTAGGACATACGGATA

TGTATCCAAAAGTTGGATATGGGTAGCTGAACACATCATGAACACGGATA

CAGTTATATTGCATAAATGTTATAGGTCATTTAGGAATTTACTTAAAATT

AGATGGGATTTAGGGGAAAAGAGGATTTCTATCTGCCTTCCCATATGAGA

GGTTATAAAATTTTCTTTATTAGTAAATTTGAGTTTCTCTTCAAAACACC

ATTAAAAAAAAAAAAAGAAGAAAAAAAGAAAAGTTAACAGGATCTAAGTG

TCTAACTTGAGTGATATTTGATGGACACGGATACAGTCAGGAATAGAAAA

GTCCATAAAACTTTGGTTCCCATTGCCTACGTTGGCCTCATCAGCAATCT

TGAATTTCGACTTCTATATATATATTTTTATTGTAGCAATATGCCCAGAT

GTTATCTGGTTCAACTACAATTTTAATGTCATTTAAAGTAATGAATTTTA

CATGCTTGGTGGAATTATTATTGCCTACTCGCTGCAAGCTTTGCTGTGGT

CTTGCACTGCTTGCCTGAGCCCATAAAGGATGTCAGTTACCTCATCTTTG

AGGCTTATCTAAAAAACCCACTGCTCAACGTGGGCGAGCTTTCTCTACCT

CATTGTTAATGTATTTGGTTTCAACCCATTTGGCAGTTACAGATGAAGAT

TCTTTGACATGTTTGCTGGATTGTTATGAAAATTTATGTGTAAGGCTTTC

ATTCTGACAGCACTCGTTTGCATTTTTCAGAGAGGTATAAGGGATATTGC

TGAACTGTATGGATCTGAGTTATCCTTGGATGGCATTCAAAAATACCGCA

AGGGCAATGGATTGCAATCCATAAGCTCAATATGCTTCAAGGCTGCAAGA

ATCGCTGCAATACTCATTGATGATGGAATTGAGTTTGACAAAATGCATGA

CATTGAATGGCATAGGAATTTTGCACCGGTGGTTGGTAGAATATTGAGAA

TTGAGCATCTGGCTGAGAAGATTCTTGATGAAGTGAGATTCTTTCAATCT

TTCCCTCTGGGTAGACATAGTTATACATGTTATATTTATAGAGTAGTTAA

TTATTATGAATGTATCTTTTCCATTGCTAATGCATTTCACTTGGTTAGAT

TAACTTTTGGTAAAACTATAATTTTATGCTGTGGTCGGCATTAATTAATG

CAAAAATAATTTTATAGGTTAAATCTACAAAAAATGTAATGTTCATGATA

CTACCATTTTCATCAGAAGATATTGTAATTGGCTTTCTCATTTGATTCGA

TGTTCATCTGAAAATTTAGTCACTGGCATTAGGTTTCTAGCTATTGCCTT

CTTTGTCACTTTCCAAAGTAGAAGTGAAGCAGTTGATTAATGATCACATT

CCATCCCAAGTTTGATGATTGTAACAAATTTTATGTTGAACACGAAGATT

TGTTATGTCTAATATACAGTTAATTTGAATATTCATCATTTGGGATCATT

TCCTCTGGCTAAGTGAAGAGGGCATTCTACTTGCATTAATACTTTCATAA

ATATTCCAATAGAATGATTTTGTTGAAGAATAATTGCATGAAATAAATGG

AAAACCTGAGTATGAAGTGGTTGATGGTATTGAGATATGAATGTATCTTT

TCCATTGCTAATGAATTTGACTTGGTTTGATTAACTTTTGGTAAAACTAT

AATTTTATGCTGTGGTCGGCATTAATTAATGCAAAAATAATTTTATAGGT

TAAATCTACAAAAAATGTAATGTTCATGATACTACCATTTTCATCAGAAG

ATATTGTAATTGGCTTTCTCATTTGATTCAATGTTCATCTGAAAATTTAG

TCACTGGCATTAGGTTTCTAGCTATTGCCTTCTTTGCCACTTTCCAAAGT

AGAAGTGAAGCAGTTGATTAATGATCACATTCCATCCCAAGTTTGATGAT

TGTAACAAATTTTATGTTGAACATGAAGATTTGTTATGTCTAATATACAG

TTAATTTGAATATTCATCATTTGGGATCATTTCCTCTGGCTAAGTGAAGT

GGGCATTCTACTTGCATTAATACTTTCATAAATATTCCAATAGAATGATT

TTGTTGAAGAATAATTGCATGAAATAAATGGAAAACCTGAGTATGAAGTG

GTTGATGGTATTGAGAGAGAAAGGAAGAAGCGGAGGAATTGAGAAATACT

TCTTCTGGATCACAGTTCTATGTTATGAGCAACTAGCATCCGGCATAAAT

GTGGTCCTCTTGTCCCACAGTAAAAATTAATCAATATAGTATGAACATCA

ATTGGGAGTGTAGCTGGGCCCAAGTGCTTGGGTGCTTGATTGGGAGCAAA

ATGAGAACTGGTAGACATTTTCTAGGGCACTGAAGAGAAAATATTTTCTT

GTCAGGAGTTACTTCAGGCTTGTGTGTGTGAGAAGAATCTACTTACTATC

TGGAATTTTCTTATTATTGAAGGCTATGAACTATTATTATCTGGAATTTT

CTTTTCTTTTCTGTTTTTATTGTTAATATTATTTATTTATTATTTAGGGG

AGGCCAGATGGATCTACCTGGACATTGGACAGTTTCACTGAAACATTTAT

CGGAAAGTTGAAGTCATATCCTTCCAGATTTTGTTTTAGTTTGTTATGTT

TTTTTGTTTTGCAATATTGCAGATACCATGCCTTTTGTTCTGTTTCATCT

TTTTGGTTAGTAGGAGGGAAGGAAAGTAAGTGAGCGAACTTGTTTGAATG

GGGGGATTTAGAAAACCAACCTCCTTTATGTTGTGTTTAGTTGGGGGATT

TGGGAAGGGATTTGGAAAGAAAAAAGGGAAAAGGTGTGTGAAATTAGGTT

AACTGTAGGTATATTTTATTCATATTTCACATACCTTTTCCCTTTTCCTT

CTCAAATCCCTTCTCAAATCCCCCAACCTAACACAGCGTTAAAGCTCTCC

CTTCTTTCATTGTTGTTTCCACCCATTTTTGGCCAGATTGATTTTGGTGA

GCCTAAACTCGTACTCCTCATCTTCTCCTCCTTTCCCTTCTCCTCCTCAT

ACGAAACAAAGGAGAGTTTCTTAATTCCTTCCCTTTTCCTTAACCCAATT

TCCTTCCTCCCAGTTTCTTCTCTACTAAATGTACTGTTGTTTGTATAAAC

AGATATTCCTTGTTCCACATAGTTTCCTTATCCCTTTGTTACAGTTGCTA

ATAAAATTGTTGGCTTGAAAAGCATAGCTGCATACTGCAGTGGTCTTGAG

ATTAATACAAATGTCACAAGGAAGGAGGCTCAAGCGGGTCTTGTTGAAGT

TTTAAATGGTGAGTATTAGGACAATGTAGAATGGATTTTCATTGTTCTAA

GTATTCACCACTGGATATTCTAATCAGTCTGTCTTTACCTTTACAGCTGG

GAGCCCCGTTCGTATCACAAATAAAAACTTCATTGACTATCTCTTCGTGC

AGAGTTTGGAGGTTGCCATACAATATGATTTGCCAATGCAGATACACACT

GGGTAATGCGTTTACGATTGTTAAGTGGTTTCATTTGATGTGGTTGATTG

CTGAGACATTTGGAAGAAGAAAATAATAACTTGCAATGCTTTTAGAAGTT

TGGTATGGGCTAGATGTCCCAAGATATCAACTTATTTCTTTTTCACCTGC

CCTATTGTGATAGATCAAACAGAGAATTAAGGCAAATCTGTTTTCTCTTT

TTGTAGTGCTTGTTTGACTTTGACTCTTTATTGACTACATTTTTTTGTTC

ATTCTTTCCTCGCATCAGTTTTGGAGATAAAGATTTGGATTTAAGGCTCT

CCAATCCCTTGCATCTCCGCACCCTTCTTGAGGACAAGAGATTCTCTAAG

TGCCGCTTAGTACTTTTACATGCATCATACCCATTTTCAAAGGAAGCATC

ATATCTAGCCTCCATTTATTCTCAGGTAAGATATTGAAGATTACAATGAA

CAATTTGTTGGGATGTCTGTCTTTGATGTTAAACTTGCTGGTTCTCTAAC

AAAGCAATGCATTCAATTTTTAGGTTTACCTTGATTTTGGTTTGGCTGTT

CCAAAGCTTAGTGTTCATGGGATGATATCATCTGTCAAAGAACTTCTGGA

GCTAGCTCCAATAAAGAAGGTGAGAACAGAACACATGCACTTGAAGAAAT

ATTGGAAGAAATGCTTGTTTATTTAGTAAAGAAATCTCATAAAAAAATAA

AGAAAATTAAAAAACTGGCAAAATCTGACACGATAAATTTCTCAAGTTTA

GAGGTTATAATTAAACATTTTTACACTTTTGTATTGAACTTAGATTAATT

TGTTTAAAATCATAAGGTGAACGGAGGGTAGAGGATAATAATAGGAGAAT

GCGAACTGTAGGAGGACGTGCTGCAATACTCAAGGCTCTTTTGAATTTCC

TTCCAATTTTTATTCATCAGTTAGCTATTCCTGTTTAAATTCCGTGAGAG

TCAAGGCTTTTTCAAGAACCTAAAAATAACTAACTAAATAACTCTTTATA

AGAATCCATCTGCTTTTGACTCACTCGCACATTAAATAAACATTCAAAGT

TATTTATTTCTGGTGGGCCTCCATTTATGTGATCTTTATTTGACAATTCA

GGCTTTTAGATTGTGTCACAATGTCCTATAAATGGACTGTGCTTTTTTTT

TTTTTGTGTGTGGGTGTGTGTGTATGCCTGGGATTGGCCTGATTTGTCCT

CTGTTTATTAATTTTGTAACAATTTTCATTTTCTCTATAATTCAATTTGC

AGGTGATGTTCAGCACTGATGGTTATGCATTTCCAGAGACTTTTTACTTA

GGTTGGTTTAAGTAGTTATCTATCAATTTTGTACTTTATTTTGTGATAAT

ATCTTGAAAATCTTGGAGGAGCCAGTTCTGATTGCAGATTGAAATTAATA

TGAATACAAAATAGAGCCTATTTCTCTTGTTTCCTGGTCTTATTTTCTAG

TTTATTGTTTTTACAGGTGCAAAGAGAGCACGTGAAGTTGTCTTCTCTGT

TCTATGTGATGCATGTATTGATGGTGATCTTTCCATTCCTGAAGCCATTG

AAGCAGCCAAAGACATATTTTCAGAGAATGCAAAAAAGTTTTACAAGATT

AATTTATATCTCAAACCTTTTGATTCTAAAATCAATGAAGTTTGCAAAGT

TGTGAAGATGGAGACTGATACCGTACAATCAGATGTTGCCTTTGTTCGCA

TTATCTGGGTTGATGTTTCTGGGCAGCACCGGTGTCGTGTGAGTTACTTT

ATTCATTAACTTTGTTTGGTTAGTAATTTTTGGGTTCAAATATTAATATT

TTTAAGAAGTTTGACATATTATTAGAAAAGTTTGTCGAATACAGCACAGC

CATTATTCTATGAAAACAGCATGCTTGAAACAAACATGCCTGTTAAGTGG

TAAGAGAATACTTTAAGTTCAGTGGAAGGAGATGGATGGAAATCATCTTG

AAGTCTCACATTCATCATGGGGATTGATGACTTCTTGATGACTGATTTAA

AATTAAAATTGTGATTTGCTATTCTGGTTTGGTAAGTATTGAAAGGAAAA

AATAAATATATAATTAATCAATCAAATTTTAGTTAAGTTAGATTACTTTT

CATAGTTTACCATTTTCAAATTAGTCTGTAACGTAGGTTCCTGTACTTGT

ACAAAAGTTTATTTGATTGGTTCTCACACTAGACTTGATGTGATTTTCCA

CTTTGATTCCAAAACTGCAGGAGAGCCATTTGGCCCTCATTTCTGCTATC

TTCTTTAAAACCTTGATCAAACTAAGAGACCCCTTTATTGGGTATGCATG

TTATAGTAGTTAATCAATTAAATTAGATTCCTAGAGTAGACTTCATTTTT

GTATGAATTTTTGTTACCAAATGTTGAGGTAGTTTTTTTTATTTTATTTT

TATTTATTTAAAATGTCTGCACACCAATGTGTGGCTGGAAGTGATTCAAT

GGTATCATTATCATTTACTTGTGAATTTTGATATAGGGTATAGTATACAA

TATAGCAGTCTTGGCAGATTGCTATGAAAATCCATTGTTTTAAGACAATT

TTATAGATTTAGAACAACAGCAAAGTTAGATATAGTTTGATTTATGTTTA

TTCGTTCCTTGTATATCATTTTTCCAGGCTGTTCCAAGAAAGCGTTTCCA

TGATGTTGTTGTAAAGAATGGTCTAGGCCTAACTGTCGCTTGTATGGCTA

TGAGTTCAGCTACTGATTGTCCTGCAGATGAGACTAATCTGACTGGTGTG

GGTGAGATCAGGCTAATACCTGATTTATCAACGAAGTGTATAATTCCTTG

GTAGTACATCTCTTTCATAAGTATTACCTTTGTCTCTCCATTGCAGCATA

ATTTTTGTTAAACATTCACGATCTTTTTTTATTTTATTTTTTATAGTTGC

ACTGTATATTTCACAGATCAATATCCCCGTTGTCAAAACTTGAAGAGTGA

AAATGCAAAGAGGAAAAAAAAAACTCTTATCCCAAACATGGAAGATGTAC

ACTAAGGGCCAACAAGATTGTCCCAAAAATGTGATTTGGGCAATTTTAGA

GTTTACCCATTTAAATATCAGATAATTGGACGTGTTTTGACTATCAAAAT

GAAACAACCCAAAACTTGATAAAGACAAAAGTTTTATTCTAAAATTGTGG

AATTCAAAATATTTTATGTTTAATTTCTACACAAACTTGAATGAACTCAT

TTACGTGTGCATAGGATTTGAATTAGTTAATTCAAAATGGAGCAATTCAT

GCAACCATACAGGCCCTAATAAGATGGTCTGCTTAGCGGTTTATAGTTCA

TTTGAGATCTTCGACAACAAATCATTATATGACTTTAGGATGGCACATGG

CATGGAAAGTAATATTATGTGCCCAGTAATCAGAATAGTTTTTCACATAT

TTTATTCTGTTGGTCTACTATAACAAATGTGGATCATATACATTCAATAA

ACATATGCATATCTCAAGTGTGTTGTCTAGAATCATCTGGTTGGGCAGCA

GATATATTTGTGATCATAATTTAATTTATTTTTTTTGACCTCAACAGGGC

AAAACAAGAAGAAATGGTTTTGGGTGACATGCATCTTAAACCTGGTGAAG

CTTGGGAATATTGCCCAAGAGAGGCATTGCGTAGAGTTTCAAAAATTTTG

AATGATGAATTTAACTTGGTAAGCATGATGGTTTATTCATATGAAATATG

TTGTCATTCAATTTCTAATTGTATATATATACAGGTGATGTATGCAGGTT

TTGAAAGTGAGTTTTATCTCTTGAAGAGTGCGTTAAGGTATGTGATGCAA

TTGTTTCCAGGCCTTCTTTTAATTTTATTTTCCAGTCGATATTGCAAGTA

TTAACTATAAAAATCTGCAGGGAGGGGAAAGAAGAATGGTTTTCATTTGA

TATGACGCCCTACTGTTCTGCATCTGCATTTGATGCTGCTTCCCCTGTAC

TTCATGAAGTTGTAGCTGCTCTACAGTCCTTGAATATTGCAGTTGAACAG

GTAGAAGGAGATAAGTATTGTCGTAGCTTTTTTGTACCGGAATTATGGGA

GGTTGTTCTACTTAGGTCAAATTTTGAGGTCATAATCACACAATCCATAT

TATCACTTAATTCTTTTTGTAACTTCTCTCCGTGGATTGGAAAATGACTT

CTTATATATTCTTGACATTGCTCAGCATGTTAATTCCAATTCTAGTGCCT

CAACCATGTGAGTATATATATGCATGTATTTATTTTGTTTTTTTTAAGAA

AAGAAATTTAAACCACAAAGACTTCATAATGCTCTTATTTCTAGGATTTG

CTATCTCTCTATTTTCGTGACCTACCTTTGAGGTGACCCAAGTTATTATG

AATTGTACATGTGTAGAAAACAATGGTCATTTGCTATTTAATTTTAAAGG

CAGTTGTCTGAACACACAAGACATAAGGGTACTCCCTCTAAATTTATTGT

TGTTATATGCTTTTAGATCCATCATATTGGATAACTTGATCTACACTTTT

GGGCCCATTTCCTAATAGCTTAAGCTTTTGGGATTAGTGGTGATCTAACA

TGGTATTAGAGCTTAGGTTGGGGGAGGTCTTGGGTTCAAGACAGCCCATT

TGTATTTGTTCCCTGTTTGCATTGTGCTTTCTATTTATGTATGTCAGCTT

AAGGTTTTGGAATTATTGGTGATACATCGCATCATATCTATGAATAGAGA

TTTTTTAAATTTTATTTTGCAAATCACTAGTTAATGTATGCAACAAAATT

GTTGTTTTACGTAAAGTACCCGCCACTATTAGACAGACTGTATTGTCTGA

TTCAAAGGTGGATTTTAGTTTCTATATATTTTTTCATTATGTTTCCAGTT

CACTGCAATTCAAGTAACATGGTTCTTGTTTTGTGTTGTGGCTTTTTAAA

TGTTGTAGTTGCATTCAGAAGCAGGGAAAGGTCAGTTTGAATTGGCATTG

GGATACACTCTTTGTTCTAATGCTGCAGACAACTTGATTTTCACTCGTGA

AGTTGTTAGGTCTGTTGCAAGGAAACATGGGTTGCTGGCAACTTTCATGC

CAAAGTAAGTGTATGATTTTTTATGACATGGGTCCAATTTTTTAAATGGA

ATTCAGCTAGAACTAAACTAGGGTATGGAGTTGGGAACAGATTCTATGTA

TAATGTATTTGTAAGAATTGTTCATAGTGGTTTCACCTCTCTTTCAGTGT

ATCAATGTTACTGTATATCTTTGGATGAACGGTGAGGAGAGTGTTCTATC

CGTATGATGATATAAGAAGTATGAGCAACATTATTCTTTTGGATTAAAGG

GGAAAAAATGAATATCTTTGTTTATTGTTTGTATTGGGCAAACATTTTCC

TTTGCCTTGTATTTTATTGTTTCATCAGTAATCATCTACACATGTCATAT

CCTTTATGTACCCTATATGTCAAACCTACCACGCTAAATGACCGTGGGAT

TTTTCCTGTATCATTTCCGAGAGCTAGTGGAGGAGATTGTTCTATCCGTA

TGATGATATAAGAAGTATGATCAACATTATTCTTTTGGATTAAAGGGGAA

AAAATGAATATCTTTGTTTATTGTTTGTATTGGGCAAACATTTTCCTTTG

CCTTGTATTTTATTGTTTCATCAGTAATCATCTACATTTATGTCATATCC

TTTATGTACCCTATATGTCAAACCTACCACGCTAAATGACCGTGGGATTT

TTCCTGTATCATTTCCGAGAGCTAGTGTGATGACTGATTGAATCGTTCTA

AGGTTTCTGCACTATACCATATTTGACTGTTTTTTGTTAAATATGATTTT

GCAAGAGGGTAAATATGTGATTCCACTTGTTCTGTTAACGTCATTAGGCC

AACATTTTTGACAAGGGGTGTTAGTGTTATTTTAGAAACCTGAAGGGTGT

CCTTGTCAAAAACTTCAAGGGTGTTAGTGTAATTTACCCAGAAATTTTTT

TTAGAAAAAAGACCTCAAGGGTAAGAAATACTGCTTTGAAGTTATAATTA

ATTGTAAAAAAAATAAAATTCAGTTTATTATTTATGTTTTGTTGTAATAG

TTCATAAATACTCTGCAAAAGAATCCGTCAGTGTTAAAAATACTCACTCA

AAGTTGAACTTTACTCATTTCCTCACCTGCAGAATTTTCTTCTTCAAGCT

ATGTCTCTTAACTGTTTTCTCCATTTTTTTGTTTGCCTCAGGTATGCATT

AGATGATGTTGGTTCTGGATCTCATGTGCATCTCAGTTTGTGGGAAAATG

GGAAAAATGTATTTATGGCATCTGGTGGACACTCTAAGCATGGAATGTCC

AAGGTTGGAGAAGAGTTCATGGCAGGGGTTTTAAATCATCTTCCTTCAAT

TCTGGCCTTTACGGCACCAATTCCAAACAGGTTTTTAATTTTTTCTTATA

ATTTTGATGATTCTTGTGCAAAACATGTGTCAGAGTTGATTGAACCCATA

TGAATAGGTAGTTAGGTACTCACAGAGAAGTATGCATTTTTACCAGTTCT

GTTAAAATATCTTGTCTACATAAAATTATTTTTTAAGCATGCGGTTATAT

TTGGAAATTTGATGGGCGTACTATTGATCTTGAGGAAAACCTTTCAAGGT

GATGTTGTTTCCATCACTTTCTGTTCTGCATGAAGAAAGCATATATCTCA

CAATAATTTCTGGAGCCACTATTCCGCAAAGACTTTTTCCCAGTTAATCA

TTTGAATTGAAACAAACAGATCCTTGCATTTCTAAGTCTCCCTGCAGACT

TGAATTTATTTATTTTTGTGCATGGACCTTTTTCTAATCTGGAAGTTTTT

ATGTAGAACTCGTTGGCCTAGAAACATAAAAAGACAGATAAGATGTTTGA

AATTTTGAATGAAATTCAATTTTTGATGAATTGAAGGATTTCAATTTTCA

GCAGAATATGTAACTTATTTTTTAAAAAATACATGTATTCTTTTCCTATT

CTTATCATAGGTTGTCTGTGTGATATGGTCTCGTGTAAAACTCAATCCCA

ATAGCTAGCTTGTAAAGTGAGGGTGCACTTTTGACACTTATTCTAATTGT

CGATGTTCCCTTTTTGCTAAATTTAAATAATGCCCTGCTGTGCAAATTCT

TTTTGCCCATTTCTTCACTTCCAAATTTATGCAGTTTGAAGGATCAAAAT

TTTAGTACGTTGTTGGGTTTTTGAACTTCAAATAGATGTGTATTAAATTA

ACAAAAAATAATATAGTTTGTTTTCTCTAATTTCCTTGTTCAATTGTTAT

TGGAATGTAGTTATGATCGCATAGTGCCCAATATGTGGAGTGGAGCATAC

CAGTGCTGGGGAAAAGAGAACAGAGAAGCACCCTTGAGAACCGCATGCCC

ACCTGGGGTTCCAAATGGTGTTGTGAGCAACTTTGAGATTAAAGCATTTG

ATGGGTGTGCAAATCCACACTTGGGGCTGGCTGCTATAATTGCTGCTGGG

ATTGATGGCCTTCGTAGACATCTGAGTCTGCCCGAACCCATTGGTAAGTA

GGATTATATGATTGTACTATACCCAAAGTGTCACATCGATTATTTGTTAG

TGTTTTTTCTGTGCATTGTTTCTGAATATTTTTTTAGACATACCATCCAT

AAGTCCCCCATGTTTCATGTCCTTACGGCCCTGGCACTTACCCAACAAAA

AAAAAAAATCCTTTATGAATTATGTGAAAGAGTTCAGATTCAACTGTATC

ATTCCTCTGGCAGTGTTCATGCCAAATATTCATACTTGGTTTGATATGCA

TTAGTTCATGGTATGTGCTCTGATGTTTCCAAAAACATAAAAAGTGTTTT

CTCTTTTCTGGGGGATTTGGTCAAAACCCCCTCATAATAAAACTTTAATG

TGTTAAGGACAAGCACAAAAAGTTTTCTTTGAAAAGTTCAACAAATATTA

CTATTATCAAAATATTAAACTTAAAAATAATATAACTTTTGATGCAGATG

TCAATTTTTAATAAAATTAATATTTTTAGAATTCTCTCATTAAGATCTAT

GAAATAAAATCCATGTTGACTATGAAATTCTCTATAAAAAAAAAATGATT

TCCGTTGAAATCGATGTAAACAATAACAGTAATATGTTACTTTGTCTTAT

AGTAACATGTTACTCTATTTGACAGTAACATGTTACTCTGGCAATATTAT

CAAAGAAAAAAAAAAAAAAAATTTACGTTGTCCTTATTCAATTATATGTT

TTGTCCTTGGGGGGTTTATGAAGAAAAATAAATTGGGCTGTCCTTTTCAC

CAAACAGAGGTCCAATGAAGGGGTTTTGACTAAATTTTCCCTCTTTTTTC

TTCCTCAGCAATCTTTATGTCGAATCTTCCAAGGGTCCAAGCCCATCTAG

GCCCTTTGATTCCAAATTTTTAACTGTATCTCAGATGCATTTACTACAAT

TTGGAGTCTAATATGAGTTCTATTCTCATTCCTCTCAATCCGGGTGCTTT

GGTATTGTTAAATTGTAGGACTCATTTTTCTGACATATCACTAGTGTGCA

TATTTTATAATGGAACTGTATCTGTATTGGTCACAACACCTCTCAAAACT

CCCACATGTACGACATCTCCTAAACAAGTCCATTGCTCAAATGGGAATTT

TCTCAATTTCACATGCTGGAAATGCTTATATAATTCTATTAATTCCGCAA

ATCGACAGGCCTTAGGATGTTGAAAAGATAAGTAATTAATTATTTTTCCA

AATCGTAGGCATTTCATAGTTGATTTTATTTCATTGTCCTGTATATACTA

AAGAGCACCTTCAGTTGTGTGTGTTATTTTAACAATGATTCCTTCCTCAC

CTAACCTTTTATGGCTTTTTTCTGCATTCACTTACCAATATTTTCATTCA

AGTAGATTATTATATAGTGATGAACAGTATTGGGTATATATATATATATA

TATATGCTTCATTTCTGCACATTGAATAAAGTTTTGTATTCCTATCTGAC

ATTTTAATGATATTTTTTGGTGTGACAGATACAAACCCTCATAGCCTTGG

CACAGAAATTAAACGATTGCCAGAGTCTCTTTCAGAATCTGTAGAAGCTC

TTGACAAAGACGGTATCTTTAAAGATCTGATAGGTGAAAAGCTTTTGGTC

GCTATAAGAGGAATTCGCAAGGTATGATCCAAATAGAATTTCTCAAACGT

GTTCTTGAGGGATATTAGTAATGAATTCAAGCAAAGAAGGAAACGCCACA

GTTTTTCTAAAACCTGCTTAAATGCCTTATTTTTATGGAAAATGATATGC

TTACCAAAATTCATAAAAGGAATGATGTTGGTACATCATTCTCGAACAAG

AAGGAATTCAGTTTTTAAAAAAGTTACCCTTCTAGAACTACCTGGAATGT

GCAGTAAACTTTTTTTTACATAACTAACGATGATATGTAATGTTCTAATC

ATCAAACCCAAATCTGAACAGTTATCCAATTTAAAGTACATCAGAAGGCT

TAAATTACATTACGAAGGGGCTACTCTGGAGTTTAATCTGAAAAAATTCC

ATTTTCTGTTCTAAAATTAAGCAAGCAAGGATGGTGACACAATGTTCTGT

TTTGCAGGCGGAGATTGCATTCTACTCGGAAAACAAAGATGCATACAAGC

AACTTATACATCGCTATTGAGAGTAAGAGACGTGATGATTCCTTTTTGTG

TACTATTGCTTTTTGAAGATGTGATAATGGTTATAGCCACAAAGGGATTA

GTTAAAGTAACGGAAATAATAACAGCAGGTACGCATGTCGCAACCTGTGG

AGTATGTTGTTGTTGTTCTTATATTACTGGCTACTGTTTTTGATTTCTTT

GGTCCTTTCTCTTTCAATAATGTCAGTCCGAGTGTTATATTTTGTACACT

TCTTTTTCCAGCAGCAAAAATAATAAAGTTCATGGTGGCTATAAGCTTTT

TTC

>TEA020018.1 locus=Scaffold1219:1345177:1349058:+ aquaporin PIP1-3-like

ATTGTGAGTGGCATGGACATCATTTCTCTTACCTTTTTTAAAAAAAAAAA

AAAAATTAAATTTTTTTAATTTTTTTTCCAATGCAAAATAATGAGTTGAA

CCCTATTATAAAACAACCCAAACACTCCTGTGTCACCACTTCCGCAAAGC

CTACTTTTCCCAGTATCCAATCTGTGAAAGAAAAAGAGAGACAGAGAGCT

TTGCAGAGAGACCATGGAGGGTAAGGAAGAGGATGTGAAGCTAGGAGCCA

ACAAGTTCTCAGAGAGGCAGCCACTGGGCACAGCAGCTCAGACAGACAAA

GACTACAAGGAGCCACCACCAGCACCATTGTTTGAGCCAGGGGAGCTAGT

GTCTTGGTCATTTTACAGGGCTGGGATTGCTGAGTTCATGGCCACTTTCC

TCTTCTTGTACATCACCCTTTTGACTGTTATGGGTGTTGTTAGGGCACCC

AACAAGTGTGCCTCTGTGGGTATTCAAGGAATTGCTTGGGCCTTTGGTGG

TATGATCTTTGCCCTTGTCTACTGCACTGCTGGTATCTCAGGTTAGCACC

AACTTTGCTTTCCAAAAAAGTTTTTTTTGTTTTTTTTTTTGGTGGGTGAA

TTTTTAAGATGGGTTTATGCGATCTTTTCGGTCTTTATTCAAACTTTTTG

TTTGAAATCTTGATTTGACCTGAAATAGTGAACAAATTCAGATGTAAGTA

CAAATATGAGATAGTGGTTTTTTTTTAGTAAGCTCTTGATTTGGGTAAGA

AATTTGACGGATTAAAGAATAAAAAATTAGTGTAGACAGATTAAAATATT

TTTTAAAAAATTGACATAATTTTTTTAAAAAATTCATTAAAAACAAAAGT

AGAAAACATAAGTCAGCTTAAGCTATCCCAAATTCTTTTTCCTTTGTGTA

TCTTCTTCTTCTCCTTTTAGATTTCTTAGATCTACTGTACTCACTTGAAT

CTGGATCACTAAAAAAAAACTATTTCAAAATTGTTAAGTGGAGATGGATG

TCAATAAGTAAAAATATTGATGAAATAATTTCAAAGAATTCACTGAAAAG

GAAAATAAACCTGCTTATAATCTGTGCCAAATGTGTACTTTCTTCTTCTG

TTTAGAATGGGTTTATGCGATCTTTTCGGTCTTTATTCAAACTTTTTGTT

TGAAATCTTGATTTGACCTGAAATAGTGAACAAATTCAGATGTAAGTACA

AATCTGAGATAGTGGTTTTTTTTTAGTAAGCTCTTGATTTGGGTAAGAAA

TTTGACGGATTAAAGAATAAAAAATTAGTGTAGACAGATTAAAATATTTT

TTAAAAAAATTGACATAATTTTTTTTAAAAATTCATTAAAAACAAAGGTA

GAAAACATAAGTCAGCTTAAGCTATGCCAAATTCTTTTTCCTTTGTGTAT

CTTCTTCTTCTCCTTTTAGATTTCTTAGATCTACTGTACTCACTTGAATC

TGGATCACTAAAAAAAAACTATTTCAAAATTGTTAAGTGGAGATGGATGT

CAATAAGTAAAAATATTGATGAAATAATTTCAAAGAATTCACTGAAAAGG

AAAATAAACCTGCTTATAATCTGTGCCAAATGTGTACTTTCTTCTTCTGT

TTAGATTTGTTAGATCTTCTCTTTGCTACTCATTTGAACCTGGATCACTA

ATTTATTTTCCTTTTTTTTTTTGATAAATAATTTATTTTCCTTTTTTTAA

GGTAAAATTAATGGGTTTTTCTTAGTTTTTGTTTTTCTGCACTCTAATGG

ATCACTATTTTGTAATTTGTGTTTTTATGGGTATTTATTAGTTAAAGTTT

GTGGATTTGTGTGTGTATGTGTTGGTTGAGCCACACATATTTGATCATCA

TGTGTGCTTTTTTCCCTTTGGATTGGATGATTTGATGAGAAAAGAAAAGA

AAAAGGAGGACTCTCCCTGATCAACTATGATGAGGGAAATAGAGAAATGA

AAGGAAACCAAAATCCTTGTAGCTCTTTTTCTTTTCCTTTCCATCTTTGA

CAAACAAGGGTGAGGATGAATTACTATTATCTTTCTTAATCCCTTCTAAA

TTATCCCAAAAAAAGCCTATTTTCTTCTCTTTTCTCTCCTAAATAATCCA

AAAACAATCCAAACAAGAAGGAAAGATAGATACCACTGTCATTTTCTTTT

TCTTTACCTAAGCCTGTCTAATATTCTCTTCTGGTTCCAAGATTAGCTCC

ATACATCGTATATTTTGCTTGCTTAATTGTGTCTGTAACTGCAATTTTTT

TTAGTCAAAAGTACTTCTCTGATCAAACTAAACTTGTTGGTGACAGGAGG

ACACATTAACCCAGCTGTGACCTTTGGATTGTTTCTAGCAAGAAAGCTCT

CTCTCACCAGAGCTGTCTTCTACATGGTGATGCAATGCCTTGGTGCCATA

TGTGGTGCTGGTGTTGTCAAGGGCTTCCAGCCTTCCTATTATCAGATGAA

CAATGGTGGGGCCAACTTTGTGCAACATGGCTACACCAAGGGTGATGGTC

TTGGTGCTGAGATTGTTGGCACCTTTGTCCTCGTCTACACCGTCTTCTCT

GCCACCGATGCCAAGCGAAGCGCTCGAGACTCGCATGTCCCCGTACGTGT

TCTCTCTAATAAATTTACTTGAACTAAGTTCTTGTCTATTATTTGGATTC

AATTTTACAGGATCTGACTTATATGTTTGCTTGATTTGGTTGTGTCTTTT

GTGTGGATAAAAGTATGTGTTAATTATTAATGATCCTTGCTCAGCAATTA

TTCCCCGGCCTATAGTATAAACGTTTGGGGCTGGGATCATCCTTGCTCTG

TCCTCATTCTCTGCCTTTGTCTCACCCTTATAGTGTTCATCACAATTTGA

ATCCAATGCAATTTATTTATTTTTTAAATTACTAAGGGACTTTGTGGTCC

TACAAAGGGTTTTGACAGAGAAAGGGAACAACAGGATCCAAAGCCAAACT

TTTGTTCCTTTATGGGAAAAAAAAAATTTAGTTACATGATTGCATATACT

ATTCATTGAATTTTGCTCAGGGGTCAGGATCCTCTGCCCCGCTGTTCATA

TGACACCACATGTACATAAAAAAAATCAACCCAAGTGCACCCAAATTCGT

CCCCTCTCAAAATGGGCATACAATGCAATAGCAACAAATAAGTTGGGTCA

TCATCAACACCAAGAACACATACCGATTGAAAATTTAATCATTTTCAGCT

TTTAAAACTCCATCAAAATTTGTTTTCAGTTATTTGGAGCAAAATTTTTC

AAAACCCAGACAAAAAATATTACACAAATAGAGATGACAACACAGAATTT

TGGGTTTTTGCCCGATGAACACATGGCAGCATAAAAGAGCTGGGGGATAG

AAAACTGGGACAGATGATCCCAGCCCGTGCTCAGACCCCTGTATTTTACT

ATATTTTGTTAATTTATTTGACAAAAGTCGACCCTGAAGGCATTGATTTG

TATATGTGTAATGGTGAGGGGAGTTTTCAGTAGTGTGACCATAGGGTACT

CTCAAGCTTTAGACATCCATTTCATAGTTTGTTTTGGGAGAATAACTTAT

ATTGTGTGAAACTGCAGTGTGGTTTTTTTTTATTTTATTTTTGTGTGAGA

TTTTACTTGTTTTTAAAACCTTGATACAAACTTAATCAGGTGTTAATTGA

GTAAAAAAAACAATGTTGTTTTAATTTTTCCATTTTTGCAAATATAATGC

AGATTTTGGCACCTCTTCCCATTGGGTTCGCTGTGTTCTTGGTTCACTTA

GCCACCATCCCCATCACCGGAACTGGCATTAACCCAGCCAGGAGTCTTGG

AGCTGCCATCGTCTTCAACAGGGACCTTGCATGGGATGATCAAGTCAGTA

CACCCATGATTGAGATCACTATACATTATTAA

>TEA001162.1 locus=Scaffold1290:679604:690563:- probable WRKY transcription factor 57

TCTTTCTCTCTCTCAATCTCTCATCACTCGTCACTCATCACTCATAACTC

ATCATCAGTGTTTTCTGTGTGCTCTGTATCGACGAGATAAAGCTTTCATT

CTCTCATCACTCATCATCAGCGTTTTCTGTGTGCTCTGTATCGACGAGAT

AAAAGAAAGAAAGAAACAAAAGAGTTTGAGAAACTGAACACACTTCGCGT

ACGCGTCCTTTCCTTATCCCAAAACCCTAACTTTATCTCTCTACTTTCTC

TCTCCGATTCCTCCCAATTTATCTCTATATATGTGTGTGTATATATAGAT

AGATCTTCCAGAGGGTAATGGATGAGAACGACAGAGTCGATCCAGGAATT

ACAGATTTATCGGCAGAGTCGAGCTGGTCGTTCGGCGGTGACTCGGAGAG

CGTTTACTTGTTCGGAGGTAGCGACAGAGAGAGCAGCATACTCAGCGAAT

TCGGCTGGAATCTCCGGCCAGAGGCAGGTAGTTTTTCTCACTTTGATCGG

ATCGGTATGGAGGAGGATTTGGCCGGAAATGATCAGCCGAAAAGTACTTG

TTCTACATCTACCACTGGTGGTGGTGGGACGGTGGTTCAAGCGACGTCGA

ATCGGTCGATGTCTTCGAGCTCCTCCGAGGATCTGCCGGAGAAGTCGACG

GAGTCCGACGGGAACAAAGCAGCGCCCGAGACAGCGTGAGTGTGTGTGTC

TTTAGCGTTGTTGCGTTTGTGGTTTGGTGTTGCGTGAAGACTACTCCATT

CCGTTCCGTCACCACGGCTAGTATCGTAATAAATCCGGCATTTCTTGGGC

TAATTCACCGGCTCTTTCTTACTCCATCCAAAAAATCACCCTAAATTTTA

GCTAAATTTATTGTTAAACAATGCCATCTCCGTGTCCGTACTGATTTTTT

TTCTCTCTCTCTCTCTCTAGAAGGTGTGGGGGCCCAGAGAGCAAATTCAG

CAAAATAAGGGGGTAGAGGAGGGATGAATCCCGCCGTTATTGCGGCGGAT

ATGGTGTGCTGTATTTTTTTTTTTTTTTTGAATGCTAAACATACTTTTGA

AAATCACATAAACTGATAGTTGTTGCATTTGTTACATGAGTGAGAGAAAA

GGAGAAAAGGAAAAAAGTTAGAGTATATGTAAAATCTAACTTTTTTCTCT

CTTAAATTCAATTCTAGATGAAATACTTTTTTGAGCATATCTACATTAGA

TTTCTTTTCGTGCATTTACATCTTCCTCGTGATTTTTTGTCTGTAATAAA

AATCTACTAAAAATTGAAGTTAATGATATATACTTTTCTTAAAGCTGTTT

GGTGGTATGTTTGTTGTTAGGTTTCTTCATCATTTCATGCTCCTTAACTT

GAAATGGTTCTTTTTTATGTTTGTCAGTTCTCTTTTAATTTGTTTTTTCT

TGAGTTGTTTCTGGATGTGGTGAATGTTTTCAAAAACCATGTAAGGGCTA

AGTAGTGAACAAGTCCTCCTCCCAATTAGAGTATTTTATGCCATCTCTCT

CTCTCTCTCTCTCTCTCTCTCTCTCTCTCTAGCTAATAGAGAGATTGTGT

TTACTTACAAAAGGAAAATGGAGGCATTAATTTGAAAAGAAATCCTGTCA

TGAGTAACAAATAGCTATCCCAAATGCTGTGCAGGTTTTGTCAGTGAAGT

GTTGATTCATCTAATCAGATCCTTTATCACTCCCCATTGGCCTTGAGCTA

TTTTCAGTCTGCACCACTTGCACCACTCACTCACAGGAGGACTTGTATAA

AATTTTGTCTGATTTTTTGTAGTTTGTTTGTTATGATTAATAATTGATTT

CCCTCTTTGAGTATTTAATTTATGTTTTTGGTTCTTTTTCTCTAGTTGTC

AGACCTGAGTGGAAATTTTATATTCTTCACTTCTTTGGGGATATAATAGC

TGAGTTATTTATATTCTTCACTTCTTTGGAGATATGATAGTTGAGTTAAA

ATGTCTTTCTATTGAGCTTTCCTTAGCTTTTGGTTTAGGTAGTGTATGGT

TTTACTGTAATGATTGTAAACAGAAATGAATTGGCCAGTTTTTGACCTTC

ATATGGATTGTTCATTCTTCTAAATTGAATGATAATTCTATGGGGGAATT

ACTTGAGAGTTGGAGAAAAAATTTCAAATATACGGGGTACAAGTGTGTCT

ATGGTGATCTGTAAATAAAAGGCTGTGAGAGTACAAGTGTCACTTGTACC

CTGTATCCCTATATATGTTAGTAATTTTTTTTCCTAACTCCCACCTACAT

GTAATTTTCCCTAATTATATTTTCAATGTAAGTTCATTTTCTTTCTATGG

AATCAAACATGAAAATAGATATTCTAACAAATTGCCATTTTATGCCAATG

GCCTTGTGGGCTAGTGGCATGGGTGCAACACTTAAAGTGCTTGCATGGGT

GAGGCTGGGTTCAACTATCAACTAAGTTTCAATGATCAAACCCCCCAAAA

AAAAAAAAAAGGCCATTTTATTCCAACTTTATTTAACTCAACCAAAAATT

GTATTGGCTAAAGTTTATGTATGTAGCTTAATCCTCTTGTGCCCTTACTT

TCCTGCAATATGAGTGACAAATATTCCATTCTAACAACATTTTTTAAGCA

ATTTCTTTCTTGTTGTTATCCTTTTGTTTTGTTTTCTATAATTCTCAAAG

TCTTTCTTTGTTTCTTTGACTTTTGTTTTTGTTTTTTTTTTTTCTTTTTT

CTTTTTCTTCCCGAGTTGTTGTTCACCACAACCCTAAAATGAATGTTAAT

TGAATACTCTGACAAGTATTACAAACACTTATTCATTTATTACTAAGGCA

CTAGATTGTCTTGCATGAATCCACTGGATTGTCTTGCTTGAATACATGCA

GATTTGTTTTTCTTTTGATTAAATGCGCTCCTATTCATAATTTAGGACTT

GCTTTGACTATGTTTTTTCAGTCTTTTTCAGAAATTTGCTTTGACATTGA

TGACCTTGGACTAAAATAATTTATAGGAGCAAAGTAAAAAAGAAGGGGCA

AAAGAGAATCCAACAGCCACGTTTTGCATTCATGACTAGGAGCGAAGTTG

ATCATCTTGAAGATGGTTATCGATGGCGGAAATATGGCCAGAAAGCAGTT

AAAAATAGTCCATTTCCTAGGTATGTGTTCACTTCTCTCTAAGGATTACA

CTTGTCAAGATCGATTTTTTCATTTTTAAGTTGACATCATTTCCCGTATA

ATATTGGTTTGTTTTAGAGCAAAATATTGTCAAATATATTACCGTCACAT

GTCTCTATGTCTGTGTAAGGGATTTAGATATCTGTCTATCCCTATTGTTA

TTTTATTAAGGACTAGATACGAGGCGAGTTGAGTTTGAATATGTATGAAA

TTTTTTATACTTTCATGTACTGCAATTTCTTAAAGCATAGCAAAAATTAA

TATCTCTTATTAATTTTGTTTTGGATTTTTTTTTTAATTTTAAAATTATT

AATATCCTTGCATTCAATATCTGACCCATTTTACCATAGCATGCTCTTAT

TTCGGATGGTTTAATTTTTACACTGATTTTTTAGTCTGACCGACAGGTAC

CTCTCAAAGTTTACGAGTGTGTATTTATATGATATTCTGAAATATTTTGC

TTAAGCAAGTTGCACCAAATACCATTTATTGGTGTTGGACTCTTTTTTCT

TAAAACGCAAAATTCAAGGATACAAATCACTGTTTACCTTTCTATACATA

TAGCAGAGTGGCAAAAAATGATATAAGTGATGGAGATTCAAGAATCTGAA

ACATCAAACAAGTATTATGTAGTGGAAGTGAATTCTCAGTCCCCTGAAGA

TGTTTCTGGCATAAATGATGGTTAGTTAGATTGAAATCACAAGAAAAAGA

AGACTAATACTAATTGATCGCATTCCTTAATGCATTTTGCAGGAGCTATT

ATCGGTGTACAAACAGCAAATGCACAGTGAAGAAAAGGGTTGAACGCTCT

TCGGATGATCCAACTATTGTAATCACTACATACGAAGGCCAGCATTGTCA

CCATACAGTTGGATTCCCCAGAGGTGGAGGTGGAGGTGGTGGACTCGTCA

ATCATGAAGCTGCCTTCGCAAGCCAATTGACTCCTTCAGCTTCACATTTT

TATTATTCAGGACTAGGAGGACTCCAATTTCCTCCACAACCTCCTCCTCT

CGGTATTTTACCACAATCATCATCTCATCAAGTTCCAGGTGAAGCAAGAG

AAAATCCTAAGCTTCCCACTGATGAAGGACTTCTCGGTGACATTGTACCT

CCTGGAATGCGCAATCAATGACAGCAAGTTGTTGGTATGCACTAAACTAA

TTCACCACAGCTCCTCTTATTTTCGGAGGAGGATCCCCTACAGTTTTTGG

ATTGGAGGGTATTGTTGTGACCCATAGGATTTACGGATCCCGTTTTGATA

ATTCAAATCATTCATTTTGTCAGGTATTTCATATATTTCATTCACAAAAA

GCTTGGATAGATCTGAAAACTGTAAGTAGTTCAATTTATTATGTTTGTCA

TCAAATAAATGTATAATTTAATTTTTTTTGTCCTTTTTTTTTTTTAAATC

TAGAAAATTGAACTAGTTATAGTGTCATCAAATCGACATAATTTTTTTTG

TGTATAAAATACACGAAATACCTGACAAAATAAATGATTTGAATCACCAA

AACGGGATCCATGATAAACCCTATAAGTCGGAACATCGCCTCCTCCTGTC

TAGATTTTGGACTGGAGGGATCCTCCTCTCTTATTTTGATCTTCTATTCA

CTTTCCTTTTAGGGGTTTTCTTAATAAAAGCTATTTTACACAAGAATCAT

TTGATATTCAACATAGAAACTTAGGTCTTAAACAGATGATTTATTAATGA

AAATATTAAAGAGTTGTGTTTTTTAAAAGAAGCTGACAGTAGCCGCAGAA

AAGTTACAAATCCTAAAGGTAAATTGCACTTTATCCTCCTAAAGTTTGAT

TCATTTTCTATTTTAATTTTTAAGTTTAAATTTTTTTTATTTTTTTATTT

TGGCTATTCATATTTACAATCTTTTTCACTTTTACCTTTTATATTTACAA

TCTTTCCTATTTTGACTCTTTAAGTTTGATTTTTTGTACGTTAACTCTTC

ATGTTTGCAATCATTCTCACTTTAGCCCTTCATGTTTTGCAACCCTTATC

ACTTTATTCCTAAACTAATTTCCATAAAAAAAAAAATAACAAAATCTGTC

ATATCATCAACACATCAGCTTTATATGTGATGTAAAAAATTCACTAAAGA

TGTAAAAAAATAAAAATTAAAAACAAAAACATTGAAATTTTTTTTGTCTT

CAGTGAATTTTTTTCAAATTTATTGATAAACATTATTGCATTATAGATGC

ATCAAGCTCAGATGCACTTAAAAAAATAAAAACAAATTTAATTAAAAAAA

AAGTTCACTAAAGATGAAAAAAAAAACGATAGTAAACTTTTCATTTTAGA

ATGGACAATACAGATGTTTGACTTATTATATTTTTATTTTTTTCGTTTTT

AGTAAACTTTTTTCTTTTTTTAATTAATTTGTTTTTATTTTTTTAGGTGC

TTCTAAGCTAGATGCATTTATAATGTAATAATATTTATGAATAAATTTGA

AAAAAAAATGTTCACTAAAAACTAGGCTCTGTTTGGTAGAGCTTTGAAAC

GTTAGTTCCAGGTTTTGATTTGCACATCGTGTTTGTTTAAAGTTTTCAAA

AATTAATTCCACTCACTTTTGAAAACTCTGTTGAAGACGAAAAAATTTGG

ATTAACTGCTCATTCGGCCGGTTGACTGGCACAGTGTTTTAGTATTATTA

TTAGTTTTTATTTTTTTTTTATTTTTTGCAATTGACACATGTTACTTGTT

TTATATTCAATAAAAAATATTTTTCGAATTAAATATTTCTTTATGAGTTT

TGAATAGAATTTTGAGTTGTGTGTTTGGTTATGAGTTGTTAATTTTTGTA

TCAAATATCATATGTCACATCAAAAACTCCCTACTCAATTCAAAATTCTT

TTCAAAACTCATATACCAAACAAATTTTCCATAATCAAATCATAATTTCA

AAACTCATAAATTCAAAATACAATTCTATTCTCAAATCATATTTAGAAAA

CTCTACCAAACGCAATCTAAATTTTTATTTATTATTTTTTTGTTTTTTAT

TTTTTATTTTTTTGCATCTTCAGTGAATTTTTTGCATCACACATGGAGCT

GATGTGTTGGTGATATGGCAGATTCTGTTAGTTTTGGGGATAAAAGTTAG

TCTAAGAGTAAAGTGAGAAAAATTGTAAACATGAAAGGCTAAAGTAGGAA

TGATTGCAAATATGAAGAGTCAAAGTGAGTAAAATTAAGCTTAAAGGGCC

AAAGTGAGAAGGATTGTAAACAAGAAAGGATAAAATGAGAAAAATCAAAT

TTAAAAGGCCAAAATAAAAAATGAGTGAAACTTTAGAGGAACAAAGTGTA

ATTTACCCAATCCTAAATTATAGTCTAAATTTGGGTATGAAACACCTGAA

ATCAACAAGAAAGCAATAGATTTCCCTGTACTGCATTTGGGTTCACGCGG

TCGTTCCTCCTTCAACTTCCACTATTATGACATGCAGCGATTATACTATT

TATTGTTTATTTATTTGTTTTATAAGCAAAGGAAAGAGATTATATTGATA

AAGAAGAAATATAAAATGAAGATTTGATGCAATTGCATTATAGTTAAAAA

ATAATATCCCAACTAAATTGGTATCTATTAAAAATCCAATCATATCAGCA

ATTATACTAATAATGTTGTTTTTTCTTTATATAATTTTTCCAAACAAAAA

ACATCATATGTACTAGGGCGCCTTGAGATTTCTTAAGATACGGATTACCC

AATAGTTTCAAAAATTACAAATATTTCATTGTCAGAATTTTTTATTCAAC

ATAATTAACTAAAAAATAAAATTTCTAAATATTTTGTTATACAATGATAA

AAATACCTGAATTATAAACTGAAATAAATTTCTTGTATCATTAAAAAATG

TTACTTTTTTTTTTATCAGCAAAAAGAAACTTTATTGATGAAGAAAGCAA

TATACATGCGAAAAACTTACATCAATAAAGAAGAAAAGTGCAAATAGCAT

CATAACTAACCTTACCAATTTCAACTAGGCTGGTACCAATAACGTAGCAC

AATCATTTGCTAGCACTTAAAAAATGTTACTTGATTTAATTATTTATTAA

TAAAAGGGGTCATTTCTTATAAAATCAAAAATTTCAAGAGATGTGATGTA

ATTTCTAAAACTACGGAAGCAAATATCAAAAAGTGTATTTGTAATTTACA

TAAATACTATCTCTCTTGTACTGGGATTTCAGGATTTCATATTGTAGACC

ACAGTCTAAAGGCTCAAATATTAGCCCCTTTGACAAATTAAGAAGAAGCC

CACTAGCGTGCGTGAATGTTTTTTTTAATTATTTTTTGTCTTTTAATATT

ATTGTTTAAGTTCAAAAATTAGATTCACTTTTTAAACTCAATAATGCAGT

ACTCTATTAAAATATTTAAGTCAATTCTCTCTCTTATTTATTTTTCAATT

ATCAATCAATTATTTCATTGATAAATCATTAGATCTAGAAAGCTAGTTTG

TATATTATTATTAATATTTTTTTAAGGGTTTTACTAGCTGCCTGTAGTGT

ACTACTTCAAGACAAGTTATGAGAGCGCCACTCTGACCCATTTTGAACTT

GGTAATAAGTATACAGATTTGGTATGCCATTATCTTAAATTTGATATTTA

CGCATTGTGTTCTTGGTTCCTCGTGGTCCTGCATTATGATTATTATAAAC

AAAAAAAAGTGTAGTTCTCATTGGTTGTGAACTTTTTGTTTTTTTTTGGA

TAGGCATAAAAAAACTTTATTGATTACTTCAATATAAAACAAAGATGAAA

TCAATAAAGAAGATGTGATGTAAGGATCATCATAGCTCTCAAACTAATCC

CAACTAAGTTGGCACCCACAATCAAAAAAGCTAGCATACACCAAGTTGGT

AACGAACCAACAAGCAGAGAGAGGACAAAGAAAAGGGAAAAAAAAAACAC

AAGGAAAAAGACAAAACAATCCAGCTGCATTTTAGTTCCAAATTTACTGT

TAGACTAGTACTACTTATTTGCTTGCTTTGTCATGCTTGCTAGTATTTAT

AATGGCTTTTGTAGGGTTGGGTGCACTTGGAAGTAGCTTCAATAAAATAA

TGATCCCTAATGGCCAGTAGAATATTTTAGGGGAAATTGCAATTCATTGT

ATTTTTGTTTTTTGTTTTGTTTTGTTTTTTTTTTTTTATAATCAAATTTC

AGGTCAAGGTAAATTTTCAGATCAGTGGACATGTAGTGTGAATTACATGG

AACATCTGGAGCTCCTGTTTTAGATGTATGCTAATTAATAGAGACAAGAA

TGCTAAAATGACCCCCTTTTTTTAATCATAATTCGTATACCAACTAATGT

GTCAATATTTAATACGTCACCACTAGTGCATGAAAGGGGGGAAAAAAAAT

TTGATAATTATAGCATTGTTGTAATAGAAAAGTCCACTTTTACAAAATTT

ATGATTGCAATGATATGGGATATATTTGAAAATTTAACTCACTTTGTTGG

ATTAAAATAAGAGACTCATTATAATAAGAGAAACACCAGTCAAATGAGCT

CTTTGCTGCTGAGGCTTTCTTCTAGGTGTTGATCTGTTGCTCTCCCTTGT

GCTTTTTGGGTTGGTGTCTTACCAACTGCGAGTGGTGCTAGCTAATGCAT

GGTAATGCTTCTCGGTGCCAACCTAGTTGGAATCGGGTTGTCGTTTTATT

GATGATTGTCAATGTATTTTGCTTGATCATTATCAATAAAATTTCTTTTT

GGCTATTTCAACAAAAAAAAAAGGAAAAAAAAAAGAAAAAAAGAAAAAGA

AAAAAGAGCTAGGTCTAATCTTTCCACAAAAGTTTAGAGCTGAAGTATAT

TTTGAAGTTCATTCAGAAGTCACTTTTCAATGAATTTGTTTCCTACCCTA

CCACACCATTTTTGGCGGGTTACTTTACTTGTGCCCTAAAAGATTAAAGT

CTCTCTCTCTTTTTTCTTTTTGAATAATCAGGTGTCCGGACTAGTTACCC

TTATACTAGTTTTGTAATATCTGTTTGGCTTAGAGTCAATTTGAGAGTAT

CATGAGTGGAATTGTCATTCATCAGATTATATTTTCCACATTCAATGTTA

ATGGCTGAAATTGAAAGGGACTTTTCATCACCATTTAGTAGAGTAATATT

CATATTGTCATCATGAAAGATGGTTCTCTTGATTACCAGTGAGTATTATT

ACATGCATGAGGCTATGTTTGGGTTTTGAATATGATACGATAGGATATGA

AGGAGCATAACGTATCTCCATCATTTTTGAGATACTTGAAAGAAAAAAAG

TAACAAAGAAAAGCATATTTTCGCACTCAGCACACACTTTTGAGGTGACC

ATCCTCCTCTCCTCACCACCAATTTTCGAAAGAGAAAAATATTTGTAATC

TTGCATTATGCTTTGCTTTGCATTTCAATGAATATTATCTCTCGTGCATA

ACATTCAGACCTGATTGAATAAAGCATGCCCTTTAGGTGTAGGTCCACCA

TTTATCACGCCTTTGATTGTAACTTATGCTAAGCTATATATACCCTATAT

ATCTTTCCTATTATTCTCTCTTTAGTCACTATCCTACCAACAATGTTTCA

TATATATATATATAAACAAAATATATGAATTTAAAATATGGGATTCGATT

CATTTTCAATATAACCTACACTATAATAAATTTAAATTTATATCCATAAA

TGATGGATATAGAAATGAGATACGGATATAACATAATACATATATGCTTA

TATAAACAAATTGCCTCTTTGCTTTGTTTTGTTCTGTGGGTTCTTTGCTT

TGGATTGGTCCCTCACCAATGCCCTTGCATTGCCCTGCCTTGTTGATGTT

TTTGCATGTTTTATGCTTTTTCTCATCAATAAAGCTTTTATTTTGCTGAT

CAAAAAAAAATAAAGCATGCCCTTTAGGTGTAGGTCCACCATTTATCACG

CCTTTGATTGTAACTTATGCTAAGCTATATATGCCCTATATATCTTTCCT

ATTATTCTCTCTTTAGTCACTATCCTACCAACAATGTTTCATATATATAT

ATATAAACAAAATATATGAATTTAAAATATGGGATTCGATTCATTTTCAA

TATAACCTACACTATAATAAATTTAAATTTATATCCATAAATGATGGATA

TAGAAATGAGATACGGATATAACATAATGCATATATGCTGATATAAACAA

ATTTCAAAAAATATTATACAAGTATGGTTAGAATACATTAAAAAATAGTA

ATTTTTAGTATTATATTATACATGAATTCAAAGAATGTTGATCAAGTGTT

TTTTAATAATTAAACATTAATCTAAGAAAATAGTCAATTTTTTTCTAATG

AATAGGTTAATTTGACAAAATTTGAAAAAAATAAATAAACATATTTGAGA

ATGATTTTTTATTTTTTTTTATAAAAAGATGTGTAAAAATTATAATGAAG

AAAAATAAAATTTTGATTTTTTTTCGTATAAGCTGAAAGTTTGTTGACTT

TTTATGTAGGCTAAAATGGTGAGTTCTCAAATGGCCCAAGTGGGATAACA

TTTCAATTTTGGTTTTTGTTGGACAATTGAAATTTACATATGTACATGCC

ATGCAGGAGTGAAGCAGACGACTTCCTGTAAACACTATTGTTGTGTGGAC

TGGAGAAGCTAAAACATGACCACAGGGCTACAGCTGCTATTTTTGACCCG

GATGTGAGTCAGTTTTTTAACCTGTGAGGGTGAAACCCTTTTCCTTCATA

TCAGTCAGTTCAAGTGGCGTGTAAGTACACTAATGTGAGCTACTCACATC

ATGTGTCTCAATATTCTTGCATTGAACTTACATTTATTTTATCTCCACTA

TAAAACCATG

>TEA022960.1 locus=Scaffold1341:4830581:4834542:+ Anthocyanidin reductase

TCACTTACAACCAAATAAAAAGCTTTATTAAATTGTTTTGGATTGTGGGT

ATCAAGATCTTCATCTTCTTCATCCAATTCTTGCTTTGATCTTTATAAAA

AGTGAAGCCATGGAAGCCCAACCGACAGCTCCGAAGGCCGCATGTGTTGT

TGGTGGCACCGGCTTCGTGGCGGCGACGCTCATCAAGTTGTTGCTTGAGA

AAGGCTATGCGGTCAACACCACTGTCCGCGACCCAGGTTAGTCTGGAATT

CAATTTTGTTCACACCTACAATAACAATTAGATTAAGATATAATTTTTGA

TTGATTTAAAAATAGTAGGTACTATTTAAGTTTGAGTGGGTCGAAATATT

TTGAATAATGGCCTATTATATTTGGTGAAGTCAAAGAATATAGTAATAGA

TTACATCGCATAATAAGTCCTCGTTTGGAAATAAATTTTTTTTAGTAATG

TTTTATAATTTTTGATTAAAATGTGATGTAAAAAATTAATTTGTTTGAAA

TAATTTTTTAATTTTTTATAAAAAGATATGTAAAAAGTAAAGTGAAAAAG

TACTCTTTGATTTTCTGGTATGAATTGTTGACTTTTTGATTTTTTTATTA

ATTTTTTAATTAAAAAAATCACTTACCAAATGAGACTTGAGAGAAGGTTA

TATCTGATTTTATAATTGATAACTGTTATTAATTGTAGACTATTTATAAA

TTATATTTTGTTTATACACTTATTAGTCAGTGTCAGGAGAGAGAAAAATC

ATTTTCAGTTGTTCTCTTCCTCCCATTTCATAGATTCGGAGTACCTGCAA

TTTTTATTTATTTTATTTTGCTTTTGTCCAAGTGATCATTTTGGCACTTT

TCATTTATTCCCTCAGCTGGCTATTTAAAGAGTCTTTTTTTTTTTTCATT

TCAAATTTGTTATTATTATTTATTCTAATATATATATATATTTTGCGCGA

CTAAAAAAGACTGAGAAACCAAACAAAATATCACCTGCCCAACTATTCTT

CGCACAAATGTAGTAGTTGACTCGTCCTCACTATGTTCTTTTAATTTGTA

CTTTTTCAAATATTTTAATTGTGGATAAATTACAAACGCACATATACGGT

TTATTTATAATATAAATTATCGGTTGTAATTTTGAAAATTAACAAGATTT

TTTTATTAGAATTTTTTATTTAACAAAATTAACCAAAAAGAATTCTTAAT

TCCTCTGCTATAAAACAATAAAATTATTTGAATTATTAATTGAATATATT

TTTGACATTATTAAAAGCTATTCATTGACTTGATGATTTATTAATAAAAT

GCGTTATTTGTTATGCGATTAGAAAGTTGAAGAGGTGAGATGTAATTTTA

AAAAATTATAAGGGCCAATGTGTATTTTAAACAAATTTCAGTAGTGGTAA

ATTAATTACAATGATCACTTTTGAGATTTATAAGAAATACAAATAGTTTT

ATAATTTTGATATAACTATACAAATTTTCTTTTTAATAAGATTAAAAATG

TTAAAACTATTACAAATAATATGAAGGTAGAAGAAATAAAGAACTTATAT

ACATTTCAACGTAAAACAATTGTGAAAGTTACAATATTTTTTTAAACCTG

TAAGTTCAGTATATTCTATCCAAAACTTTATTATTCGCGGCCTTATTTGT

CTAATTTATAAAAATTTGTATGTTTGCCCAACATTTTTCCATTACATGTT

GTGAAGATAATAGAAGTGTGGGCCTTCAAAAAATTTAGGAAAAATTATAT

ATCAGTTATGCTTTTCCAAAATAATTGCATATTCATAGAAGGTTTATAAA

TCTGAAATATTTTGTAAAAGTACTATTGATATATAATTTTCCCAATTTCT

TCTACAAGGGCCATCTTTTGGTGAATGAAGGTCATTAAGTTTTGAAATTA

TTATTTAAAAAATAATATTTATTACCAGATGAATTTCACATATTGAACGG

TCCTTCTCAAACCATAACCCTAGCCTACCTAGCCTGGCTCGGCCCGAACC

ACCTACCAACTATTTCTAGGATAGGAATGGTTGGTGGCAAATTGATTGCA

TATAAATTTTTGGAGTCGGTTGGGTGGGAAAAAAAAATAATAAATGTTAA

CCCAACCTGTGAGCACACCTAGTTGCAATATGGTGGCAAAAGTCTAATTG

ACTATTTTATCCCAAGGAATTTGGACATGAATTTTATTTATTTATTTTTT

CACTCTCACGCAGGCAATCAGAAAAAGACCTCTCACCTTCTAGCACTAAA

GGGTTCAGGCAACCTAAAAATCTTCCGAGCAGACCTCACCGATGAACAGA

GCTTTGACACCCCTGTAGCGGGTTGTGACCTGGTCTTCCATGTCGCTACA

CCGGTCAACTTTGCTTCCGAGGATCCAGAGGCAATTTGCATTGTCATTAA

CAATAACAATTGTGACAACAAATTTTTATTTTTTTGACAAATTAACATGG

TTTTATCTCATTTTTAATGTAGAATGACATGATAAAACCAGCAATTCAAG

GAGTAGTCAATGTTCTAAAAGCTTGTGCAAAAGCAGGAACGGTTAAACGT

GTCATTTTAACATCATCAGCAGCTGCTGTATCGATCAATAAGCTCAATGG

GACCGGCCTGGTCATGGATGAGAGTCACTGGACTGACACCGAGTTTTTGA

ATTCTGCGAAGCCGCCCACTTGGGTAAAATTGTGTCAAATTTTTAAAATT

TTTGGATTTTGAATGTGTATTTTCTAGATGATCTGGAAATAAATTATGTG

AATTTGCAGGGGTACCCTTTATCGAAAACACTAGCTGAGAAAGCTGCTTG

GAAGTTTGCCGAAGAAAATAACATTAATCTTATCACTGTCATCCCAACTC

TCATGGCCGGTCCGTCACTTACTGCAGATGTCCCTAGCAGTATTGGTCTT

GCCATGTCCTTGATCACAGGTATTAATTCAGAACCTGAAGTTAACAAATT

GTTGTTAACGACTTGAACTTTGAACCATAGTTTTAAATAGCGGTATCAGC

CAGTGTAATCATCTGAGTCAGCCAAGAACTGATGAATTTTTTTCGAACAT

TAGCAAAAAAAAAAAAAAAAAAAAAAAAAACTCTTTGATGAGCATGTTCT

AACCTGATTTGTTACAATTTAAATGTTTGTAATGATTTTTAATCAACATC

ATAGCACCTAAAACACGTTTTTGCAATAAATACATGTAGAAAGGTATTTG

AACTATTTACAATAACTGGAAATGCTACTAAAAATAAAATTGATAATCCA

TAACAACAAAACAAAACATAATTCAACATATGTTGGTAGTACAATGATGC

ACATACTTATTACACTTCAAATTTTCGACATTGTGCAGGGAATGAATTTC

TCATAAATGGGTTGAAAGGTATGCAAATGCTGTCAGGCTCAATCTCCATC

TCCCACGTGGAGGACGTTTGTCGTGCCCACGTGTTTGTGGCAGAGAAAGA

ATCGGCCTCTGGTCGATACATTTGTTGCGCTGTCAATACCAGTGTTCCCG

AGCTAGCCAAGTTCCTCAACAAAAGATATCCAGAGTACAATGTCCCTACT

GAGTAAGGCCCCTTCTCTATACAACATATTCGAGTCTTATCTGTTGCGTG

ATGCAGTTTTCCCTTCTCTCGGGAAATTTCCCATCATATATTAGCATCAA

TTTTCAAAACTGTCTAGTTATTTGAACTTATTTTCGCTTGGTTGATTGTA

GTTTTGGAGATTTTCCATCAAAAGCGAAGTTGATCCTCTCGTCTGAGAAG

CTTACCAAAGAGGGATTCAGTTTCAAGTATGGGATCGAAGAAATTTACGA

TCAATCTGTGGAGTACTTCAAGGCTAAGGGGATTTTGAAGAATTGAAAGC

ATTAATAGTGTACCTATTTTGGCTATTTAAAAAAAAGGCTGTGTTATTAT

TGTTCACTTGACGATGTGGTTTTGCTTGGTTTGCTTGTATGCATAAATAT

ATAATAATTAAC

>TEA008904.1 locus=Scaffold1440:945752:946615:- probable aquaporin PIP2-5 ATGGCGAAGGATATGGGGGTGGCGGAGCAGGGGTCTTACTCCGCCAAGGA

CTACCAGGATCCGCCACCGGCACCGCTAATCGACGTGGAGGAGCTGACCA

AGTGGTTGTTTTACAGGGCTTTGATTGCGGAGTTCATTGCCACGCTTTTG

TTCCTTTACATTACGATTTTGACGGTGATTGGTTACAAGAGCCAGGTTAA

CCCGGTCAAGAATGCTGACCAGTGTGGCGGCGCTGGGATTCTCGGCATAG

CTTGGGCTTTTGGTGGCATGATCTTTGTGCTTGTTTACTGCACTGCTGGA

ATTTCTGGAGGACACATAAACCCAGCAGTGACATTTGGGCTGTTCTTGGC

TCGGAAAGTGTCGCTGATTCGAGCAGTGATGTACATGGTGGCTCAGTGCT

TGGGTGCCATATGCGGAGTTGCGCTTGTGAAGGCCTTCCAGAGCTCCTAC

TACGACGTCTACGGCGGTGATGCTAACGAGCTCTCCACCGGCTACAGCAA

GGGCACCGGCTTGGGCGCCGAGATCATCGGAACATTCGTGCTCGTCTACA

CCGTCTTCTCCGCCACCGATCCCAAGAGGAGCGCCAGAGACTCCCATGTC

CCTGTATTGGCACCACTTCCAATTGGGTTCGCTGTGTTCATGGTTCACCT

AGCCACCATTTCGGTCACCGGCACTAGCATCAACCCTGCTAGGAGCCTTG

GAGCTGCTGTTATTTACAACCAAGAAAAGGCTTGGCATGACCATTGGATG

TTTTGGGTAGGACCCTTCATTGGTGCAGCCATTGCAGCCTTCTACCACCA

GTTCATCTTGAGAGCTGGAGCCATTAAAGCTCTCTGGTCTTTCAGGAGCA

GCTCCCATGTCTGA

>TEA024031.1 locus=Scaffold1471:1691728:1725195:- ferredoxin-dependent glutamate synthase-like

CGAGACGAGAGGAGGAAATAGTAGATGCCAGAGGAGAGGCGGGTGGTTCC

GGACCTCTCTCTTTTTTATCTCCACATCCATTTCCTCCCCTCTCCACACG

ATCCTCTCTCTCTCTCTCTAAAACTCTCTCTCTCTCTCTAGAGAGAGATT

GTGTTGTGTTGTGTTGTGTTGTGGCAAGGGCAAAACGACGCCATGAAAAA

ATGTACCGCGAAACAGGAAGTAGGGCTGAGTGGTGCGTAAGCGTATGTCA

AAAACTACACATCAGCACATTAACATCAACTACGAAGGCGAGACGAGAGG

AGGAAATAGTAGATGCCAGAGGAGAGGCGGGTGGTTCCGGACCTCTCTCT

TTTTTATCTCCACATCCATTTCCTCCCCTCTCCACACGATCCTCTCTCTC

TCTCTCTAAAACTCTCTCTCTCAAGAGAGAGATTGTGTTGTGTTGTGTTG

TGGTGTGGCATTCATTGATCAACAACTATGTCTGTGCAGTCAGTGCCTCA

ACTTCTCTACTCCAATGGCCAGTCTCTCAAACCTCCTTTTTCTTCTTCTT

CTTCTTCTTCAGCCTTCGCTTCTAACAGAGGCGGCCTCTTGTTCGTCGAT

TTCGTCGGTCTCTGCTGCAGCTCCTCCAGGCAAACTTCACGGAAGAGACT

CGGAGCTTCTTCGACTACTCAGAGGTTTCACGGTTTACCAGCTAAGAACT

GGTCTTCAATCAGAGCTGTTCTCGATCTTCAACGCCTCCATAATTCATCG

GAACAGTCCTCGGATTCCAGACCAAAGGTCTTGTTCATTTATTTACTAGT

AATTGTGCTGCAATGAATTTGTGTGTGAATTTACTGTTTTTTTTTTTGTT

TGTTTGTTTTAAATGACTGTTTATTTTGTAGCTAAGTTTTAAGTATGTTG

CATTTTGAAATAATTTGCTTGATTTTGGAAATTTATTTTGGTATTTCGCA

TTTGTGAACAATCGAATGCTTTGTGAAATTGTGATGCTGTTTGGCAGGAG

TATAGTTTCTATTTTTTATTTTTCTCTCTTATAATGTAAATGGACCAAGA

TTCGAGTTCACTGCGGGCTGGATTTTGATTTTGAAGGGAGTTGATTGTTC

CAATTATAAGGATAGTTTTTTTTTTTTAATTTATTTATTTTAAATATAAA

GATGAGCAAGATTGTTCCAATTATAAGGATAGTTTTTTTTTTTTAAATTT

ATTTTAAATTTTTTTATTTTAAATATAAAGAAGAGCAATTTGAGTCTGCA

ATTTTTTTTTTCGTCAATCGTTAAGGATTAATACAAATAGACTGTATATG

TACTGAACTAGCATAGTAGCATCGTGTTTAAATTGTTATAGAGCTGTAGC

TATTGACTAGAATTTGTAGTGGAACTGCCAAAGCAATTCTAACTCTATTA

TGGGTTGACTTATGCTGGAGTTGGGTGGGTTGCTACAGGCTGGTGGTCCC

CAAAAGACTGCTATTTGTTTGTGCAACCTTTTATTATCTAGAACACTGTT

GTTGTTGAAGACAGGCGTTTCCTCCTCCACTCCCTCTTGGTCTTCTTCAC

TGCCAAGATTATTTATTTTATTTAAAGAACCAAAAGAAAGGAGATGCCCT

TTGCACACGCATTGTGCTCTACATAAAGTAACCTACACTATTGACACAAA

AGGCATATCGAAAACACTTTCTTTAATTAGAAGATACACTATATAGCCTA

ACATTTAACCTGATGTGATACCTCAAAATGAGAGAATATACTAATTTTGT

TAGCATTGCTTTAGCAACTTGCTATTGATTGTTTTCTTTGTTTTGTTACT

TATCAAAAAGAAAAAAAGAAGGTTATCTTTGATTTGTTTACTTTCTATTT

TAGTGCTTTTAGGCTATTATTTTTGTCCATTTATGTCATGTGATAAGGTT

GTGACTACAATTAAATCATTTGCAAGTATTGCATCAAGGGTCACTTTTGA

TTCCATACATATTTACTTTAGTCATGGATGAATTAACTAAACACGATTAG

GATGATGTCCCATGATGTATATTGGTTGCATCTGATATTGTTCTATTAAA

TGAAACTAAGGAAGGAATTAACAAAAAATTAAAAATTTGGAGAGAAGCAC

TCGAGTTGAAAGGATTTAAAATTTGTAGAAATAAAAAGGGAATGTAAATT

TCTTGACTGTAGAAGTAGAAATAATGGACAAGTGAAGATTGATAACAAAT

AGTGAATTTTTCGTTATATAGGTTCGGTTATTAATAAGGGCAGTGAGGAT

GGAGATGATGTGATCCATAGAATCAAAGTTGGTTGGGTCAACTGGAGGAG

TACATTTGGCGTATTGTCTGATCGATGAATACTAACAAATTGAATGAAAT

TTTTTATAAAACAAAACAACTATAAGGCTAATAGTGCTTTACAAGACAAA

ATGTTAGGCAACTAAAAAAACAACACAGTGCTTTACAAGACAAAATGTTG

GGCAACTAAAAAAACAACACATTTATTAAAAAAAAGCATAGTTAAGATGA

AAATGCTTGGATGGATGTGTGGCAAGATTGAAAAAGATAGAATTAGAAAC

GAATATATTCGTGAAATAGTAAGAGTAGCACTAATTGAAGATAACTTGAG

GGAAAACACACCTAAAATGGGTTGGTCATGTACAACGAAGATCTATAGAT

GCAACGGTAAGGAAAAGTGTTATGATCACCATAAATAGAAATGTTGGGGA

AAGGGAAAGACCTAAATTGGCATGGGATGCGCTAATGAAAAAAGAACATG

AATTTATTTAATTTAACTAAGCATATATCCCTTGACAGCGCAAAATAGCA

AAAAATGATTCATGTCTAGACAATTGATTGGGACAGAGGACTTAGTTTGG

TTATGTCATGCATCACTGACACACAACTAGTGGTAGTTAGATTTAAGGCA

ATTTGTCGAGATATCATTTGATACTTGATTAAATTACGTGATACTTTGTT

ATTTTGGTAAGCAACTTTTATTCAAAATTTTCCATGTTAGTTCAAATGCA

TATTTGGCGGCACATCTAAGTATTGGTCAATATTGAAAACCATTCATCCC

AAAACATAAATGGATTAATTGTGATAAAAATTATGTGATGCCATTAAGTG

TTTCATGCATCCCTTGCCAATCAAAAGAAAGTGTTGCATGCATCCCTTCC

CCCCCAAGACATTCTATCCGTCGACGTCCAATGATGGCTTTATCATTATT

TAAAAGGTCCATCAGTATTGTATTGTTGTCGACCTCCTTACAGTAATGTC

ACTATTTTTTTTTTTCTCTTTTATGGGATGTTGTTTTCTGCTATGATTTT

AAATGTCTGTTCTTTTCATTTCTCTATTATGCATGTTTCGATTGTGATAT

GAGTGTTCTATCTATGAGTTTCTACGTGCTTTGATGAACTGGGTTGTATG

CTTTTCAGGTTGCAATCTTGGATGATATAATATCAGAGAGGGGGGCCTGT

GGAGTTGGATTTATTGCCAATTTGGAAAATAAAGCCTCACATCAGATTAT

TAAGGATGCTCTTACTGCTCTTGGTTGCATGGAACATCGTGGTGGTTGTG

GAGCAGATAATGATTCTGGTGACGGTTCAGGATTGATGACGTCAATTCCA

TGGGATCTTTTTAACAATTGGGCCAACAAGCAAGGGATTGCTTTCTTCGA

TAAGTTGCACACTGGTATTGGAATGGTTTTTCTTCCCAAAGATGACAACC

TTATGAAAGAAGCCAAATCAGGTAGCTTTAAAGAGCTTTGTGCAATTCAA

ATCTTGCAATTTTTCTTTCGTTTATGAAAATAATAGAATGTATACAATTC

TTTAAATAGTTGTTCGTGCATGATGAACAATAGCATTTATGATATTTTTA

AAGATACTTCTTTTGAAAGGATATAATTGGTACTGTTATGTTATCCACAT

CCATGTGTAAGTATAGACTTGCATTTAAAGTGAATAAATGATATTATTGG

GAGAATTTTATTTATTTTGTTTTTATTTTGTAAGAAGGTTAAATGCCTGA

CTGAGTGAAACATAATGGATGATAAGGAGCATGATTCAAACTTACTGTTG

ATGCACCTAGCTGGCATAACAATAGCCTGGACATTGTAAATTTACTATTT

ACAAAAATTGAAACTTTTGGTAGGAAATTAATTAAGAGTATTTAGGGGGT

TAGGCATAGGAGTTGGGTTGTGTTCCCTTCTTGTGGAATGTCAGGGGTGT

TTTAATTATGTGGGATGATAGGGTGTTGACTGAGGCTGTAGTATTAGTAG

GAGCTTTTATGGTTTCAGTTAGGTTTGCAATAATGATGGAAGTGAATGAT

GGTCATTGGGAGTGTATGGACCCAATAGATGTTGAGAAAGTCTTTGTGGG

ATGAGTTTAGTAGCCTTTTTTTGGGTTATGCTATCCTAACTGGTGTTTGG

GCATTGATTTTAATGTTAATAAGAGTGATTTCAAAAAAAACCTAATGGCA

GGAAAGTCTCAAGACATGTGAAAGATTTTGATGCTTTTATTAGTGATTGC

GACTTAATTGGTCCTCTTTTGTTTGATGTTTATTTTACTAGGTCAAATTT

TTAGGAAAACTTGGATAAATTTCTATTTTCTTTGGTGTTGGAAGAAAAAT

TTCCGAATTGAAATAGGAGGTACTTCCTAGAGTACTTTTGTTTCATTGTG

CGGTGGTTATGGAAAGTTGTCCTTTTAAATAGGGGCCTACTTCATTTAGA

TTTGAAAATGCGTGGTTAGATCATCATTTGTTTAAAATGTGTTTTCAAGA

GTGGTGGGAAAATGGTAGGGTTCAAGAGTGGGAAGGTTTCAAATTTATGA

AGAAACTTGATTTAGTCAAGGGAAAGTTGTAAGTTTGGAATAGGGAGGTT

TTTGTTGAATTTGACGAAGATAAAAAGTGAGATTACTAGAAGGATTGAAG

AATTGGATAGATTAGAGGCTAGTGGTGGATTCAATACTCCTTTAGTAGGG

GAAAGAACAAAGTTGAAAATTTCCAGGCGTGATGCCTGAGTTTGATGACG

TGCTCCCTTACCAAGTGTAATGCCTAGGTTCTTTAAGACCAAGGGGTTGG

CCCTTGGATTCGAGTCTTACTGCTGCGAATGCAGTTTTGCTTCCCTATTC

CCCTGCTGCAGATTGTAAAAATTAAAATTAAAAACCAGGCCGTCTTTCTG

GATTTTTGTAAACCATTCCTGTGACTTGAACCTGCTTCACAAGGCTTGGG

CTGGATACACATGTTACGGTCAGTAAGTGCTGCTATATAAGTATCAAAAT

AAAGAAGTGGCAGATATATGGTTTTATGTGATTATAATTCTGTAATTTTC

TTTTGCTTTATCAGTTATCAATCTCCATGTGATTATTTTTATGGACGTAT

ATATGTTGGCATATGTTTGAATTTGTTTTTTTTGGATTTTTATATCACTT

GTGGTCCTATTGTGCTATATTTTGTCAGAATACTTTCATCCTCTTTTATT

GTTTGATGATCTCTTAGTTCTAGTTCTAGTCATTCCTGTTTATTTTTTTG

TACTTGTATTCAGTTTTCTAAATGTGGAAATAATTGTTTATGTAGCTATT

ATTAGCATCTTTAGACAAGAGGGTCTTGAGGTGCTTGGATGGAGGCCTGT

TCCTGTAGATACTTCTGTCGTTGGTTACTATGCAAGAGAAACCATGCCCA

ACATACAGCAGGTTTTTGTTAGAGTTGTTAAAGATGAAAATGTAGATGAT

TTTGAGAGAGAATTGTATATTTGCCGGAAATTGATTGAAAGAGCAGCCAG

CTCAGAAGCTTGGGGAAATCAGCTTTATTTCTGTTCTTTGTCCAATCAAA

CAATCGTTTACAAGGGAATGCTTCGTTCAGAAGTTCTTGGTAGATTCTAC

TTTGACCTCCAAAGTGATCTCTATAAATCTCCTTTTGCTATTTATCATCG

GAGGTACAGCACAAATACTAGTCCTAGGTGGCCTCTTGCTCAACCAATGA

GGTTACTTGGTCATAATGGAGAGATCAATACCATACAGGTTACAGTTTCT

CTATTCATATGCTGTAGCTTGCCTAATAGAGTGTTACTTCGTATTCCTTT

GTTTTGATGCCGAAGCCTTTATGCAGGGAAACTTGAATTGGATGCAATCT

CGAGAGACCTCATTGAAATCTCCTGTTTGGTGTGGTCGGGAAAATGAAAT

TCGTCCTTATGGAAACTCAAAAGCATCTGATTCTGCAAATCTTGACAGTG

CAGCGGAAGTATGGGCTATTGCTTCTTTATTCATATAATCTGCATATTTG

CTGCTGCTACGTGTTTTCTGGAATGCTTCTTATAAATCGACTCACTGAAA

ATAATGAAGAAAAAATTTAATTGGATTTCACATACAAATCTTATGCTTAA

CTGAAGTATCCAGGTCTGGATTTCACACACAAATCTCTATTGTGAGATTT

GTTTCTAGTATGGCTGGCTGCTAAATGGCTTTTGAGATCCTTGAAGCAAT

TCAGAATTTGTTTTCTGAATAACACTAAAATCTAGTTTGGTGAAAAAATG

ATGAAAACTTCAAAAACACTCATAATTTTCTTAATTGTTTTCTTTCCTTT

TTTGTTTTTATGTGTCTGTGTGTGTGCGCTTGCGTGCATGTTTGCACAAA

ATAGTCTTTGAAAATTCAAATACATCTTTTCGGTATTTGTTTTCATCAAA

CTAGTTTTTATTATTTTTCAGAGAACATATTCTGAAAATGAAACCAAATA

GAGTGAATAGATTTCATGGGAAAAAAACAAAGAAGAAATCCTTGTTTGCT

ATGGTCTTTTAGTGATGGGAACTGATGCAGTCGATAAGAGGCTGAAGGAG

CAGATTTGGTGAGAAAAATCTTAAAAATAAGTTAAAATACAAGGTATGGC

CTACTTGGGGTTTCTACTAATCAAGCCTTCTGTTATAGTTTGGCCCTATT

TTATGTCTTGTTTAGATCATGAAGTAGTGCTTACATGGCAGCCATGTCAC

TCTTGCGTGGATGCCATGTTGGTATAGTGGGCAGTCAGTGGCGGACCCAG

AAATCTTGTTAAGAAGGGGCCAACATTTATATAATCATAAATTGATTATA

AATTTTATTTTATCACATTCTTACAAAAGGGTTTTTTTTTTTTTTTTTTT

TAAATTAAATTTTTTTTTTAAACATATATTGTACTAAAATATTTACTTTT

TATAAGTATAAAAATTAATATTATCATTATCTTTACAAATAATATTTTTA

GTCAATAGACATTTACAAAACAAAAAGATTGGAAACAAGATTATAAACTT

CATTTACACATTGTACAAAACATAAGACCAAAAAAAGAAAAACATACAAG

TAATTTTTTACATTAATCATAGTCACATTGGGTCATAAACCAAAACCAAT

TACAGCTATAATAGAAGGTCATTTGGGTTATAATCAAAGTCACTACAATG

AGTTGCAACGAATACCACTTTGAGACATAATCAAAATCATAATGAGATGT

AATTAGTTTTAATTTAATAGTTTTCAAGCTACACACTTATAACCAAAACA

ATATCAATTGATAAAAAATATCAGTTTAGACTACAATTTATGTAATAGCA

TGACTCTAGTAATGGTTCAAATAAATTGGCTCTTTCGAGAACCTCAAGTG

GTTGGCCTAGTGGGCATGACTTGGGGCTTGGGGGGCGTGCTCCCTCCAGG

TCTCAGGTTCAATACTCACTAGTTGCAATAAGCTTCTTGGGGCCAGTCTA

TGCAGGGCATTTTCTTTGGTTTTAAATGGGGCCCTGCAAGTGGGTGGTGG

GATTGGTCCACTTGGATTAGTCAAGGTGCATGTAAGTTGGCCCGAACACC

CAGTTATCAAAGAAAAACAGGCTCTTATGAGGGAATAAAAGAAAAAAGTA

CTGAGGGTTGGGGGGGCCAACTCTTAAGAAATTATGGGTTCAACAAGAAT

ATATACGATGCTATGGAAGGTAATGGAAATGGATGCAGGGGCAGGGGCAG

TGACAGTTGCAGTGGCCACCCCTAGCCAGCCATTGCGTCTGCCACTGTGG

GCGGTCAAAGAGTCTCTTCGACTCGGCATAAATTGGAAGCATCTTTTTCA

TTTATGATTGTCTAATAGGTTATGTTTTGAACGAATGTCGCCCTTTTGTG

TTTTTGATAAAGTTGGCAGTAGTGAGAGTCCTTTGTTTTTTTGATGAGGT

TAGAATTTGTGGATGTAATTAATGATTTAGAAGAGTGCAAGTATAAATAT

TATTTTGGAAGATGGGGTGTAGCTATTGACTATTATAGAGCTATGCTTTT

TTCCTACTCTTCTCTTTGTTGTGATTTCTTGTTTATCAACCTTCTTTCTT

CCTCCCTCTTATTCACCCACATCCAATCATAATACTGTGTTTTTGTTGGG

TTTTTATGATTTTTTGAGTCCAATGTATTTGAGGAGATATATATTCTGTC

AAGCTCAGCTGTAGTAGACTCAGTCATAGTGCGACTCTGCTTCTACTCAT

GCCCAACAATTATAAAATTACCAAAATACCCCTAACCACTAAAACCTAAT

ATAATGACATAAAATTTATCTAGATACTAAATTAAAATAAGCACAAATAA

GAACTCTAAATCATTCCAAAACTTCACTCCATGATATCTTAAACGTGCTT

CATGTGAAATTGCAGATTTGTTCTTTTTATTCTTTCCATGCTCGTCATCA

CTGTCTCAGTTTGCTTTGGGTGGGTAGGGGGTTATGTATGCTGCAACTCA

TTTATCTATTTGTACTTTTTTGTTCATTAATATGAGACTGCTTCAGACGG

TCCATCCATTTAATGTTCTGATTCATTATTTGAATGTTTGACACAGCTTG

TTTGTCTGACTAGTCCTTCCCTTTTGTACTTTTCAGTTTTTAATAAAAAG

TGGCCGTAGTCCTGAGGAAGCTCTAATGATTCTTGTCCCAGAGGCTTACA

AGAATCATCCAACTTTGTCGATTAAGTATCCTGAGGTAATTTATGTTAGC

AATTCAAAATTAAAAAGCATCAATCAATATATGCTTTTGGCAAAGGTCCT

TTGCTTTAGTGCATTGAGATTTTTTTTTAGGGCTTAAACATGGCTATTCT

TAAGTGTACTACTTTGTAATTTAAGTTCATAGGATGTATTGTTATTCTCA

ACCTGAATCCATCTATTATGTCTGACTTCTGAACTGTTTAGTTACTGTGA

CCTTATTGAAATTCATTTGATATTCCTTGCGAAGGATACTCAGTATTTCT

CTGTTCTTAGGTTGTTGATTTTTATAACTATTACAAGGGTCAGATGGAGG

CTTGGGACGGACCTGCTTTACTCTTATTTAGGTAATACTTCCAAGGTCAC

TTATTGCCATTTTATGGTTACACAGTGATTGTATTACTGAATTCTGTGAC

TAAGAAAAGCAAATAACTGAAAATTTCAAAATCCTAATGCAAATTAAATA

ATGATGATATGGGCTAATTCCAAGTGTTTTAAAGGGAATTGGAGTTGCAA

TAATCAATATGAAATTGGCAATTGATAAGTTCTCAGAGTTATGGGGTGTT

GGGGAATGGCTAGCATACTAGATCACACTGGAGATAGTGAAAATTTTAGT

TAGGTAATACTTCAACTTCTAGATGGCTTGATATACAGTATTGTAACTGA

TGCTGTGGGTAATCTACAGGTTCCCTCATATATGATATAACCTTGAAAGT

GGGCCCAGTTTTATACGTAATTATGGTATCCAAATGCTTTGGTTTTTTGT

TTCATCGATGTCAGTTAGCTATTTTAGAGGCTGCTTGGCCTAATGCTAAA

TGTTTGCCATTAACATCTTTGGGTCACGGAAACAATTTCTCTAGAAATGG

GGGTAAGACTACATACATCTCGCCTCCCCAAACTCCGCTTTAGCGAAAGT

CTCTTGCATTGGGTCACCTTTTTTATATATATATATATAATGTTTGATGG

CTAGTTTTTCTTCTTTTCTGTAAAAATTATCCGGAAGATGGATTTCCTAT

GTGAACAATCTTAATGTGTGGTTGCATTTTTCGCTTGTTAATTTTTGGAT

AATTTTTTTTACACCACTGCATTTTTGTCTTTTCTTTCTTTGTTTGTGAT

GCCACAGTGATGGAAAAACGGTTGGAGCTTGTCTTGATCGGAATGGACTT

CGCCCTGCTAGATATTGGCGTACCATAGACAATGTTGTCTATGTTGCATC

TGAGGTGTGTTCTGTTTTCTTTATCACAACATTTTTTTTGTCTTAAACTG

TAGACACTTACAAGAGTTGGATAAGATCATGCATAACTCTGGTTTAGGTT

TTTTCCCTGAGGCTTGGCTTAACTTGCAATTCTTAGGAGCTTGAGGAACT

GAGCTTGAATGCCACTTTCCCTGCATATGTGATCCGAAGGTTTAGAGAAG

AGAAAAAGTTAGAGAGTGAGTTAGACCTCATTCCCTTTTATATTAATAAT

ACTGAGTAATTATAGGAGTGCCATAAGGCATAATATATGGGCCAACTTAT

TACACCAATCAAGGAATAAATAAGAAGAAAATACAATCACATATTTCCTA

TTCTAACTCGTTGCCTATTCTAACACTCCCCCTCAAGTTGGTGCAAAGAT

ATCTCTCTTGCTCAGTTTGTTGAGAATGTCATGAAATGGTTTGCTCCCTA

GCTCTTTGGTGAGAATATCGGCTAGTTGGTCTCCAGTCTTCACAAATAGA

ATACATATGATGCCACTAGTGAGTTTTTCTTTGATGAAGTGCGTATCAAT

CTCCATATGCTTAGTTTTATCATGTTGAACTGGATTATGAGTTATGTTGA

TTACGACCTTGTTGTCACAGTATAGTTGCATGGAGTCAGTCTGATGGATT

ACCAGGTCTCCCAATAGTAGTTTAATCCATAGTAACTCACATACATCCTG

AGCCATAGTCCTGAATTCTGCTGCAGTACTAGATCTAGCCACCACATTCT

GTTTCTTACTTCGCCACGTGACTAGGTTGCCACCCACAAATGTGCAGTAT

TCAGAAGTTGATCGTTTGTCAGTGACTGATCTAGCCCAATCTGCATCAGT

AAATGCCTCCACACTGAGATGGCCATGTTTTGAGAAATAGAGACCCTTTC

CAAGAGAAGACTTCAGGTATCTCAGGATTCTCATAACTGCCTCCAAGTGG

GAAGTTCTAGGAGTATGCATAAACTGGCTCACCATGCTCACAGTAATGGC

AATGTTGGGACGTGAGTGGGATAAGTATATGAGCTTGCCCACTAGTCTCT

GGTATCTCCTCCTGTCCACTGGATCTCCCTCTTGATTCTCACTCAGTTTC

TGGCCCGGTTCTAGAGGCGTATCACATGGTCTACTTCCAAGTATGCTAGT

TTCCTTTTGCAAGTCAAATACATATTTCCTCTGACATATGAAGATGCCAT

CCTTGGACCGAGCTACTTCAATACCCAAAAAATACTTCAAATAGCCTAAG

TCTTTGATTTCAAACTCTTTGGCTAAGTATTCTTTCAAACGAGAAATCTC

CTCAATATCATCTCCTGTCAGAACAATATCATCAACATAGACAATAAGAG

CTATGGTCTTACCATGTGAGGAGTGTTTCACAAACAAGGTGTTATTAGCT

TGACTTTTCTTGAACCCATATTTCAGCGTAGCTTGAGCAAACCTTTCAAA

CCAAGCCCTAGGAGATTGCTTTAACTTATAAAGTGGCTTTCGGAGCCTGC

ATACTTTACCGTTAGATTGAACATTCGCAAAACTAGGGGAATATCCATAT

ATACCTCTTCTGCTAGGTCTCCATAAATGAATGCATTCTTCACATCAAAC

TGATAAAGTGGCCAATCCAAATTTGCAGCAAGAGAGAGCAAGATTCTCAT

AGAATTCATCTTAGCAACCGGTGCAAATGTCTCTGGTAATCCACCCTAGA

AGTTTGAGTGAAACCCTTAGCAACCAGTCTCGCTTTGTACCGTTCTATAG

AACCATCTGCTTTGTGTTTTACTGTGTACACCCACTTACACCCAACTGGA

TTTTTGCCTCTAGGTAACTCCACTAACTCCCAACTGCCACTCTTGCTGAG

AGTTCTTATCTCTTCCATCATGGCATGTTTCCATTCAGGAATAGAGAGAA

CATCCTGAAAATTGGTAGGTAGACTTACAGAAGGCACTTTAGACAGAAAA

ACTTTATATGAGGAAGACAGATGAGAATAAGAGACATGATTAGTAATAGG

ATGCTGAGTACATGACCTAACCCCCTTTCTATTAGCAATGAGAACATCTA

GATTTGAAAAAGCAGGAATATAAGCACTAGAATCAGAATTATCTGAACTC

TCAGGAGAGGGGGGTGACTCTGGACACTCATCCGGTGGCAATAGAGGAAG

GAAAATAGTCTCTTGCATCCAGGTGACTTGCTGTCTGTCCTTGTCCCTGA

TATAAACTTTGCCTGCTTCCTTTTCCTCAACAACTGCTGGCTCATCATCC

TTTATCTGTTCAATCACTGGCGGAAGTGGAATAAGAATCTCTTCACACCA

AGGCGCTTCCTGAAGGAATGACTGTGAGCCGAGGAGGTCGGAAAGTAAGG

TTGAGACTCAAAGAAAGTGACATCTATGGAGACAAACTATTTGCGAGTGG

GAGGATGATATCACTTATAACCCTTTTTGGTGGCAGAATAACTCAGAAAA

ACACACTTAAGAGACCTATGATCTAATTTATCACGAGTGGGTGAATGATT

ATGGACAAAGCAAACGTAGCCAAAAGTCTTAGGAGGAATGGAGCACTCGA

AAGGGGTAGACCCGAACAAAACTTGAAGATGTGTTTGTTTTTGAAGTACT

TGGGTAGAAAGACGATTAATCAAATAAGCAGCAGTGAGAATTGCTTCCCC

CCAATAGGATCAAGGAACCCGTATCTCAAAGGATAGACAACGGGCGATAT

CAAGAAGATGGTGATTTTTGCGTTCTGCAATACCATTTTGTTGAGGGGTA

TTAGTGCATGAAATCTGGTGAATAATGCCATGAGTGGCGAAGTAAGTGGT

AAGACCAGAGTCAATATATTCCTTCCCATTATCAGAACGTAGAATTTTGA

CAATCTCCCCAAATTGAGTACAAACTATTTTATGGTAGTTTTGAAACACA

AAAAATACCTTATTTTTATCTTTCATCAAATAGACTCAAGTGGCCCTGGA

ACAATCATCTATGAAGGTAATAAACCATTTTTGACCGGTTAAAGACACAA

AACGGGATGGATCTCATACATCAGAATGAATTAAAGAAAATACAACTAAA

CTTTTATTAATAGAAGGAGCAAAAGAGGCTCTCTGTTGTTTTCCAAGTTG

ACAAGACTCACATTGAAAATCGACACATTATTTTTCTCAAAAACAGAAGG

AAATAAATGCTTAAGTTATAAAAAAGAGGGATGTCCTAAGCGACGGTGCC

ACAACCAAATGTCTTCCTTGGTAGATGATAACTGAATAGATGAATGAGCC

AGCTTGTCTGTTTGATCCATAATGTAGAGCCCATTTTCCTCTCTACCACT

GCCAATCCTCTTCCTCGTGATAAGGTCCTGAAAAAGACAATGATCAGGAA

AAAACGTCATGCTATAATTTAAGTCACGAGTAATACGATTCAAAGATAAA

AGATTAACGCTGAAATTAGGAACATGAAGGACAAAAGACAAAGGAAGGGA

AGAGGTGGCATGAATTAAACCTTTCCCTGACACAGACGAAATGGTACCCT

CAATAATACGTACTTTTTCCTGACTTGAGGAGGGGTTATACTCGGAAAAT

AAATTAGAGGAGCCAGACATATGGTCGGAAGCACCAGAATCAATAATCCA

TGTTATAGTCGTATGAGATAGCAATGCACATGGGGAGGAAGCCAAGTTAC

CTTAATTAGCAAAGGAAAAGAAGGTGCACCTGTAGTAGAAGTGGAGGCTC

GATGGTCTAGCAAGTGGCGAAGAACAATCTCAAAATCTGAGGCAGATAAT

CCCGAGACATCAGTAGCGGAGGAGACAACTGGAGATGAAGATGGTGGCTT

AACAGTGTCTGAAAGATGGGCTTGTGCATTGGCCTCGTGGCCTCTAGTAC

CAGACCAACCCCTCGAGTACCAAGACGGTCGCCTCGGCCACCAGAGGGAG

TTCCATACAGCTTCTAACAAGTCTTACGTGTGTGGCGAGGTCGACGACAA

TAGTCACAGTAAAGATGATCCTTCTCAAAGGAAGGACGAGAACCACGAGT

CACACGATCGCCATCGCGAGAGGGTGCAGAAACCAAGGCCGAGCTATCAG

TAGTAGCAAAAGGAAGTATCGCAGAACGGCGACTCTTCTCATTCTGTACA

AAAGCAAAGGTTTCTTGGAGAGAAGGAAGCACATCCATGCCAAGTACTTT

GCTCCGAACCGGATCATACTCCACATTTAGGCCTTCCAAGAACTTGAAGA

CCCGCTCTTGCGCCACAAACTTCCGGTCAGTGTTTTTAAAGGCTCAAGGC

GCACTAAGGCGCTGAAGGTCTCTCGGGGCCAAGGCGCAAGGCGAGGCGCG

GACTTTATTGAAGTGAGGCGCACAGTTTTAGTAAAAAATAGAAAAATTAA

TATAGACTAATCGTTCATAGTACTAAGACAATTTTTTTAAAAATAAAAAA

TAATCAAGATTCAAGGCAAAATATAGAATAATTTTCAAAGCCATGAAAGT

GCATATTACATAACATTTCAATTTTCAAATATAAAATAATCCACATTCAA

TAAAATAAAAATAAAAATAAAAACATAAACACCAAATATCCAAAACATAA

ATATCCAACAAATACTAATATATGTCTTAACACTTAAAAACATCAATATC

AAAGGAAAGGGTAGGTTAGGTCGGTCAGTCAATCATCATCCTCAACATCA

ATGGCAAGCAAACTATCATCTTCTTCTCCATCAGTTGATTTGTGTCCCTC

AACATCTTCCTCCTTTGTGTCATCTGAGCCCGCTTCCTCTTCATCTATTA

ATTGAAGTCGTGCTAATATTGATGATTTAGATATCCTCAGTTGTGCCTTT

GAGCGTGAACTTGTTGCTCTACTTGCATGGCTACTTTCTTCTACTCCAGA

AGCTCTAGCAACAGTGGCTCACGTCAAGCCATCATCATCATCAAACACAG

GCTCATTATCTTCATCTGATTCTCCATCCATCCTCCCAAGCAACCACTCA

TTGCTTTCATCAATATTTTGCAAAGATATGGGATCAAGAGTGTTGCGCAT

GTCATACCTAAATCTCAATGTTCTATTGTATTTGACATACACTAAATCAT

TGAGACGTTGTTGTTCCAATCTATTTCTTTTCTTGCTATGAAGCTGCAAT

TATTAAAAAGATATTAAATTCTATATAATGAGAATAAAAAAATTTGAATT

CAACAAAAAATTAGTTAGAATGTTAAATACTCACATGTTCGAATACACTC

CAATTTCGTTCACAACCCGATGAACTACATGTTAGGCTAAGGACTTTTAT

AGCAAAAGTTTGCAAGTTTGGAGTATAAGAACCATATGATGCCCACCAAT

CAGCTGCGATAATCCACTAGTTAGAAATAATTGTTACCAAGTATTAAGTT

TTACTAAAAGTATATATAGGACAGAAGACAAAGGAAGAGAAGCAGTGGCA

TGAATTAAACCTGAAGATAGAAGTGGAGAATCCGAATTGGTCTCTTGTAC

CCAAGTAACTTTCTGTCTATCATTGTCCCTAACATACACTTTGTTGTCTT

TCACTGCGGATTGAGAGGCAACATTCTCAAGTTGGGTCACCAATAATAAT

AGTCTCAACATTCTCAATGGCTGATGGTACTAATACAGAAGTCTCAATAG

CTGGTAGTGATACAGGAGTCGCTTAAGGCTCTCCTTGAGAGATGACTGGG

ACACCGGAGCGACAAGAAGTAGGACTGAGATTCAAAGAAAGACACATCCA

TAGAGACGAACCATTTCCGAGAGGGAGGATGATAACACTTGTAACCCTTT

TAGGTGGCGGAGTAACCTAAGAAAACACACCAAAGAGACCGAGGATCCAA

CTTACCACAGGTAGGAGAATGGTTATGAACAAAGCACACACACCCAAAGG

TTTTGGGAGGAATGGAGAACACAGAAGATTGGAACCCGAACAAAACCTGA

AGAGGAGTTTGTTTGCAAAGAACCTGGGTAAGAAGACAAAGATTTCATTA

TACTTCTCTTCTTTATGATCAAATGAACTTGCAATGCATTCTTTTGCCCT

ATCCATTGCCTCATAAATGTATCCCATGGGAGGTTTCTTCTCGGTATCAA

CCAATCGAAGCACTTTGATGAGAGGACCGAGACCTTAAGGGCAAGTACAA

CAGTATTCCAAAATGAAGGCATCAACAAAGTTTGTGCCATTCTTTTACCG

TGTGGCTCTTTTGCCCATTTGCTTGAGGTCCAGTCTTCAGAGGTGACCAT

CTTTCTCAAATTGTTTTTTTGTTGATGCATCCTAGACAAAGTCAGAAAGG

CGGTTGCAAATCTAGTTTTTGCAGGCTTGATCAAATTCTTCAAATTTGTA

TAGCGCCTCATCATGTTTAGCAACGTAGGTCGGTTGTAAATGTAGTCATG

TACTGCAATACCCCTTTCAAATGTCTTTTTCAGTCTAGGTATCTTGAAAA

TGTCTTCTAACATCAAGTCCAAGCAATGAGCAGCACATGGTGTCCAATGC

AAGTGTTCATACTTGGTTTCTAAAAACCTCCCTATAATGTAACAATAACG

AAGAATTATTTTTTTCAAGTTTTTAAATTTCAAATACAAAACAAATTATG

TGTAAACAATTTTGATGTAAGCAAATATTCTTACTCGCCAACACATTAGC

TGCAGCACTATCAGTGATAACTTGCACTACATCCTCTTTTCCAACTTTCT

CCACAAACTTGTCTAGTAATTCAAATAGCTTTTGCCCATCCTTTGAATAG

CTAGAAGTATCAACCGACTCAACAAACATGGTTCCTCTTGGACAATAAAC

TAAAAAGCTAATCAATGTCCTTCCTCTCCTATCAGTCCAGCCATCAGCTA

TTATGGAACACCCGTATTTTGCACAATCCTCTTTATGATTCTTCATCAAA

TCTTCAGTATGTTTCACTTCTTTTTTGAGTTGGGTAACCCGCACTTCATG

GTAAGTTGGTGGCTTCATACCAGGACCATATTGCGCAATGGCTTCAATCA

TTGGTCCAAAACTCTCATAATTTACTGCATTAAAAGGGATACCAGCATCA

TACATCCACCTTGCAAATCTTTGCACTGCCCGAGCTCTCATTTCTTTTTG

TAAGGGTCATTTGCATCAATTTTGGTTTGCTTCCCTTTTGTTTGGTCTCT

TCGATTTTTCACTACTGATTCAGGATTGGGTGTGAAGTAAACATCCATAG

GCCCTTTTCTCTTTGGCATTTTTGATTTTGATTTGGATGAACTTTAGCTG

CTACTTGTCAACTTACGGCTTTGATTGACATTGATCTCAACAACATCATC

ATCTTCATCTTTATCTTCCATGTCCATGTCATCGAAATCAGGCAATATGT

TCATTTCTTCTTTTTGTGTCTTCTTTTTTGACATGAACTCTCTAATCTCT

TCTCTTACATGAGCTAGGCACTTCGGGCAAGCAGTGACATTCCTATAACC

TCCCGTGAGGTGTTATTTCACCCGATAAACACCTCCATTTGATACTTTAC

CACAAAAATTACACTCAAACTTGTTTAAATTATTCTCCTCTACCAAATGG

TTATGCCGGATCTTTTCTATTACTTTCAGATGTTATTTTCTACAGTCTAC

ACAACAAATTTAATAAATAAATATAGAATCAGAGACTCAAACCAGTAGCA

CCTAACATAATTTATTTTTCTCCCCCAACAGATAAATACAGAATTTATTA

AATACAGATAAATACAAAATTTAATAAATACAGAATTTATTTTTTTCCCC

TAACAGATAAATACAGAATTTATTAAAAACAGATAACTATAGAATTTAAT

AAATACATAATTTATTTTTTTCCCCTAACAGATAAATACAGAATTTATTA

AATACATAATTTAATAAATACAGATAAATACAGAATTTATTAAATACAGA

TAAATATAGAATTTAATAAATACAGAATTTATTTTTTTCCCCTAACGGAT

AAAGGGGAAAAAATGACCAGTAGCATTGTATTTATTTATTTATTATTTTG

TATTTTGTATTTATATTTATTTATTTTGTATTTGTATTTATTTATTTATT

TTCCTAACATAATTGAAAAAAAATAATAAAAAAAATAAAAAATAAAAATA

AAACAAAGGCGAATGGAAGCCATAAGAAGAAGAAGAAGAAGGGGAGAGAG

AGAGAGAGAGTGAGATCAGAAGAAGACCAAAAGAGGTCGAAAAAAAAAAA

AAAAAAAAAAAAACGAAGTCGAATCGAGGAGACCAGAAGAGGAGGAGAAG

AAGAAGATGGCAAGGCGAAGCATACCTGTGCGAAGTCGAATCGAGGTCCA

CAGGCGAAGTCGTTGTGAAGTCGAAGTCGAATCGAGGTCCACAGTCGAAG

TCGAAGTCCAAGGCGAGGCAGAGAGGAAAAACGACATAGATTAGGATTCA

TTTTAGTCGTATTTGGTGATATTCTGCATGAGTTGTTATCCTCTGTGGTT

AAGAGCCATTCGAACACACCTTCCGTAGCCGTTAGATCAGATCAAGCTGC

TAATACATCAAGCGCGCCTAGTCGTGCGCCTCGCTCGCCTGGCTCAAGTC

GCGGCTAAGGCGCTCGCCTAGCTCAAGTCGCCTTGCGTGGGACCTGTACA

GGCGCAAAGGGTGTGCCTCGCCTCGCCTGGCGCCTAGGCGCGCTTTTCAC

AACACTGCTTCCGGTAACCAGCAGAATTAGTGGGACAGACATGCTTGTAA

TCTTGAAGGTGATCAAGCCGCTTCTACCCATTGCTAAGGAAGGTAAAATA

CTGAAGGATAGTTTGCTCACCTTGTACTGACCGTTCAATGGAGCGACGTA

ACTTGAAAGCCTGTGAGAAGTTTCCCTTCCTAGAGTAAGTAGTTGCTACG

GCCTCCCAAATATTCTGAGCAGTGTCTATAGACAAGTAGCCCTCTCCAAT

CTCCAAAACCATAGAATGGATCAACCAGCCCATAATCAGAGAATTAGTAA

TCTCCCACTAGTCATAACGAGGGTCGGTGATATTGGGTGCCTGAACTCTC

CCATCAACATATTCCATCCTCCCTTTGCTTTTCAAGAAAATTGTCTGACT

CTGCGACCAAGCTAGGTAATTCTGACTATTGAATAGAGAGGTGGTGATTC

TAAGGCCAATGTGGAGGTTATCAGTCATAGTGGAAGGGATAACAGAACTC

TCGCCCTGAGATGGCTGAGAGGTAGTAGATTCATCGGTCAAATCCGACAT

GGAGACTCTACTAATTATTCCTCAAACACGAGGCAAATCACAAACCCGGA

AGATGAGAGTGGAGTTACAAGCACAACAGTAACTATAAAAGCATAAAGGA

GGGAGAATCCAGTGGGGCTTTGTGTCTGGACTATTTTTACATCAGCAGAG

TTTTTTTTTTGGACTGCTTCCATACTGTTTTACCTTACCAGAAGACTGTA

TATAATCTTCACCAGTCCACTATATAAGAGACAACTTCGTATTACATCAC

CAGAGGACTTTGAAGAGCACCAGAGGACTTTGAAGAGCAGCAGAGGAGCA

GCAGAGGAGCAGCGACCACCCTAGGACTTATGTGAAATTTTTAGTAATCC

CATGTGAGTTGTATATGGTATTTTTAACAATTTCCACACAAGAATTTATT

TATTTATTTATTTTTTTTCTGGTGGTTCCTATAGTTTTTTTTTTTTAATT

TTTTTTTTTTTAAAATTTTGCTTACTCTAGCTTTGCATGTGTTGTTTTTC

TATCCAAAATGTTAAGCAAAGTATCACATTAGTCATTGTATTATCAATTT

GAATATCATATTTTTAGTTTTGTTATAATATTATTCCTCTGTCATGACTG

GAGTGACTACATAAAATTCAAAATAAACTATAGAGACCACTAATATAATT

TATTAATTTGTTTTTCTTGTTTGAAAATTACTAAAAATCCCATGTAAGTT

GCATGTGATCTGTTTAAGTTCAAAATTATAATAGTTAATGACGAATGGAC

TACATTGCTACAAAACGGAAAGTATAGGATTTAAATTTAATACACAGAGT

GACACTTTGTTTAAACTATAGAGAATAATAGTGTAATTTACTCAAATTAT

AAAATTTTATATAGAATGATGTGATTGATTTGGTTTAAATAATTCAAAAA

AAATATCATGCAAATTAATAAATCTCAGTAGCTTTTAACAAACTCCAATG

AATAAAAAAAGTTAATAAATTTATTAATTTATATAATATTCCTCTTTATG

AAATTTATGGGATTGGGGTCACTGCTGTGCCTTTGGCACATCAGTGACTG

TTGTGCCTCATTTTGAGGTGCATTATGGATTTATTTTTATAATCTGAACC

GTTCATTTGTAGGGTTTGTTGTGTTAGAATTTCCCATCTTTACCTTCACC

TACTCCACTACACACTCCACTTCACCTACCTCCACTTCACCACACACTCC

ACTTCACCTAACCCACTATCACCTCCACTACACCTACTACCACATTTTCT

TCTATATATAGATTGTATAGGAATTGTAATTAATCAATCTTGTAATACAT

CAATCAATAATATACTTCAATACTTTTACAGTTGAGTATTTCATGCCTAC

CAAAAATCATATTAATCGGATATCAATAACTATATGATCGAATTGCATAT

TTTTATACTATTTTTCAAAAGAAAAACTTCTTGTTTATTTTTTTGGTTAT

CAATCATATAGTTATTGATATCCGATTAATATGATTTTTAGTAGGCATGA

AGTACTCAACAAATCTTACAAAATGAATGGTTAAAATCATAAAAATAAGC

CCATAATGTACCTCAAAATAAGGCACAACAGTCACTGCTGTGCCAAAGGC

ACAGCAGTGACCCTAATTCCCATTTTTATTGGTTTAGACTCAGCAGTGAC

CCTAATTCCCATTTTTATTGGTTTAGACTCATTATCAACTTTTTAGACAG

AAGAAAAATGTTTACACAAATTTTCATCTTCTCTTCAAAGGATAATAATG

GTCAATAGGACTTTAATTACTACAACTAAGCAAATTACATATATTGATGA

GTTTTCTAGATTTTGCATTATTGTCAAGCTTTTATTTTACATATTGTTTT

TAATTTGTATTTGTTGTCTAGATTAATGGCATCCAAGTATTTTAAATAAA

AAATCTAGACATACATTTCATTGGATAGCAAAATAATAAATAAACAAATA

AATGCAGATTTTTTTTTATTGAATGCATTATCTTCCTATCTGTCATTCAG

TCCTCTACTATAAATATTATGTGGAGTTTGGATAGTATCAGTATTCACAA

ATGAGTGCACAGTTGTCAGAATCCACAAATCCATCAACATTGCTTCTCTC

TCTCTCTACTTTCCTCCTCCACTGATTTGAGATCTGAGTCAAATTCTAGA

GATCCATCGCCGGTGACGAATGCAGAGAGACCATTTGTAGCCGTCGCCAT

TGCCATCGCTGAAGGCAGAGAGACCAGATCTGCTCAATGGTAGATTCGCC

GCCACCTGACATCACTCGTTTTTCTCTCTCAATCCTTGCTCGAACCCCGA

CTCTTCCTCTCTCCTCTCACCCTCTCTTTACGTCGTTAGTCTAGGTGCTA

GAATATCATGTCCATAGGGACAAGTTCTCGCAAATTGACCAAACTACCCT

TAGATATATCTGACTCAAGTGTTTCCTTATATGTCATCATTTCAAATTGA

AGAACCATTAATCTTAACAATAATTAGAGAAATTTCACTGTTGCTTTACT

GTGGATGGGTTCTTGATGAAATCCATTTTGTTTCTTTCATCTCTTTGTTC

TGACCCAAACATTGGGATATAAAAATAAGAACTCAGCCCTGGATGAAATC

CAGCACCACCACCAAAAGAAGTCTGGCCCACCCCATATCCAAAGGGATTG

GCAGGCTGTTCATGCACAATCCTATCAGTGCGGATTCCAATCTTAGTCCT

AAGTTCCATCATAGCAGCCCAATCCCTTTGGATATGGGGTGGGCCAGACT

TCTTTTGGTGGTGGTTGTAAGAATTTATGTATTATTGATTGATATTGTTG

TTGTTATAATATTGATATTACAAGGATTCCTATTATATAGGAGAGGATTT

GGGTAGTAGGTGAGGAGTAGGTGAGGGTACATTGGTGGTAGGTGTAGTAG

GTGTGGTAGTAGGTGGTGGTAGGTGTAGGTACATTTTAACAGTGGTGTTG

GAGGTGCTGAATTTCATCCAGGGCCGAGTTCTTTTGGAGGTGCTGGATTT

CTTGCTGGGCTGAGTTCTTTTGGTAGTGGTGGTGGTAACACTGCTGCTGG

TGGTGGTGGATTTTTTGCTGGGCTTGGTGGTGGTGGATTTTTTGATGGGT

AGAGTTCTTTTGGTGGTGGTGCTGGTGGCTCGTCATTTCAAGCGGGCCCC

AGTTTTGCTGGAGGTGGTATTGGCTTGCCATTTCAGGCGGGCCCGAGTTT

TGCTAGAGGTGGTGGTATTGGTGCTTTACTATTTGGTGCAGGGCCGGGTT

CTTTTGGAGCAGATGGTGGTGGCAGCGACAACAATGGCATTGTTCCATTT

GATGGTAGGGTTCCAAACGCCGGGTGGTTCGAAGGCTCCTTATAGATTTA

ACCATGGCTATGGTCGTGGTTTTTACTAGAAGCTTATATGGCTGATTAAT

ATTAATGTTTTTTACATTGTGCACAGTTTTTCTTTTTCCTTTTTTTGTCG

TTGGTGATGTTGAACCCTATATTGGAATAAATTGGTACTTTTATGTGTGT

GATTCATATGGTCGTTGTGAGTTTACTTTCAATTTCTTGGAAAATATTAT

ATGATTTTTGCCTCCTGAAAGTAATTAATTGTTATCTTATTTATCAATTT

TCTTATTACTTTCTCAGACAAAATCTATCTTAACCAAATAAGTACAAGAA

AATAAATTTTCTTTTCTCTTCACTTTCTATAATTTTCATTCTAATGAAAC

ACACCTTTAATTTGAGAAGGAAAAATTATCTTAGCCATCTAGCATTGCTT

TCAAATTATCTCTCTTGTTTTTTTTTTTTTTGTCTTTTAATCTTGTTTTT

ATATCCCAATGTTTGGGTCAGAACAAAGAGATGAAAGAAACAAAATGGAT

TTCATCAAGAACCCATCCACAGCAAAGCAACGGGGGGCTGTTAGAATTTA

CCATCACCTACCACCACTACCACCTCACCTACTCCACTAAACCTAACCCA

CTACACCACTATCACCATCCACTACACACCATCCACTACACCTACATCTA

TATAAGGAACCATTTGTAATAGATATAAATGTATCAAAAAATCAATCAAT

AATACAATTCTCTATTTCTTTTTATACATCAATTTTAACAGACAGATAGG

AAGATAACGCATTCAGTAAAAAAAAATCTGCATTTATTTGTTTATTTATT

ATTTTGCTAGGTGTATTTGACTTTTTTTTTAATGGAGATGAACACTCAAT

ATGCAAGGAACCAATGCCAATGTGTTAAAGACATATATGGATTTGTGATT

TGATCAATCCAACATATACAGAGTTGAGTTTTTGTTATGTTTTATTTTCA

ATGATAAACTTATCGCCGCGTAGGGCTGAAAAAAAGGGGTAATGAATCTC

AAATTTCATGAAGTTTTATTTGTAAAGTCATTACGATTTCAAAGCGGGAA

ATGAACTTGAATGCGTTCCTAATAAAATGAATGGAGAATTTAATGTGAGA

GATAGTTTGACATTGAGGTATTTAATTTGTGAGAAACTTTGTGGAAATCA

AGTCATTTGATGTATTAAAAAATAATTCATAGAAAATATATACAGTTTCG

TTTTTTTTGTCATTTATTTATATATATATTTTTTTATGGATTGTATGAGG

AGATTAAAGAAATATGACCTTTCTAAAGTGTCAAATATGAATGTTTTATG

AATTTCATATGGGACTAATTACTTACGTAAAGTTTACTTTTTATGTACCA

ACAAGGTTTATTGAAGGAAAAAATGACGATTTATTGAATACTCTTTTCTC

ACAATTACCTGTTGCATTCTTTATGCATTCTGAGAAATCATTCTAAAAGT

TTCTTTAGAATATCAAAACTCCTTAACCATTTGATTATAGTTTGAAAGGC

TAAAAAAAAGTTTTTGAATAATCACGGACTTGTATAAATTTTTCTTATTG

ATTTCAGAACCTGCAATGCTTTTGAAGAAAAGCATTCAAGTTGACCAAAT

TGAGTTAATTTTTCATGCAAAATATATTGCACATGAATTGGCAAATCTTT

TTTTTTTTTTTTTTCCAATGCTGCGATGGCACAATTCATGAATGCAAAAT

GACTTGTACAGCTAAGCAACACCTAGGTCAAGCCGAGACATTTATTTATT

GATTGACAAAAGTCAGTTGTTAATTATTAGATTAATACTTATAGAAGTTA

GACATTTCCACTCTTGCAAAGTACTAAATGCTTGCCTCCTTAATTAAACT

TCCCCTTAAGAAGAATTGAACATATAGATGAATGTGATTAAGTATAAAAC

CATACATTATATAATGCATAATTTTTTTTCAATTCATTTTTTTTGCAATT

TTTAATTTTCACACAAAGCAATACCAATCAGATTTTGCTCAGTGTGTTGT

GAATTGTAAATTAATTATACAAACTATACAAGTCCAACCTAGAGAGTAAC

CTATTTAGAAATATTCAGTGCTGATATAATCCAAGAAAAGATTTCTGAAA

TCTATACAACCTGTAACAATGTCAAAAATTCTAGCAAGACTTGGGTTTAT

ACTTTATATGGTAAAATGAAATCAATGGCACCTATTCAGAAATATTCAGT

GCTGATATAATCCAAGAAAAGATTTTTGAAATCTACACAACCTCTAACAA

TGTCAAAAATTCTAGCAAGACTTGGGTTTATACTTTATATGGTAAAATGA

AATCAATGGCATGATTTCTAATTTTTTCATTTAGTTAGTGTTTTTTAAAT

AATTTTAATTACCTTTCAAAGTGTTTATTTTTGATGAAAACATGTTTATA

TGGTTAAGTGAACAGCCTGCAAAAAGGACAAAAAGCCAAAATGAGTGTCT

TTTATTTTTTATTTTAAATTGTGAAGCATGTTAAAAGAATGTAAGACCAG

CAAGAAGGCTGGTCGGCCTCCTGGGAACCCTCGGTAGTGGCTGGAGAGGC

CTACCAAGGGAGAGAGAAGCCTAGGTGATGACTGTCAAAGGAAGAGAAGA

GACTGGGAGACCTGGGTAGAGTCTGTTGGGGTTAGACGAGGCTGGGTGAC

TTGTTTGGGGCAATCGTCTGGTCTATAGGAGAGAGGAGATCACCTATAGT

GATGGGTAACAGAGAAGGAGAGTTGACTCTGATACCAAGAAGGTTTAGGG

AAGAGAAAAAGTTAGAGAGTGAGTTTGACTTCATTCCCTTTTATATTAAT

AATACTAAGTAATTACTGGAGTGCCATAAGGCATAATACATGGGCCAGCC

CATTACCTATGAAGCATAGACATGGCAAAATTAGCGTCGTGGTGGTGTCG

AACACGGGGACACGGGGATACGCCGGGGACACGTACGGGACACATCATCT

GCCGTGTCCCTTTAATTTAATTGTTTTCTTGTAGGGGACACGCGTGTCCA

AATCTGGACACGGCGTGAGACACGACAAGGAGGGAGAGACAAAGGAGAGA

GTGTGAAGGAAGAGAGTGAAGAGAGACAAACCTGTCGAAGATCGAAGATC

GAAGCTCCATGAATCCAGTCGAAGATCGAAGCTCCAAGCTCCATCAGTCG

ATCGAACAATCAAACACCCCAGACGATCGCGATCTGTCTCTCTCTTTCGC

CTTCACAATCTGTCTCTCTCGATCGCGACCTCGACCTGTCTCCCTCTCTC

TCTCTCCTTCTCTCTCTCCCTTGCTTCTGTCGTTTTATCTGAGGGTCTAA

CTATTATTATTATATATTATATAATAGTTATTATATAGCATATTATTATA

TATTATATAATAGTTATTATATAACATATAGTATATTTATAAGATAATAT

ATAATACATATTGTAAGATAATATATAAATAAACACATGTATGTAAAATA

AAATAAAAAAATTAATTATTTAAAAAATATATATTGTAAGATAATATATA

CTCATACATATTAATAAAAAAATAAATAAACACATGTATGTAAAATAAAA

TAAAAAAATTAATTATTTAAAAAATATAATAAATAAATAAAATAATTAAT

GTATATTAAAATAAAAATATTTATAAAAAAATAAAAAGATTTGCCGTGTT

TCCTGCCGTGTTCGTGTCCCTATTTTTTAGAAATTGCTGTGTCCCGTGTC

CGTGTCCGTATCCGTATGCGTGTCCGTGTCCGTGCTTCATAGCCCATTAC

ACCAATCAAGGAATAAATAAGAAGAAAATACAATCACATATTTCCTATTC

TAACTTGTTGCCTATTCTAACATGATCCTATGTTGAACTTCAAAGGGTAG

GCTTTAAGAGTGCAGTCGTATGATTATATATAATTTTCTTGCTCTTATTG

CACAGAATGCAGTTGTCCTAAAGAGGGACTCTGGAAGTTTGTGTGATTTA

AGTGCTAGGCAGTTGCTCATGTTGTTGGTTGAGAACACCTGTAGTTTTCT

CAATAGTTTTTCTGCCATTTATCATGCTGATATGATGTGAAGTTAACTAT

CTGGAGAGCTTTAGGTCAGTCATACGTGGTTGGTTTGTTATATTTAGAAA

TTAGAAGTGTTTGTGCTTCTTAGGAGATGACCCAAAGTAACAAACCAACC

CTCCAGAGCAGTGTTACCAGAATAATGGTTAATATAATAAAAAGGCTTGG

TTTGGTTTGGCTTATATTATCTCCTGTTCATGAGTTTTATCACATTTTGC

TTTTGACAATGGCTTACAACAACAGTAACTCATGACACTAATCAAGTGGT

AACATACTTTTATCTAGGTTGGTGTTCTTCCTATGGATGAATCAAAAGTT

ATCGCAAAAGGTCGTCTTGGTCCAGGAATGATGATAACGGTTGATCTACT

TAGTGGTCAGGTTTGTACAGTAATTTATTAATGACTTGGTGCATAAATTG

CAAGATGGTATTGCCTTTTGATGATTATGTTTGCATCCACACAGTAGAGG

TTTTTTGTCGACTTTGCTGTTCAGCTTTCAATATCCACTTTGACTTGTTA

CTGGTTTTTCATTTAAAAGCTTCTAGACAGATTTCCTTTGCAAAATATCA

ATTTATTCATTTTATGAAAAATATATGCCACTAATCATAAAAAATATGAA

GCATATATGTCATTTATCATAAAAAATTATGAACATGCCCCCTCGGCATG

TTCTGTATTACAGTTTTTTCTTTGTTGCTGATAAAGAAAAGGGGATAAGT

GATGCACATCTAATACTCTTTTTTTCTTCTTCTTCTTCCTTTTTTTTTTT

TTCCTTACTGTTGCTTTTATAATTGTGTGCCATTCTGTTTTATCTATGTT

GATCCCATTTATCTTTGCATACATTTTCTGTGCAATAAGAGCGCTTTCAA

ATCTTTTTGAATCACTATGCTAAAATGTTCAACACTAAGAATTTATAAGA

ACATTAAAATGCTACTATAACAAAGAAAGTGTACGACTGAAAAAACCAAG

AAACTAATAAGTAGAAGTAATATATTTATTATGAGCCACCATGCAGCAAT

ATAGCAATTGCTTTTCCATGTTTGTGCTTGCTGTTAGGTAAATTGTGGTG

TGACAGATTCCTTTTATTTATGCAAACTTACAAGTAAACCGACATGTATT

TTTTGTTCAGCAAGAAAACAAATTTTTTTTTTCCCCCTCATGAAAATCAA

AAGACTACAAATGAGGACAAGCAATCCTCTATAAGAAGAAAAAGACTAGT

TACAAATTACACACCGCTCTCCAATCATGATTAATCAAGTACAAATAGAT

CTCCTTAAAGGGAAGCTGGAGCTCAAAAAAATGCCACGAAGTGAACCTTA

TCTCTAATCCTGTTAAGACCCTTAACTTTATCTTCAAATACTCTTGTATT

CCTTTCTAATTACAGTAACCAAAACATAGCACAAGTCAGACACTTCCAAA

GAATACGTGGCTTCTTACTCGAATCAAAACCCCAAAATTTGAGGGTAAGC

ATTTGAGTATAAGAATGTAGAGTCACCCAATCCTTTTGTATCTCAAGAAA

GAGCCTCTACCATAACTCAAAGGGAACAGGACAATGCAAAAAAGATGGTT

GATGCTCTTCTCATACCCCTTGCACATAATGCACCACTTAGGGGCTTTTG

TTATACTCATACATAGGGCTGCAAAGGAACCGAGCCGTTTGTGAACATGT

TCGAGCTCGACTCGGCAAGAGCTTGTTCGAACTTGGTTCGTTAATTTTTT

TTTTGCGTTCGAGCTCTACCTCAAGCTCTAGCTCAAGCTCGTTAAATTTT

TAATAAATAAGTCCAAACATACTAAAATTCGGCTCGTAAGCTCAGCTCGT

TAGCTAGCTCATTTAACATTTATTATTTTTATGTTAAATAATATAAAAAT

ATGAATAAAAAATAAGATAAATATGTTATTTATATATTTAAAAAATATAA

ATTTAAATAATAGTTAACTATAGATAGTACATTTATATAATAGTGGTGGT

AGATATTATTATTTTAGTAAAATATTGACTTTTTTTTGGTATGTTTATAT

ATATATATATTGTATATATAATTACATAATCAAAGTTTTAATTGTAACTT

TAGTCACATATGTAAGTATGAATTTATAAATTTTTTGGCCCATTAAGGCT

TGTGAATAGGCTCGAGCTCAGTTCGTTAAATTTTTATTGAGCTCTTGAGC

TCGAACTTGTTAAAATCTTAGTAAACAAGCCCAAACATACTAAGCTCGGC

TCGGCTTGGTTCGGTTCGTTACTTTATCTATCCCCCAACACACACATGCA

TGCGTGCACACACTCAAAAAAATAAAATAAAATCGAAATAAGGGTTTAGA

TCCTTGGACCACTTCTTATAGAAGAACTGTGATGCATTAAGAGTTAATTG

TGTCAAATCTGGTCCTAAAGCCATGTGCATCTGACAGAACACGTAGTTTT

CTCAAGATTTTTAGTATGAGATAGAAATGTGATTCTTTATCTAGAATTCT

AAATACTTCACTTTTGTCCAGCCTTTATGTGTGAAATTGTGGGTTCATCA

GAATATGGAGACTTATGATAAGTCTTCTTTATTTTTCAATCAACTTTGTT

GAGGAAAGTGTGCACCTGATTTGAAGGAGATGCTGCTTTGGAAAGGTTAA

TTCACACCTTAAACAATATGAAATGCAGGTTTATGAGAATACGGAAGTAA

AGAAACGAGTTGCCTCATCATATCCTTATGGAAAGTGGGTGAATGAAAAC

TTGCGATCCTTGAAGCCTGTGAACTTCCTCTCTGTGACAATTTTGGAGAA

CGAAGCAATATTAAGACGCCAACAGTAAGCATTTTAATTTCTTTACGTGT

TTCAAACAGCTTCAAACACCCTCTTTTTTCCATATTTAGATCTATAAGTA

TTTCTGGAACTCCATTCTAAATATTTGGCTCTTGCATAATTGGTCTAAAT

CAGTCAAGTAATTTTTCTAAGCTGTTAAAGTTCTGTCAGAGAGTAGAATC

TTTGAACTTTGTCTGACATACCTGTTTTACATGAAAAAAGGGAAACCAGA

GAAAGAAGATGGACACAATACTTCTCAAGTTGTTTAGTTAAGTAAACTAA

AGTTGGCTAGAATACAAGCTTGCGAATTGTTTGTATTGCTATTATTTTGA

ATGTAATATAAAGGGGTCATTGCTATTATTTGGTTTTCAATTTTAAAGAA

TTTGCATGTTATTCCGGTATCGGTGGCGTGACGGATGGAGAAACTTCAAA

GGAATTTTCCGTGGGATAGTGGGGGGGATGATTTTTGATACCATTTGGTG

GATTGGGGGCAGATCTGTCAACACTGGGGGGTTGGGGGTTCAGAGTTTAG

TGACTTTCAACAGAGCTTTGTTGGGCAAGTGGCTGTGACGCTTTGCTTGG

GAGCTGGAGCGGCTATGCAGACGGGTGGTGGTTGGTAAGTATGGGCTGGT

TTGGGGGGACTGGGGGTCAGGGGATGTGATTGGTTCCCATGGTTGTGGTT

TATGGAAAGGTATTTGGTTGGAATCGAAAGGAATATGGTTGGGGAGGGTG

AAGTTTTGGGAGAGGGTTGGGTTTCGGGTGGGTGATAGGATGAGGGTGCG

CTTCTAGAAGGATAGGTGGTGTAGTGAGGCTTTGCTGGAGGATTGTTTTC

CTTTGATATTTGGCATTGCGGGGGATCAAGAAGCTATGGTGGCATCTTAT

TTGGGGGGTGGGGATGTGGTGGTATGAGATATTCACTTGTGGTGCCTGGT

TTAGGATTGAGAGTTTGATTAACTGCTGGAGTTATGGGGTTATTTGTATG

TGTTGGGTGTGACTAGGGAAGGAGTGGATGTCTTGGGGCGGAATGTGGTG

GGGGCTAAGGGGCATTTTCTGTTTCGTCCTACTACAGGAGTTTGGTAGGG

GTGGTGATGGGGGATTTTTCTCGGAAGGAGCATTTGGGTGCTGGGGGTTT

CTTCAAAGGTGGCCTTCTCCACGTGGATGGCGGCCTTAAGTCGGAATCTT

ACAATCAACTTGGTGCAGCGTGGTCATATTCTAGTGAATCGGTGCTATAT

GTGTTGTGCAGATGGGGAGTCCATAGATCATTTGTTTTTGCATTGTCCTG

TGGCTAGCCATTTGTGGGGTTTTATTTGCTCTCTTTTTGGGATTGTTTGG

GTTCAGCCGCGAAGGGTGATGGATATGCTGTGGAGTTAGCATCGAGCTTG

GGTTGGTCACCGGCGGCAGCAAGTGTGGTCATTGGTGCCTTTTTGTTTGA

TGTGGTTAGTTTGGTTAGAGCGTAATAAGCGAACTTGTAAAGAGGTTCAT

CATAGCGTTTCTTGGCTTGAGAGTCGTCTTCTTATAGTTTTGTATAGTTG

GATGGAATAGAAGTTTGATTCGGATTTGTTTGTTTTTTTAGATTTTTTAG

ATGACATTCTTGATTAGGAGTTGGTGTTTATTCCTTCGGAGTTTGTTACG

AGTTTTTTAGTCTTCGCTCGATGTTTGAGCAATTTTGTATTCTTTGCATC

ATCTTGATGCTTCTTCAATAAAATTAATTTTGTTTATCAAAAAACAAAAG

AAGGGGTCGTTGTCATAGGACAAATTTCTTTCTTTTTTTTGGATAAATAA

CCAAACTTTATTAAAAAGTATCCAGATGATGCAAAGAATATAAAAATGGG

AGCCACAAAGACTCAACAAAACTAATAAATCAAATCCTCCACAAAATCTA

CAAAAGCCAGAACATCAGGATCAACTGAACTTGTCACCCAACTATGTAAG

ACCGTAAGAAGCTGTCTTTCTAACCAAGAGACCGGATGAGACACCCCTTG

AAAAACATGCCTATTACGCTCCAACCAAATCAACCACATAATACAATGAG

AAGCAAGCATCCATGCCTTCCGACGCCTCCTCCCAACCCAATCCCCAACC

CAACTTTGCAAAACTGCCTTTACCGAAACTGGCTGCACCCAAACCAACCC

AAACGTTGCAACAAGAAGCATCCAAAGTTGAGAAGTAACCGGACAATGAA

CAAGCAAATGATCCACCGACTCCGCATTACCACAACACAAGCATCACCAA

TTGACCCATATATGGCCACGACGAATTAAGTTTTCAATAGTCAAGATACT

CCCGAACATGACCATCCACACAAAAAAGGCCACCTTGGATGGGGTATCTG

GCACCCAAACACACTTCCAAGGGAAAACCGCAGCCTCCTCATGAACACAC

ATCATAATACGAGGATACAGAAAAAAATCCCCTTAGACATAGGACAGATT

GCTGGAATGTGGTTGCTAAATATATATATATATATCATAAAGCGAATCTG

GAAGCTCGCAAGAATACCTCGTATGTTGGAACATGGCAGCACTCTAAAAC

ACATGTGTTGTCAGTGTTTATAACTTATTTTTCCAACCAAACATCAGAGC

ACCGGTCTTATGCCTTGAAATATATTTGCAGACTTGCAGTAAGAATGATA

CCTAAATGGCGAATAACATTTATAATAATATAGGGGTAAACCATAGTGTT

TCCATATTATAAAAAGATAAATCCTACCTTTTGGGGCTTTCTCTAATTTT

TACCCTTTCTTTTCTTTAAAAGCCTTCCTACATTATCATGAATTGAAATG

TTACATTAAATTTGTAATTGGGTTAACCTGGATTGAAAATAGAGTTGGTT

CCTCAAAATGTTTTTGGGGAAGAATTGAGAAGGGAGGAGAGAGTATGCAC

ATGATGTGTGTTACCAGTTCCTAGTATCTGCATAAATGGATGTCATGTTT

TAAGATGTGCTTCACTAACACTTGGAGCGAAATAATTATATGGTTTGTCT

ACTAACTATTAATGGTGTAGTGCAAGAAAAATATTGACAGCATTATATCC

AGAAGGTCATGATTTTAAAGAGATTCTACGTAAAAATATCATATTGGGTC

TGTATCAATTGATTTGAGTGATTTTTTTGATTTATCCATCCTGGTTGAAG

TATCCTAAGTTTTTTTTTTTTGACAAGGAAAAGTCAACTGTGTCTTTGTA

GTTTGATTTTGATGAAAGGTTTACTTTGTGTTTGATTATTGCAACTTTAA

CCATCTTAGAAGGTGTTAGAATTTACCATCACCTACCACCACTACCACCT

CACCTACTCCACTAAACCTAACCCACTACACCACTACCACCATCCACTAC

ACCTACATCTATATAAGGAACCATTTGTAATAGACAGAACAACACATCAA

AATCAATCAATAATACAATTCTCTATTTCTCTTTATACATTAAATTTTCT

ACAGAAGGTATTAAAGGTACTCGTTCTTATACTTGATGGAAACAGGGCTT

ATGGCTACTCCAGTGAGGATGTCCAAATGGTCATTGAAACTATGGCTGCA

CAAGGAAAGGAGCCTACATTTTGCATGGGGGATGATATTCCACTGGCGAT

ATTGTCTCAGAAGTCACATATGCTTTATGATTATTTCAAGCAGCGGTTTG

CTCAGGTTTGCCAATTGTTTTTAATTTTTTTTTTTTTTTTTTTGGTTTTA

GATGAGCCTGAATATTCTAATGGTGTGGCTAGTTATTTGTTACTATAACA

ATATTTGATTGAATTGTGATGGTAGTAAAATAATGTCTCGGGGTCATGGT

TAGGAATTATTCTTGACGTGACAACAATAGGTGATTTAATTGTTGGTTAA

TTTGTCAAAGGTGTAATTTACAAGTTGGATAGTGGTAGCAGTGGGGCAGT

TGTGGCACAAGCAACCGTTAATACCAAGCCCCACTTCCAATGGAAGTTGC

CCCCACCTGATCCCTCCTTGGCCAATAGTATGGTCTCATATCATACTAAT

GGAAGTTACCTCACCTGA

>TEA005658.1 locus=Scaffold1473:657698:668583:+ Pyridoxal-dependent decarboxylase conserved domain

ATCTCTCAAACCCTCTTCTCCTCGGATTCTCAAACCTTACCACTGTGACC

CACCGAGTCCATCAAACAACGAGGTGTCTCCTCTCCATTTTCATTACTAT

GTCTTCGTTTTTTCGAGAAATTCTATAGTATATACCTCTCAAAAATTATA

TATCAATGCATTCTGTTATGTATCTATATATTTTGTTATATATTTTTATA

ATAAATGTTACGTATTATTTTTTATAGAGTGATGATAATTTATAATTTAA

AAATAAAAATTAAAATACGCATCTTATAGTAATGTTACTTTATCCAAATA

TTTCCTAACAAAAAAATGTATAATATTATTAAAAAAATGCTTGATTTTAT

TTATGAGTTACAAATTACCGTAACTTTATATAAATAAATAATAAAAAACA

TAATCTTTCACAAAAATATATATTCAGAGTCGATATGGTATACAGTTTTC

GAAAGGTGTGTGCTAGACATTATCCAATGTTTTTGTGTTAGCTAATCCGC

AAATGTGAATCACAGATCATCATGGAAGGGACTGTGTCAGTGCTATCGAA

TGTGAGCAAGGTGGAGCTGTTGTCGAAGTGCTTTGATCTGATTACCATCC

CTGTGGAACCCTTGCCTCCGGTTGTGGCTTCCAACGGAGGTGAGATCGGT

TATGAGCATCCGCGTAGCTACATTATAATTGGAATTTATTCAATATTTTA

ACTAATAATGATTTGTATTCTCAAGGAATAAATTAAAAAATTAAGTGATA

TTTTTTAGTTTATTCAAATTGACTTCACTAAATTTTTTAGTTAAAGAGAT

GTACTAGGGTTTCATAACATGAAAAAGTATTTAAATTTTTTTTTTAAATG

TGTTTCTTACTGAAAATTTATTTCCAAGTTAAAACTAAAATGAAAGATAT

TTTAGCTTTTTGTATTTTTTAATTTTTTAAAAGTAAGTTGATACTCATAG

AATTAATGTATTGAGTACTAATTAATCCGACTAGTTGCTGGCGGAGAGAC

GAAGAATATGAAGGAGAGGGATATTGTTCTGGGGAAGAACGTGCACACAA

CAAGCCTCACCATCACGGAGCCTGATGTGGACGATGACTCCACCAGCGAT

ATGGAGGCCTTCATGGCCGGTGTCTTGGTTAGGTATCGCAAAACTCTCAT

TGAGAAGACCAAGTATCATTTAGGTAAGCAATCAATTGGTTTCTTTTTCT

TTATTTATTTATTTATTTGCTGTTTTCTCTTTTCATAATGCTGTAATCTG

TTTGGTTGTCGAGAAACTAGAGGCAAACTTCAGAAATAGGTATAGGAAAA

AGATATGATATTTTCAGTAATAGTTACCTTACCTGAGCCAGAGTTGGCTA

AAATGTCATAATAGGGACAATTCAATCCATATTTGCGTTAATTTTTATTT

ATTTATTTATTTTGCTGGAGTATGTTCTAGACAGTTTGAACAGACCACTT

CTATTATTTTTTAGGTTTTTTTTTTTGTAATTTTACATTTAATTTTTTAG

ACTATTTGATGAATGAGGGAAACCGTTTATATGCGAAAAACCTTTTAGCA

GTTAATTGGATGTGAAGTGCATTATTGGAAAAAACAAAAAATGTGACAAT

ACCAAATAGAATGTAAACTTTATGCAATAGAATAGATTATACCAAAAGAC

TGGATTTTATTTTAATTACTACATTTTATGAGACACATGTTCATAAACTC

CTTTATTAGAGTAAAATAACCGTGATATATAGAGTTGCTTTAGTCACTAT

ATAAGGCTTCCACATATTAACTTGGTCTTGTCTAATATTAGTTCTGCTTT

TAAGATACATTTGTTGTTCAAATACCTGTATAAGTTTCTTAGAAGTTACA

CATTGTGTAGTTCATGGCCTTGTAGGTATGTACAACTGGTCGGTACTAAA

ACTTCTGGAGACCTTTCTCAATTCTCACCTAAAATATATTGGTTTCTTAT

TTATTTTTGTTGCTAAATGTTGTTGCAGGCTATCCATTTAATCTGGACTT

GGATTATGGTCCTCTAGCGGAATTGCAGCATTTCGCCATAAACAACCTTG

GCGATCCATTTATTGAAAGCAACTATGGTGTTCATTCAAGACAATTTGAA

GTGGGTGTTTTGGATTGGTTTGCCCGTCTATGGGAAATAGAGCAGAAAGA

ATACTGGGGATACATTACAAATGGTGGCACAGAAGGCAATCTTCATGGAA

TCCTGATTGGGTTAGTATATTCTAGCTTTGTTTCTCATTTTTGGAGATAT

GTCTGTCTGTTTAAGAATTGTTACAAGATATGCATAATTTGATTGTGCAG

AAGAGAAGTGTTTCCAGATGGAATTTTTTATACGTCGCGAGAATCACATT

ACTCTATCTTCAAAGCAGCACGGATGTACAGAATGGAATGCGTTAAGGTC

GGCACTTTAATCAATGGGGAGATTGATTGTGCAGATTTCAAAGCAAAGCT

ACTTTCTAACAAGGACAAACCAGCCATAATTAATTTGAACATAGGTATCT

GCTCTCTCTCTCTCTCTCTCTGTCTACTCGAAGCCATGCATGTATATGGT

TGTAGAAGCCCACATCTATACCATTAATATCTCCATAAATTGGTTCACAT

CTTGTTGTATTGCAGGTACTACTGTCAAAGGAGCGGTTGATGATATTGAT

CTTGTTATACAAACCCTTGAAGAATGTGGATTCTCGCATGATCGATTCTA

CATCCACTGTGATGGGGCTCTGTTTGGATTCATGATGCCATTTCTCAACC

GTGTGAGTTATTTGCTTGTCTCCACTTCTCATGTCCTGTTTGGGCCTATG

AATTTTTGAATTTTCAAGGAGATCTATCAGGAATCTCTAGGCACATATCT

TCTACAGGTTATAATATATTGTCTACAACAAATCACAATGTGTCACATTA

CACAATAGAGTAGAAGAGTTTCTATTCATATTTTCTGTAGTCATTACCAA

GAAAAATTAAGGAATGCCCAAGTTTATTCCACACTATAAAATCTTACTAA

GGTGTTAGACATGGTGATTTAGAAGGCTGGTGAACCCAATTGCAGGAACT

TCACTGAGAACCCTCTTGGTAATTTCATCTTGACCATTATTTGTTTCTTC

CTAGCTTGCCAGACCATTGAATTAGTTTTACAAAAAAAAAAAAATGGTTA

TGGTCCTAATTCATGGCCTGCTAATCGAGGATGGGAATATGTGCTTCATT

AGATGTCTCTTCAAATAGTTTTAATCTCCCAACTCTCCCTTTCTTGAGTC

TTCTTGTTTTCAATCTAATTCGAGCTGCTTACACTTTGTTGATGGACTGA

TGGACAGGGACCGAAAATAACCTTCAAGAAGCCCATTGGAAGTGTGAGTG

TTTCTGGCCACAAGTTTATGGGATGTCCAACGCCGTGCGGTGTCCAGATA

ACAAGGCTTGAGCACATTAATGCCTTATCAAGGAATGTCGAATACCTTGC

TTCAAGGGATGCCACAATCACAGGAAGCCGGAACGGCCACTCTCCAATCA

TTCTGTGGTACGCGCTGAACAGAAAAGGTTTCAAGGGGTTCCAGAAAGAA

GTCCAAAAGTGCCTCAGAAATGCTCACTATTTGAAAGACCGCCTTAGGGA

AGCAGGTATTAGTGCCATGCTAAATGAGCTTAGTAGCACGGTTGTGTTTG

AGCGACCTCTAGATGAGGAGTTTGTTCGGCGTTGGCAACTTGCATGCGAG

GGAAATATGGCACATGTTATTGTGATGCCTAATGTCACCATTGAGAAGCT

GGATGAATTCTTGAATGAATTAGTTCAAAAGCGCGCGAATTGGTACAACG

ATGGGAAAGCTGGACCTCCTTGTCTTGCACCAGATATAGGAAGTGAGAAT

TGTGATTGTGATCTTCATAAATGATATCTATAAGGGGGCGTTTGTTTAAT

GGGAAATAAATAGTGCAGGAACTATTTTTCTCAGGATTAACAGTCCATTG

CATTACAATCCCTCTTATATAATCTCCCCTTAGGATTTACTGCAACTGCT

ATCTGTTGGTTCTACTATCGTTTGTGTGTGCCAAAACAATAAAGTTCACC

AGTGATATCTTCAATTTGATATGTATTTTTCTTGTTATGCAAAATTTAAT

TATCTTCTGCAATTTGTGGAGTGTTATCAATGAATTTCTTCTTTACAACA

GATGAAAATCTTACTAATGTTCTTCAAAAAAAAAAAAAAACATCTGATAA

AATTTAGTAAATTACGAGTTTAAATTTCTGGCATTCAAAGATTGGTGCTA

ACGCCAGCCCAAATATTTCTAGCTCTAACACCCAACGTTTGAAGAAAGGT

AAGAGGAAATGGAGTAAAAGAGAAGGGCTCAAGTACTATTTGCTAGCTGG

GAAATTCTCTGGCTTTGCTAGAAGACTAGTGTAATCGGGTGCAACCTCAA

ATAAGGGACTTGTCAATAGCTTCAAATCTGGATCCACAACCGCAGCTTTA

CCTGTTTCAACTCTTAGTCCGAATTTACAACAATCTCCTAACCGTGGTCT

TGTTCAAGAGGCTCAATCCACTTTGCAAATGGGGCAAAGACTGGGTTTAG

AATTCCAGGGCCACGAGTCCGAGGTGATTAGAGAACTAGTACAAATGGAG

GAAAGGGACATGGAGCGACTGGAAAGAGGAAAGGGTAAAGTGGAATAATC

CACAATCACTGGGTGTTATCAGAGTCAATTTATTTCTCATATGAAGACAT

TAAGTTAGAATATCAGAGGGCTAGGAAGACATTATAAGAGGAGTAAAATC

AAAAAGCTGTTATTTGATAAGAAGATTGACATGGCTTTGATCCAAGAAAC

AAAAAAACCAAGTCTAACCAATCAGATGGTGAAAAGTGTTTGGCCAAGAG

ATAGGCTTGAGTTTATGCATGTGGATGCTGAAGGCTTTGCTGGTGGACTG

CTTTGCATGTGGGACCCGGACATATTTCAACTCTCGGACTGCTGTTGCAA

CAGGAACTTCATTCTCCTATCTGGTAAGCTTCTCAACTCCTTTAATTGTG

CTATTGTCAATATCCATGCCCCTACTGACATCATAAGGAGAAGGAAATTT

TGGGAATCTCTGCTATGGTTGAAAGCTCATTTCCCTATGCCATGGTGTAT

CGGTGGTGACTTCAATGAGATTAGAAATTTAGGGAAAAGAGTAGGTGTCT

CATTTAGGGATAGGGGTATGAGTGACTTCAACAATTTTATTGATAAATGT

GAGGTAGTTGATCTTCCTTTGCTAGGCAGAAAGTACACTTGGTGTAATGC

AACTGATGGCCACAAATGGAGCCGAATTGATAGGTTTCTGCTCAGCCCTG

AATGGTTAGAAAAGTTTAAATTCAGCCAGTGGGGTCTTGCTAGGGTCTCC

TCTGATCACTGCCCTATTGTACTTATAGAGGTACTAGAGATTGGGGGCCC

AAACCATTTAAGTTCTTAAATGCCTGGATGTTGCATTTGAACTTTGCATC

CTTTGTGGAACATACTTGGAGTCACTCACAGTTCTTAGGTCCTGCCGGCT

TTATTCTTCAGCATAAGTTGTACGCCCTCAAGTTGGCTCTAAAAATATGG

AGTAAAGATGTGTATGGTGATGTCTCATCTAAGCTGCATCTTCTTGAAAA

TGAACTTCATCAATTGGACTTAAAAGCTGAGATTATGCCATTGGTTGAGG

ATGACCTGAAGCAGCAAAGAGAGAAAAAGAATGAGATGTGGAAATTAAGC

AAGGAGCTAGAATGGGAATGGTTACAAAAATCTAGGCAGGATTGGAATAT

GAAAGGTGATAAAAATACAAGATTTTTCCATGTTATGGCTACTTGTAGAC

AAAATCGAAATTCTCTCAATTCCATCACAGTGGGAGATGTAGTCATCGAG

GCACCAGACCTAGTTAAAAATGAAGTCTGCTCATATTTCTCTAATCAGTT

CTCCGAGGCTTGGAAGCATAGGCCAGTTCTTGTTGGTGATTTCAAGAGTG

TACGGCTCAGTGACCACTTCCCTTTGCTTGAGGCAGAGTTCTCGGAAGAA

GAAGTCTGGGCAGCTGTAGCTGATTGTAATGGAAACAAAGCTCCTGGGCC

GGATGGCTTTAACCTGCTGTTTTTTCAAAAGTTTTGGAAGATGCTTAAGG

GGGAGGTGCTTAATTTTATGAAGGATTTTCACACCAGTGGCAGATTACCA

AATTGTTTCAACAGTACATTCATCACACTGATTCCAAAGAAAGATAAAGC

TGTCACTTTAAATAAGTTTAGGCCTATAAGCCTTGTTGGTTCTGTGTACA

AAATTCTCTCTAAAGTTCTTGCATCCAGGCTCAAAAAAGTTATGCCTGGG

ATCATTGGTGACTCACAATCAGCTTTTCTAGGAGGCAGGAACATTTTGGA

TGGAGTTTTGGTTTCTAATGAAGTTGTAGATAGTTGGCTCAAATCCAGGA

AATCTAGGCTGCTGTTTAAGCTGGATTTCGAAAAGGCCTTCGACTCGGTC

AATTGGGATTTTCTTTTCTCAATGTTATCAAACTTTGGGTTTGGTCCCAA

ATGGAATTCCTGGATCAAGGAATGTGTTTCTACTTCTAGAATTTCAATCC

TCGTAAACGGCTCTCCAACTAAGGAATTCAATCCTCAGAAAGGTCTTAGA

CAAGGAGTCTTCTTCAAGAGGCTCAATCCACTTTGCAATTGGGGCAAAAA

TTGGGTTTAGAACTCCAGGGCCAAGAATCCGAGGTGATTAGAGAACTAAT

ACAAATGGAGGAAAGGGACAAGGAGCGACTGGAAAGAGGGAAGGGCAAAG

TGGAATAATCCACAATCACTGGGTGTTATCAGAGTCAATTTATTTCTCAT

ATGAAGACATTAAGTTAGAATATCAGAGGGCTAGGAAGACATTATAAGAG

GAGTAAAATCAAAAAGCTGTTATTTGATAAGAAGATTGACATGGCTTTGA

TCCAAGAAACAAAAAAACCAAGTCTGACCAATCAGATGGTGAAAAGTGTT

TGGCCAAGAGACAGGCTTGAGTTTATGCATGTGGATGCTGAAGGCTTTGC

TGGTGGACTGCTTTGCATGTGGGACCCGGACATATTTCAACTTTCGGACT

GCTGTTGCAACAGGAACTTCATTCTCCTATCTGGTAAGCTTCTCAACTCC

TTTAATTGTGCTATTGTCAATATCTATGCCCCTACTGACATCATAAGGAG

AAGGAAATTTTGGGAATCTCTGCTAAGGTTGAAAGCTCATTTCCCCATGC

CATGGTGTATCGGTGGTGACTTTAATGAGATCACAAATTTAGGGGAAAGG

GTAGGCGTCTCACTCAGGGATAGAGGTATGAGGGACTTCAACAATTTTAT

TGATAAATGTGAGGTAGTTGATCTTCCTTTGCTAGGCAGAAAGTACACTT

GGTGTAATGCAACTGATGGCCACAAATGGAGCCGAATTGATAGGTTTCTG

CTCAGCCCTGAATGGTTAGAAAAGTTTAAATTCAGCCAGTGGGGTCTTGC

TAGGGTCTCCTCTGATCACTGCCCTATTGTACTTATAGAGGATACTAGAG

ATTGGGGGCCCAAACCATTTAAGTTCTTAAATGCGTGGATGTTGCATTTG

AACTTTGCATCCTTTGTGGAACATACTTGGAGTCACTCACAGTTCTTAGG

TCCTGCCGGCTTTATTCTTTAGCGTAAGTTGTATGCCCTCAAGTTGGCTC

TAAAAATATGGAGTAAAGATGTGTATGGTGATGTCTCATCTAAGCTGCAT

CTTCTTGAAAATGAACTTCATCAATTGGACTTAAAAGCTGAGATTATGCC

TTTGGTTGAGGATGACCTGAAGCAGCAAAGAGAGAAAAAGAATGAGATGC

GGAAATTAAGCAAGGAGCTAGAATGGGAATGGCTACAAAAATCTAGGCAG

GATTGGAATATGAAAGGTGATAAAAATACAAGATTTTTCCATGTTATGGC

TACTTATAGACAAAATCGAAATTCTCTCAATTCCATCACAGTGGGAGATG

TAGTCATCGAGGCACCAGACCTAGTTAAAAATGAAGTGTGCTCATATTTC

TCTAATCATTTCTCCGAGGCTTGGAAGCATAGGCCAGTTCTTGTTGGTGA

TTTCAAGAGTGTACGGCTCAGTGACCACTTCCCTTTGCTTGAGGCAGAGT

TCTCGGAAGAAGAAGTCTGGGCAGCTGTAGCTGATTGTAATGGAAACAAA

GCTCCTGGGCCGAATGGCTTTGACCTGCTGTTTTTTCAAAAGTTTTGGAA

GATGCTTAAGGGGGAGGTGCTTAATTTTATGAAGGATTTTCACACCAGTG

GCAGATTACCAAATTGCTTCAACAGTACATTCATCACACTAATTCCAAAG

AAAGATAAAGCTGTCACCTTAAATGAGTTTAGGCCTATAAGCCTTGTTGG

TTCTGTGTACAAAATTCTCTCTAAAGTTTTTGCATCCAGGCTCAAAAAAG

TTATGCCTGGGATCATTGGTGACTCACAATCAGCTTTTCTAGGAGGCAGG

AACATTTTGGATGGAGTTTTGGATTCTAATGAAGTTGTAGATAGTTGGCT

CAAATCCAGGAAATCTAGGCTGCTGTTTAAGCTGGATTTCGAAAAGGCCT

TCGACTCGGTCAATTGGGCTTTCCTTTTCTCAATGTTATCAAACTTTGGG

TTTGGCCCCAAATGGAATTCCTGGATCAAGGAATGTGTTTCTACTTCTAG

AATTTCAATCCTCGTAAACGGCTCTCCAACTAAGGAATTTAATCCTCAGA

AAGGTCTTAGACAAGGAAATCCTTTATCCCATTTCCTCTTTAATATGGTT

GTTGAAGCTCTTAACATCCTCCTCCTCCGAGCTCGGGAGTTGAATTTGCT

CAAAGGGGTCGTCATTGGAAATAATCAAGTTCAGTTGTCTCATCTCCAAT

TTGCAGATGATTCTCTTCTGTTCTGTGAAGTGGAGTTATCGGAAGTTCTC

ACTTTGAAAAGGATCTTAAAGTGCTTTGAAGTAGTGTCAGGTCTCAAAAT

CAACTATCATAAGAGCGTGCTTTGCGGGGTTGGTATTCCTGGCAATACTC

TAGAGGAGTTTGCATCTCTGTTGAATTGAAAGACTCAAAGTTTACCACTC

AAGTATCTTGGATTGCCACTGGGAGCTAATCCCAAAAGAAAAAGGATGTG

GAAACCAATAGTAGATAAATTCAAATTGAGGCTTGCCGGGTGGAAAAGGA

GGTTTCTCTCCCATGCCGGGAGGTTGACTCTGGTGAAGGCAGTGTTGTCA

AGCTTGCCTGTATTTTACTTATCTCTGTTTAAGCTACTTGAAGGCGTAGC

CAAGGAGTTAGATAAAATTCAAGCTTCATTTCTTTGAGGAGGCCCAGATC

TAAAGAGGAGAATACATCTTGTTAAGTGGTCAGAGGTAACAAAGAGCATA

AAGCGAGGAGGATTGGGTATCAAAAGAATAAGGGATATGAATGTATGTTT

ACTACTCAAATGGTGGTGGAGATTTGCCTCAGATCATGATTCACTGTGGA

AGAGGGTCTTATGTAGTAAATATAAGTTCCTAGGAGGGTCTTGGCTTCCA

AACCTTTGTTCTAGCATTCATCATTCTAATATCTGGGGTGGCATAGTGGC

AGCAGCAGATAAGCATGAGCCTCTCCTATTGTTCTATATTAGTAATCTTC

ATCTAAAAATGGGGAATGGCTGTCGTATTACGTTTTGGTACGATCAATGG

TGTGGGGGTATTTGCCTCAAAGATGCATTCCCCTGACTTTTTCAACTGTC

CAATAGTAAGGAGGGTCTTCTCAAGGACTTTGTGTCACACTCTGCTTCCA

TTTGGCTATTTACCTTCAGGAGAGCCCTCTTCGAATGGGAAAAGGATGAA

CTCCACAGGTTTATTACCACCATAGCTGCTCCCCCTACCTTCAATCTTGG

AGTTGCTGACAGAGCTGTGTGGGCAGCCCTTCACCCTAGCAAGTCTTTCG

TTTCCACTATTTATAATCATTCTAATCTTGCCTTGGGTAACATTGTTCAC

TCTAGTGATTTGGTTTGGCTCAACTATCTGCCTCCCAAAATACAATTCTT

CGGTTGGCTCGCTTGGAAACACAAGGTTAAAACTTTTGTCTTCTTGCAAC

AGATTGGTGTCTTGGATAGGTCGGCTTCCACCTCATGTCTTTTCTGTAAC

TTGCAGCATGAAACTGTCCAGCATGTTCTAATTCACTGCCCTTTGGTGTG

GAAAGTTTGGATTGGGTTATTACATTGGTGGGGTATTCTCTGGGTGGTTC

CTGCCTCTGTTGAAAATCTGCTGCAGTGGTGGGCAGGGTTCAATTGCAAG

AAAAAGGAGAGGAAAATCTGGCATGCCATCCCTTTGGTCACCCTCTAGTC

CTTTTAGAAGATCAGAAATGAGTGTCTCTTTCAAGCCTCCCAACTTAACA

CCCAGCAGCTGCAGGAACTCATCATCATCGGGTTAGCGATATGGTTAAAG

TCCTCTTCCAAACACTTTCCATTCTCCATCAATGATTTTCTCTTCAACGC

TTCAGGTGTTAGGCACTGCATACGCTCTTGAGTTGCTATCTGATATGTTC

AGGCTTTCTGATATGTTAGGCACTGCATACGCTCTTGAGTTGCTATCTGA

TAAGTTCAGGCTTTCTGGGTAGTGTAATTCTGCTGTGTTTCGTTTGCTGT

TGTCTGTGTAATTCTGCTGTCTGTGTGGTTTGGTTACTGTCTGTGCAATT

CTGCTGTGTTTGATTACTGCTGTCCGTGTGTAATTCTGCTGTGTTTGGTT

TGTTCAAGGGAGTGTAATCTGGTTCTTGGCTGCATTTTGTAATCTGGTTG

CTGCTGTAATCGGTTCTGCTTCTTGGCTGCAGGTGTCGTCCATGGCCAGC

TCAGTTCTGTTTCAGTTCTGTTTTGGTCCTTCTCTTCTCTATTTCTTGTT

GGCTCCTTGCCAACTGGAGCTCCAAGCTTGTGTTAG

>TEA024083.1 locus=Scaffold1689:1997089:2034391:- Protein NRT1/ PTR FAMILY 8.3-like isoform

ATGGGTACCAAAGAGCATGAGAGATTGGAGGAGAGATTGCTCTTGGAAGA

TGGATTTCCGCAGGTACTTTTGAAGCTTTGATTCAAATTTCTAGAACAAA

ACTTATAGATGATGTATTGCAAAACTTTCCTAAAACTTTTGACTGATTTT

TGAAACAGATTCAAATTTCAATGGAAGATGGATTTCCAATATTTTTTGCT

TTTTTACTCTTTTTATAGATGCATAAGAGCTTTACCAATCTTTCTTTTTT

TCTTTTTCTTTTTTTTTTTTTCAAATTTGAGTGAAAATTTAATTAAAAGC

TTTATATGATATGAAAATTTGGCTCAAATTTAATGAAAATTTTGTTAAAA

GCTTTACAGCTTAAAATAGAAATGATTATAACTTATTTTAATTTGGAGTT

TGGTACTGTTTGGTAGACTTGTGTCTGTAGTTTATATGGCTTATATAGGC

AGTACTAATTTAAGAGAAAAGTTCAAAAACTATTGATTATCTAAAAGCAG

GAGAGAGCTGCTTTTAACTTATGGATTGGTTTAGGAAGTGGGTTGGTTTT

TGGTTTTTTGATTTTTTTAGTTATTTTAGTTTGGATTAAAAAGTTTGATT

TGGAAAAAAAAAATTGACTTTTTAATTTTTTTAATTTTTTTTGTGAGAGA

AAAGTGTAATGATTATGAATTTGATGTGATATTTTATTTGTTATACCTAA

AATAGTCTATTTTGTCTTTTGTGACTATTTTAGTCATAAACCATCTTACC

TTGTTTAGCTGCTTGACACATGGAGTGAAAATTGAATAAAAGGAAGGCAA

GCTGCGAAATATTTTTTTTATAATTTTTTTTTTCAAAATTTTTCTTAGAG

AGAGAGAATATAGATTTATTTAATTATATGCATGGACTGATTAAAAAAAA

AAAACTATTATATATAAGTACTTATATTGGCAATTGGCATTACATGGCAG

AGCTAATTTTAACTCTTTTTTTTTTTTTTTTTTAAATTGCAAAACTTATC

CTAAAAATCTGTTTTAACCATTTTGAAAAAAGAAAACCATCTTCAATCCT

TAGTCCTTTTAAAATTTCATCAAAATTGAGAAAAATTATGAATTTTGGAA

ATTTGGCGAAGGATTTTGTTCATCCTTGATTTAGTGAAAAAATTGCCTCT

ACCTTGATTTCATACGGATTTGATAAAATGGCTGAAACGCACTTTTAGCC

AATTTTTCATCCAATTTGAATTTTATGAAAAATTTGATAAATGATTTGAA

GATGCTCCCACTCGAAGAACAGGGTTTCCGTTTTAATCATTTTGAAACCT

TTTGAATTGATTCTGGTTTAGTTTTTTTCCTTTTTCATGGGCAGGATTTT

CCTGTATTGACGTCTGAAATTTCAGGCCCTGTAATGGGAGCATTGTTTAA

TTTGTTTTGCTCCCACTAACAAAAATTCTGAACTTTGTTCTATGAAGACC

CGGTTTAGGGGAGTAGCCATGGAATGCAAATATGAAGAGAGATAGGAAAC

CTATTTTGGTTGAAACCCATAGTTCAGTTATCCTAATTTGTGTAGAGACC

GGAAATAAAAGTCCTGAATTGGTTGCTAGAACTGAATACCAAAATCAGTT

TGATGGTTAAGCCTACTTCATTAATACCATGCAAATGTTCCGAAGGCATA

CCATGTAGGCAAATACAATAGTTGCTGCAATATTTGAACTTTATAAAGCT

AAAGCATTCTAAGAAATGTGAACTTGAGAAAGTTTTTGATTGATCGGTGT

AAGAGGGGGTTAACTGGAGCCCATTAAATTAATTAGGCTCAATCCAAGTT

CAGTCGGAAAGCCCAAAATTAGTGGGCTAAGAGAAATTATCGCCAAGTTG

ACTAAAGTGAATTCTATTGAACGGACCCGCAAAAAAGTCAACAAATTGGG

CTGAGGAGGACATATCAAACTTGTATATCTAAAGAGAGCCCAACAAACAC

ATTCGAGGAGGGCTAGATCATATGGGCATTTGGAAGAATTAACTGGCGCG

CACTCTTGAAGATAGATCATTTTCAGAAGAAGTGAAACCTTCAGAGGAGA

TCCATTCTCGAAGGTAATTGCTTTGTCTCGAAAAGATTCTGATCCACGAA

CGCAGAGGCTTATTGAGCTTGGACTCGAAAGGGTTCTAAGCAAACCTAGT

GATCCGATGCATTTTTTACTTCAAACGGATTCAAACTCAATCTAGAAGTC

GCTCCGTTAATGAGTCTCATAACACTTGCATGCTTGAAGCTTTTCGGGAT

CGATGTACCAGAGCCATAGGTGACAGAATCCAAACAAAAGACCCAATCCT

GAGGACGGTGGATGGAGATAAATTCACTGTTCGATTAAATTAGCCTATAC

TTGGGTCGAGGTGGCACGATCTAGAGGTTTCATAAGACTCGGCGATATTT

GCGGTAGGGCGTTCTCTTGAACTACCTCTGATCCTTTGGCGACATGGCGC

CCTCTGGGATGATTGGGGATCATTGGTTAGATGTGGCTAAGAAGCAAGGT

TTACTGAGTTATTGATCTACTCTTTGGGATTTTTCTTATTCTCGGAGAGG

TCACCTCTTCGAACAAAGTGGTTAGAGAGAATATTTTATCTTATATCATG

AGGTGCTTATAAATAGCACCCACCCATAACTTTACAATGGAGATGATTTT

TGGAGAGAATTTTTGGAGAGAGAACTAATCTCCCTTCTTCATTATTTACC

TTGAGAGTCAAAGTGGGCGATCCATGGGAGAGCCTTACCATTATTCTCTT

TCTAACTACTTTGCCTTTTTCTTCACCCAATCTCTTATCTCTATCGTCTC

TTTCATATTATGTTTCTAGTTACAACCGCTGAAAAATCTAAGCCTCTCTT

ATTATTTCTAGAGAATTCGGATTTTCCGGCGATGTTCGAAGATCTTCAAA

TTTTCTGGCAAATCTTCGAATTTTCCGGCAAACCTTCAAAGTTTCCGGCT

ATTTTTCTGGCGACATTCGAATTTTCCGACAACATTCTTGGACCCTCACA

TACAATCGGAAATCAGAAATAGTTGTACCAATATGTCAAACAATTAATTG

TTAACAAGACCTATCTGGTGGTGAATTTGCACATCTAACACATTCAATGT

TTTCATTCTTGCTGCTTTGCACATTGTTCATTTTGTTTCAATGTATTGGA

ATGACAAATAACATTATTTACAGAACTTAGCTACTTACTAGTAGTTGTTA

CAGTAGCTGCATATTAAGGTGGTTCAGACTAGATTGTCATCTCCATGCAC

TAAACATTATATAAGGATGTCAAAAAATTAATAATAGGTCAGAAAAACTA

CCCAATTGTAAAAACTTAACAGACAAAACTCTCTCTCTCTCTCTCTCTCT

CTCTCTCTCCCTCACACACACACACACACAACACATATGCACACATAACA

CAGGAGATTACTCTGCAAGGTTTATATACTAAAAAGTTTCTGCTTTCTGC

CTTACAACTTACGTCTTCAGAATGAAAGCAGTGGACTATACACTGGAGAT

GGTTCAGTTGACATCAAGGGGCAGCCTGTGTTGAAGAGCAACACTGGAAA

CTGGAGAGCATGCCCCTTCATTCTTGGTAGTTGCTAGTCCATCATTCTCC

ATATTTGTTATGTTTCTGATGTTCCATCTTTCCTTCTAATATCTTTATAT

TTAAAACAGGTACTGAATGTTGTGAACGTTTAGCTTTCTATGGGATTGCT

GCTAACCTGGTCAGCTATCTCACCAAAACAATGCATGAAGGAAATGTCTC

TGCTGCAAGAAATGTTTCCAATTGGGCAGGCACTGCCTACCTAACACCGC

TTATTGGAGCGGTCTTGGCAGATGCATGTTGGGGAAGGTATTGGACAATT

GCTGTCTTCTCCGCAATTTATTTTTTGGTAAGTATGAACTGAAGTTCGCA

ATGTTCTAAATAAGTTCAAAAAGAGATGGGTGATGATACATAAACAACTT

CAGCTTTTTAATCATTGCTTTGATCCTTTTTTGTGTTGGGCATGCATTAC

GTCATCTTAAAGTTTTCTGATAAAGGCTTGCTTGTTATTTGAATGTTCTT

GTAGCGTCTGATTATGTGTTACCTACAGCTTTCTGTTGGAACCATATTGG

CCTACTGTAGGGCCACAATCCATTCTAGATGCTTGTGACTTTATTAGTTA

GTGATGGTATTTCCTAGGTGTACCCATCATCTAGTCTGGTATCACTTGAA

TGTATGAATCACGGTGGTAGTGGTTTCAAGAATAATGAGTTAGCAGTCAG

CAACTTTACCAACAGCATGGTGCAATGGCAATAGACCTACTTGTGCCAAT

TAAACTGGGGATTTATCTATAAGGATAAAAGGACCAATAATCTGGACATT

TTATTAAATCAGGAACGGGAGAAGAATTATATTTGATGAAGGTTGTTTGA

TCGGTTGACTACCCTCTTTTGTGAATTTTATTACTTTGTTTTAGCCTATC

TTCTTTTCTGATAATAATTTTTTGGTAACTCTCAATTCTAGAATGTTTTA

TTTCACAAGTACTTTTATTACTTAATTTGAAAACAGGAAAATAAGTCGGA

ACAAATTTATGGACAAATTTAATGTTCTGAAAGTGATTTGGATCATGTCC

CAGTTGGGGTTTCCATATTAATTATGATCCATGCAACTTCGTCGGGTATT

TTTTTTTTTTTGGGGACTTTCAGTTAAGTTCAAACATACCATGGCTAGGT

ATGTTGGCAATGATGGGAAATAATTACATGAGTCTTTTATGTGTTCTATC

CATTGTTGTCGAATTGTGAATTGTTTTGCTAAAATAGTGAATCGTGAGAT

TCATTTTATTTAATATGATATATGATTTTATATATTTATATACATATAAA

AATACACGTAATATTTAAAATGAAGTAAATATCAATATAAATAAATTGTG

TATATGTATATACATATAAAATCATATATATCAATATAACTAATTGATCT

CTTCTATATATCTTGATAGTTTGAGAAAAATGACATCGATTCGTGCATTA

TGTAAACAAAAACAAAAATAAAAATGAATCTCAGTGAATTGCAAATTGAT

TCGTACAATTCACTCAATTTCCGATTCAACAACACAATTCAAATCGTTTT

TATTGAAACTCATCTAGAATCGTATGACTCACATGAATCAAATCGTGAGT

CATACGATTCTAGAAACCATGGTTCTTTCCTCTCAAAATTTTGAAGAGTG

GCTTTCATCTATATCTCTCTTACGTTTTTGAACTTTGTTTTTTTGTAAAG

GGAATGTGCACATTGACTCTAACAGCCACAGTTCCTGCGCTTAAGCCAGC

TGAATGTGTTGATTCCATATGCCCTCCAGCTACTCCTGCTCAGTATCTGT

TATTCTATCTTGGGTTGTATTTGATTGCACTAGGAACTGGCGGGATCAAA

CCATGTGTTTCCTCATTTGGGGCAGATCAATTTGATGACACCGATCCTAA

AGAGAGGGCAAAGCAGGGATCTTTCTTTAATTGGTTTTATTTTTCTATCA

ATTTAGGTGCTCTTGTATCTAGTATTTTAATTGTGTGGATTCAAGAAAAT

GCTGGGTGGGGCCTAGGTTTTGGCATCCCTGCATTGTTTATGGGCATTGC

TATTGTAAGTTTCTTTTCAGGCACTCCACTATATAGATTTCAGAACCCGG

GGGGAAGCCCTCTTACAAGACTTTGCCAAGTTTTGGTTGCATCATTCTGC

AAGTGGAATTTGGAAGTCCCTGCGGACAGTATTCTCCTGTATGAAACACC

AGACAAAACCTCAGCAATTGAAGGAAGCAGGAAACTAGACCATACCAATG

ATCTGAAGTAAATCTTTCCCTTGTTCTCCTAGTTTAAACACTTCAATCAA

TTCTTACCCTTTTTTAATAATCTGTTTTCAGTTTTCTTCTGCCATTTAGA

AGCTTATCAATTGATGTACAACTTATACGTATTTATAGGGTGTTTTGGGC

ATTGCACATAAGTAAGGCCTTTTATGTTTTTGCATTGAACACACATGCTG

GTTGTGTAAATGGGATCACCACCATAATTATTCATATACTGATAGTAATG

AGATTTTATGATGCTCTTTTGTGAAGTTTTGAGAGTCTTGGGACTAGGTA

ATAGGTTTAATGAATTACCTTTAAAATTACCGGGTTTACCTCAATTAGTG

TATGAAGGCACAATAGGAAGCTTAATATCCATTAGGTGTCCTCATTTATT

TTGTCATGGTTCTGTAACGTTATACTGGGATGGTAATTATTGCTTTATTT

AATGCAAGTCATTTTAAAAAAAAATATGTTGTTTATTTTTATTTTCTTTC

ATTTCTTTCAGTCTGGTTATTTAAGGCGTTATGAAAAAAATCATTGTTTC

ACAAACAAATAACTTAATTTTTGAGCTTGGATTCAAAAAATATTATTTCA

TGTGAGATGTACATGAAATGCAATCTTAAAGTGGAAGATGGAGGGGTTTC

TAGTAATGTGGTCTTTCTATGGTTAGTTTTATGAATTCCTTTCACTGAGT

CCTCTCACTAGTTGTTTATTGAGATTGGCTTTATATCCTTATCATCTATG

GACTAAGAACAAGCATTGGAAGGCAAATGAAAATTACTGGGAAGATGCAG

CAGTGTGATAAAGTATGGTATGATTTGTCAGCACAGCAGTAATAGAAAGT

TCACTGATCTATTTCATCTCTTTAAAATAGTTTATTTGTGTATACTAAAA

TAAATCTTAAATTCCTGATTTTGGCAATTGTATCATCCTAATGATTTTCT

TTAAGGACACTACTAGTGACAGGTTTAGGGGGAAGGAATCTGTTTGATTT

TTCTAGAAATAACTGTTCATTTGTCCGTTTGATTGGAAACAGGCATGAAA

CAATAGTGTAAAACCCAAGTTCCAATATTTCTTTTTTCCTTTCAGCAACA

TGTCCATTTCCTAGTAGACCAAAACCACACCCAAAATGAGAATTTGATCC

TTCTGTTGGGTTTAGGGAGGGGTTGGTACTTGGACTCCTCAACACAATAA

AATGAGAGTTCACCACTACATTTGTCTTATTTTTATTTTATTTCTAATCA

TGCTTTGTATGATATGTTTTCTCTTGGCAAAACATGACTTGTTATGTATG

AAATACTTATAATTATCTTTGGTACTTCCATCTTTTTTTTAGACTTCTAC

AATACTTTTACTTTATGATAGTTTGTATACTTGATTAATTCTCACCAATT

TGTTTCCCAGATTATTCACAACAAATTATGTCATAACTTCAGTAAAATTA

TTATCCCTTTTGTAGGTGCCTTGACAAAGCTGCGGTAATTACAGACATTG

AGAACAAAAATGGGGACTTCTCCAATCCATGGAGGCTTTGCACCGTAACG

CAGGTGGAGGAGCTAAAAATATTAATTCGCATGTTTCCAATTTGGGCTAC

TGGAATCATCATATGTGCTGTTTATGCACAATTGGGTACATTGTTTGTTG

AGCAAGGGATGATGATGGACAGGACCATTGGTTCCTTCTCCATTCCTGCA

GCCTCTCTTTCAGCATGTGACATCATAAGCGTTATTTTCTAGGTCCCCGT

TTATGATAGGGTCATCGTCCCTATTGCAAGGAAGTTTACTGGCAGAGAGA

AAGGCTTTACAGAGTTACAACGTATGGGAATTGGCCTCTTTATTTCAGTA

CTATGTATGGTATCTGCTGCTTTGGTGGAGATGAAGAGATTGGCACTTGC

ACAAGCCCTTGGTTTGGTTGATGAAAACGTTCCAATACCACTTAATATCA

CGTGGCAAATACCCCAATATTTTTTGTTTGGTGCTGCAGAAGTATTTACA

TTCATAGGGCAGCTCGAGTTCTTTTACGAGCAGTCACCGGATGGCATGCG

TAGCTTGTTTGCTGCATTGTCCCTTTTGACGACTTCCATGGGAAGCTACT

TGAGCTCTTTTATTCTTACTGTAGTGACATCTATAACAACCGAGGGTGGG

AAGGCTGGATGGATACCGGATAACTTGAATGAGGGCCATCTCGATTATTT

CTTCTGGCTTGTGGCTGCACTCAGCATAGTAAACTGGGTGGTTTTTGTCT

TTTGCGCCACAATGTATAAACCAAAAAAGTTCTCGTAAGTATTTTAAGTG

CTGGGAATATGATCAGTGTATGTTTCTGGAATTTCATACTTCCATCCAAG

TTCCAAGAAGATGGTTGTAACATAAATGTTGTAGAAAAAGTATTTGTTTG

TAATGTAAGTACGATGCATTTACGTTTATAGGCCGTAAAACTTTCATTCT

GTCATGATGTAAAATTAATAAAATAATACATGAAATTGTTTGAATCTTTC

TGGTTATTTGCATTCACATTTTGTTGGTACACTACAAGAAAAATGGGTTT

AAACGGTGTTTTTTTAAATGCCACGTTTATTAATATTTAATGACGTTTTA

TAAAAACGTTGCATACTCCTTTAATAAAACGGTACCGTTTTGCAATAGGA

GGCGTTAAACGCCTCCAAATACGTTGTGCCTCACGCGCCTATTTTAAATC

TGAAAATACTTGTTAAAGCTTTGTAAACTCCTATTCTGAATCCTCACGCC

GTCACAATCCTCACGCCTCTCTCTTGCTCACCCCCAATTGCTTCTAAACC

CTTGAGAGCGATTTTGATTCAGAGCGATTTCGTCAGAGCGATTTCGATTT

GAAGCTTTGAAGCTCCACTGTCTCTCTTTCTCTCGATTCATCCTTTGTTC

TCTGCGATATCGATCTGAAGCTCTGGGAGATTGCGAAGAGAGAAGAGGGG

TTGGTTTTGATCGCAGAGGAAGTGGGAGAGGCTGTGCTTGATTTCTCAGA

TCGGTCTCAGAGCGATTTCGATTTTGAAGTACTCTCTCTCTCGATTTCGA

AGCTTCACCGTGAGGTATTGATTTCTCTCTCTCTCTCTCTCTCTCTCTCT

CTCAAGCTCTTCCTTCCCGTCTCTCCCGATTTGGTTATTGAGATCTATTG

CTTATTTGTGTTGTGTGTGTGAGTGTATATAAATGTTTGGTTTCTTCGTT

TTTTTTTTGCCCCACTATGTCACTGTTTAGCTTACCTGAAATCTTAGTTA

CATTTTTTCTAGCCGTCTGATATGTAAAGTTTTTATTTACTCAAACTCCT

TATTTATCACCTGTAGAAATTTAATGTATAAAGAGAAATAGAGAATTATA

TTATTGATTGATATCTGTTTGTATACAATGACTCTCCTATATATAGAAGA

GAAAAACATATTTACCAACCACACTCAACTCTCACCTAACACACTTACTT

CTGGATTAGTTTATACAAGAATCTTTGTGTATGTACTGTTTTGTTAATCC

AAAGTTGAATCTATGGTGTGGATTAGATTTGGAATAAAGCTGTAAAGTTT

GTAACATTTTGTGCAAAAATCTTGGTGGGTTCTATAGATTAGGATTGCTT

AAATTGGATTTGTTTCTTATAATGGAAGTCTGGCACTGTGCTCTAGTATG

GGGTTTAGTTATAATTTGTTTGTTTCTCCAATTACATTGGTAGCTTAAGG

TCATTTAACAGTAAATTGTAGTCATGTACGTGACTTAGTTAGCTTTCTAA

CTTAGCTGTTGTTTGATGTGTGGAGTTCTAACATCAACTATAACTCTGAT

AGGTTTTTTGTAGCCTGACTTGGTTTCATCAACCTCTCTCCTGCTTTCTA

TTGAATTTGCAATTACGTGCCCAGGTATAAAAATCGGTTCTAGAATTTTG

TTAGATATCTACGGGAAATGGGAGATGAGGTAAATTCCAGAGGTTCTGAT

ATTGAAATTTTGTTTCAGGAGTTCTGATATTAAAATTTTGATATTGAAAT

TTTGTTCCAGAGGTTTTGAAGCTTGGGAACTCCACTCGGATTAATGAGCA

GTGCTTGGAGCTTTAAAAGAATATCAAAGATGATGTTTCCAAAATGAAGG

TAGGTATGATGGTGGCTGCTTTCCTAAACCTTGTATTCATCTTGTGAAGC

ATGTTTTCGGGCTAATTTGGCCTGCATTTGCTTTCCTTTCATCGTTAATA

CCTTAATGATTTCATGGTTTTGGCAATGCAAATTATTGTGTTAACTTGAT

AGACAATATCACTCCAAAATCGCAATTAAAAGTACAATGATTTCAGAGGT

TGCAACAATTAGAACAAGAGATCAATCCTGTTGCTTGCTTATTAACTTTC

ACATAATTCATCGATTTGGTTAATACTACTAACCTCTGACTCAAATATAT

TTTTCATATTGGTTGCTAAATGCTAAGCAATGCCTCTATCTCTTTGGTGA

TTTTTTTATTCTGTTCATTCATAATAGATGATCTAGTTGATAGTATATTG

TGATTCTCTTTGGCATCTGAATGGACCAATAGGGATAGTAATAATAGTAG

TGTTGTAATGTTTTCATTCATATTAAGAAATTCTCCTTTTAGGTTAAATG

CAATAAACTTTCAAACTGAATTGGTAGGGTAGAGTTGAATGATGAAAGAT

CAATTTTGTCTGTTGAATTTCTATAAGAGATATTAAGAACAACCATTTGC

TTACATTCAAGTCTCCATCTCTGTTGAACCGAATGTGTTTTGTAGAGTAG

CCATTGAAGGCCTTTCAATGCATTTTTGTTGCTTTCCAGTTCAGTACTAT

TATTTAATATCAACCAAGTGAGCAATTACATCTATGGAGAAGCTGAGGCA

TTGACTGATGATATTGAAGAAGAGAGTCAGATTTTTGGGGAGGTCTTGAG

GATCAAAAAGATTATTGATAATTGTCAAGATGAGGTTTATCTTCATAACT

TCTACTAAATCTCTCTTTTATGTGAATTTAATTTTCATTTCTGTATTTTG

TTGCTTTTGATTTTTTTTTTTGGGTAAGAACTTTGACTGATAAATGGGAG

ATTAAGCAGAAATTCCTTGTTATATTCTGATCCCAGAGTGTGCAACTCAT

CAATGAACAGAATAACGACCCCTTTGCTTTGCTTAACTTCAACAGCCAGT

AAAATGCTTAATACTACTTTCTGAATGAAACTCAAAGCCAGTAATTGAAA

GCTGTACATAACAGTGTCATCCAGTTGTCATTGTTTTATATATCATAGTC

CACATTTATTATTTCAGAATATTATCAAATGCACATAAATGTAATGATGC

ACTTTCTAACAATAATGTTTGGCATTCCCTCTCTCTCATGAGAACATTAC

TGAGGAGGGTACAGCTTCCTAAGAGTTTTTTCACATATATGAATGTTTGG

CAAAAACTATCTTTACCTGTGGGATTGAATGTATCTAAGTGATATCGTTA

CATGCTAGAACCCACCTGCCTGCATTTGTTACCTACCTGCTGCATTGTAT

AAGTTTCAGCATTAAGTTCCCTCATTTGTGCCGGACATTTCTTAATATTC

TTCCTTTTCAAATCGAGTGTAACAGGTCATCAGAGAAGAGAGTGAGTGCA

GTTGCCATAAGCAATGATGGTCTATTTGTTTGTTTTGCTGACAAATTTGG

AGTTGTTTGGTTTGTGGATTTGGATGGATTTCATGAAAATCAAGCTTTAG

CCGGTAACAAGGCAGCGCCAATTCTTGCTCACTACTGTAGCATCATTACT

AGCCGGGTATGTCATTTGTTATCTTTGATATCTTTTCTCTTTGTCCTTGC

TCTATTGTATTTATGGCCTTTTGTGATGACCTCTAGTATAACAACTCATC

TTTTTCCATGTCAGTTATAATGCTAAAACCATATCATAATTATAATGCTT

AAAAAACAAAACTTTATGCTTTTTTGATTGGCAAAAACAAAACTTAATTA

GATTCTCATTATACGTGCAATACTTGGTTATGTAGATTTGTACTCAAGAG

TTGTGGATCAGATGTTTCAGTTTATTGTCCTAGACATTTTTGCTAAGTTT

TATTTTTTAGTTTTTATTTTTGGTGAATCATTGGTTATTCCCTCCATTTT

CCCATGAATAACAGGAATAAATTGGACATAATAGATTTTATCATTATCTT

TTTCCTACAATTTTTCCTGCCAATGTTTTTGAGGTTTATATATATATATA

TGTATTCTTTCTCTACTCCTATACACATTGTTAAATTCTTATGAGTAATT

ATATAAAAGTCTTTGAAATTAGGGCTACATTTGTGTGGATGCATGAATGA

CCTTTACTTGTAAGCTGGGCTTTTTCTAAACAATTTTATGTTCTTGCAGG

AATTCTCGCCGGATGGACGATTCATTATTAGTGCTGATCGGGATTTTAAA

ATTCATGTAATTGATGATTCTTACCCATTGAAACAAATAAATTATTAAAT

ATTAAATTTCATTTCAATTTGTTTTTAGCAATGTTATTTTGTGACAGGTT

ACCCTGCTTCCAAAGAAGCCTTTAGATGGGGCTCATGAGATACAGAGCTT

TTGCCTTGGTCATTCTGAGTGAGTGAGCCCAAATATTTTTTAGGATAGCT

GCTGTGTTCCAATCAATAAGACATTTAAACCCCAAACCACCTCCCCTTTT

AGGAGCAAAAATATGCTGTGAACTAATCTTAGCATGGCCTTTTAGCTCCG

AACCAGTCCATAATTGGCTCTACTACAAAGACAATTCAACAAGGTTAAAC

TATTGATTAAAAAGAACCAGCTCAATTATCCTAGTTTAATTCCTATCTTA

GCAGAAGCTACTTATAAGTTTAAATTTATGTTCTGTACAGAAATGCTTGT

TGCAAATACCTAATCAGTTTTCTTTCTTCTATTATTAGTTTAGTAGCAAA

AATGACTTCGATCCCGTGTTGCTTATTTTTTTTTTTTTTGCTTTTGCTTT

TGCTTTTGTAGATGGAATTGTTTTGAAATGTGTTTTAAAGGTGAATTTTG

GCAAAGACTAGACTCAATTATTCAATTGATTGTAAAACATTGCCAGCTTG

TTCCTGCTTAATTTTAACACCCCAGGGTATGATTTGTACATTTGAGGTTG

TAGATGTGCAAATATCATGAGCTTTCTTGTGACTTCATCTTCACAAATAT

AATAACTATGTATTCTATTTTTATTTTTATAAATTTTGTTGTCTTTATGG

ACATGTAGGGTTACTTTAGTTGCAATTTATTAGTAACTCTATTTTTTGAT

GATAAGAATGACAGACCAATATGAAGAAACATACAACTGATGGAAGAGAC

TAAACAAAAATAAGAAAACCATGTTACTTTCTGCCAAATGAAGAAACAAA

ACTCTAGCAACTCTGTTCTAGAAACAAAACAAAGCTACTACTGATGAATT

AAAATCAGAAGGACTACTACCATTTCTCTACTCTTGCTGCGTTTGGTATG

TGACATGAAGCTTACTACTATTGTTTTGTAGGTCCTAGCTCAAGTCAAGC

CACACAAAAGTAGACAAGGGGTCCCACATGAGGATCAGTGGCGGGCTTTG

GTCTACTATTAGGGCACTCAAAGTGCATCGGTACAATTTTATTTATGTGA

AAAAATGTTTATATATGATAATTTTTTATAACATCTTAAATAAATTTCAT

ATTTGACAGAAATCAAGCAAGAAGAATAGAAAAATCCGGAAGAAGAAGAC

ATTACATCATAGAACAGGCCGAAAGCCTTTCTCAGTAGTTCGACTGGAGG

TATAATTATTCTCTCCATTCATCATTTTCATTCTTTGTCAACTATATCTG

TCTATATGTGCTAAAAAGGTGTCAACAATTTGCACCATAATGAATTTATA

CCAGTTCTACAAAAACAGATGTTATTTGCTTAGTTGTTTATTTTATTTAC

ACTTGTTCACATATTTAATTGCTTAATTATTTAATTTTCTTATTTATCTT

AAGATTTTGGTTGTCTTTCATTTATAGGAGACCAAAAAGAACAATGGGGT

TCCTGCTAGTAGGTCACAGGTGTGGATGGCCGCATACATGAAAGATGGGA

CGTCAAATAGTGATTCAGTTAATGAGGTTATGGTATGTAATACTACTTTA

TCTTTGGACTTTTAATATGATTTATATTTATATTGGAGGCTAATTATTGA

ATTCAAACTTGCAGACTCAAATGAATGAGTTAGCAGAGCGTTATTGATTT

CTTTTGGGAAATTACTTATCAGTGGCAAGAAGAAAATTATGTTGGGAGAA

AATGGTAGGGCTGAGTTGGTATTATAATAGTTTATAAGAATACCGAATTA

CAAGCCGCCATCCTTTTTGGTTCAAGACTCTATATTTGTCTACGTTGCAT

TTTCACGTGATTGTTGGATTGACAGATCATGAAATAGAGAGGAAGATAGT

AAATGGGTTACCTTGATTCTCTTGAAATGATTGCTTCTGGCTTTTTGTTC

ATTAAATGTTACACGTAAGGTTGCCAACTCCTGTTTGACAAATACACACA

TAATAATTGAAAAATTAGGATTAATTTCCGATAAAACAAATATAGATTTG

AGTTCAATTAGATGCTTTGGTTGCTTACCTTGTTCAAATTTATCCTTTGC

ATGTGTGCATATGTAATAGCAAAGTCCTGTATCTAATCCCTCTCTCCAAA

CCAAAGAAGATTAAATTGAGAAGTCAAAATCCTCTAGCATAGTTTTTTGG

TAGAGTAATTTTTTTATGCCTGAGATTCAACTATAAGCTTGCTAATAAGA

GGGGATGGGATGGGCCCTTTACGTAGAGATATGTGGATAAGTTCAAGCCC

AGCATAAGGACATTAGGCCCAGCCTTTAGACCCACCAATAAAACAGAAGA

AGAAAAAAAAAAAGTTTAAAAGGCAAGAAGAAAATGGTAATAGAGTTTTT

TAATGTTGGGAGTGTGCTACAATGCTAGGGCTGAGAGTAGAAAACGACAT

TGTTTAAACAATGTCGTTGTTGAGTGTAGGGGCAAATGGTTCATTCAACA

TCAGAAAACTATGAATTGATAGTAAATTTCCTACTCTCCACTCCTACCAT

AATTCTCAATCTTTTTCCATTTATTTGTAACATAAAATTGCTGTTGTCAC

TGGGAAGTAATTCCTCCACATAACTTTTCTGAATAGAGATAGGGACAGAG

GATCCCAGCCCGTCAGACTTTAAGTCTTGGTGCACGACAAGTTTGGATCC

TCTGCCATTCTATCCTGTGGTGCATCCTGTTGTTGTACCAAAACAACATC

GTTTTGGCCATATATATATATATATATATTCTTTTTTGTCCAAGCTCTCA

AACCCTAAGTACTAACATCACTAGTTTTTAATTTCTCACTTTTCTAAATC

TATTAAGACATGATGTGAATTGTGGAAAGAGTTCACACTTGATCTGGTCT

ACAAGCAGCATGATCTGCAATTTCTCAATAGCGTTTTTATTTTTATTTTT

TCTAACATCTGAAACATGCCTTTTAAGTAGTTTAAGATGCAGAACCATGG

GAAGTTGAATGAACTGTGAGGGAAAGAAAAGGTGGTAGTACAAAGTTGTG

GCCTAGCAATTGGTAGGTAGCATAGTGCTAGAATTGGGCTTGTACACTTG

TTTAAATGGGTATTTCAAGCTTCAATGATCTTGTATTCTCTTTCCTATTT

TTTTATGGGTTTTGTAGCCTCTTGCTTGGATTCAAGCTCTCTTGTGTGAA

ATTGATCATACGTTGAAGAAATTGTTGGCAATTTTTGCAATTTTTTTAGA

AAGAATGGTGTTTGCCGAATTGGAGAGCATCAGATGGTGCCATCATAGAC

CTGTTCAACATTGGCCCAGACTTTTGGTTGGATTCAAATTTGAATTTGTA

TGTGATGGCAAATCTCAATTTGATAAGGTTATACACATTCTAAAAGAAAA

AACATGTTAAAGACCCTTCAGAAGTCTTGCCTAATTTGATGAGTGGGAGC

TAGATCAAATGGCCTCTAAATTTGTTTATGCTATGAGAGCATTATTTATT

TAATTTTACATGATTCTCATATGTGTCTGTATTGTCTTTGGATATGCCCT

TTTCTTTATATGTATTTAGTGCTTCTTGGTATGTGATAAAATCTTCTTGT

TAGTGCTTCTTCAAGTTTTAAGTACTGTTTATAAGTATTTGGTACAAATT

CTTCAATTTGTATTTATTTTGGTGAATCTGATGCAGTTTATGTTCTGATG

CAGGCATGTTGACCAGTGAAGACTACAACTTTAATACTAAGACATGATTT

TTGGATATATGTTAGTTTGTGGTTAATGTTAAAACTTGATTTTTGGATTT

TGGATATGTTAATTTTGTGGGTAATGGTGATTGGATATTAATTGAATGAG

AAAGTGAATTAATACTTCATTACAGCTATTTGAATGGGAGTTATTATTTT

TATATTAGTGCTATTACAAGTATTTTTTTAGTTCAATCAAATAAAATTAG

CACAATTTCAAGAAAAAAAAAAAGAAAAAAAAGAGGATATGACGGCGTTT

TTCCAAAACGTCGCTAAATATCGTCAGTGACGTCAGCGTGTGTTTAGTAG

GATATTTGGCGGCGTTTTTAAAAACACTGCCTATACTAATATCACGACAT

TTTTAAAACGCCACAAAACTATAGCGACGGCCGCGTCCTCGGCGTTTGGT

GCTAAGCGTCGCAAAAAGAATTGGCGGCATTTTTGGCACTTGTAGCGGCG

TTTTTTAAACGCCACTAAAAATCTATTTTCCAGCAGTGGTACAGGAAGAA

AACAAAGTGGCTTATATTGCCCAGATTATGTTTTTTGATCTGTCCCCAGT

GTCAAGTGTGGAAATTCAAGACTTTGAAGGCTCGTTCGAGTGGAGTCCTT

AGGCTGCTCAAGCGGAGCTTCTTAGCCACTCGAGTAGAACTCAGGGAAGA

CCGCTGGATCATTCTGCTTGGGCAAAGACAGTGGCACCTGAGAGTGAAAA

TTAGTGTTTTGTCTAGATTTGAACTTTCTGCACTATTTTCTTCTTGTTTT

AGGATTTGGTCTTTCAGCACTATGTCATACATATGTACTTCATAGTATCT

CTATTAGATAGAGTTTTGTGATCATATTGAGAGAGATACATTAGTAAGAG

ATTATTAGAGAGAGTTTGTGAATTTGATTATTTGAATATGAGACTTGTTA

TAACCCCTTTTATTCTAATCTTAGTGAAATCGTAATGATTTTGTTGACAT

AGCCCCTGAATCCCGTAAAATCTCTTACAATGCTTGTTCGAGCATTTGGT

TTTCTTCTCCTTTTTTAGACCTGTTGTGTTTGCATGATTGTTTACTCAAC

TTCAGTGCTTAGTATTATTTATATGTATACCATAGCACCTAGCACTATTT

TTATTTATTTTTTCATTATGATAGACTTGCCAAGAAAACTTTAGGCTGTA

TTTGATTCAAAGATTTTGAAAGGGATTTGGAAAGGAAAAATGACTTTTTA

AAATTCTTCTCCAAATTCTTGAACCAAACACAACGTTAGTGTCAATTGCT

ATCAGGTGAGTGAGCAAATATTGAATATAATGGCTATATTATATGGTTGT

ATCATCATTTAATGAGCAATTTTAATTTATGATAATGATTGTGGGAGACA

ATGATTGTCCACTTAATCTATTTGTAACTTTTTATTGACCAATAACACTA

CGTAAGATGATATCATTATAATTTTATTGGCTAGTAATATCACGTAAGAC

GAATGATATCGTCATTACACTCAATATATTTTTGGATGGTATCATCAACT

TTTTATACATAGGATGATATTATGTTACACTCAATATATATGTTTCTAAG

TGAGGAAATGGGAAATCAATAGCAAAAACTGTCCTAAAACAACCACCACT

TCATTAACAAGGGGTTTTACAAACCTCTCTTCCTTTCGATCAATCAAATG

TCTCAAAACCAAAGGAATTCATTCCTCGTCATTTTTTTTCAAAATTTTGA

AAGTCATGATCAATGCCATATACAAGTGGCACAAAAAAGTACTCATAAAC

GGCAGTGGTATTATTCTCATTCAAAGTTTCCAACTCATCCAATTCCTCGG

TTACAACTGTAGACACCCCTTTTTTGACCGTCCTCAAGACTTGGGCGTCA

CCGTTTGAACACTCGGTGATCATCCTTGTTTGTGTTCGTCTTGAGTTCCC

GGAGGTCTCGGCTACGCGCCTTGCTTCAGGACCTCTCGACTCGACTGTTT

TGTGTTTGGTTTTGCACCAAACACTTGTGTTTTTGCTTTGGAGGATAGGG

AATGGCTTATTGGCCATCCCCACACTCCACCGGCCGTGGCTTGAGGCCAC

CGGCTATGTTTTGTTTCCATCGGCCAAATATTTGGCCGGCCGGCACTATA

GATGGTCGACCGGCCATCTATTTTGGCCAAATTGCATTTTTGACTGGCCG

ACCACTGTTCACCTGCTTTTGCAGATATTTTTGTGCCAGCTGTGATAGCT

GGCACCCACCTTTGCTCCCCTCCATACATGTGTGTGGAGGGATTACAACC

TTGTTAGCTTGCCTTGGAAGCTTTACAAGGTTGAATCTTCCATAAGAGGA

GGCCTCTTCTCCCTCTTTTAGGTTAGACAACATTTTTCAGAGGGCTCTAG

TTTTAGGGTTTGAGTTGTGTATCTATCCTATGCCTATATCTCCCTCTCTC

TTGTGTTTTGTGTGTGTTTTATCTTACTTTTGTGCACAAACACCCACCAT

TACACCTTCCTTTAGCTACTGAGGTACTAAGAGGCTGTTCTTGAAGTTTT

GGAGCTGGTTTGAGCCAAAGAAGCTACTGGTCCATCATTCTTGAAGAGGG

TCAACAAGGGTAAGAACTTATCCCTTTTATTCCCCATTTTTGCATAGCTC

TTTAGTCTGCCTTGAAACTGTTTTTAGGCTTGGTGTATAACATATAAAAG

TTTTGTTTTTGCCTACTCTAAATAGTTGGTTAAGAGTATTTTGAGCCTTA

AGGTTGTTTGACCCCTCTTAGGGCTTATTTGACCCCTTTTTTGTGCCATT

TTGGTGAGCTCCGGGACCATTGTGTTTGGGCCTCCTCACCACTGAAATCT

TGAGGGATTTCAGTGGTAAGGATGGTGCCTTGAGGTCCTAAGAAGGAGAG

GGCCACCCCATCTCCTTGTTGTCCTTGAAAAGGGCCATTTTAGCCTCTTG

TGTGAGCATCTAGTTTAGGTCCATGTTGATCATGCCTTAACTGATTTTTG

TGCTTATTATGTCATAATATAGCTCTGTTTTTAGTTGGTTTAGGTACACC

AAGCTTGTTTAGCATACATGCCTTGTAATGCCATGTTTTAGCCTTAATAT

GCTTTAGATTTTTGACCCTTGTTCTAGCATGTTTTTGGCCAAGCTTTGTG

AAGTTTTTGTGCTTGTTTCAGCATGCTAAAGTTCTGTTTTTGGTTGTTTT

GGTCGACCATAACATGCATTGCATAGCCTCTTTGCATTCTTAGCTTGTGA

GCCTTAAGTGCATGAACCATGAGCATGCATTACTTGTATTATTTTAGTGA

TTTGGAACCACTTACCATGTTATTTCAGTCCTTGTGGCATGCCTATATGC

TTGCATTTGATATCCCTTTAATGTGTTAAAAGGTTGTTGTCTTATGCTCC

TTGTTTGGGCCCTTGTGCATGCTTCCATACTTAAGCCATTTAGATGCCAT

TTAGGTGGGTTTTGAGGCTCATTTTAGCATGTCACATCTCTATTTTCAGT

CACTTAATTCATGCCATGTGTGGTTGCACTAAGTGTAGTTCAATGCTTCT

TTGGTGAGCCTTAGGCTTTAGACCATTTGTTGTATGCTATGCTTGTCTTG

TTTGCTTGTTTTGTCACCATCATTAACATGATTTCCAGAGCTTACATTGC

ATGCCATATGCCCATGTTGTTCCTCTTTTAAGTGTGCTTAAAAGTTGTCT

CTATGTGCCCAAGTGTTGGACCTTGCATGCATGCCTATTTGTATGTGTTT

TGGCTTCCATTCTTGAGCATGATGGCCACTATTTTAGGCTTGTTTGAGGG

CTGCCTTAATTAGTTTAAGTCCATCCTTCATGTGTTATAGCATGCTTGCC

TCGTGCATTGTTGCATACTTGGTCTATTACATGTTATTGCTTGCTTGAAA

TCATGGTGCATTTGGCATGTATTTCATTGGCCAAGTTGCTTTGCATTGTT

CATATGCTTAGTCCACTTTACATGTTGTTGCATGTTTGCCACTAACATAG

TTGTCACATGCCTTGCATGCTCATTTTGCATACTTAGCCTCCATGTCTAG

GTTGCATTCCATTTGCATGCATGCTTCACCTCTTGAGTTGAGTATGATAG

AGGTCGAGCCTTTTTGTTTGATTATGTGCATTGGATTCTATATCAAAATG

CATACTTGGTTTCTTGGGCCATCACGCATTGAAATGCGCGATTGGTGGTC

CGAGAATCGAGACCCATCCTCCTTTGAGTCATGAATATGATAAGGAGTGT

GGTTGGTGCATACATTGCTTGAAATGCATTGCCATGCTATGTTTTGCCCT

CTGCTGCTGTCAAAAACAGATTCTGCAGGAAGCTTTGGCCGGCCGGCCAT

CTCCAGTGCCGATCGGCCATCCATAAATGGCCGATAGCTTGAACAAGTGG

CCGGTGATCATTCCCTTTGGCCGGTGACCAATTCCATTAGCTGGTGGTGT

TCCACAACACTCAGAGAAGCAACCCTGGGACTTGTACAGTTTTCAAATGA

GGATAACAGTCAAAGCAGAGGCAACTTGTGCATGGCAGTGGCTTTTCGAG

CGAAGCCGAACACGCATTGCATTGGGCCCTCTTCCCGAGGCTGTTTACTG

TTTTATTAGATTAGTTAATTCACTTAACTAGTTTAATTAGTTTAATTGGT

CTGTATCATTTTAATTAGCTTGTAGGTTAATTAATTATATTATTCTTTCG

GTCCCTCCGAGTGAGGGCGTATTTGTAATAAGTGTGGGTGCGTGTATGTC

TTCCAACCCCCACTTCTTTCTCCTTTTTACTTACCTGCTTTTGATGGTTA

GTATTGGGTCATATTTAAATTTAGCATACCTTATGCTATGCATGCGTGCA

TCCATACATTTGTCTTGCATGCACCGGGCTCGGCGCATCATACTAACTGC

ATTGTATTTCAATTCCAAGTTGAATACATGTTAATTGTAGCATTGTCACA

TGCCATCTTAGAATAGCCGAGCCTATTGTAGTTGCATCTTGTACGTGTTG

CATTAGCCATCTTAACATGAGCTAGGGTCATGCATTGATTACTAACTAGC

TTTTGTTTTGGCTCGGAGTACTAGGTAAGCTTTTATGCTTTAGCATCTTG

CATGAGTGTGAGTATTTGCCATGACAGCATAATTACTATGAGGCTAGCTG

CTTATGTGATATAGCTGCCTGAATGCCTGTGAGTGAAGATTGGGTTATTT

GAATTGGAACAATGCATTGAATGCGAGTTTTAAGCCCACTTGGAAGTGGC

AAAACCATATTTCCTAAGCCCACTTGCGGGTGGCAAAATTCATGTCTTAG

CCTACTTGTGGGTAGCATCATCAAATCATTTTCAAACAAACAAACATGAA

ATGTTTTGTTCCAATTCAGGCGACCCGGTCTATGCTTATTTGCTATGCGC

ATAACATAGTAAATCTAGGCTAATAACCGGAATAAGAACGCATAATGTTT

TCAAAACTAGATATCATGGTGAGTAGAGGCTGATCTTTGATCGGGCGAGA

GGGGGTGCTCATAGCTGACCTTCCCCAATCATACCCAAGTTCTCGAGCCG

AGGCAACTTTGGTGATGACGGACACGTGGCCTTTGGCACCGAGTTAGGGT

TTCCTAAATTCTCCCAAAATAAAATATTTAGGTGGCGACTCTGTATCTGG

CAAAGCAGTCCATGTTTGGCACTTCTTTTTCTAAAAATCTATTTTCCAAA

ACGATTTGCCAGATTTGGACTCTTTGGTTCAGATTAAGAACCGTGACAAT

TCGAGAGTCCGAACTGTAGTGAGCCCAATATGAACAGAACGGGACTCATC

CTCCCGGGGCGTTCGGGGACGTCTACAGTTTCTGGCGACTCTGCTGGGGA

GTTTAGAGAACTTGACTCCAACTCGTGATATCTTGTTTTGACTACACTTA

TGTGTTTCTTTGCATTGCATGTGCATTGGTGCGCCTAGGACACTGTGTTT

GCGTCTGCTATTCCTGAACGGTTTGCCGGAGGCTTGCTTTGAGAAGCATG

CCATAAGGCTGACCGGTTATGGGCTCTGCCAGTATGGGAACACAAACCCC

GATTTTACCGGGACACCCTTTTCGACCATTCGACCTAGTGCATATTCGGA

ATCCATAGTGACCATTCCAAGCTAGAACAGGTTGTTTACCCGATTATAGG

TTGTTTGAAACCGCTGCGTGCGTAGTGAAACCCTAGCTCCTGACAAAATA

GGATAGGGAAACCCTACCCCCAACATAGGGGTGTTTTGGCTCTCACTTGT

TTGCTATGTGCTCTAGAGTGTATCTCTACAAGAATGCGTACATCAGTGTG

CAAGTTTGGGCACCAGGCCTGAGGGCATCAGTACCAGTGTTTTTGAGAAA

TAGTTATATCTGCGGGTATCCGATTCAGTGTTTGGGGCAGTGATTTTGGG

GAACAGTGATTAGTATTAGAGTATAGCTGTTCAGTTACGCCTGTGTTTGT

TCCGTTCGGGCTGAAATGCTGCATGTTTTCTCTTTAAGGGGGCTTAGAGA

CTCGAGTACAGAATAGACAGTGTTCTTCCTCGCTGGGGCAAGGCAGATAT

CTGATCCGTTAGTCACCGTTCAAGATCGGCGAACTTATGACGACCCAAAG

CTTAGAGGAAATTGTGGTTATGGCAGCTGCGTTAGACCAAGGGATCATCT

TGCAGGATGAGACCAATGTTGATGTACCGCATCCGCCCAGGTCGCCTGCT

GCACACCCGATCTGGACTCCCCAGTTGTATCAGGCTCGTGAGCCCACGAT

GTTTGAATTAATGGGAATGATCAGCGACCTCCAAAGATCCGTGGCCGATC

TGGCCTATGGAATGTCGACTTCGCCACCAGCCACATCATATGCCGGGAAT

TTTGTACCACAGGAGCCCCCGCTACTGGGGCAGATTGTGATCGAGCTCAG

CCAACCAGAGGTTGCCTCCAGCTATGGCAATGACATGTCAACAACAACCA

GGTTCCGCTCTGATATAGCAACCCAAACCCTCGATCAGGCTGAGGCTAGA

GGTAAAAACAAAGTGGGAATAGATGCTGATCCCGTTGGACAACTGCTGTG

GGCAGTCGAGGCTCTGAAAGCTGGGGGAATGAAGAAGCAGCAGGTGTTCG

CAGTCATGACTAAAGCTGTAGATGATGCTTTCCCCACTCCAACCGAAGAC

AGTTCAAGCCAGGCGGAAAATCGGGATGGGCCCCGTCATGCCAAGACAGT

TCCTGTCATTCCTAACCCTGCACCAGGAAAAGATCAGAAAGCCAAGGCAG

TGCCTGCCGACCCTTTTGCTAGTGCGTCTAGCAAGAACCCTCAGACTTCG

AAGTACCTGCCTCGAGGGTGCAGAGTCTTTCACGCCCTCTACATGCCTTT

GTCTAGGGCATTTCAAATTTTGGCTAAAAAGGGGCATCTTAAACCTCTCG

AGCCTCGGCCTATCCCAAAGAATCTCCCTCTCTCACATGATGCTACCTTA

TACTACGCGTACCACCAACAGTCCGGTCACAGCACCGATGGTTGTTTCTG

CCTTCGTCATGAAGTGCAAGATTTGATCGACAATGAGGTCATCCTCCCGC

CTACCTCAGCAAAGTCTGTTACCACTGAGCTATTGGACTTCGAGGATACT

ACGAGTTTGTGATCAGGGGTTTGACCACCTGCGGGCGGCACTCGATGCAT

TTCAGATGTGGCGTCTAGCACGCACTTTTGAGCATCTGCGAATGCCAATT

TTTGTGGTTTCCGACTTAAGTGTTGAGTCAGTTCGATGGTCTTGTTTTGA

GTCTGTCTCTTTGCTTTGAGTCAGTCTTTCTGATTAAGGGTCTGTCTTGT

CGCGTCAAGTTAGTCCCATTGTATAAAATGTCGGGTGATTCCAGAGTTGT

TGCCTTGAACACCCTTTTGTTTTGTAATTACCCTTTTACTTTCTTTTAAG

TCTGTCTCTGCCGTGGAGCCAGTTGCAACCAGGGAATCTGAATGAAACAG

TGCGGCTTTCATGTTTCCATGCTTACTGCTATTTTGAGCTAGTTGTGCCA

GAGGATTTGAATGGAACAGCAGTACATTCCACCTACTCTCTTGTGCCCTG

AAAATCATTGCATTGACCTAAGTTTGTCAAAACTTTTTTCAAACACTGTA

GTGATGAAAACGAAGAGGAAGGAATTCCTCGGGATTTGCAAAACTTTGAT

CAATCGAGAGAAAGAGAGGCGTGCTAAGCCCTTAATGATCGAAATCATGC

TTGTTAGTGTGAGTACTGAGAAGGACCCTGGCTTGATCCAGACTGGTTCC

ACACTATCTTCGGCGCGAGCGATTTGTCGCCTTCTTAAAAACTTTCAAAA

ACAAAATGTTTTGCGCGGTCCTGCGAAAACCTGTGTTGAATTGCTCTTGA

GATTGTGCAGCTTAGGATCGCTCTCAGTCTCAAGACTTGAGCCCTTGCTT

TAAAACCTTGAAACACAATTCGGAGTGTTCAACGATCGACCATCAAGGGA

GGCCTGTGTGGCCAGATCGGAAGCAGCCCTCAAACAAGAGGCACCAAAGT

CAGATCAAGAAGATCAGACCTCATCAAAAGAAGCTTGTGAAGCTAGAACA

AAAACACCCCTCCAACTAGGGGCAAAAATGTCCGATGTGCTCCAAGAGCC

GAAGAGGGGTCCTTCAGCAAATCGATACAACAACCACTCTCCTGGACACG

GATGGCTTCGAAACCCTAAAACCATTTGGCATGGACTTGTTACACTTTTA

CCCTTTGAGAGGCTCGCTGGGCCGAAAACCCGGGAGGGTGACCCAGGCAA

AAGTTAGAGCAAGAGAAAAATACAAAGAGAGAAAAAAGAGAAAAATACAA

AAAAGGACAAAAAATGCAAAAGAAAATAAAGGGAATAAAAAGATACAAAA

AAATTGAAAAGAAAAGAAGAAAAATAAAGCTCGCAAGGTCGAAAACCTGC

AACGGGCGCCTTGGCAAAAGTTAGAGCCTAAAAAGTGAAGGTTAACACCC

GAGTGTACCTTAACCGGAACTACGTAAGGCTTGATTCCCATGTTTGGGAT

ACGTAGGCAACTTCATCCTGAAGTCCAGCCCCCAACGCTTTCATAGCGAC

TAAGTCCATGCAATGGTTTTTAATCATCCTTGGTTCCCATGTTTGGAATA

TATAGGCAACTTCATCCTGAAGTCTAGCCCCACGCACTTTCAAAGTGACT

GTGTCCATATATTGGTTTTGACCATCTTCGTCTTCTCCGCGTCCGAGAGT

TTGGTCGTGATCGTCACTAATGAGGACTGACTACAAAATGCTGCGGCATT

ATCAAGACAAGGGGCAACTTTCCGTTGCCATAAGTCGCCGTTCAAACCAG

AGGCAACATTCCATTGCCGTGCAGTGTCCTTCAAACAAGGGGCAATTTTC

CATTGCCACCGATCCGAGGTTGCGCATCTCGAAAGTAAGCTCCGCGCTCG

TTGAGCGGTGGCCGTTTGCGAAAATAGTTTTTGAAAATTTTTACTGAGGT

TGACTATTTTCTCGAAAACTATTTCATTTCTCCAAAACTTAGACCATTTT

AGACCCAAAAAAAGGAAAATTTTCAGTGCTCATGGCCTGACTTGCATAAT

TTTCAAATAAGAAATCGAACAAATATGTTGAACTAACTCTCATCTTGGTC

AAGACTGTTTTAGTAGAAAACCAAAAACAAAAAAATGGTCAAAATGATGA

ACTAGGGGCACGGTTGAGTCAGGCTTCCTGTCTTTCCCCCATCGCCAACA

TACTTGCCAAGAAGGGCACAACTCAAGCTGTCATTAAAGGGCTGAAAGCT

CTCTCAACCTCCGAGCAACGAGGTATCAAACCAAGTGCAAAGCACGAATC

TGAAGGGATCAAGGTACTCCGTTCCCTTACCTGGGGCATCGACCAAGCGC

CACCGGAAGAAACAGCTGTGAGCTAACCATGTCGGGCGTGAACCCACAAC

TTCCTATTCATCCATTAAACACCCCGGGAAGGGATAAAAATGAGAGAATA

AAGAATAGAGAGGTGTAAGTTGAGAGAAAAAGCGAGCCAAAGGGTCTTGA

GAGAAAAATTGAGCCTGAGGGCCGAATTATATGAGAACCACAAATAAGAA

AGGGTGGGAAACAATACCCAGAGAAACTGGCTCCTCGTGATCTGAGGTCC

CCCATCTCCGTTAACGGGTCTGTATGTCGTGTCTTTTGTCTTGTTTACTT

CAACCTTTGTTTTGAGTCTTTTCCGAGTGTGTCTCGTTGTGTTTAGGTTG

TTTACCTTTTTTGTCTCCGCTGTTGAGTCATTCTCGCTATGTCTAGTTGT

AGGCTCACTACCCCGGTTTAAAAAAAAAACCCTTCTTCAATTTCAAAAAT

TAATCATTTTATGCTCAGAATGCTGTGTCATTCTCTACATTTGTGCAAAA

ACATGATTTGGTTCTTTCATACAACTGCAGGGTCCACTCCCGATAAGTGT

GAACAGGAAAACCTGGGCCTCTTTGAAGCTTCTGGGTTTCAGATCGCATC

TGAGTGTGCTTCAACTTCAGTAGATTCAACCTTCATGGTCCGTCTTGTCA

GATTTGGGCTGTTTGTTCTGGATCCGCTGTGTGCAAAATGAACCAAGCGC

TATGTTATGAGCAGTATGCAGATTAATCCCCAGTAGAGTATGAGCTGAGT

GTTCGGAATGAGCAGTGGTTGGTATGGGTTATTTTGCTGTTGGCTGGTTC

GTTATCTGATTATTACAATGCAAGTGTTCAATTTTCAGACAACAGATCAA

TTTCATTTACATTGAGGTGCAACATGATGCAATGCGATGCAATATATGAA

ATGTACAAATGCAACACTTTTTCAGATAAAAAGTTTGTTTCATTTCATCA

TCTTTTTCAAGTACATGGTTTTGGAAAAAAAAATCTGCCTACGGTAGTAC

AAGGTACTCTAGCTACCAGCAAAAGGTACAAAAGGAAAAGAAAAAGAAAA

ACAGCAAAAGTCTGGATCAATGTCAAAAGGCCGCGGGTCTAGGGGTCCAT

CCTCGTCCTCTTCCTGGACGGAGGTCCAGGAGCATTGTCATCGTCATCTG

AGTCTAAGCCCGAGCCGTCTCCGGTCTCCTCCTCGGACGACTCTGCCTCC

GCCTCAGCCTCAGCCTCGGACTCATCATCATCATCAGGGACGGGTGCCGT

GAGCTACTCCCGACATGGGTGCGCCCTGCCTTGGTGCGGGGGTATACTCT

GCCATCTTCTCCGGTAAGAACAGAAGGTCCAGCTGGAGCACCTCCCACAG

AAGGGATCTGGAAGGGAGGCTGAAAAACACAAATGACAAATGCATCAGTA

TGCTACAGAATGCAGTTGTCTGAATACAGTGTGTGAATACTGTGAGCAGT

GTATCAGTGCAGAATGCAGTTGTCTGAATACAGTGTGTGAATACTGTGAG

CAGTGTATCAGTGCAGAATGCAGTGTATGAATACAGTGTGTGGATACTGT

GAGTAGTATATCAGTGCAGAATGCAGTGTATGAATACAGTGTGTGGATAC

TGTGAGCAGTGTATCAGTGCAGAATGCAGTTGTCTGAATACAGTGTGTGA

ATACTGTGAGCAGTGTATCAGTGCAGAATGTAGTGTATGAATACAGTGTG

TGGATACTGTGAGCAGTGTATCAGTGCAGAATGCAGTTGTCTGAATATAG

TGTGTGAATATTGTGAGTAGTGTATCAGTGCAGAATACAGTGTATGAATA

CAGTGTGTGAATACCGTGAGCAGTGTATCAATGCAGAATGCAGTGTATGA

GTACAGTGTGTGAATGCCGCGAGTAGTGCTGTGAACAGTTTGTGGGCTAC

AGTATGTCAGATGCCGTGAGGGCAGTGTATCAAGTATTGTGTTGTGCATC

AGATATGAGAAACAGTTTATGTATGGTTTGAGACCGCGGTCAGAGAAACA

TACCAGAATGCTGTAAATCGCCAGAAGGTCGAGGCTCTGCTGGAGCATCC

CCATCACGGAGGCGGTCAGCTCTAGGGACTGGTGAGTATACTCGGAAGAG

GCCTGCAAAAGCGATAGAAGCATCAGTATTCTGCGGAATATCAAATGAAT

TGAAATGAACTAACTATGCATAATGCAAAACGCAGTAGTACATACCGGAG

GCAAGCTGTAGAACCCGACTAACTCATGATCAGCTGGAGGGGGAGCAATG

GGAATCTGGTAAGTCGCCCCGCCCTGTCCTCTACATGTCATCATCGTGGG

TAGTGCTGGCCAGCTAACCCCCCGACCCCTCCGAGCTCCGCTCCTGCCAC

GGACTCGAGCTGTCCTCGACGTCCCTGCCTCTGCTCCCGCTCCAACTACC

GGCGCCGCAAGTCCCCCCTCTTAACTCGATGGCCACTGCTCAGAGGCCGC

ATAATGTACGTCCGAACGTACTCTGAGTAGTCGCCCTCGCCCCTAAAGCC

CTCTACGTCCCAACCGATCGTGTAATCGGCCATCTCCTCGGCAATGCACC

TCTCGGTGTCCCTCATGTGGGTTAGGAGGCGGACCCGAAACTATCTGCTC

TGGCAAACCCAGGGTCTGGCGTAGGAAACGCTCACCCAGGTACCAAGCCC

GACAGACCGGGCCCTCCAGCAAAATCCTGAAACGTGCAGTCTCCTCTACA

CCAGCAAATCGATCTCGAATCATCGCAGGCAATGGAGCCCAAGGCTGCCA

AGTGATCTGTAAATGACATGCAGTTTGTTATCTACTGATATTGAAAATAC

AGTATAAAGCCAAGCCAAATAAAACTCTAAAAGTTGAACATACCTCTGCA

GGGGTGATGGTGTCAAAGTAGCGGCGGAAAAAGATGAATGTCTCCCGGGG

CCTCTGCACGCACCTCACGTCAAAACGACGTGAGAACAGAATGACGAGAG

GGGTCTCCACGTCTGGCACTGGGGTGAGTCTCGGAAAGTAGGCGTAGACC

CATAACTGCAGAGCAAATACCAGCAGGATTATCAGATAAAGCAGAATACA

GAATAAAACAGAAATTACAGAACCGTGTATTTTGCAGTGTATTGTGCAGA

GTGTACAGTGTGTGCAGTGTACAGTCAGTGTAAAACAGAGTATGTGTGTG

CCGTGTGTGCAGTATATAGTCAAACGTGAAGCAGACCGCAGCGTGTGCAG

TGTACAGAGTACAGAAAATATAGAGCAGAAACGAAGAACAGAAGAAGAGG

TGTACCTCCCAAGCTCGCCAGTAACCCCCCAATAATTGCCCGCTGCAACG

GGAGGCTGAGCTCATGTATCCATATAGAGTGGCTAGAGCAGCACCACCCC

AGTCGAAGTGCAGAATCCGTGTCGCATCCACCAGTGCGCTCAACAAACAG

AGGGGCACGGTGTTCTCCTGGTCGGCGAAGATAGTAGTCCCCAGCAGAAA

CATGAGGAAGCCACGCGCATACCGCTCCACCCCCTCCTGAGTCATCGGAG

CCTCCTCGGTCAAGGCTGGCTGAACCCTGAACTGGACCTCGAACCACGAA

TATCTGATGAAACCGGGACGCGCGAGAGGCGGCACCTCGCCGAGTAGGTA

AATCTGAGTGGCCTCCCACTCGTCCATGTCGGTGTCGAAGGGGATCGGAT

CACCCCCTACCTCGATATTGGTGAGCATCGAGAAATCATAGGGAGTCATC

GTCATGTCTCTTGTGGCAGAGCAATGGAATGAGTCGGTGGTGTCCCACCA

CCTCTTCACCAGAGCGGCGAGAAGAGGCCTGCTCGCGGTGAGTCGGGAAA

GTCCTCTACAGAAGGCACTGAAGTCGGCCTGATCGACAATGTGACGGATT

GCCTCTGGTAGCTCCAGATACCAGTCTCGAGCGGCGGCATTCCCATACCC

TCGGCAGGTATAGGAATCTCCCTGTATTAAAAAACAACAGTTGCGAAGCA

ATATTCAATGTACAGTTTTGAAGAACATTCAGGATTGGTGACAATACAAC

GTGTGTGCATGTTTTCAGATTCAAACAGATATCAGGTGCCGGTGTACTCG

AAGTGAATTACGGCATATGACATGTCAGTGTTTCCAAGTGGTTCAGTACA

GATACAGCGTGAGTACACGTTTTCAAGTTCAAACCAACATCGGGTGCTGG

TGTGCTTACAGTAAACTACAACATGTGATGTGCCAGTGTGTTTGGGTGGT

TCAGTGACAATGCAACGTGTATGCACGTGTTCAGATTCAAACAGACATCA

AGTGCAAGTGTGTTCATAGTCAACTACAACATACAGTGTATCGGTGTATC

CGGGTTAAGCAGTTAAAGAACAAAATCAACTATGGTAGTTAAGTGTTCTA

CATTACGGAAAATGAAACGGGGAAGAATATACCTCGAAAGCGCCGTATGA

TAGGTGTGACTCCGGCTCACGCAGGAGAAGCTCTGGTGGCACACCTTTCG

CAAAACTCCGGAACTGGCGAAGTCCGTCGGGAGGAAACACGTGAACCGCC

GACCTGTAGGTCACCGGGTCGAACGGGCGGACCCTCAAGGGGAGGAGCTC

GGCCTCTGGGTCCACCTCGTGATCCGGGGGAGTCCTGCCCCATGGAATAT

CATCCGCCATGTCAAAAGTGGTGTGAAGATGGCCGGAAGAGAGAAAATCG

GGCTAGAATGCGAAGGGTCAACGGAAGGTCAACCCCGAGAGAGCGGAAAA

TGGTCGGAAAAGCCGAAGTGTAGAAAAGGCACAGAAATCAAGAAAAACCT

GAGGAAAACAAGTCCCGAGTGGTGAAAATCGAAAAGGAAATACCGAAACG

AAGTTTCGTTCGGATGAAAATGTTCGAAACGCGAAGAACTTCGCGAATCA

AACAACGAAATGGGCCATAAAACCGAGGAAAGTGACGAATGGGAGAGAGG

AAAATACTCTAGAGAGAGGGAGGAAGGAATTTCGGCCAAGAGAAAGTGAA

AAGAATAGGGCTAGAAGCCCTATTTTATAAAGCCCATGCCGAAATGACCA

AACTGTCCCGACTCAGCAAGATTTGGCCGGTCGGCTAAACTTTTGGCCGG

TCGACTAAATATTTGGCCGGTGTAATGTTTCTATGGCTGGCAGATGGCCG

ATGACATCTCCTGATGGCCGGTGAGCCTCCTACACCTTTGTTTGGGTCAG

ATATGTGGTTTGGGACCTTCATTGCTCAAGACAGTGCAAGAGCAGTGCAT

TTCCGAAGCTCAACCGGCCCCTGTTTTGACCTCGGACCGTGTCTTCTGTG

TGCGGCACTCGAATATGTGCAAGATGTATCCTTCTTGTATCCTTTTGTCT

CTTTCAAGACTCGAAAGTTACCCCACATGCGTTGCTTCGCAATGCTTCGT

CGTGAACATGCGTTGGTTGTACGTACTTTCATTCACATTGATATGCATCT

TTCATTTGCATTAGTTCACTTGCATTGTTAGGCATTACCCCAACAAACGG

TCTGGCGAGACCTTAAACCCGCTTGGTCTGGCGAGACCTTAAATCCGCTC

GGTTGCATTGTCGTGCATTTCAAGCGACTTGCGTTGTCGCACATTCCATG

CAACATGCATTTCAAACAAAAACAACAAACAAAAACAAAAACAAAAACAA

CAACATGCATCATTTGTATTTCAAAAAAAAAAAAAAAAAAAACAAACATG

CATATGTCATGCATCATTTTAAGTATTGCATATGTCATGCATTTCATTGT

AATATATTGTATATGTCATGCATTTCACTGTGAATATATCCATTCCACTG

CATATTGCAGATATTGCATTGTAGCTTGCATGCATTCACTGCATCACTTC

CATCTAGGGTTTGCGTCTTTCCTTGTTGAGCTTGGTCTGGTATGTGTCCA

TACTTTTCACGTCTATACACTCATAGTTCTGGATGACCGTTCTATATTAG

ACGTGGTTTGTTTGTGTCCATGCCTTTCCTAGGAAAGATACGGTTTGCTG

TAGGCCTTAATCCACTGTGTCCATTCCATTGCATATTGTAGATATTGCAT

TGTAGCTTGTATGCATTCACTGCATCACTTACATCTAGGGTTTGCACCTT

TCCTTGTTGAGCTTAGTCTGGTATGTGCCCATACTTTTCACATCTATACA

CTCATAGTTCTGTATGACCGTTCTATGTTAGACATGATTTGTGTGTGTCC

ATGCCTTTCCTAGGCAAGATTTGGTTTGGCTGCATGCCATATCCATTGTA

CATATTACATTGTAGTGTAATTTTTGCATCCAGCATTAGGATCATTGGTC

TTTCTTCAGCGAGTCGTTTCCCTACTTATGTCATTCCACCCAATTACTTG

TTCCTGTGTCCGTCTACATTACTCATAGTTCTGCATGTGTACGCGCCTAT

TCCACTGCTGATATTGTGGTTTGAGGGTTGCGGCACCTTTTCTATGGTAG

ACAGACTTTGCATCTCTATCCATCTTTTTTTTGACATTTGTGTGGTTTTG

AACTGCTCATGATTAGATCATGATTCAATTGCATTGCATATCGTTTGCAT

TGTTTATATATAGTTTTGTATGCTTTGCATATTATTGGCATTGTTTTGTA

ATTTTACATATCATTTGCGTTACATTAGACTCAATTGCATATCATCGCAC

TGTTTGTGCACCCCTTGGTCTAAGGAGACTTTAAATCCTACATTCGCGTT

ATCGCGCAGCATTCATTTTACATATTATGCATGTTGCCAACGATATTGTA

CCTATTGAGTTTTCTTTCTCTTTTAGGCAAGATATTGTATGTACGGTTCA

TTTGAAGCTGGTGCAACCAGACGAAGCGCGCAGGTATTGTATTCTCCGAG

TGAGACGTATCGAGAATTGCATAATTCTCTTACATCCTTTCTGCATTGTA

GTGTAGTTGGATTGTTGAATGCTAGCTTAAAATTGTTGCATGTTATGCTT

CGTTACAGCTTTCTTTGCATGATCATAAGTTATGTTCGGCTTCTTGCATT

ATCAGTTGCATTTTGCCCCTGCCCTCGGGCGCATATCTTTATTATCTTGC

ATGCTTTGTCTAAGTTTTCGGCCTTGTCAAAATGGGTCGTCTCTGGGCTT

TGACACTCACTCCCGTCATTCTTATGACTTTCGAGTTCTGTCAAAGAGGG

GGTAACTGTAGACACCCTATTTTTGACCGTCCTCAAGACTTGGGCGTTAC

CGTTTGAACACTAGGTGATCATCCTTGTTTGTGTTCGTCTTGAGTTCCCA

AAGGTCTCTGCTACGCGCCTTGCCTCAGGACCTCTCGACTCGACTGTTTT

GTGTTTGGTTTTGCACCAAACACTTGTGTTTTTGCTTTGGAGGACAGGGA

ATGGCTTATTGGCCATCCCCACACTCCACCGGCCGTGGCTTGAGGCCACC

GACTGTGTTTTGTTTCCACCGGCCAAATATTTGGCCGGCCGGCACTATAG

ATGGTCGACCGGCCATCTATTTTGGCCAAATTGCATTTTTGGCTGGCCGA

CCACTGTTCACCTGCTTTTGCAGATATTTTTGTGTCAGCTGTGATAGCTG

GCACCCACCTTTGCTCCCCTCCATACACGTGTATGGAGGGATTTTAACCT

TGTTAGCTTGCCTTGGAAGCTTTACAAGGTTGAATCTTCTATAAGAGGAG

GCCTCTTCTCCCTCTTTTAGGTTAGACAACATTTTTCAGAGGGCTCTAGT

TTTAGGGTTTGAGTTGTGTATCTATCCTATACCTATATCTCCCTCTCTCT

TGTGTTTTGTGTGTGTTTTGTCTTACTTTTGTGCACAAACACCCACCATT

ACACCTTCCTTTAGCTACTGAGGTACTAAGAGGCTGTTCTTGAAGTTTTG

GAGCTGGTTTGAGCCAAAGAAGCTGCTGGTCCATCATTCTTGAAGAGGGT

CAACAAGGGTAAGAACTTATCCCTTTTATTCCCCATTTTTGCATAGCTCT

TTAGTCTGCCTTGCAACTATTTTTAGACTTGGTGTATAACATATAAAAGT

TTTGTTTTTGCTTACTCTAAATAGTTGGTTAAGGGTGTTTTGAGCCTTGA

GTTTGTTTGACCCCTCTTAGGGCTTATTTGACCCCTTTTTTGTGCCATTT

TGGTGAGCTCCGGGACCATTGTGTTTGGGCCTCCTCACCACTAAAATCTT

GAGGGATTTCAGTGGTAAGAATGGTGCCTTGAGGTCCTAAGAAGGAGAGG

GCCACCCCCTCTCCTTGTTGTCCTTGAAAAGGGCCATTTTAGCCTCTTGT

GTGAGCATCTAGTTTAGGTCCATGTTGATCATGCCTTAACTGATTTTTGT

GCTTATTATGTCATAATATAGCTCTGTTTTTAGTTGGTTTAGGTACACCA

TGCTTGTTTAGCATACATGTCTTGTAATGCCATATTTTAGCCATAATATG

CTTTAGATTTTTGACCCTTGTTCTAGCATGTTTTTGGCCAAACTTTGTGA

AGTTTTAGTGCTTGTTTTAGCATGCTAAAGTTCTATTTTTGGTTGTTTTG

GTCGACTATAACATGCATTGCATAGCCTCTTTGCATTCTTAGCTTGTGAG

CCTTAAGTGCATGAACCATGAGCATGCATTACTTGCATTATTTCAGTGGT

TTGGAACCACTTACCATGTTATTTCAGTCCTTGTGGCATGCCTATATACT

TGCATTTGATATCCCTTTAATGTGTTAAAAGGTTGTTGTCTTATGCTCCT

TGTTTGGGCCCTTGTGCATGCTTCCATACTTAAGCCATTTAGATGCCATT

TAGGTGGGTTTTGAGGCTCATTTTAGCATGTCACATCTCTGTTTTCGGTC

ACTTAATTCATGCCATGTGTGGTTGCACTAAGTGTAGTTCAATGCTTCTT

TGGTGAGCCTTAGGCTTTAGACCATTTGTTGTATGCTATGCTTGTCTTGT

TTGCTTGTTTTGGCACCATCATTAACATGATTTTCAGAGTTTACATTGCA

TGCCATATGCCCATGTTGTTCCTCTTTTAAGTGTGCTTAAAATTTGTCTC

TATGTGCCTAAGTGTTGGACCTTGCATGCATGCCTATTTGTATGTGTTTT

GGCTTCCATTCTTGAGCATGATGGCCACTGTTTTAGGCTTGTTTGAGGGC

TGCCTAAATTAGTTTAAGTCCATCCTTCATGTGTTATAGCATGCTTGCCT

CGTGCATTGTTGCATACTTGGTCTATTACATGTTATTGCTTGCTTGAAAT

CATGGTGCATTTGGCATGTATTTCATTGGTCAAGTTGCTTTGCATTGTTC

CTATGCTTAGTCCACTTTACATGTTGTTGCATGTTTGCCACTAACATAGT

TGTCACATGCCTTGCATGCTCATTTTGCATACTTAGCCTCCATGTCTAGG

TTGCATTCCATTTGCATGCATGCTTCACCTCTTGAGTTGAGTATGATAGA

GGTCGAGCCCCTTTGTTTGATTATGTGCATTGCATTCCATATCAAAATGT

ATACTTGGTTTCTTGGGCCATCACGTATTTAAATGCGTGATTGGTGGTCC

GAGAATCGAGACCCATCCTCATTTGAGTCATGAATATGATAAGGAGTGTG

GTTGGTGCATACATTGCTTGAAATGCATTGCCATGCTATGTTTTGCCCTC

TGCTGCTATCAAAAACAGATTCTGCAGGAAGCTTTGGCCGGCCGGCCATC

TCCAGTGCCGACCGGCCATCCATAAATGGCCGATAGCTTGAACAAGTGGT

CGGTGATCATTCCCTTTGGCCGGTGACCAATTCCATTAGCCGGTGGTGTT

CCACAGCACTCAGAGAAGCAACCCTGGGACTTGTACAGTTTTCAGATGAG

GATAACAGTCAAAGTAGAGGCAACTTGTGCATGGCAGTGGCCTTCCGAGG

GGAGCCGAACAGGCATTGCATTGGGCCCTCTTCCCGAGGCTGTTTACTGT

TTTATTAGATTAGTTAATTCGCTTAACTAGTTTAATTAGTTTAATTGGTC

TGTAACATTTTAATTAGCTTGTAGGTTAATTAATTGTATTATTCTTTCGG

TCCCTCCGAGTGAGGGCGTATTTGTAATAAGTGTGGGTGTGTGTGTGTAT

GTCTTCCAGCCCCCACTTCTTTCTCCTTTTTACTTACCTGCTTTTGATGG

TTAGTATTAGGTCATATTTAAATTTAGCATACTTTATGCTATGCATGCGT

GCATCCATATATTTGTCTTGCATGCACCGGGCTCGGCGCATCATACTAAC

TGCATTGTATTTCAATTCCAAGTTGAATACATGTTAATTGTAGCATCGTC

ACATGCCATCTTAGAATAGCCGAGCCTATTGTAGTTGCATCTTGTACGTG

TTGCATTAGCCATCTTAACATGAGCTAGGATCATGCATTGATTACTAACT

AGCTTTTGTTTTGGCTCGGAGTACTAGGTAAGCTTTTATGCTTTAGCATC

TTGCATGAGTGTGAGTGTTTGCCATGACAGCATAATTACTATGAGGCTAG

CTGCTTATGTGATATAACTGCCTGAATGCCTGTGGGTGAAGATTGGGTTA

TTTGAATTGGAACAATGCATTGAATGCGAGTTTTAAGCCCACTTGGAAGT

GGCAAAACCATATTTTCTAAGCCCACTTGAGGGTGCAAAATTCATGTCTT

AGCCTACTTGCAGGTAGCATCATCAAACCATTTTCAAACAAACAAACATG

AAATGTTTTGTTCCAATTCAGGCGACCCGGTCTATACTTGTTTGCTGTGC

GCATAACATAGTAAATCTAGGCTAATAACCGGAATAAGAACGCATAATGT

TTTCAAAACTAGATATCACAGTTAGTAGAGGCCGATCTTTGATCGGGCGA

GAGGGGGTGCTCATAGCTAACCTTCCCCAATCGTACCCAAGTTCTCGAGC

CGAGGCAACTTTGGTGATGACGGACACGTGGCCTTTGGCATGGAGTCAGG

GTTTCCTAAATTCCCCCAAAATAAAATATTTAGGTGGCGACTCTATATCT

GGCAAAACGGTCCATATTTGGCACTTCTTTTCTAAAAGTTTATTTTCCAA

AACGATTTGCCGGATTTGGACTCTTTGGTTCAGATTAAGAACCGTGACAA

TTCGAGAGTCCGAACTGTAGTGAGCCCAATATGAACAGAACGGGACTCAT

CCTCCCGGGGCGTTCGGGGACGTCTACATACCCTCAACTAATTCTTCCCC

ACTCTCTTTCTTTCTAAATCCACAGACAACTTCTGCATATATAATCAGGG

ATTGTCAGATTTTTGATCAATTTGCAATCAATTTTTTATTGGTAAAGGTT

AAGAGATTCGGATTAGAGAGATGATCAGAACTGGTGCGGTTTCGCAGCGG

AAGAGATCGTCGGAGATGATCAGAGAAAGCTTCGACGACGTCGTATCGCC

TTTCGATTCTCCGGTGCTGTTTCACAACGGAAGAGATCGTTGGAGATGAT

CAGAGAAAGCTTCGACGACGACGACGTATCGCCTTCTAATTCTCATGAAG

TTCATGTTCTTGCCGTTGATGATAGCCTTGTCGATCGAAAAGTCATTGAA

TGA

>TEA015580.1 locus=Scaffold1785:17047:23131:+ glutamine synthetase cytosolic isozyme 1-like

AGCACATTACTCCTCGCCACAGACTCCAACACAAACCACGTATAGTACGG

AGAGAAAGAGGAGAGAGGGAGAGTAAAAGCATCGTGTGGTTTGTTTGGAG

TGGGTGTTTTTGATCGATCGTCACTATGTCTCTTCTTTCCGATCTTTGCA

ACTTGAATCTATCTGAGTCTACCGAGAAGATCATTGCAGAGTACATATGG

TTAGTTTTACTTCTCTCAGATCTCTCTTTCTCTCTATGTTTTTGTTTTTC

ATGTTTAAGGCACTCTTGATTGCAGATCAGTGTTTGTTTTGCAATTTTCT

CTTTATTTTGTTCTTCATGGTTGTGACTTCTATGTCTCATGCATAGGTCA

TAGAGTCATATACATTCCATTTTGTTTGAAACATTTTCTGTGTTTTTTCC

TCTTTTTTTTTTATAGTTTTTTATTATAACAATTTGGATTTTTTTTTCCC

CTCTGTCTCTTCATATTTTATTATTATTAATATGTATTTCGTATTTTTGA

TGTGTATATATGTGTTTGATCATTGCACAGGATCGGTGGATCTGGTATGG

ACCTCAGAAGCAAAGCCAGGGTAATACTTTTAATGAATTTAATTAATGCA

CACACATAGGATCTGTTTGGTTGTCTTTATTTATTTATTATTTATTATTT

ACTTTTAATTTTAATTTTATCATTTATAGAAAAGTTTGGAGAACTGTTTT

TAAGAAATATTCTGAAAACAACTCAATTTTTATGAAATACTGAATGGTTG

GTGTTTGGCAAGTGGTTTTGGGGCTAAGATAGCCACAAAAACAAAATAGG

CTATTTTAGCCACAACAAGCAAAACATCACATCTAACCCATAATCATTAC

ATTCTTCTCTCTCAAAAAGTCAAAAAGTCATTTCCAAACGCTATTTTAGC

CACAACAAACAAAATATTACATCTAACCCATAATCATTACACTCTTCTCT

CACAAAAAAAAAGTCAAAAAGCCAACTCAATTCCTAAACGGAGGCAAATG

ACTTTTTGAAAATTATTTTTGCCCAAAAAATGAAAACTATATGAAAACAA

TGATGTTTTTGAATATTGGACTTTTGTTTTTCCTGAAGCAAAAATCAAAA

CACAATCAAACAAGCCCATATCTTCTCTATTGGTGGTCTTAGTTTTTAAT

GCACACATGGGTTTGTTTGATTGTGTTTTCATTTTTCATTTATATCTTAT

CATTTTTAGAAAATACAGATACAGAATTTATTTGGCCAACTGTTTGTTTT

TATCAAATACCAAAAAACTTGTTAACGAATTTGGAAATTGTTTTTGCACA

GAAATAGAAACCAGATGAAAACCAAGGGTGTTTTGAAACTGTCATATTTG

TTTTCTAATTTTGAATTTGAATTTTTCATGTAATTTGGTCGCTGATGATC

TTCTTTATATTTGGTTTCTTACCCTTGAATCAGACCCTGAATGCACCAGT

CTCTGATCCTTCAAAGTTACCACAATGGAACTACGATGGTTCCAGCACTG

GCCAAGCCCCTGGCGAGGACAGTGAAGTGATTTTATAGTATGTTATCTTG

ATTTCAATTCTCAGTTTTCATTTCAGTGTGTTTCTCAAAATTCTTTGGAG

ATTGGATATAACATTGGTTTGTTTATGCAAACAGTCCCCAGGCAATTTAT

AAGGACCCATTCAGGAGAGGCAACAACATTCTTGTAAGTCCCTTCTTTTC

CCCCTATAATGTAAACTGATTTATAAAAAGAATTAATTTATGGAGTTTGT

GGTTGTGTTTCTAATATGAGCAGTGACAAACAGAAAAATATGCAAAATGA

CGTGGCATTTAAACATCATTCTTGTGACCAATTTTGTATAAAAAATTGGT

ATGAATAGTATTTTTCTTCTAATATTGCAGGTAATGTGTGATGCTTACAC

GCCGGGTGGAGAGCCAATCCCAACAAATAAGAGGTTTGATGCTGCCAAGA

TATTCAGCCACCCTGATGTTGTTGCTGAGGAACCTTGGTATGTTCTACTT

TTATTTATTAATTTGTTCATAAAATAAAAAATAGTTCTTTTCATATGGTA

CTGTGCTTTTTAATGTTGATTATTGGGAATCTGCATTATATTTTCTGCTC

CAATTGTGTACTAACTTTTATTTACTTATTTATTTTTATTTTTTCAGGTA

TGGTATAGAGCAGGAGTACACTTTGTTGCAGAAAGAAGTGAAGTGGCCGA

TTGGTTGGCCTGTGGGAGGTTATCCTGGACCACAGGTAGTGAAGTTTCGC

CATCACCAACTATGTCATTGTATTACTTCTTTTTCTTTGAAAAATGATTG

TGTCACATTTTTTAATATGTCTTGATCACTATTTTTTTATTTGCCCAATT

TTCAGTACTAGAAATTCTGGGTGTATTTTATATATGATATATGAAAAAGA

AATACTATGTTTTAGTTTGTGTTCTAAACTGAGTTTTAAATGCAATTGTG

ATTTTGCTTCTTCTTTCTCTTTTGCCAAATTACCACTTGAGCTTGCTGTT

AGAAACTGTATGTCTCGCTCCATGAAAGTAGTAGTAGAGGACCGTTTGCA

TACAATATGCACAGTAGTGGCCCAAGTTTTTATTTATTTCTTTATCATGA

TGTGCTAAGCATCAAAATGAGGTGTTATTTTTCTGGCCATTTCAAAAATT

GAGGCATCCAGTAATAATAATTTATTTTAGTGAGTCTTATACATTGGGCT

AATTGTTGATGAACCACCTCTGGATATGTTTTCTTATTGGGATCTGTTTT

AGGAAATGTAGTCTTGGTTAAATGTGATGAACCTGTTTTCGATGAGGCCT

GTGATACTCTCTTGATGCTTGAATTTAATGGCTTTGTAAATAGATTTGAA

TTTAAAAAAAAAAAAGAACTTGTTTGGCCTGAAGATTCACAAGAGGATTG

TTCTCTAGGTTATTTAGCTTCAGCCAACCTGATTTGTGTTGCTCACCACC

ACTGTTCCTCTTCCTTTTTCATATTGAGGTTTCTATGTGTTTATAATAAT

TGTGAGTTTTATTTTGGCTTTATCAAAACAGGGACCATACTACTGTGGTA

TTGGTGCGGATAAAGCTTTTGGGCGAGACATTGTCGATGCCCATTATAAA

GCATGTCTTTATGCTGGTATTAACATTAGTGGCATCAATGGAGAGGTGAT

GCCGGGTCAGGTAAAAGATTAATTTACACATTCTATCCATTTTATTTATT

TATTTTTTTTGGGGAAAAAAAAAGTCTAGGAACATAGAAACCGATCACAT

AATTGAAGAAGGGGTTGCGTTTGGTTCTGTGATTGATTTCTGTACAAGTA

AAAGAACTTTTTTTTTTTAATCTTTCCCTTATAGAACTTAGGCCATGTTT

GTTTGGGCAGACTGGATTAGCATTCCCATGACTAATAGTCCTAGGACTAC

GAATCCGTTGGATTATGTATCCTATCTTAACTAATAGTCAAGTTTAAAAT

AATTAGTCCATACTTTAATTGTTTAACCAAACATAGGATTAATAATTTTA

TTGGACTATTAGTCCAATCCCAAACTCATGTCCACCCAAACAAACATGGC

CTTAATGCTTTGTCTCGTCAGTTGCCCTGTTTATTGGTTTTGTTGACCTC

AAAGTTTTAAATGTTTTTCTTGTGATATGTAGTGGGAATTCCAAGTTGGG

CCTTCTGTTGGCATCAGTTCTGGAGATCAGTTGTGGATGGCTCGTTACAT

TCTAGAAGTAATTTGATAAATACTTATACTGCAGTAAAGTGTCGGATATC

TGCATATATGTTTCCTTACTGACACATTTCCTGAACTGACAGAGGATCAC

TGAAATTGCTGGAGTCGTTGTTTCGTTCGACCCTAAACCTATTGAGGTTA

GTTTTTGTTTGTGAATGTCCCCTCTTCCTTCCTGCTAAGTCCTCTACTAT

TTGCTACTAATGCTTCGTTTGTTTGAGTGGAACACATTTTCGCGCACAAT

TTGTTATTTTCTGTAGAAAATGTTTTCCTATATTTAGTTGCAACTTCAAA

ATTAATATTTTAGTGGTTGTCTTAGCGGTGGTGGTGGTGGGATTGGTAAG

ACCCGTCAAAAGTATAAACCATTTTCCATTCAAATAGGCTTATTTTCCGT

TTGCCTTTCATTTTCCACTGCACCAAATGTCAGAAAATGCAAAAAATGTC

TGTTCGGCTGAACAAACGGAGCATAATGTTCATTACATTATATTATATAG

TGTAACCAAGTCTACATGCCTTTTCCAGAAAAGAACTAGGCAACCTGTCT

CTAAACTTTGTGAAATTTTGTCAATAGGGTGACTGGAATGGAGCCGGTGC

TCACACCAACTACAGGTAATATTTTGCTCTGTATGCCAAAAAAAAAAAAA

GAAAAGAAAAAACCTCGCAAGAATGATTTGTAGACCAGGGTCACACCCTT

ATACGTGTAATATGTCTAATATAGCCTATTCATGTTGGTAACAGTACCAA

GTCCATGCGAAGTGATGGGGGCTTTGAAGTCATTAAGAAGGCCATCGAAA

AGCTTGGATTGAAGCACAAGGAACACATTGCTGCTTATGGAGAAGGCAAT

GAGCGTCGGCTTACAGGAAAGCACGAAACAGCTGACATAAACACATTCTT

ATGGGTACGCTTATCTTCAGAGTAACGCTAGAGACCCATATTTGAGACCC

CAAATGCACCTCCAATGATATGGCAATGAAGTAGAGTCCCACGTAATTGA

GAATGTGATGACAAATCAAAAGTTGATATTTATCTATTTATCCAAAAAAA

AAAATAAAAAATACACCTATCATGCCACATCATTATAGGTGAAATTGTGG

GGGATTTATTTGGGGTCTCAAACATTTTCCCTTATTTTATTTTCCACGAG

TTCTTATGCTTTCTATAACATGTTTTTGCTTATTGTTATGGTGATGAATT

CTGAAATCTCTCTCTCTCTCTCTCTCTCTCTCTCTCTCTCTCACTAATAG

CATTTGTTGCACATATTTTGGAAGCATTTTAAGTTGATGGGTCCAAGAAA

GAACCCAATTGAGGAATGGTACACGGATATATTGGGAGTTATTAGCTTTT

TCTTCTTTTTGGCTCGACTTATGGGCTTCCTTCATGATTTTGTTTTGTTG

CTGAGCTTCGTTATGTTTGTGTTGTTTGAATTATTTATTTAAGGGCAAAT

TACATATACACCCCTCCAGGTTTGGTTAAATTACAGATACCCCCCTGAGG

TTTCGGAAATTACATCCCGCCCCCTGAGGTTTGTATTTTCATAACAATTT

ACCCCCTGAGGTTTATATTTCATAACAAATTACCCCCTGCTTATTGTTAT

GGTGATGAATTCTGAATTCTCTCTCTCTCTCTCTCTCTCTCTCTCTCTCT

CTCACTAATAGTATTTGTTGCACATATTTTGGAAGCATTTTAAGTTGATG

GGTCCAAGAAAGAACCCAATTGAGGAATGGTACACGGATATATTGGGAGT

TATTAGATTTTTCTTCTTTTTGGCTCGACTTATGGGCTTCCTTCATGATT

TTGTTTTGTTGCTGAGCTTCGTTATGTTTGTGTTGTTTGAATTATATATT

TATTTATTTATTTTAAATTTCAGGGTTGTCTTAGACTAGATCAACTTCAA

AAGTAAAAAAAAAATCACTGAAGACTAAAAAATCCCCCCGAATATTTTAT

TGGGGACATTTTCTTGTCAACACTTTTTTTTGCCTAATGATTTTTGTCAC

AATTCTCAATAGTGCTTGTTCATGTAATTTACTGATAGGGAGTGGCAAAC

CGTGGAGCCTCCATTAGGGTTGGCCGGGACACAGAGAAGGCAGGCAAAGG

TTATTTTGAGGACAGGAGGCCTGCATCAAACATGGATCCGTACGTTGTCA

CTTCCATGATCGCAAACACCACCATCCTCTGGAAACCGTAAGAACGGAAG

CAAGAATTTCTATTGTTTTTTGGATGTGCACATTTTGTTTTAGTTGATTC

CAAAATGTATTTGCAGTTGTGGGTTTTGGACATTTGCATCTTCATTTGCC

GTGAATTGACAATAAGTTAATTGACATTAAAAAAAAAGCAGGCTTTTGCC

TTATTAGTAAATTCATCATGGTATGTTTGGCTGTT

>TEA015178.1 locus=Scaffold1951:533903:537744:+ probable aquaporin PIP1-4

ACTCACTCATTCACTTCAATCACTAACCATTCTAGAGGGAGAAAAAGTGT

GACATTTGTTGTTGTGTTTTAGAGAGAGAGAGAGAGAGAGAGAGAGAGAG

AGAGATGGAAGGGAAAGAAGAAGATGTGAGATTAGGAGCCAACAAGTTCC

CAGAGAGGCAGCCGATCGGCACTTCGGCGCAGGGCGGCGACAAGGATTAC

ATGGAGCCACCGCCGGCGCCGCTGTTCGAGCCGAGCGAGCTGACCTCGTG

GTCGTTTTACAGAGCTGGGATCGCCGAGTTCATGGCAACTTTTCTGTTCT

TGTACATCACCATTTTGACGGTGATGGGGGTTGTTAGCTCCAAAACAAAG

TGTTCGACGGTTGGGATTCAAGGCATAGCTTGGTCGTTTGGTGGCATGAT

CTTTGCACTTGTCTACTGCACCGCTGGCATCTCAGGTTAATTTCCCCACC

ATTTCTACTCCAATTTTTTAAAATTCTTTTGCTTTCATATTATCATACGC

CTGTTTGGAAAGGATTTTTGGACTAAAATAATTAATAAAAATAAAATAAA

TTAAAATAGTCGATAAATTATTACATTTAAATTATAATTATTTTAATTTT

TCTCTCATAATCATCTAAAAAAAATAAAATAAAAATCAATCAACTTCTTA

AACACTTTTTTTTAAATTTTTTTTTTTAAGTTAGGCTAAAATAACAAAAA

AAAAAAAAAGTCAAAAAAACTAACTAAAATAACTAACCTCCCAAACAAGT

CCAATATTAATTCATGTTCCATCGTAATTTTTGATAGTGATATTGAGAAA

TTATTAGTCGTGATATAATGATTTTTTTTTAATACACGTTTAATTTTTAT

TTCAATCAATAAATATATCGTAATATATAAATTAAAAAAAGATACTAATA

GGTAGTCAAATCAATAAAAATGTTCTTTGAAAAAGGTAAAATTTAGGCTT

ATTTTTTAGTTAAGAAAAGGGAAAATTACACATATGAGCTAATTTAACAA

AATATTTACGAATTGATGGTAAAATTTTTAAATCTAATTTTTAATATTTG

CAAAATTTACCACTAGTTTGAATTTTTTTTTTCAAAAGAAACACTGAAAC

GTGATTTTCCTTAAAAAAAATTAGTTTATTTAGTAAATAAATCTACAAAA

TCTAATTTAAAAAAGCTTGGTATATTCTTAATTTTTAAAATTTTCCTAAT

ACTTTTTTAAAATACCAAAGTTGGCATGTGAGGGAAATTTAATTTGACAA

ATGAATTAAATATTTTTTTAAAATAAATTCATTTTTCAACCACAGTGAAA

CCACCACTCGCTTTTGCATATTTGGGATTGATCGGATTAGGCCTCATTTG

GAAACTAATTGTTTAGTTTAAAAAATAATACAACAATGATTCATCCCCTT

TTCCCCTACCCTCTGTGACCCCCTCTCTCATTGGTTACATTTTCTTTTTC

TTTTTCTTTTTTTACCTGCTCCTCCAATCACCACCCGTCACGTGCCAAGG

GGGTGAGGGGTGACTCCTAAAAGGGGTCCCAAGCATTATTCAATATAATA

ATAATATATGACGTGATTTAAATTTTTTTTCCTCACAATTATTTATTTAA

TTAAAAAATCATTTGAACATACTATTTTATCTTCAAAAAATTATTACATA

TGCACAATTTTTTTTTTGTATAATTTTAATTATTAATACTAATAAAATTT

ATCAGTAATTTGTAATTATTTTTGAAATTACACACTGATGTAATTTTTTT

TTCTTTTTTTACCTCATTAAAAAACTCAAAAAATATCAGAAAAGGCCAAA

CAATCACTAAAAAAAAGGCTAAATTAAATGAACCAATATTTTGATTTGTG

GGATTTGTGTAGGGGGACATATCAACCCGGCGGTGACATTTGGGCTGCTA

CTAGCAAGGAAGTTGTCATTGACGAGAGCAGTGTTCTACATAGTGATGCA

GTGCCTTGGTGCAATTTGTGGTGCTGCTGTTGTCAAGGGCTTCGAGGGGA

CGACCCGGTACGAGCTTCTTGGTGGTGGTGCCAACTCTGTCAACCATGGT

TACACCAAAGGCGACGGCCTCGGTGCTGAGATCGTCGGAACCTTTGTCCT

CGTTTACACCGTCTTCTCCGCCACCGACGCCAAGCGTAGTGCCAGAGACT

CCCACGTCCCTGTAAGCACAAACACTTCTTCTATTTACACATAATTCGAT

TATAAAATCATAAACAACTCTTTCATGGGGAATGAAACCTATAAATTTGG

TGAGTCCCGTATAATCAAAATTTTCACAAAATTTCTATCAGTTATTTTTT

TCACTTTAAGTTTGATGAAATTGTTAAAATATGAATGATTGGAAATTTTT

TTTTTTGCTTTACCTGAGTTGAGTTAGATTAGGTATTTTTAGAACAATTT

TTTTAAAGAAAAAAATTTGAATCAAATCAAGTTTTTAGTCTAAATTTTTG

AACAAATTTGATGTGAAAAAAAAAAATAGAAGGGTTGGACATGCTTGAAG

TATACAATATCTAATATCAAATTGGAGGCAAATTTATCACATAAATCTGC

TTTATATATGCATATTTGTGTATCGATTGATTCGGTTTATAATTTTTTGG

TCACGGTGGGATGTAGATATTGGCACCATTGCCAATTGGGTTCGCAGTGT

TCTTGGTGCACTTAGCCACTATCCCCATAACCGGCACGGGTATCAACCCA

GCAAGGAGTCTTGGTGCTGCAATCGTCTTCAACAAGGACCGCGCTTGGGA

TGATCACGTAAGCAAACCACTCATTTAGGGTCCACACATGTAAAAATAAT

AAAGCATTGAGAGACGTCAAATTTATCACATAAATCTGCTTTATATATGC

ATATTTGTGTATCGATTGATTCGGTTTATAATTTTTTGGTCACGGTGGGA

TGTAGATATTGGCACCATTGCCAATTGGGTTCGCAGTGTTCTTGGTGCAC

TTAGCCACTATCCCCATAACCGGCACGGGTATCAACCCAGCAAGGAGTCT

TGGTGCTGCCATCGTCTTCAACAAGGACCGCGCTTGGGATGACCACGTAA

GTAAACCACTCATTTAGGGTCCACACATGTAAAAATAATAAAGCATTGAG

AGACGTTGATTAATGTTCTGCTAATTTTTGTTTGGCTGTGATTGGCAGTG

GATTTTCTGGGTGGGCCCATTCATCGGTGCAGCACTCGCAGCTTTGTACC

ACCAAGTCGTGATCAGAGCCATCCCCTTCAAGTCCAAGTGATGATCATGT

TTGGATCCACCGTATGATGCTTTTATTTGTGGCCATTTATTGTCTGTTGT

TTTGTTTTTAGGTGTGTATGTAATGAATGTCTTAGAAAATGTATTATATA

TCCATGTAATGGGATTTGTGGGCATGTTTGGTATTTTGATAATATAATCC

AAAAAAGAGGGGATGATGGTTGATGGGGCCCAATCAGAAGGTGGAGATGG

GGTGTCTGGATCATTGCATTTTGTGTGGAGTGGAGTGGACTCTGTAATGT

CAATTTGGTCACAAGACAAATAATGATTCACCGACCAAACCAAACCAAAC

CAAGGTCTACACTTTATTTTTTTTATTTATTTTTTATTTTTTATAATGAT

TTTTGGACATGTGTGTTTTTGTCTCTTTTTGCCGTTGCTGGTGCTGGATA

AGTCATTTCATGTTTGTTCCCATTATTGTGATTGTGCCGGTTGTGTTTAA

GATTTGTTCCAATTATTGTGATTGTGCCCGTTGTATTTAAATTTGGAATT

TGAACTTTAAATGATGTTCGGCCACAAAAATGATCTAGGAATGAATGTAG

GTGAGGTCTGTTACATTAAAAATAAGGAAATTGCTTTGGAGC

>TEA028587.1 locus=Scaffold2142:2844139:2852568:- Glycosyl hydrolase family 3 C-terminal domain

TGAAACTGCAAGACAGAAAGACAGAGCAGAGCTATCTATTTATATCATCC

AACCCCAGGCTCTCTCTGTACATCTCTCTCTCTCTCTCTCTCTATCTATC

TATCTATATATATATATCTCCAGAGTCTCCATTTGAGACCAAGCTTTAAG

AAGCTCCATCAAGTAAGTTCAAAGCATTTCTGCACTTTCTCCTCCTGCCT

CATTTCTTTGTTTTCCAGTGAACCTTGCTGTGCTCTGAGATCAATGTGTA

TATATATTGATAAGACTGACTAGATTTGACCTTACAGACTCTATGGTTGT

GCTATGATTTTTCAGATTTGAAGGCAGAGAGATAGTTGATTTGAGACACC

AAAATGGGGAGATTTTCAATACCCATCATGATTGGGTTTGTGGTGGTGTT

ATTAAGCTTGTGGGTAGGCATTACAGAAGAAGCAGAGTATATGAAATACA

ACGACCCAAAACAGCCATTGAATGTTAGAATCAATGACCTAATGAAACGA

ATGACGGTGGAGGAGAAGATTGGGCAGATGGTGCAGATTGAGCGCAATGT

TGCATCATCTGATGTCATGAAACAGTATTTCATTGGTATATGCAATCTTG

CTTTCCTTCATTTCTCTATAGAAATTATTCAATTTCTTCATATATATCTA

TCTATGTATACTATTATAAATTGAACACATAACTCTTATGGTGTCCTGAA

CCTTTACTCTCCTCATGCCATGTGGCCGTATTTTTTATTTTTTTAAAAAA

AGTTAAACAAAGAGATGGGCAAGGCACCTTTACTCTCCTCATGCCATGTG

GCAGTATTTTTTATTTTTTAAAAAAAAGTTAAACAAAGAGATGGGTAGTA

TTTTTTATTTTTTAAAAAAAAGTTAAACAAAGAGATGGGTAAGGCTTGAA

CCCATGACTATGGGTTTAAGTACGAGGACTCGTACCACTTAAACTAAATA

CACATTATAATATTAATTTCATTATTATTATTTTTATTCCTACTTATTTA

AATTGCACACACATCGTGTGTGCCACCCACTAGTATATATATATATATAT

CTTCATATGGTTATTTACCAAACCCACGTTTTTTTTTTATTTTTTAATTT

CTGAAATTCAAATAGTTCTCAACTTATTTTTCAAATCCATATCTTGTTTG

TTTTTTTGGTGTTAAATACTTTTGTTTATTTTTTTGGATGGTTTGGAGTT

TGATTATTTTTTTAATTTATTGATTTTTTTACAAAAGGATGTGAAAAAAA

AATAAAAATAAAGAGGTGATGTTATAATTTGTAGCAAATAAGCTGCTTAT

TAGTAGGTCCCTCAAAACAAAAGAAAAAAATCAATGGCCTTGCTTGTTTT

CACTTTTAAAAAAATATTTTTCAAAAAATTATACTTTCTTATTTTCTGTG

TTTTTTTTGTTTTTACTGAATTTTTTTAATATTTTAAGCAATTTTTTTAC

TTTTTAGACACACCTAAAAAGTAAAAAAATTGTAAAAAAAATATTTTAAA

AATTTATTAAATACTAAAAATACAAAAAAAAAAAATATAAGAATTCAATA

TATTTTTTGTATTTTTATGTATTTAATTAATTTTTTAAAATAGTTTTTCA

CAATTTTTTTACTTTTTAGATACATCTAAGTAATCTAAAAAAGTAAAAAA

ATGTTTAAAATTTAAAAAATATTTACTAAAAACAAAAAAATACGAAAACC

AAAAAAGTCAATTTTCAAAAAATTTAATCCAGAAAACTCAAACCAAACAC

ATTGAATAAATTTCGGTCTGTTTTGTCGTGATATTTTGAGATTTCTTGAT

AAATATTTCACTTTTTTAAGTTTAGATCCACTTAAATTTTTTTTAAACAA

ATATTAAAATATGAAAGAAAAAAAAATCACTAATAACAATGAAACAAAAA

ATTAGAAAAGTGATACATACCCATTAAAAAATTTTAAAAACTAAATCAAA

CAAGGTATTGAAATTGTTTTTGAAAACACTTGGAAGTAGTCCTGTTTGAA

AACACAATTTCATGATAGAAATGACATCATGAGTTTGAAATAAGTTAAAA

ACTGCTTGAGGCACCGTTTGGGAAGGTGGGTTGGAGGTTTTTTTGGCTAT

TTTAGCCACAAAAAACAAAATAGGCTATTTTAGCCATAACAAACAAAACA

TCACATCCAACCCATAATCATTACATTCTTCTCTCACAAAAAAACTCAAA

AAAGCCAAAAAGCTAAAAAGCCCTTCCCAAACCAAGCTTTTAAGCCCAGG

CTAAAATAGCCAAAAAAGCCAAAAAGTCAAAAAGCCAACCCACTTCCCAA

ACGGAGGCTGAGTTTGGTTAATAGAGATTTTAAAAACTCTTTTATCTTCT

ACAATTTGGTGTTCTGTAGTTTCTTACATTGTCTAAACTACTTTCAGTGT

TGTTTAGAGCCATTAATTGTTTATGTGAATGTGTTGATTTTGTACTGAAG

AAGAGGAATTGAATTGAGCAGGAAGTATACTAAGTGGTGGAGGCAGTGTA

CCGGCTCCAAAGGCTTCTGCCGAAACTTGGGTTAATATGGTGAATGATTT

TCAAAAGGGTGCTGTCTCTACCCGTCTTGGAATTCCTATGATTTATGGGA

TCGATGCTGTCCACGGCCACAACAATGTCTATGGTGCCACCATTTTTCCT

CATAACGTTGGGCTCGGAGTTACCAGGCAAGTAGAGCTGATTCTAGGACT

AAAAATTGTTGATATAACCCTTTTTAGATTCCATAATATTTCTCTTTGGG

TTCGCAGCAATATTTAGCATTGATTTATGGAATGATTCTGGGACTAAAAA

TGTTTTTTAGCCTGACAGGGTTTATTAGCCTAGATATGCTTATCTTTTGG

TTCTCGTGTGAAATGCGAAAGGAATTTGAATCCTGGTAGTCTCATATGTG

TGGTCTGTGTGTGGTGAACACTTTTCAAGATCACACTAAAGAATTATTAG

TGACTAATACATTATATTATAACTTGATAATTTAAAGGGAATTTTGTATA

AAGTAATACTAAATTGACAACTTAGAACTTGTGTGTTTGAATAGGATAGG

TTTTTGACTTTAAGAGATTTCGTAAGTATTGATTTATTTTCAATTTTATG

TATGTTGAAAATTTAGTAATTTGACAGCTGGGAAACTTGAAAAATGGGTC

ATTTGATTTGTTTGTTTAAGCTTGTGTTTTAACTTCAAAAATTTCAAGAG

TAGGTAATTTAAAACTTTCAGCGTTTTGGACTTTACCAAAAAATTGACAG

CAGGAACTTTGAAGAATTGGCTCATAATATGTTTATTTTGGGCAAAGTTT

CAATGGCAAAAAATCTCATAACCAATGATCATGATCTCCTTCCAATGCTG

AAACATGATTGGTTTGACTCTAGAAACATGATTCTACCTTTATGTTTCAA

TTCTTTATTTGGTTTAAGTTTTAAAAAATTGATTCCAAGACTACATTTTA

ACACACTCACTTTAGAACATGTTTGGCAAAGGAGTGAATTTTGAAAAGAG

GGGATTGGATCTACCCCGGTTGTTCGCTCGGACATCCGAAAACATCTTTT

TGGATGTCTAGTGGGTTGACCAAAATAGTATTTTTGTTTTTGTTTTTATT

TTTTTGTGTTGCAGTTGACATCTATATTTGTTTTTGATATTTTTTTACAT

ATAGAAATTATTTTTTAGACCAAATTATTTGGCCATTCATTTAAAATTAA

CAAAAAAAATTTAAATAAAAATTAATTTGTCACAATAATTTTTAAAGAAT

AAAAAAAAATTTAAACTAAACAATTAAAATAAATTTTCAAACAAATATAC

AATCAAAAGTAATTTTAAAAAAAAACTAACTAAATATTTTCGTGTTTTTT

TATTTTTAGTGAATATTTTTAAAATTTTCAATATTTTATTTTTTATTTTT

TTAGATGCACCTTACTTAGATATATCTAAAAAGTATAAAAATTGTGAAAA

AATATTTTACAAAATAAAAATATAAAAAATGTACTGAATTCTCGTATTTT

TGTCGTATTTTTGAAATTTAGTGAATTTTTAAAAGGTTTTTTTCACAATT

TTTTTACTTTTTAGGTATATTTATGTCTAAAAATTAAAAAAATTGCTTAA

AATATTAAAAAAATTTATTGAAAATATTAAAATACGAAAAATGAAAAAGT

AAAATTTTCTGGAAACCGTTTTGTAAAAACTGGAACCAAACAAGCTGATT

TGGATTTTTTTAAAATTTTTTAAATAAAAAAATTATTTTTTTAATAGAAT

TTTGATTATGAGTTAAGTATAGTTATTAATTTTTAAAACTCATAATTATT

CCTACAAACTTTCGAGCTGTGGTGCCTAGTTAAAATGAAATTAGACGACC

TTGAAGTGCTCGCTTGCATTTATTTGCTTCCAATTTTAAATCCACTAGTT

CCAATTTTTTGTTGGCTGATCTGTGTTGAAATTTATGTCGACTTTTTAGG

GATCCTATACTTCTCAAGAAGATTGGGGAAGCAACTGCACTTGAAGTTAG

GGCTACAGGAATCCCTTATGTTTTCGCTCCATGTATTGCGGTAAATATAC

ATTATTAGAGAACACAACAAACTAACAACCTTGTTTGAATTATAGTTTGG

TTTGGTGTCTGATAATTCTGTTTCTACAGGTCTGCAGGGATCCAAGATGG

GGCCGGTGTTATGAAAGCTATAGTGAAGATCATAAGATTGTTCAACAAAT

GACTGAAATCATACCCGGCTTACAAGGCGATCTCCCTACTGCAAATTCTC

AAAAGGGTGTTCCTTTTGTTTCCGGAAAGTATGAACCTACATCTCCTTTT

ACCTCTCTCCGCTTTTTCCACCAGGACAATTGTTATTAACTAGTGATGTT

TTATTAAGGCCAAGTTTGGCATCCCCATATAATTTATCTGTTTATCTTTT

TTTTATCACATTACTTCTTTACTTTTCTGCACATCTTTTCGTAAAAAAAT

AAAAAGTACACTTATTTAGATCAAGAAGTGTGTCGATGTTTTATTAAGAC

ACAGACACGTGACACGATACACTTGTCAAGAAGTGTGTCGATGTTTTATT

ATCGCAGGACTATTTTAGAAAGATGATTATAATCAATTTAAAAAAATTGA

TTATAAGTAACTTTTTTTTATGAGAGAGAATGTGAGTTATAATTTGGATA

AGGACTTATAATCCACTTTTCAAAAGGGGTCTCCGAAGTGTTCGTACTGC

TTAAATTAAATGGTCCCGGACAAACTCTTTGATTAGCATATACTTTATCA

TCACGTGGAGTGCAAATCTTTTGACCTTGAAGTTTCTCATTCGATTTTCA

AAAGTTAAAATACTTTCCATGTAAGATTAATTGCTTGTGGACATATATTT

GCCAATCACATGATGGTGGGCCTAACATTTGCTAAAATGTTGCACAATGG

ACATTGAGAAAGGACAAAAATATTAAAGTTTGTCCCCTATATTTTAGCTT

CATTTTTTTGTAACTTGATAGGATGTGTTCCTCGTGTCACTCTAATCATT

TTCTAGTTGAATGTGACGGTTCGGTAGCTAGCTGTGATGGCGCAGCCCTT

GACTGGTTGGAAGTCCATTATTGGACCTAGGGTGAAGTCCGTGAGCCTGA

AGACCTTCTAGTCTCATAAATGTCATTGATAAATTAATATTTTTGATATT

TAGTTAGATTAGTTATATTATTATTTATAAGATATTTCTGATTGTGTTTC

CAAATAATAATAGGGATAATTATCTATTCGACTAGAGTTAATAGACTATT

ACATATGCTAAGCTTTACAGTGTAATGATCAGCTTTTGATTTATAAGATA

TTGAGTTTAAGAAAGACTGACCGTTTGAGTTCTTCTCTCTCCCTCTTCTC

TACTGTTTTCACATCACTTTCTTACTTATATATTTTCACATCACTTTCTA

TTTTCTTATATACCATGTACAATATTATCTAAATGTTTATCTGTTATGCT

TGCAAAGGTTTCGTCTGTACCCAAAAAAAAAAAAAAATGTAAAAAGTTAG

TTGGACTGAGAAATCAAATGTGTCATTGCTTATTTCAATTTTGTAGAAAT

ATGATTTACATGTTTCGTGGCCAGGAAAATCACATGGGCGAACTGTTTCT

AAGATTAATTTGAAAACCATTAAAAGCAAACGCCTGACTGATATTTTTTT

TTTATTTTTTTTTAATGAAGGACAAGAGTTGCAGCCTGTGCTAAGCACTT

TGTGGGAGATGGTGGCACAACCAAGGGCATAAATGAGAACAACACCGTGA

TTGACTTCCGCGGATTGCTCAGCATTCACATGCCCGCATACATTGACTCT

ATCAGGAAAGGTGTTGCGACAGTGATGGTATCTTACTCAAGCTGGAATGG

AGTAAAGATGCACGCTAACCGTGACCTTGTCACTGGATTTCTCAAAGACA

AGCTCAAATTCAGGGTAAACGAAAAAAATCTATTCTATTGTTGATTATAT

AAAGCACACAACTGACTAGGATTGAAGCTTTGTCACACAATTTTGATTCC

AAAAATAACTTTAGCAGCGCCATATTTTTAGTGATTTTTCAGTTGAAAAT

CAGTGGAACGAAATTCTAGCTGTAATTCTTTTTTATTCTTGTGTTTTCAG

GGTTTCGTCATTTCAGATTGGCAGGGTATTGACAGAATTACCTCCCCGCC

CCATGCTAACTACTCGTATTCTGTTCAAGCTGGAGTCCTTGCTGGAATCG

ACATGGTCAGTCAAATTGCATTTGTGATGTAGAATTTAGTAAATATTATT

CTATGATCATTGAAAAAATTGATACAAGTTGACATTTTATCTTTTATTTT

TGAATTGTAGGTCATGGTCCCGAACGACTTTACAGAGTTCATCAATGATT

TGACCCTCCAGGTGAAGAACAATATCATCCCAATGAGCAGGATTGACGAT

GCTGTGAAGAGGATCTTGAGGGTCAAATTCGTCATGGGACTCTTTGAAAA

TCCAATGGCTGACAATAGCTTAGTCAACCAACTGGGAAGCAAGGTTCATA

TCATTGTGAACTCGAATATCTGTTCTTTATTTTTAATTTTTGTATATATA

TAATTAATTGTTATATTTTCTATTCACTTGGAATAGGAACATAGAGAATT

GGCTAGGGAAGCTGTGAGGAAATCACTCGTGCTTCTAAAGAACGGTAAAG

CTGGTTATAAGCCAATGCTTCCGCTTCCCAAAAAGGTGCAAAAGATACTT

GTTGCTGGAAGTCATGCCGACAACTTGGGTTATCAGTGTGGAGGATGGAC

AGTTGAATGGCGGGGTGTTAGTGGCAATGATCTCATAGTTGGTATGTTTT

CCAAGTTTATTTTTCATTAAAATGTTTATGATTTACAAAACTTTTCTTCA

AGAACAAATTAATTCTTAACCAGTGCAATGGGAATTATATTGGATTAGAG

TCTTGTACACTGAATCGCTCTTTTATACTGTATAGGATAGTCACTTACAT

ACATTCTCTGTTTGGCAACAGATGAGAAGTCACATATTTTTTTTTTCTTC

AATGAATTTTGTTAAAATGGTTTTTTTTTTTTGTAGTTTCATCTAGTGTA

GAAGGATATAAAAAGCAAAAAACAAAATAACTACTGCAACACTACTATTA

TAACCATTTCATCTGTCTCATGAAACAACATTTCATTAACAGGTACATCA

ATCTTAACTGCTGTGAAAAACACTGTTGATCCAACTACCCAAGTCATCTA

CAACGAAAACCCAGACGCAGACTTTGTCAAGTCCAACCAATTCGCCTACG

CCATTGTGGTCGTGGGTGAAGCAACCTACGTTGAGACATTTGGCGATAGC

ATGAGCTTGACGATGATAGATCCCGGGCCAAACACCATAAAAAACGTATG

TGGTGCTGTTAAATGCGTTGTCATCGTCATCTCTGGCCGCCCTGTTGTGA

TTGAGCCCTATGTCGCGACAATGGATGCTCTTGTCGCTGCCTGGCTTCCA

GGAACTGAAGGCCAAGGCATTGCTGATGTTTTGTTTGGTGATTATGGATT

TACTGGCAAACTTGCGCGGACTTGGTTCAAGACGGTTGATCAGTTGCCGA

TGAATGTGGGTGATTCGCATTATGATCCTCTGTTCCCATTCGGGTTTGGT

CTGACAACTCAACCTGTCAAGAATTGATATCGATGATTCACAATCGCATC

TATTATCCTCTGCTCTGTTCTTGGAAAACTAATTGTTTTGAGTTGATTTG

TAGAGAAAATTCCTAATTCGGGTTTGTTTGGTGATTATGATATATGATTT

TGGTGTTAACGAAGCCTAATTCCTGTTTGTTCAATTCATATATATGCGGA

TATAGTTATGGTAGTTATTTTGAGTGTATGATTACATACAATGATAATTA

ATATATTTAAGTGATTTGGTGAGGAAAAATTCATTTTTGAGTAATGTACT

GTGATAAATAAAAATTATATACAATTCCCC

>TEA015536.1 locus=Scaffold2145:2574785:2580939:+ prostatic spermine-binding protein-like

ATGGGCTCAACAATGTATATCAAGCTATCTATCAAGGACATTGAGGAATT

GGATGACACCGTGGGTGATGGGGATGATGATGGAGACAATGACACCAACA

ATGTTGATTGTTGCAATGATGATAACTTTGCGAAGATGATGACTTATTTT

TGTCTCTTATTTGTCCTCTTATTTTAGTTTGTTTTCTTTTTGTTAAGAAA

GGATGTGAACGTTATGAAAGTGTTAACTTGAATTGCTGTCATCCTTTGTT

ATGTGTTTTGTGTCAAGTTTTGAGAAAAGTCTTGAATGATAATTTGATAA

TTACTATCTCTTTATTAAGGAAAGATGTAAAATGTTAAACATAAATTGCT

TATTGTTAGTGAGCAAGGCACACAAGTGAGAATGTGAGGATTTGAACCTT

GATCGGCTCATACTGAACTTTATAATTCACAAATTTCAGAGTTGGTATTA

GAGCTTTATCATTTGTACATCTTCAATTTTTTTTTTTTTTTTTGAGTCAT

TCTAAATTTAATACTTTTTTTCATTATTTTAGTAGGATCTTATGATCTTT

GATTCGGTCCTACGATCTGATCCGATAGACCCTTGAAACAATCCTGTGTA

GGATCCTGATTTTAGCAACCTTGCTTGTTGCTTTATGTGGATGACATGAT

TACTGGCTTTGATCCTTCTACCATCACTTAGGTGTGGTGCCCTAGGAGTA

TTTTCTCTCTCAAGCTAAATACGTTAATGAGGTTATCTACCATGCCCGTC

TCTCCAATACTAAGACTTTTGATACTTCGATTGATCTTAATGTGAATCTC

AACACTGCTGATGGTGTTCCTCCGGATGATCCCATTTTGTATCGGAAACT

TATGGGTTGCTTGGTCTATCTGACTATGACTCGCCTTGTGTGGTCAGTCA

ATTTGTCTATACTCCTTGATCAACGCACTGGGCTGCTTTAGTTAAGATTC

TGCACTGTCTTCGGCATACTATCTTCTAGGGCTTACTGTTATCCTCTACT

TCTTTGAGCTTATGCTGATTATGATTAGGTTGGTGATGTCACTTATCACA

ATTCCATTTTTGGTTTCTGTGTTTCTTGGCAATTCCCTCATTTCCTGGAA

AAGCAGGAAACAAGCGGTTGTTGTCTCTTGTTGAAGGTGAGTACCGTACT

ATGACTCATGTTACTGCTGAGGTTGAGTGGCTTTGCTGGCTTTTGTCTAA

TTTGAGAATTTCTTAGTCTTCTTTGACTTTGCTTTACTTTGACAACAAAA

GAACCTTCTAGATTGCCCATAACAATATCTTTCATGAGAGGACGAAACAC

ATTGAGATTGATTGTTACTTGGTCTGTTAGCATCTTCAGTCTAGATCTAT

TTCTCTTCCCATTTTCACTTCAGTTCTGCAGTTTGTTGATTTATTCATAA

AGACTCACTACTGTTCGCTTCCGGTTCCTTCTAGATGAAGTCTATATGTT

TTATGGCAAAACACATTGGGTTTGAGCGGGGGTATCAAAATTGTAGATAT

GCTCCAGATATTATGTATATTTTGTAGATATCATAGATATTCTGGAGATA

TTGTGAAGATATTATTCTGTGAGAATATTGTAGAGTATTCTCTAGATATT

ATGTATATTCTAAAGATAATGTAGATATTTGGGGAGATATTATGTTGTGT

AATATTCATGGAATTGTTTTCTTTTTTGTTTTATGGTTAGCCTAGGCTAT

AAATATTGATGTAATTATTTTGTATTTGACATAATTGAATATACAGTTTC

TTATGCTTAGAAAAATTTAAAACTATGAACCCTTTCCAGCTCCATTGCAA

TTGTGTAATGCATTCTAACCCTCAGTGTGATGCCTACATGAATTGGGCTT

ATGTATAATATAATATTAACATACAAACGTATCCTAACCATACCCGTATC

TTATTTTTAAAATTTTACTTTATTGCTGTATTTGTATTGTATTGTGTCCA

TAACACGTATTTGTATCCATGCTTCTTAGGTGACCTTAATACAAAGCCTT

AAGAACCCAATATCAATGTTTGGTTGAATTTTCATAACTTGGTTTTTGAA

TTTTTTGTAAAAATTAGAAACTACTTTGACGAATATACACACCAAAAAGG

GTATTTTTGAATTTTCAAAAAATTATTATGGTGCAATGTCACGACCCAAA

CCCGCCTCGAGAAGGTTGGCATCGTAACCGGCCAGTAATCCTAGATTACC

ACAGGCCTTTCTGTACAAAACATACATAACCGGGGCTTGCGGAAGCGAAA

ACAAAACACTAATAGTCCAAAGAGCTGTGCATTTATATGAACATACATTA

CATGCCAAAAATGTTAATTTACCCACTACATTACATCACATATAATCAAA

ATTCATATACAAGACATAGGCTCACGCCTCATGTACAACCAAAACCAAGC

TTCCTAACGGCCTTCTAGGCCTAGCCCCAACTCGGAGGCACCAAAACACC

GGGTAGGCTTCAAAACGGCGGCTCAGCTCGGACTCCGCCTAGGCTTGACT

CCCAGAGTGCTCACCTATAATTACATATATACAAACAATGAGCTAAAGCT

CAGCAAGTAACATTAAGGTAAAGGGGTAGTGAACGAATTCCACATTTCAT

ATATCCATAAAATAAATCATTCATAAACATAATCATCTCATATCAGAACA

TACATAATGGCATACTCCATAGAACATAACATTTGTCGTCCAACGACTAG

AGTAACCGGTCCCGGACACCGATTAAACACATACCGAGATCTCGTAAACT

GGTCCACTTAGGACTATCTATGGATGCCATACTAGATTCGCAAACTGGAC

ATTCGTTCACAGAAAACATTTATCATTTCAAAACCATAGCATAACGTAGC

ACTTTTAAACATAGATCATACTAACATATCTTTACAGAAATGTTACTCAC

AGTGGGTTTCCAAAACCGGTTGCACGGTCTTCGCCTCTTGACCTGCAATT

ATCAACATTAGAACACATTTCTCCCATACACATAAACCATGAACATGTAC

ATACAAGCTTCATTTCTTACATCCATGGAGGTTCTCCCTCAAATGTGACA

AACATGCAAGGAAACCATGATTTTGACCATGAACTTCATACTATTATGCC

ATAATCTTGAGATCAACATGCTAGAGGCCAAGAAGCAACAATCTATTTCA

TGAAAATCTTACCTTAGATGGTGAATTGGAGAAGTTAGCTTGCTTTTCCC

CCTTTTCTTCTATCTTCTTCTCCTTGGTTTCTCTCTTCCACGAATTCACT

CTCTCTCTCTCTCTTAGATATTTTCTTTCTTTTGTTTTGAGTCAAAACAT

GGTCAACATCCTTATCTATATGTACATATATGTCATAAAAATAGAATTGT

GCCATAATCTTGAGCCAAACAAGGCTTAACGAATCAGACGAAATTCAGTC

TTCAATTTATTGTCCCAAATGGCTCTAAAAATTCCCGGTATCACATAGGC

GTGCTCAATTTCGTTTTCTGAACCCATTACGGTCATCAATTTCAGTCCCC

CAAAGTTCTCATCTCAACAGATATTCTTCTAATTCAAACAGATATTCTTC

AATTAATTCGCATAAACACATAATTAATTACTGATAAATATGACACATGT

TTTGGGGTCATTACATGCAAGCTATATTACATGGACTCTTTAAAATAAAT

CAAACACCTGTGTTGGACATTTGACACTTGGACAAGGCATGGGATATGTG

TCAGATACTTTTCATCGGACACATATATTTGTATTTAGCAATATCATTAT

ATGTATATATTGTTGTATAATTTCTATAAAATGAGTAAAATGCCTTTTTA

GCATTGTATGTTACATATAACACCATTTTATCCAACTGAATAGATTAATA

CTATTGAAATATAACTGCTTTTTGTTGAGTTCGCGTATCTTTATATTTGA

GATTTTATGGGTCTGATTTCTAGATATGCACTTGCACCTGCACCCGACAC

CCCTACCCTAGTCCATGTAACATAGGGTGCAACTGTGCAAGACAGGTTCT

ACATACTATGATATACTATGCAAAAAATGCTTATAGAATTTGATTTAGAA

AATGTGGCAGGCAAATTCATTTCTTTGAACTGACATGAACATTGGATTGA

TTTTTACGAAAAGGCTGTTCCTCTTGCACCATGAAACACGAAGTTCCAAA

ATTTGATTGCATTTTCTTGGTTAATATTTTCCTTAATGTATTATGCTTTA

GTTGGTTGCAGACTAAATTATTTTTCTTTTTAGTCTTTAATGCTATAGGT

TCATTTTGGTGAATTTGTTTTCTAAGTTGGACTTTATTTCTTTTGTTGCA

TATCTGGACAGACTTTCATGTGTGTAATAAACACAACGGTTCATTTATTA

TGCTCCTCGTTCAGAGGTGTCTTGTTCATGAATGAAAACATACAGCATCA

AGCATCAAGTTTTGCTACTTTTTCTTTCATTATTTTATCATTGTAATCAA

TGTGGTTAACCCATTAAGGATTTGTGTCATGTTCTGGATCTCCGAACATA

GAATTCAGCTATTGATTGCAGTCTCTGGCCATCTCTGATAATCCTAAACT

GCATCAGGTGCCTGTTAATCATGTTGGGAATGGTCTGCAGCATAACCAAT

CTATTCCAAATAGCTCTTTCCTGAGTGGTATCAAACAGCGCTTGCTTTCA

TTCATATTCCGTGGGATATGGGCTGAAGAAACTGATCAAACCCTGGTTGG

TCATGTGACACTCAAATTGTTTTTTATTTTTAATTTAATGATTTAAATTT

CATGATTGTGTAGCTCATGCAACTTATGCTAGTCTGCTTAATGATTCTAG

TAAAAGATGTGTTAAAACCTACCACTACCACTACCACCTACTACACCTAC

TCCACCCACTTCACCTACTCCACCTACTCCACCTACTCCACTACATCTAC

ATCTCCTATATAAGGAGTCCCATACCATTTGTAATAAATAACATACAACA

ATCAATAATACATCAATTTTAACATGGTATCAGAGCAGGTTTAGAACCTA

ATATTTTCGCTTCCCCGGCGGGCGGCAACTTCACCTTAGCCGCCCACCGG

ACCTCAACCTAGTCCGCTAGAATTTTCTACACATACCTTCACTCTGCTTC

ACCACCTCGCCGAAACAAACCCAAAATCCCTCTGGGACATTTCGTCGAAC

AACATGTCTGCAACAATGATGGGCATGACACACACACAGCCTGATCCACA

GACCATGCATCACACATCTCTCTCTCTCTGTGTGTGTTTTTAATGCAGAG

CAAGAGAGACAGAGAGAGCCATGTCTTTGCTATCAGATCTCATCAACCTC

AACCTTTCGGACACTACGGAGAAGGTGATTGCTGAGTACATATGGATTGG

TGGTTCTGGCATGGACCTCAGAAGCAAAGCAAGGGTAATAGCTTTTCTCC

CTAATAAAAAATTGTTTCTGGTTATTGTGTATGGTATCTAATGTAATCGA

TTGTGCTATATAGACTCTTTCTGGACCTGTGAGTGAAAAAAAAAAGCTTC

CAAAGTGGAATTATGACGGATCTAGCATAGGACAAGCTCCTGGTGAAGAT

AGTGAAGTCATTATCTACCCTCAAGCAATTTTTAAGGACCTATTCAGGAG

GGGAAACAATAAATTGGTCATGTGTGATACTTACACACCTGGTGGTGAGC

CAATTCCAACAAACAAGAGGTTCAACGCTGTATCAAAGCTGGAGATGAAT

TGTGGGTGGCTCGTTACATCCTTGAGAGGGTCACGAAGATTGCTGGGGTC

GTGGTTTCCTTTGATCCCAAACCTATCGAGGGTGATTGGAATGGCGCTGG

AGCTCATACCAATTACAGCACCAAGTCTACGAGGAGTGATGGGGGTTTTG

AGGTCATCAACCACCGAACCCCGACCATGTTCCGACAAGCGCCTCAGCTC

ATGGCACGAGCCACCACGGTGGCGATGAGGTGTCACTGCCAGTTCTCCAC

CGACGTCCCGGTGGAAGCTTCCGCGGACTCGACGTTCGTGGAGGCGTGGA

GGAAATTGATACCAAACATTGAGCCACCGAAGACTCCGTCGTCCTTCATG

GCTCCTCGCCCTTCTACGACTGCCTCCATCCCTACCAAGCTCACCGTCAA

CTTCGTTCTTCCTTATTCCTCTCAACAGAGCAAAGATGACCCAAATTATG

TTTGA

>TEA010322.1 locus=Scaffold2408:1026336:1027968:- leucoanthocyanidin dioxygenase-like

GTGGCTACTGTGGCAGGTATAAGAGTCGAAAGCTTGGCCAGCAGCGGCCT

TGAATCGATTCCAAAAGAGTACGTAAGGCCGCAAGAAGAGCTCACTAGCA

TCGGCAACGTATTCGAGGAAGAGAAGAAAGAGGAAGGGCCTCAGGTTCCC

ACAGTGGACTTGAAAGACTTGGTGGCGGAGGACAAGGAGGTGAGGGAGAG

GTGCCGCGAGGCGTTGAAGAAGGCGGCGACAGAGTGGGGGGTGATGCATT

TGGTGAACCACGGGATACCGGATGAGCTGATGGAGCGAGTGAAGGCGGCC

GGAGAAGGGTTTTTTAACCAGCCTGTGGAGGAGAAGGAGAAGTATGCAAA

TGACCATGATTCTGGAAATATTCAAGGGTATGGAAGTAAGTTGGCTAATA

ATGCTAGTGGTCAACTTGAGTGGGAAGACTATTTCTTCCACCTTGTCTTC

CCTGAAGACAAGCGTGACATGTCCATTTGGCCTAAGACACTATCCGACTA

TATGTAAGTTTACTTTAATGCTACATTTATTAATATACGTAATCTCATTT

TTTGTTTAATTTTTTCATTCCTAAATATAAAATAATAGTATATAAGACCC

ACTTAAATATTTCATAACTATTGATTTTGAAATGGACATTTCTGTTGTAT

TCATGCAATAGAAAGGATTGTATTCATTTAATCTGAATTTTATTTTTATT

TTTTTAATCACTTAACGTTGCATAGTTATAGTAATTGGATGTTAATGGAA

CTCAAGTGGGATTTATTGTGTTAATTATTGTGAATTTATCACATCAAATG

ATATGATAAAAAAAATTATTATAGTCATCCATACGTTATTGATAAGTTAC

TTACGCTTTATTAATTATGCTAAAAAAATATCAAAATTACTTAAATTAAT

TATATGACCTATAATCATTCAAAACAATAATGTTTAGTTTATAGAAAAAT

TAATAATTTTTTTAATCAGCAAAAAAGAAAAATGTGGTCCACTCAACATA

TTCATATAGAGAGGTGATTGAAACCCCACCCCAACCACCCCACACGCAGA

TGGGGGTTGGTGAGTGAGAGAAAAGGAAAAAAACAAAAAATGTCCAATCA

CGTCACAAAAGGGGTAGTAGGGTCACTCTCTGTGGGAGTTCGAATCTTTT

TCCATTCGTATATTCGCAAACATTCCCTTATTCAAATTTGTGTGAATGCA

TTTTATTACACGCAGTCCGGCAACAAGCGAGTACGCAAAGCAACTACGAG

GTCTAGCAACAAAAGTCCTGTCGGCCCTCTCACTCGGCTTGGGACTAGAA

GAAGGCCGACTAGAAAAAGAAGTAGGAGGCATGGAAGAGCTTCATCTCCA

AATGAAAATAAACTATTACCCAAAATGCCCTCAACCAGAGCTCGCCCTCG

GCGTCGAAGCCCACACCGACGTCTCTGCCCTCACCTTCATCCTCCACAAC

ATGGTTCCCGGCCTGCAACTCTTCTACGAGGGCAAATGGGTCACTGCCAA

ATGCGTCCCCAACTCCATTATCATGCACATTGGTGACACCGTAGAAATTC

TCAGTAACCGCAAGTACAAGAGCATTCTCCATCGTGGACTCGTTAATAAG

GAAAAAGTGAGGATTTCGTGGGCGGTTTTCTGC

>TEA003892.1 locus=Scaffold3533:267116:279475:- glutamate synthase [NADH], amyloplastic

ATGTCGGTGATTTCGAATTCTGTTGTTCAAACTCGAAACAACAACAACAA

CTCGGTGATTCCTTCGATTACCAAGCCTTTTGTGGCTTTGAACCGAGTTC

GTATTACAAAAGCTTTTGTTGATAAAAGGTCTAAAACAAAATCAAATGTT

TTGGAGAATAGATTTTGTGGAACCAAATTGAGAGCTTGTGAAAGATTGCA

TCTTTGGCAATCGGATGGTCCGGGTTTGGATCCGAAGCTCAAAGTCGTGG

TGCGATCAGCATTGTCGCAAGTTCCCGAGAAGCCTCTCGGTCTCTATGAT

CCTTCGTTCGATAAGGACTCGTGTGGGGTGGGGTTTGTTGCTGAATTGTC

CGGAGAAAGTAGCCGGAAAACGGTATATAATAGTTTTGGGTGTTTGGTTT

TTGAGATATGAGTTTGATTTTTATGAGTTTTAAGAATTGTTATTGTGTTT

TGATTTGAAAAAGGTGACTGATGCGGTGGAGATGTTGATAAGGATGTCAC

ATAGAGGTGCTTGTGGATGTGAGACTAATACTGGTGATGGGGCTGGCATT

CTTGTAGCTCTTCCTCACCAGTTCTACAAGGAGGTACACTAATCTAATAT

CTCATTTTTGTGATTTTTATATCTGATTTTGGATAATTATAAATTTTATG

AATGAACACCCAAAAAGGAAAGAGAAGAACAGATAGAAAATTAAAAATTC

TACTTAAAATGTGGATTAGGCATTCTAAGACTGAATTTATTGGTTTTGTT

TGATTGCTTGCTATTTGGGTTCAAACTCTCAAACTCATTTATTTTGCTTG

ATTTCTTCCTTTTTGGGTTCAAACTATCAAATTCAATTCTGTTCTGTTGA

TCGATTTCTTGCTATTTGGGTTCAAATTCTCCTACTTATTTTTGTAATTG

TTGTCGGGTCATCGTTTGTCAGGTTGCCAAAGATGTGGGTTTTGAGCTAC

CACCTCCAGGGGAGTATGCCGTGGGCATGTTCTTCTTGCCTACATCTGAA

AATCGAAGGGCACAAAGCAAAATCGTATTTACTAAGGTAATTTTAATATC

AAAACTTAGTTCAACTTGTGCTTGTAAGTTGTAATTGGAAAAGTGAATTT

ACCTAGACCATCTTCTTATTTGCTTTTCATAGACTAGTATGTTTTGGAGT

TTTTGAGGTCGTCTCCTAAGAAAATTAATGATTGTATTTCTAGGTTGCGG

AGTCTCTCGGGCATACTGTCCTTGGCTGGCGCACTGTCCCAACAGATAAC

TCAGGATTGGGAAACTCTGCTTTGCAGACCGAACCTGTTATTGAGCAAGT

GTTTCTTACGCCCACTCCTAAGTCTAAAGTCGATTTTGAGCAACAGGTAC

AGATCTATTTCTTTTTAGCTATACATTTCTCTTCAGAAGGAAACTGTGAA

TTTTGCATCTTTTGGAGGTCTACGTGAGATGTATAGTCCTATTAGACGTA

ACAATTGTACATGAATGAAACATCGGTTGCTCTTGATCAAGAAGAACTGG

ATGATCCTAGTTACACGATCAGTCGCAGTCTCTGATCTTTTTATTAATGT

TATATGGAAGGAGGATAACGAAACGAGCAAGCGAGGGAGGAAAATAGAAC

TTCTGTTGTAGTGTTATCTTTCTTGCTTTTATTTTCAGAACTTCACAACA

ATTTTGTAACATTTTGCCTTTTTCCTTTTATTTTTTGACAGTTATACATA

TTAAGGAGGGTTTCAATGGTGGCTATCCGAGCTGCATTAAACCTCCAACA

TGGTGGAGTGAGGGACTTCTATATATGTTCGCTTTCCTCAAGGTTTGTCT

TTATCATTTCTCTGGAATTACAAAATCTTGGTTGAATGTGACTCTCCAGT

AAGATTATACCATAGAGCCTTTTTTTTGTTTGTTTTCCAGGACCGTTGTC

TATAAAGGTCAATTGAAGCCCAACCAGTTGAAGCAATACTATTATGCAGA

TCTTGGCAATGAAAGGTTTACGAGCTACATGGCCCTGGTAAATATATTTT

CTATTCACTTTTCTTCTTTGTCCACTGACTCCATACTTCGACAACCAACA

GCATGGGATCAACCAGTTAAACGAAGAGATGCAGGGTTGAGGAATGACTT

CTGAGGCATTGAAGTATGAATGATCTCTTAAGTTCTTCCGCTTCCTTGAT

TACTATTTGAATTTGGATGCCGGATTGCATTCTTTGGGTGAGTTGGTAAG

GAACCTCATTATCAAGTAAATCGTGTTTGCAGAAACTCGATGCAGTGTTC

TCTATAATCAGTTCTTGAGGATAGACTTCCATTTAAGTGGTGGAAAGGAA

GTGTGTCTGGTTAAAGGTAGTTTAGGGTTTCCGTGATGTTATGTACTGGC

ACTGCGGTATGTTGTTTATTGAAGTGCCCTGTACACAATTTCATGGTTGC

AAGTTAGATCCCATATCGTCAATAATGTGGATGGAGTTTGGGCTGAAGTT

TTGGATCTATTTTTGGTGCAGATACACTCTCGATTCTCAACAAACACATT

TCCTAGCTGGGATCGTGCTCAGCCTATGCGTGTCTTAGGTCATAATGGGG

AAATTAACACACTTCGGGGCAATGTAAATTGGTAAGTTCTTGAGCAAGTC

GTGGAGTAATTGTTCCTGAATACAATTCTTTATTTCATTCATTTTTGTAG

AAGTATTCCGGAGATTTTGATATGATTGTTGATTGAACTTGAATATCTTT

ACCAATGACCTGTCACACCCCAGCTGAGCCTTGAAATTCTGGAAACTCAT

TAAAATTGATTGCTAAGATTTATAAGATATTTCTATCTGTAAGGAAAATC

AAGATCATCTTGTCGATTTATTTAGAAATGCCTGGTTGAATAGGTGGCAT

GCCATTTCGTGTGACATGTCATCGAGTTTTTAAGCCATTCACTACTAACC

TGTTACATGCAGCTTTATTAGCACTTGGAGAAATATCAGGTTCTGAGTTG

GAAAGTTGGTGGTTTCATTTTAAGTTTCTTGGATACCTGTTTTACTGGTA

GAGTAGTGCTATTCAGAAAGAATAAATACACACATGGTTTATTCTTTCTG

AATAAATACCTTCACTCAATTGTTTTCAAATGAGGTCCATATGTGTATTT

ATTCTTTCTGAATCAGTATACATTAGCAATAAGCCTAATAGGTAAGTTCA

AGATTATGTTCACAAGGATGCTAGTTGGGGCTTATGAACCTCTTAAATTT

CTGATTCATGTACATTACTTACTAATAGAGTTTATTGCTTCATTATTCAT

TTATTTTCCTGTTTAAAACAATTGAGTGATGTTCATGATCCCAATTTAGG

ATGAAGGCACGTGAGGGTCTTCTGAAGTGCAAGGAACTTGGTCTGTCAAA

GAATGAGATGAAGAAGCTTCTACCCATTGTAGATGCCAGCTCATCTGACT

CAGGTTGGATTATAATATTTGTTTATGTGCTTGCATACATCCGTATGTAC

ATGCATCTTGTATTTGAATGGTGGTGTTGATGAATTTTAACAATCAGCAC

GTAGACACTTAAATTGCCGATGTAAGGGGCGAAGTAGAACCACTACTCAT

GCTTGCCTGAAATTCAAATCATTTTGATAACCTTCTATTCTATCTGAATT

TCCCTCTTCTTTTAGGGGCTTTTGATGGTGTCCTTGAGCTTTTGGTTCGA

GCTGGTAGAAGTCTTCCTGAAGCTGTCATGATGATGATCCCGGAAGCCTG

GCAAAATGATAAGAATATGGATCCTCATCGAAAAGCCTTGTACGAGTACT

TTTCAGCCCTCATGGAACCATGGGACGGGCCTGCTCTGATATCCTGTAAG

AGTTCTACAAAACCTTTTTATTTCTGCATTGACTTGCTTCCCTAGCAGCA

TTTGGTTTGTGCTTACCAAAAACAAAATATGCAGTTACTGATGGCCGCTA

TCTTGGAGCTACACTGGACCGAAATGGATTGCGTCCAGGACGATTTTATG

TCACACACAGTGGGCGAGTTATAATGGCAAGCGAAGTTGGAGTAGTTGAT

ATTCAACCTGAAGATGTGTGCAGGAAAGGAAGACTTAACCCTGGTATGAT

GCTTCTGGTGGATTTTGAGAAGCATGTTGTTGTTGACGATGAAGAGCTGA

AGCAGCAGTATTCACTTTCAAGACCTTATGGAGAGTGGCTTAAAAGGCGA

AAGATAGAGCTGAAAGACATAGTTGAATCTGTTAGCAAATCTCAGAGGGT

TCCTCAGGCTATAGCAGGAGTTTTGCCCGTGAGTATTACTTCTCTGTGTC

TGTTTTAAGGTTGCAAATGCTTTCTTCTCCACCTCTTTTCATGGGCAGTT

CTCAATACAGATAACATCTCTTATATGTAACCAGGCATCTAATGATGACG

ACAACATGGAAAATATGGGCATTCATGGTTTGTTGGCTCCATTGAAGACT

TTTGGGTATTTTTCTCTCTTTTCCCTCCTCTTCGTCTGTAATTAATTTAT

TTATTTTTAAACTTTTACTGTGGCATTTCTGTTGTGGAAATGATTCAAAT

GCTGGCCTCTTATGCAGTTACACTGTTGAATCCTTGGAGATGCTGCTACT

ACCGATGGCAAAGGACGGAGTTGAGTCCCTTGGTTCAATGGGAAACGATG

CTCCATTGGCTGTGATGTCAAACAGAGAGAAACTCACATTTGAGTATTTC

AAGCAGATGTTTGCTCAAGTTACAAACCCTCCAATTGATCCTATCCGGGA

GAAGATAGTCACTTCCATGGAGTGCATGATTGGTCCAGAAGGTGATCTCA

CTGAGACTACTGAAGAACAATGTCATCGCCTCTCGCTAAAAGGTCCCCTT

TTATCTATTGAAGAAATGGAAGCAATTAAGAAGATGAACTACAGAGGCTG

GCGTAGCAAAGTTCTTGATATAACATTTTCTAAAGATCGTGGTAGTAAGG

GCTTGGAGGAGACCTTGGATAGGATTTGTGCTGAGGCACATGATGCAATT

AAGGAGGGTTACAAAACGCTGGTGCTTTCTGACGGAGGTACTGCATATCC

TAAACTAGGGCTGCACCTGAAAAAAATGGAAAATACATATATAAAGTTGG

CTTTATTTATCAAAAGAGAAAAGAAAAGAAAAAGAATCATCTTTATTCTT

TAATAGATAATATGTTATTAGCATGTTAAATAACTTATTTGTAATTTGTT

CAACTGTGTAGCCTTTTCATCAAAACGAGTTGCTGTAAGTTCCCTTTTGG

CTGTTGGTGCTGTCCATCATCATTTAGTTAAAAAGCTTGAACGGACTCGA

ATTGGGTTAATCGTTGAATCTGCTGAGCCCCGTGAAGTGCACCATTTTTG

TACTTTGGTTGGATTCGGTGCGGATGCTATATGCCCTTATTTGGCCATAG

AGGCCATTTGGAGACTTCAGGTTGATGGGAAGATTCCACCCAAAGCAAGT

GGTGAATTCCAGTCAAAGGATGAGCTGATCAAAAAGTACTTCAAAGCAAG

CAACTATGGCATGATGAAAGTTCTTGCCAAAATGGGGATTTCAACTTTGG

CCTCGTACAAAGGTGCCCAGATTTTCGAGGCGGTGGGGCTTTCATCAGAA

GTGATGGAGCGGTGCTTTGCTGGAACTCCGAGCAGAGTTGAGGGAGCAAC

GTTTGAAGCGCTTGCCAATGATGTGCTTCAATTGCACGATCTAGCATTTC

CTACACGGGTTTTCCCTCCTGGAAGTGCTGAGGCTGTATCATTGCCCAAT

CCTGGGGATTATCACTGGAGGAAAGGCGGTGAGGTCCACCTGAATGACCC

GCTTGCTATATCTAAGCTGCAAGAGGCTGCTAGAGTTAATAGTGTGGCAG

CCTATAAAGAATACTCTAAGCGCATACAGGAATTAAATAAAAACTGCAAT

TTGCGAGGGCTTTTGAAATTTATAGAGGCAGAGGTGAAAGTTCCTTTAGA

TGAAGTGGAACCGGCCAGTGAGATTGTGAAACGGTTTTGTACTGGAGCCA

TGAGTTATGGATCAATATCTTTGGAGGCACACACCACCCTTGCCATGGCT

ATGAACAAGATTGGGGGGAAATCGAACACAGGTATGCCCTTGATAAGATT

TTCTCAATTATTTGTGCTAATTAATGTCATTTATTTGAGCATGTCATGTC

AGTGTTGAATAATCTGAAACATTAGGAGGGACTTGGTTGTGTTATTATTT

TTGGCTTCCTTTTTTTAATGTCAAAAAATATTTGTGGAGTGTTGTTTTGA

ACTCATCATTTTTTACTCCCTTTTTTAATGTCAAAAAATTGTGGAGTGTT

GTTTTGAACTCATTATTTTGCATTTTTGTTTTTCCCTTCAATGCTCCTTC

AAGATCCATTTACTTGTTTGCCATTTTTGTTCTAGACTTTATTGGTCAAT

TTTGTAGACTTTGTTTCTGCATAAATTTGTTTTCTTTCATAGCTTATATT

TCTCTCGTTGCTTCTCCGGGGATGAAATTTCTCACATTCTTCTTTCCTTG

CACTTACTTTGTGCAACTTCTTTCTAGGTGAGGGAGGTGAGAACCCGTCT

CGTATGGAGCCTTTACCAAATGGTTCAATGAATCCAAAAAGGAGTGCAAT

TAAGCAGGTTGCAAGTGGAAGATTCGGCGTTTCAAGTTACTACCTTACAA

ATGCCGATGAGCTACAGATAAAAATGGCTCAGGTATATTTTGAATTGACA

TTTGAACCCCCATTTTTTTATTATTTATCTATCCTGTTGGCCTGTCTTTG

CATTTGGCGAGGTTTAATATGAACTGAACTTGTTTATTTTTGTATGTTGG

ATTTATTATTAATTTTGGACTGCCATTTTGCACAGGGAGCCAAGCCTGGT

GAGGGCGGTGAACTTCCTGGCCACAAGGTTATTGGAGACATTGCCATCAC

GAGGAATTCCACTGCTGGGGTTGGATTGATCAGCCCCCCTCCCCATCATG

ATATCTATTCAATCGAAGACCTCGCTCAATTGATTCATGATCTTAAGGTA

AGTACATGAACTGTTTCTTGTGCAAGTTTCAATTTTATTAGTTGCGTGGT

TTATCATATCTGACTTCTTTCTCCATAATTTGTAGAATGCAAATCCAGGG

GCTCGAATTAGTGTGAAGTTGGTATCCGAAGCTGGTGTGGGAGTAATTGC

TAGTGGGGTTGTGAAGGGTCATGCCGATCATGTCTTGATCTCAGGTCATG

ATGGTGGTACAGGTGCTTCCCGATGGACTGGCATCAAGAGTGCTGGCCTC

CCATGGGAACTCGGTCTTGCTGAAACTCACCAAACTCTAGTTGCTAACGA

CCTTCGTGGCCGAACGGTCCTGCAGACTGATGGCCAACTGAAAACCGGAA

GAGATGTGGCCATTGCAGCTCTTCTTGGTGCAGAGGAGTTTGGTTTCAGC

ACAGCACCACTCATCACACTTGGTTGCATCATGATGCGCAAGTGTCACAA

AAACACTTGTCCAGTTGGCATTGCTACCCAAGATCCAGTACTTAGGGAAA

AGTTTGCTGGTGAACCTGAACATGTAATAAACTTTTTCTTCATGCTAGCA

GAGGAGGTGAGAGAAATCATGTCTCAGCTCGGATTTAGGACCATCAATGA

AATGGTTGGTCGTTCGGATAAGCTTGAGCTTGATAAAGAAGTGACAAAGA

ACAGTGAGAAGCTAAAGAATATTGATCTCTCCTTGTTACTTAGGCCTGCA

GCTGACATCCGCCCAGAAGCTGCCCAGTATTGTGTACAGAAACAAGATCA

TGGTTTGGACATGGCTTTGGATAACAAACTCATAACTCTGTCCAATGCTG

CTTTAGAAAAAGGCCTTCCTGTATACATTGAAACACCAATTTGCAATGTA

AATCGTGCGGTTGGAACAATGCTTAGCCATGAAGTGACAAAGCGCTATCA

CATGGCGGGCCTTCCTGCAGATACAATCCATATCAAACTCAATGGAAGTG

CGGGCCAGAGCCATGGGGCTTTTCTTTGCCCAGGCATCACGTTGGAGCTT

GAAGGTGATAGCAATGACTATGTTGGTAAAGGACTATCAGGTGGAAAGAT

TGTTGTTTATCCTCCAAAAGGAAGCAATTTTGATCCAAAGGAAAACATCG

TGATCGGTAACGTTGCTCTCTATGGGGCAACAGTTGGGGAAGCATACTTC

AATGGAATGGCAGCAGAAAGATTCTGTGTCCGTAATTCTGGGGCAAAAGC

AGTTGTAGAAGGTGTTGGTGATCATGGATGCGAGTACATGACTGGTGGGA

CCGTTGTTGTGCTTGGAAAAACCGGGAGGAATTTTGCTGCAGGGATGAGT

GGTGGCATTGCTTATGTTCTTGATGTCGATGCCACGTTCCAATCCCGATG

CAATCCTGAGTTGGTGGATCTCGATAAAGTTGAAGAAGAAGAGGATATTA

TGACTCTCAGAGTGATGATACAACAACATCAGCGTCACACAAACAGCCAA

CTAGCTAGAGAAGTGCTTGCTGATTTCGATAATATTTTACCTAAATTTGT

TAAGGTCTTTCCTAGAGATTATAAACGGATTCTTGCAAGCATGAAAGAAG

AGAAAATTGCCAAAGAGGCTGCATCGAGGGCTGCTAATGAAGCTGATGAG

CAAGAAGAGGCAGATTTAATCAAAAAAGATGCTTTTGAAGAGCTTAAGAA

GTTGGCAGCTATGTCCTCAAATGGGAAAGATGTTCCGGTAAGTTATATTC

ATTTATTGATCAAATCTTTCTTTCATACTTGAACTGTTACATGATTTTAA

TGCTTGATAAGATGTGAGGTCTCTTTCATAGAAATAGGGATAATATTTTA

AAGAAAATTTTAATGTATAATTTTTTATTTTTTTTTATATTAAAGACTAC

AATGGGACATGTGGTACATATAAGAACAATTTGCCTCATTTTCATGGATG

CTTTTCTATACTTCTATTATATTCTCTCTCTCTCATCCTCCCCTGCATTT

TTCCCTCTAGCATGTGCTATATATCTTGGTAATGACTTATAGCCAGTAAA

AGGGTCTGATCAGCGTTCAGAATTTGCAGAAAGTTTCAGAGACAGAATTG

CCAACGAGGCCAACTCGAGTTCCCGATGCTGTTAAAAATAGAGGATTCAT

TATTTATGAGCGTGAGGGCGTCTCATATAGGGATCCTAATGTTCGGATGA

ACGATTGGAAGGAGGTCATGGAAGAAGCCAAGCCTAGTCCACTTCTAAAG

ACACAATCTGCTCGGTGTATGGACTGCGGTACTCCTTTCTGCCATCAGGT

GACCAAGAACTGCAATATTTTTTGTTCTTTTTAATTCAAAAGTCTCTCAC

AGCAAACTTTTGCCCAATTTTTTCAGTCTTATATCTGATTTCTATTTTTA

ATAAATTGTAGGAGAATTCAGGATGTCCTCTTGGGAATAAAATTCCTGAA

TTCAACGAGTTGGTGTACCAAAATAGATGGCGTGAAGCATTAGATCGGCT

TCTGGAGACTAATAACTTCCCAGAGTTTACTGGTCGGGTGTGTCCTGCAC

CTTGTGAGGGTTCTTGTGTTCTTGGTATTATTGAGAATCCTGTATCTATC

AAGAACATTGAATGCTCTATTATAGATAAGGCTTTCGAGGAAGGCTGGAT

GGTGCCACGACCTCCCCTCAGAAGAACTGGGTATGATTTTTGCTCTTGCA

TAATTGAATAGAGTCTCCATTCTCTCAGAGTTCTTTAGGGAATCATTCAG

TGGTCAGGGAAAGAAAGTAAAGTACAAGAAGATCAACTTTCCCATAACAT

TTTTATGTTCAATTAGCAAGGCTTTTGTGGGACTCATATCTTCAACTTTC

TCAGTAGGACAAGTTTTCCTTCAAAACATGCTCTGCAAAGAAATACAGGA

TTTTGCAAGAATCTCAATAATTCACTTTCCTTTTCCTGACAAATGAACAC

GCATGGAAAACAAGTTTCCTTTCCTTTACTATACAATACATTCCTGGGAA

TAGCTTTCTCATGACACAAAATTCTTGATAACTGATTGGAGCCCTAGTGA

TTGTCATGTTATGGATTTCATAATTAAATCTATATCTTTTGAGCCGAATG

TTATTTTGGTATAAATTATTGGGACACTGGATTCTAAATAACCTTGGTCA

TACAGGAAAAGAGTTGCTATTGTTGGGAGTGGACCTGCCGGCTTGGCTGC

TGCTGATCAGTTGAATAGAATGGGTCATTTTGTGACAGTTTTTGAGCGTG

CTGATCGAATTGGGGGCCTTATGATGTATGGAGTTCCTAACATGAAGACC

GATAAGGTTGATATAGTTCAACGACGGGTTGACCTTATGGCAAAGGAGGG

TGTCAATTTTGTGGTCAATGCTAATGTGGGAAAAGATCCCATGTACTCCC

TTGATCGGCTTCAAGAGGAGAATGATGCAATTGTTTTAGCTGTAGGAGCC

ACAAAACCAAGGTAAGTTTTTTTTTTTAAAAAACAAATTGCCAGTATAGT

TTTCTTTAGAACACACTAACAATTAGTGAACAAAATATTGGTTACAGGGA

TCTTCCTGTACCCGGACGCGAGCTATCAGGGGTTCATTTTGCCATGGAGT

TTCTTCATGCAAATACCAAAAGTTTGCTTGATAGCAATCTTGAGGATGGC

AAATACATCTCTGCCAAGGGCAAGAAGGTAGTGGTAATTGGTGGAGGCGA

TACTGGTACAGATTGCATAGGAACATCTATCCGACATGGCTGTACTAACA

TTGTAAATCTGGAGCTTCTCCCTCAGCCTCCTCGAACCAGAGCCCCGGGA

AACCCTTGGCCACAGGTTTGTAGATTTTATTATATGGCAACTGAAACAAA

AATAAAAAATAAAAAAATGCAGAGTACACAAGACCATTCCTCAATATGTT

GGTCATATATAGGGTTGTAAATGAGTTGAGTTGAGCAATAGGCTGTTTAA

ATTTGACTTGAAAACTTAATGAGCTTCAAAATGTGTTTAGGATTGACTTA

TTTAACTTTTGAGTTGATCTCAAATGAGCTTGAATTGAGTAGTTTGATTT

GTTATAGTGCATATAATTGAGTTGAGCCGAGTAGTAGGTTGACTATGCCG

AGCTTTCAAATTTGTTTAAGTTTGACTATGCCGATCTCAAATGAGCTTTT

AACGAGCTTAACATGAGTTGAGCTCGAGTAGCATGGTTCATTTTACAGCC

CTAGTCATGTAATGTTCTCTGGTTGGTCTTACTACATGTGCATCATCCCT

ATCAGATTTTGCAAAACTTGCTTGAAACAAAGTGAAAAATTATTGAAATA

GGTAATTAATTAACAAAATAATATAGAAAAATGGTAAATATTCAAATAAA

TTTTGAATGGTGTAAAAGCAACAGATTTTAAATGGAAGATTATAATAGTT

GGTGGTGGATGTTTTGGAATATCTGTTGAAAATCAGGGTTACATGGTCTA

AATGGAAGAGTTTGAAAATCGCAAAAATCTTGTAACATTTGATGCCTGCT

TTTTCTAATCGTTTTAATGATTTATTCCCTTCAAATTTCTTTGTTTGCAG

TGGCCTCGCATATTCCGTGTGGATTATGGGCACCAGGAAGCTGCTACCAA

GTTTGGGAAAGATCCCAGGTCTTATGAGGTATTAACTAAACGGTTTGTGG

GAGATGAGAATGGGAATGTGAAAGCACTTGAGGTGATATGTGTCCGTTGG

GAGAAGGATGCTGGTGGGAAGTTTCAGTTTAAAGAAATTGAAGGCTCGGA

GGAGATGATCGAGGCAGACCTAGTCCTACTCGCTATGGGGTTCCTCGGTC

CTGAATCAGTTAGTATTCTTAACTGCTACTCAATTATAATTTCTCTATGC

TGAACCAGTAACCAAGTTCAATTTCTGATCTAGAGTAATTGTTGGAAGTG

ATCTTGACTAATTTCTCTTGCAACTAGAATCATCAAAATTATTGGTTAGA

ATAGCGTAATTAAGTTTAGCTCCAGGCATTGATATTCTATGCCTTGGGTC

CTTCGCAAAATTGAAGTTGTGCATTCTGTTTTCTAATAGATTAGGGTTGA

GTAAAGAAATATGAAGACCAATTGGTAATTTTCTCTCTTTTTTCAATAAT

GTTCACCTGAATTTTGTCATCAACCCTAGTCAATTAGAAAGCATGTATAA

TTGATTTCATACTGATTTTTGTGATGCTTAATTCCTTCAGACACTATCGG

AAAAACTGGGCTTGGAGCAAGACAATCGTTCAAACTTCAAGGCAGATTAT

GGCCGCTTCTCAACCAACGTGAATGGAGTCTTTGCAGCCGGGGATTGCCG

GCGAGGCCAATCTCTGGTAGTTTGGGCCATCTCAGAAGGCCGGCAAGCTG

CTTCGCAGGTTGACAAGTACCTCATGAGAGATGGACAAGACAACACAACC

AGTCTGGTCGAAAGGGGCAAGAGGCAACAAGATAGCAGCAAACAGACAGT

AATGACATAG

>TEA015769.1 locus=Scaffold3826:801198:806092:- leucoanthocyanidin dioxygenase-like

ATGACTACAGTGGCTGCCCCGAGAGTCCAAAGCTTGGCGACGAGTGGGAT

TGAATCGATCCCAAAAGAGTACGTGAGGCCAAAAGAAGAGCTGACGGGCA

TCGGCAACATATTTGAAGAAGAGAAGAATGAAGAAGGGCCACAAGTGCCT

ACAATTGACTTGAAAGACATAGACTCGGAGGTCGAGGAGGTGAGAGAGAG

GTGTCGGGAGGCGTTGAAGAAGGCGGCGGTGGATTGGGGTGTGATGCATT

TGGTGAATCATGGGATAGCGGAGGACGTGAGGGAGCGGGTGAAGGTGGCC

GGAGAAGGGTTTTTTGAGCAGCCGGTGGAGGAGAAGGAGAAGTATGCAAA

CGACCCTGATGATGGGAATCTTCAAGGGTATGGGAGCAAATTGGCTAACA

ATGCTTGTGGTCAACTTGAATGGGAAGACTATTTTTTCCACCTTGCTTAC

CCTGAGGACAAGTGTGACATGTCCATTTGGCCCAAGACACCAACCGACTA

CATGTAAGTTCAATAACATTTATTAATGAATGAGCGAGTGAGTTTGCTTC

TTTTACCTGGAAAAAAATTTAAAAAAAAATTGGTAAAAAATGTTTTATAT

GATTAGTTATGATATTGAAATTAGTGTAATAGTGCTACTTATAGTGGTGA

TGAACATGATGGTAGTGGAATTGGAAAAAAGATATTGGTCTTTGGTAGGA

GTGGCGGGGTTCCGATTTAGAGCACTAAATCAGCAGTTTTAAAAGCATCA

TTAATGAATTAGCAAGTGTAAAAAAACGTAAAAAATAAATAAAGGCCAAA

TTATCACATCAATAATGGAGTCCACATGATGATATGTTATTAGTGGTGTT

AAATTAGTGGTATGAAGCATTTTTCAGGAGTGGCGATGGTAATATGATGA

CATTAGAAAAATTTTAATTTAAAAAATATTTTCTACATTTTTCAAAAGTT

AAAGAATGTGAGATTTTAAAAATATTTTTTTGCTGACAAATAGTGGGTTA

ATTAACTTCAAAATAATTTCTTAATTTTTTTTATTTAAAAAGATATGAAC

CCTATCATACAAGAGTAAGAATTTAAATTACAAACTATTTTTCTTAATAA

AATTATGAATATTTTTTCTGGAGTACTTGGATATATGGTTCATTAATATA

TATATATATTTTTTTTGGACGGATGGTTCATTAATATTTCACACTCGAAT

ATGTTTAGTGAACTTGTCTTTTCATTTGTTTGTCCCATTTGACTTCTCTA

TATCATGATTTTTTTTGGGAAATTAACTTGACAATGATATCAAGAAAAAG

TAATTACCAACAGATGGAAAATATTATAAATATATAATTTTTTTTATTAA

TTTGCAATTAAATATCCAAGCCAGATCCTTTAGAGAGTAAAGGAGAAACA

AGCAATTTTATATATATATATATATATATATATATTATCTTGAATTTATG

GAGAGGGGTCTTGAAATAAGGATATACAAAAGATTGAAACACAGAGTCTC

CTGGTTTATGTACTTAACTAATAATGTTAAGAGGATGGGCATTTCTAAAA

TTTTACAAAAAGTACAACAAAATTAAAAGATGTGGAATAAAAATGTTTTT

TTTTTTTTTAAAAAGAGAGTATATTTTGCAAACTAAAATGAATATATATA

TATATATATATATATATCAAAACCAAAATGCTAACAAATGTACTTTTTAA

AACCACATGTATTTGGTCATTTTGATTTTCTTGAGATTTTATGTAAAATT

TTAAAAATACTTATCTTTTTGTGTTGAGAGTTTTTTAGTCTTACTTTTTT

GGGCAAATTACAAATACCGCCTCGAGGTTTGGTAAAAATATAGATTCTTC

TCTGAGGTTTGAGAAATTACAAATTATTTCATGAGGGTCAAATTTTCATA

ACAAATTAGAGGAAATTAGGGGGAAATCTGTATGAAAAGAAAAACCTCAG

GGGGCAATTTGTTATGGAAATATAAACTTCAGGGGTGAGATGCAATTTCT

CAAACCTCAGGGGGGAATCTGTATTTTCACCAAACCTCGAGGAGGTGTTT

GTAATTTGCCCTACTTTTTTTTAGTCTCATTTGATTTTGTGTTGAATTTT

TTTTTTTTGTGAGAGTAAAAATATAATGATTATGGATAAAAATTAATATT

TTGTCTTATTAGCTTTTGTAATCAATTTTGTCTTTTTGTGACTAAAATAG

CCAAAAAAGATTATCCCAAACATAGCCTAAGGAATTTTTGTACTCCAAAA

TGCAACAATTAAAAACTTAAGATTAAAAGTACAATAGAATGTATAATTTG

AAATACAATTTAGTTTTGATCTAACTTAAATCTAACCATTCCTACACTTC

TTTCAAAATTGTGTCAATCTAGTCCTGCAACAGTTGAGTACGCAAAGCAA

CTTCGAGCCCTAACTACCAAGACGCTCTCAATCCTATCCCTTGGCTTAGG

ACTAGAAGAAAACAGACTAGAGAAAGAAGTTGGAGGCAAAGAAGAGCTCC

TCCTCCAAATGAAAATCAACTATTACCCAAAATGCCCTCAACCCGAGCTC

GCTCTTGGGGTTGAAGCCCACACTGACTTGAGTGCAGTCTCCTTCATCCT

CCCCAGCATGGTCCCAGGGCTGCAACTCTTCTACGAGGGTAAGTGGATCA

CTGCAAAATGTGTACCAAACTCTATCATCATGCTAATTGGGGACACTGTT

GAAATTCTTAGTAATGGCAAGTACAAGAGCATTCTTCACAGAGGACTTGT

TAACAAGGAAAAGGTGAGAATTTCGTGGGCGGTTTTTTGTGAGCCGCCAA

AGGAGAAGATTATCTTGAAGCCATTGCCGGAGACGGTGTCAGAGGCGGAG

CCACCGTTGTTCCCGCCGAGGACTTTTGCTCAGCATATTCAACATAAATT

GTTCCGGAAATCCCAAGAACTTGGCTCTAAATAATTCTGGAAAATGCTAT

GCTATCGAAATTTTTATACAAAATTTGATTTCAGAATGACATGGCGGTGA

GGTGAAATTTCATAAATTAAAATAAATTAATGATGGGTCTTTATGTTTTT

TTAATGAAAAGCCATGTTATTTTGTATGATAATTTTGTATAATTTGTCAA

TTTGTGTAGCATTAAGTCTAAAATTAATTGTTTGGTGGTTGGTTTGTAAT

GTTATGGTTTGGTTTGATGTTTTAGAGTTTAATCATTGGCAGGTCTTATA

GACCTTAGCCTGTATTTGTCTCAGTGCTTGTCCTTTGCCTGTCTTTTAAC

TACAATTTATCAATAAGATCATTTTCTTGTTATAATCTCTGGTTAATTAA

TGGTTCTTGATTATTGAAATCTAATTTAGATACTTCTATTTATATTATAT

TAGTGAAACCCGTGTTATGTATGGGATGTAAAAAAATTAATAAAATATAT

TTATATGAAATAATATAAATAGTAGATTTTTGTATGTAAATTATGGAAAT

AAAAAAATATTGAAATTTACAATTTAAGTTTTACAATTTTATCACAATTT

ATGTATCAATTTTTAATTTGTCACAACTTCAATAATGCAACCTTCAACAC

CCCTTGCTACAGTTGTAAACTTGGGCGAGTTGAATCCAAGCAACTCGAGT

CAAGCCTGTAAATTTTTAAAGATTGTGCAACATAATTTTTAATTTGGTGA

GAATCTATGTAAAATTTAAAAATTCGAATGAATTTGAATGGATTTGGATA

GATATATCAAATAACCTGAGCAATCGAAACAAAATTATATCTATAAATAT

ATAAAATTATTGGGTAATTTATTTTTTTAATAATAAATAAGATTAATTAA

AAATTTAATATTAATGAATAAAAGCAAATACACATTTTAGAAATGGATTA

TCGAATAAGTTGATATTAAAAAGATTATGTTTTATAATGGCATAAAATAA

TTTAAATATGACTTTTCAAAAATTATTTTTAATTTAAAAATTACAATAAA

ATAGTTAAATATAATAAAATTAAATTTCTACAAAATAAAAGTATCTGCAT

AAAAAAATAAGGTAAGAAGAAAATTTAGAATGGTATTCTTATGGGAAAAT

CGGTCTAAGGTCCCTATCCAACTTTTAGTAATAGATTAGGTCCACAATCT

GTTCTTTTGTAGTCTAAGGTCCCTCACCCCATTCCCTCTCCATCCACGTT

GTCCTGAGCTTGATCACCGTTGGCTCCAAATCCCCAACCCTTGACCAGTT

GGCGAGAATTTCTCTGGACAGTGGGAAGTCCAGAGAAATTTGGAGAGAAG

TCAGAGAAATTTGGCGAGAATTTCTCTGGACAGTGGGAAGTCCAGAGAAA

TTTGGAGAGAAGTCAGAGAAATTTGGCGAGAATTTCTCTGGACAGTGGGA

AGTCCAGAGAAATTTGGAGAGAAGTCAGAGAAATTTGGCGAGAATTTCTC

TGGACAGTGGGAAGTCCAGAGAAATTTGGAGAGAAGTCAGAGAAATTTGG

CGAGAATTTCTCTGGACAGTGGGAAGTCCAGAGAAATTTGGAGAGAAGTC

AGAGAAATTTGGCGAGAATTTCTCTGGACAGTGGGAAGTCCAGAGAAATT

TGGAGAGAAGTCAGAGAAATTTGGCGAGAATTTCTCTGGACAGTGGGAAG

TCCAGAGAAATTTGGAGAGAAGTCAGAGAAATTTGGCGAGAATTTCTCTG

GACAGTGGGAAGTCCAGAGAAATTTGGAGAGAAGTCAGAGAAATTTGGCG

AGAATTTCTCTGGACAGTGGGAAGTCCAGAGAAATTTGGAGAGAAGTCAG

AGAAATTTGGCGAGAATTTCTCTGGACAGTGGGAAGTCCAGAGAAATTTG

GAGAGAAGTCAGAGAAATTTGGCGAGAATTTCTCTGGACAGTGGGAAGTC

CAGATAAATTTGAAAAGAAGTCCAGAGAAATTTGGAATTAGGTAA

>TEA030315.1 locus=Scaffold4701:1294795:1314001:+ Ferredoxin-dependent glutamate synthase, chloroplastic isoform

ATGCACATGATGTGTGTTACCAGCTCCTAGTATCTGCATAAATGGATGTC

ATGTTTTAAGATGTGCTTCACTAACACTTGGAGCGAAATATTTATATGGT

TTGTCTACTAACTATTAATGGTGTAGTGCAAGAAAAATATTGACAGCATT

GTATCCAGAAGGTCATGATTTTAAAGAGATTCTACGTAAAAATATCATAT

TGGGTCTGTATCAATTGATTTGAGTGATTTTTTTGATTTATCCATCCTGG

TTGAAGTATCCTAAGTTTTTTTTTTTGACAAGGAAAAGTCAACTGTGTCT

TTGTAGTTTGATTTTGATGAAAGGTTTACTTTGTGTTTGATTATTGCAAC

TTTAACCATCTTAGAAGGTGTTAGAATTTACCATCACCTACCACCACTAC

CACCTCACCTACTCCACTAAACCTAACCCACTACACCACTACCACCTCAC

CTACTCCACTAAACCTAACCCACTACACCACCTCACCTACTCCACTAAAC

CTAACCCACTACACCACTACCACCATCCACTACACCTACATCTATATAAG

GAACCATTTGTAATAGACAGAACAACACATCAAAATCAATCAATAATACA

ATTCTCTATTTCTCTCTATACATTAAATTTTCTACAGAAGGTATTAAAGG

TACTCGTTCTTATACTTGATGGAAACAGGGCTTATGGCTACTCCAGTGAG

GATGTCCAAATGGTCATTGAAACTATGGCTGCACAAGGAAAGGAGCCTAC

ATTTTGCATGGGGGATGATATTCCACTGGCGATATTGTCTCAGAAGTCAC

ATATGCTTTATGATTATTTCAAGCAGCGGTTTGCTCAGGTTTGCCAATTG

TTTTTAATTTTTTTTTTTTTTTTTTTGGTTTTAGATGAGCCTGAATATTC

TAATGGTGTGGCTAGTTGTTTGTTACGATAACAATATTTGACTGAATTGT

GATGGTAGTAAAATAATGTCTCGGGGTCATGGTTAGGAATTATTCTTGAC

GTGACAACAATAGGTGATTTAATTGTTGGTTAATTTGTCAAAGGTGTAAT

TTACAAGTTGGATAGTGGTAGCAGTGGGCAGTTGTGGCACAAGCAACCGT

TAATACCAAGCCCCACTTCCAATGGAAGTTGCCCCCACCTGATCCCCCCT

TGGCCAATAGTATGGTCTCATATCATACTAATGGAAGTTACCTCACCTGA

CTCACAAAACAGTCTTAATGGTCGCAGGGAAGGTTAGCTTAAGTAACTCT

AATGATTGGCACCCTACTAGAAAAAAATTGATTCAGCACTTGGAGAACTT

CCTAAACGTCTAAGCTTATTAATTAAGATATCTACATAGTCCATAATCGT

TAAAGGGACAAGGTGCATTGATGTTTGGGTTTTGAGCTTTGCTTGGGTCC

AAGTGTGGAACTATTAACAAGGTATGAGCCTAGGTTCCAAAGTTGCATGC

TTTTAGTCAAGGTGCAGAAAAGACCTGCCTTATTATCTACATTTATGTTT

ATGCAATTTATATATTTACGCTTGAATTTTATTTGATAGTTATGCTTGTA

TTTGACCTGAATTTGATGTTGACACGCTTAAAAATGTGGTGTATTATCCA

AATTATGCATTGTTATCTGGAGTGATGCTTTAGAAAACTTCATGTAAAAC

TTACCCTATATGCTATTACAGTATATACTTTTTTCTTCTCGCTTTTTTTT

TTCGTTTCCTCTTTTCACCTGAAAAACAATTATACAGATATATGATTGTA

TTATTTTCTATTTTTAATACTGCATTGTTTCCTTTTTATCGATACTTTGT

TTCTTGGTTATTCCCAAACATGTCTCTAGTAACACAATGCTTATCTTTAT

CTAGCATTATGAGCTTAGAGAATCAGAAGGTAGGGATTCTCTGCCGTCAT

GTTCCAGCTTCTTGCCAATTAATTGCACTGGTTGGATTGGCAACTAATCC

ATATTCAACTTGGAAATATGTTTTTCCTTTTAATTGGAAGCACCATTACT

GAGTTTTGTGTTACATGTGAGTTGCTTTGCAGGAGGGGACCTAAGAAAAA

AAATTTCCACCCAGAAATTAGTGTTTTGCATCATGAATCATATAATAATT

GTTGTATCATTATGCTATAAAACACTAATAGTTACTTTTTATTTTTTTAT

TTACTTATGAAAACAAAATAATTATTACATCTCTCTTCTAGCATGCTATC

TGCCACTGGCCAATGTGTTAAATTAGTATGTTTGAGTGACGTTTTAAGAA

AAGTTCACTTTGAATGTAGTTCTTTTGGCATCATATTTTGTAGATTCTAT

AATGGACTTGGCTTTTGACTATATTTATTGAATCTTACGTCCTTCCGATT

ATTTCTTTCGTATATATACCAGGTTACAAATCCAGCTATTGATCCTCTAA

GAGAAGGATTGGTAATGTCACTTGAAGTCAACATTGGGAAGCGTGGAAAC

CTATTGGAGGTCGGGCCTGAGAATGCCTCTCAGGTCTGTTAGCATTTCAA

TTGTCATGGCTTTTTATTATTTGCAGCACCCGGACATGTCTGCTAATATT

GGCTTTGTATAATGCTCAATTCTCTTTCATTCAACTCTCTTTTAGTTTTT

TTTTAATTTTTTTTTGTAGAACTTAATTATTCTTTGTACCAGGTTATTTT

GTCTAGTCCTGTATTGAATGAAGGGGAGCTTGAGTTGTTGCTGAAAGATC

CCTACCTGAAGCCCCAAGTCCTACAAACTTTTTTTGATATAAGAAAAGGG

CTTGAAGGTTCCTTGGAAAAGACACTTAATAAACTTTGTAAAGCTGCTGA

TGAAGCTGTAAGAAATGGTTGTCAATTACTTATTCTCTCTGACAGGTCCG

ATGAGTTGGTGAGTACCCTTCAATGTTGTCTATGCTATAATATGCCTTTT

AAATATGGAGATCATTTATGCCTCTCTCTTTGTGTCTCTCTGTCTCTCGG

TAATCTCCAGTTTCTTTTAATAATTTTTCCGTATATTTGTTTTCAATATC

ATCATGTATTCCAATTTTGTTGTTGTACTAAGTTTTATGAATTCTGTTAG

GTGTTGAATAGCTTGATATACATTATAGGGCCCATTTTTTAATAGCTTAA

GTTTTTGGGAAAATTGGTGACTTAACACTAGGAGCACTATTTCATAACAT

CCTTTTTAAATCCTCATTGAAGGGTTTGGTTCATGTCTTCAAATAATAGG

GTAGAAGTCTGTTAAATGAGGACGTTGCTTTTAACAAAGTCAATTGAAGG

AATTGGATCCATGTAGCAGACCCTAAGTAATTGGGATCAAGGCAGTTCTT

TTAATTGAGTAGCATAAAAAAATGTTGCATTCCTCGATGTTGTAATCTGC

CAAGTTTTTGTAGTTTGTTGAGTGCGACCACTTTATTGAGCTCTTATCAG

TCATTTCTTATTGATTAGATATGGATAGTAGGCTTATTTCATGCCAAGCA

GCTTTATACCTTTTTTTGTTCTTTTATGCCTGGACAAATATTTACATTTT

TCTATTATTATTAATTTATTGATTTTATTTTTTTTCAGGAACCGACTCGG

CCTGCCATTCCAATACTTCTTGCTGTAGGTGCTGTGCACCAACACTTGAT

TCAAAGTGGCTTGCGAATGTCTGCTTCCATTGTAGCAGAAACTGCTCAGT

GCTTCAGTACTCATCAGTTTGCTTGTTTGATAGGATATGGTGCAAGGTTA

TTTTGCTTCTCTTTGACTATGGCGCGTCCAGCATTTTCCCTTAATGCTGA

TATAATATTAGTTCTTTCGCTGTATATGTTGGAGAAATTATGTATTCTTA

TGTGCATTATCTTCAAACTTAGATTAAGTCTATTCAAAAGATAAAAATCA

GGTGGTTTTGATACTGGTTTCTTGATTCATGTCCATTTCCTTAGTCCTAT

TTATTTATTTATAACTTATCTCGGATCTTTATTTCTGTTCAGTTTTCTCA

TATGCTCCATAAGTCAAGTTGTTCCTTAACTCAAAGATACTGCACTCTCT

TGACCAGACATATGTCCATCTCATTGCTAGGTTGAGTAGTCAACCAATTG

ATGTTGATGAGTTTTGTCTGGTAATGGTCTAAATTAATGGGTTGCATGTG

GATTGATAACAATTCTGCAGCGAGTGCCTCACCTAAGCCAAAGTCTAATC

TGCTTAATGAAAGAGGTTTTAGGGTTTAGGGAAAATGATTTGAAGAATAA

GATTCAGATTTCAAGTCTTCCTTCCTAGGGATTGTCTCAGAGATCTGTTG

TGTGCGGAATTCAAGCCTTTCTTGCTAGAAATTTCAGAAATAAGCTGTAA

AATCGGAACTTTCAATTTTTCCCACCTTGGTCCAATTTTGTTTTGCCAGT

GCTAGTAGTCGAAGGATTCTCTGCCAGGCTCATCACGTGACAGTGGCAGA

AACCTAATCCTGCTAATAACAATTGTGAATCCTTTTATATTCTTCTTCCA

AACTTTTTCAGAAATTTGAATGGAAAGGATCCTTTATATCTTCTGTTGTT

TCCATTCTTTCAGCTTGTTTCTTGCACAAGTAGGAATGATCACAGTGCAC

ACATTAAAATCATTGCATGTCTTTATCATCCTGACAATGCATTTAATTTT

TCTTTATAATTTTCTTTTCAGTGCTGTGTGTCCATACTTGGCATTGGAGA

CATGCAGGCAATGGCGTTTAAGCAATAAAACTGTAAACCTAATGCGCAAT

GGCAAGATGCCAACTGTTACAATTGAGCAAGCTCAAAAGAACTTCTGCAA

GGTCTGTGCATTGTGTCTGGAATGTTTTTGTGTTGTACATAAGTACTGAA

GCATCCATTAATTATGTCAATTTAAGTATCTGGGCAGACTTTGATTGATT

TTGTACACCTTAGGTGGTGAATTAATCAAGCATTGTTTAACTTTTGGTTC

TTGATATTTAGGTGTCACTTACTTGTTTATTCCCTTATTGCTTCTATTTC

ATTACTTTGACGTGAGTACTTGGAGATGATAAACTATTGGAGCTAGATGT

CCTTCTTAATAGCTTGCATGTGGGGTAGCTTTGCTCGTTATAATCTGTAG

TTTGACAGCTATTGCAATCCATAGATAGTGTCAACCATAGTGATGTGGAT

TAATAAATTCATCAAAGACATTTTTGTGGGAGCAGTGAACTTGTGACAAA

GATCTTGTTACCATCTAGTGACTTCAGCCACTAATATACAGGATTGGAGT

TGAATGCAATGCGGTACAATGTAATGGAATTCTAGATTGACTTTGATGAT

ATACCTTATTAGAAGTGCTCCTAACTTTCTTTTTGTTATAGGAAATGTAA

GATGTGTTTTTATATGCCTGATACAGGAATTTTGTGATGTATCAATCAAA

TTCCTGTGATTTATCATCCAAATGACAACCATGTATCTACACGACATCAC

CTATGGTTATATATTACATCTCACTAGCCAAGTATTTTTTCCTAACCAAT

CCCTTTTAGTTCCCTTGTATAGTCTCAATTTCTTTCCTCCACTTTTTTTA

GCCAAACCACTGCTTATTGTGACTGGGGATTAGGGACCATGGAGCATAAA

AAGAATATAAAGTGGGTTTTGAGTGCTGTTTGTCAAGAAAACTTTTGTAT

CCATGCTTCATGCTTGGTGGGATTTTAGCAATTTGGACCAACATGATGAT

CAACTACTTCATATTTAGTAAAGGAGGACTAATTGTCTTTTGATTCCGCT

ATGGTTATGTTTTTTCTTTTTCATATTCAGACTTAGAAAATATGAAGAAG

AAGAACTCCGAAAATATGAGGAAGAAGGAATATATTTCTGTTATATTCAA

AACTTCTTATCTTACAAGACCAAATGACACTCCTTATATAGGAGATATTA

CAAACATCATCATAACCCATTATATGCCACTTACATATTATATATTACCA

CTATCTACCACTATCATATTATATACATGCCACTATCTACCACCAACACC

ACTATCATATTCTATACATGCCACTATCTACAACCAACACCACTATCATA

TTCTATACATACCACTATCTACCACCAACACCACTATCATATTATATGCA

TGAACACAATTTTCAGCATTCAGTAAGAAAGATATTATTGATAATTTATT

GAAAAAATAACAAATCAGTGGGAACAGGGCCAATTTATTTCAAATCGGTG

CGGAAAGGGGCATTTATTACAAATCAGGCTGTCAAAAGGTTCCCACTTAT

TTGTAATAAGTTGTCCCTTTTTCCCAATGATTTGTTATTTTCCATGTTAT

TTTTCATTGCCCAATAGCTATGGCTGTATGGCACAAATTGTTTCAGTTGG

TAGATATGAATTGGGTGGCTCCAACTTCTTGTGTTCAGATGTTTATGATT

GACTGTAGGGGTTTTTGACATAAAATGGGGAAAAAAAAAGAGGAGTAATA

ATAAATACATATACAAGTCTTATCAGTTCTTGTTTTAACATCCAAATGAA

GTTCAATAATCATTGAACAAGTGCAGTTACAAAGTTGGGAACAGCATTCA

GATATCAAGCTACTTTTAATTATCCGCATTTCGATGAACATTGTGACTTC

AAATTTTGGGCAAAATTGTACTTATCTGCGTTCAGCTTTTTTAAAAAATA

AAAAATAAAAAATAAAATCCTTATTTTTTTATTATTTTTAGTCATGACTT

TGGTTAATGATTTCTGTTAGTATGGATTGTTGTCTGATTGTTTACTTTAC

CTTACTTTAGTGAAAGTAAATGATTTTTAACTCATCATCTTGAAGAGAAT

ATGATGATTGTAAACAACATTGCAGGCTGTCAAATCTGGCCTACAGAAAA

TTCTCTCCAAAATGGGCATCTCATTACTTTCAAGGTACGTGTCCAAATTC

TTATTTCTTTTGAATAGTGACCTTATACAACCGTAATTATTCTCGTTTGG

TTCCAACGATAGATCTCATTGACAAGTTTCTTGGATTTAGTTATTGTGGT

GCACAGATATTTGAAATTTATGGATTAGGAAAGGAGGTTGTTGATCTTGC

ATTTTGTGGCAGTGTATCTAGTATTGGTGGATTAACTCTTGATGAGGTAA

GAAATGTAACAAAAAGGATCATGACCTCTACAACTTCTTGGTTTAGTTTA

ACTCTACGTTCATTTTTAACAAACACAATGTGTATTGCTATTTAAAAATT

TCATGTCTGAACTCATTCGATCCGACAGGAGGAGTAGTTCTCTATTTAAG

AGTATGATGTTTCACAAACATGTGGTTAATTGATGTGTTCCAATTTACTC

CTTCAGAACTCATTTATCAGTAAAAAAAATTACTCTTTTAGAACTCATTG

ATCTGTCCTGCTGTATCCATTTATTTACTTTTGGACATGGCAGCGCAGAC

ATTTTGTTATGGTCAAGTGGATGTTATTATAAAAAGTGGAATTATTGATC

TAATTATACCAGAAGTATATAGCTTAGCACGGATCTACATGTTTTGTATT

CTACTTAGTATTAAATGCACGGAGATTACTTATTTTAGCTGGTGAACTAG

GAGGCCAGAATATTGATCTTCCATGTCTTCTTATCACTTATATCTTTTAA

ATGTAGAAATTTAATACCTTTGTGTTGCTATTTTGATCACCTGCAATCAT

GGTATTTAGAATCTTACAATTTACAGATCTATTCATCATACGATTTGATA

TGATTTTCAACCAAAATTATTTGATGTGTGTCGTTAAATCTTAAATTGAG

TGAATCGTATGATTCAATTGTGTTAAAATCCACCTACTCTCCACCTACTT

CACCCACTTAAACCTACTCCACTAAACCTAATCCACTACACCACTACCCT

CTCCACCTACTTCACCCACTAGTCGTTTTTGCAAAAATAAAAAAGTGGTC

TATCAATTATTGATATACATGATTTTATATGTATATATACAATTTCGTTA

TATTGATATTTATTTTATTTTAAACACCGTGTATATTTTTATATGTATAT

ACATATAATTTTTTTTGGATATTATGCTAAATAAAACGAATCTTACGATT

CACGATTCAATTTGCGATTTGACAACAATGCTTGCAATATAACTTTGGAT

GCTAGAGATCACTATTTTAGCATCTTCCTGGTAATTATTTTAATCACCTG

GAATATAGTTTGGATGGAGATGTCTAATTTGAGAAACCAATGGTTTATGC

TCTGTTCAGCAAATACATAAAGCTTCCAAATTTAACTAGTCAAAACTTTG

TTGCCTGCACAGACTTGCATGTATATTTCTCGTGGATAAGCAATATATCA

GCATTTAAACCTAACTATATGACCAATTGTAGTGGGTCTATATCCAATGG

TTTCTTTCCTGAAAGGTTTTGGTGATAACTTTGGAACATTTAATGCATTT

ACTATGCTGGTATTGCATTGTGGATATTTGAGCCCCTCATTAATTTTAGG

TTCGTGAAATATTTATGTCCTGCAGCTGGCAAGGGAGACTTTATCATTTT

GGGTGAGGGCTTTCTCCGAGGATACAGCTAAAAGACTAGAAAATTTTGGG

TTTATACAATTCAGACCAGGAGGTGAACTTTTGTTATTAAACATCTAGTA

TTTTCGACTAATTTCTGTTGGGAATATGCATAGTTAAATTGTGTTCAAAT

TACTTCTCTTTTTCTGGGTACTGAGACCTTGTTGAAATTGTAGGACAGAT

AGTTAGGACAAGGGAAATAGTAAATTACTTTAGTTTCATTGGTTTAAATT

ATTTCATACATGATTCAAATGATTCAATCTTGTTCTTAAATTTCCAAAGA

TCACGTAAAAAATAGAGCAAAATAATCTGAGTGCCTTTTTAAGGGTAAGA

TTTGATGTTATTCATTTTAAAACAACCAGCCCTCTTCCCTTTTGTGAGCC

AAAAGGAGAACACTGGTTGCAGACGCATACTTTTTACCAACATAGGTGAT

GTCTTTCATGGATCAACTGAGAAATCCTGTGTTTTATTTTCACTCCCAGG

CCGAGCCTTCGTCCTCCCAAACCTTAGTAAATCTTTTGGAGGAATGTAGT

GCTTTTGGCCCATTCTATATAACGTTGGATCTGGTAAAAATTAATTTTCT

CAAGGGGAATGTTTAGGAGTTGTAAATATTTGCTGAAGTGGGGATAATGG

AGCTCTCTAAATAATAAAGCTTTGCAACTTTCTTTTATGTTTTTCTTTGC

TTTTCTTTGGAAGGTGTACAGTTGCTGATACATAGGTGTAATTGAATATG

TTGAGGGCTTCTCATTTTTGGATGAAGGGAAAGTTTGAGTTTTCCCCACC

TTATTTTCTTAAAAAAATAGATTTTCTGGGGGAGGCACATTTTGGTGAGG

GTATATGACTTGTTTTGTGGGTTCCTTTAGGGTGCCTTGCTGTCCTTCCT

TATTTTTATGAAATAATTTTTATTTATCAAATAAAGAATACAAACTCATC

AAGTTCTATTTAGAGAGAGGAAATTCTTACTACTGTTCATAACTAGAGAA

AGGAAAGATGCAAATGGGGAATTCCTGTCCACCAAGTTCTAATTACGACT

GACTCAGCAGAATATCACTGAAAATCCATTATTGATCATATTTATTTATA

ACTACATAAAGATATCAGAATCCTGCCTTTGTTTCAAGGATCCTGGATAG

TAACATGCTTCTTTGATGTTATGGTTCTTGGATCATATTTCCCTGCTGCA

CTACCTTTCTGCCTGCAGCATAACTTAATGATTTATAATTATTAAAACTT

AGTATATTTTTTTTTCTTGTTAATGACGCTGAAGCTTTATTTCTCGTACT

GTCATACATTATGTAAAGGGGAATATCATGGAAACAACCCAGAGATGTCA

AAGCTGCTTCACAAAGCTGTTCGCCAAAAGAGTGAAAGTGCTTATTCAGT

GTATCAGCAGCATTTGGCTAATCGACCTGTCAATGTAAATTGTTGTAGTC

ATTTCTTTTTCTTTGTTGTGTGACCTTTCTGATTTTTCTTAGATAATTAA

AAACCAATATTTTGGAAAGAATCTCATGCAACATTTATTTTGTTCTTTTT

CAATGTAGGTTCTTCGTGATCTTCTTGAGTTTAAAAGTGATCGTGCCCCA

ATTCCAGTTGGAAAGGTTGAACCTGCTGTATCTATTGTTCAACGGTTTTG

CACTGGTGGCATGTCACTTGGAGCTATATCTAGGGAAACTCATGAAGCAA

TTGCTATTGCAATGAATAGATTGGGTGGAAAGTCTAATTCAGGAGAAGGT

GGTGAGGTAGTATTCAGTACTTATTTTAAGCGCATTGCACAATCATATTC

CGTCTCTTTTTTTTCATCCTTCCAACTCGAGAAGTTTTTTTTTATTTTTT

ATCCACTCTTAGATTTATTCCTTCCACCATGAACTGTATATGGGTGTTAT

GTTATATGCAATATTTATTTTAATCCAGGACCCAATTCGGTGGAGCCCAC

TTACTGATGTTGTTGATGGCTACTCTCCAACACTGCCTCATCTCAAAGGT

CTTCAAAACGGGGATACTGCTACAAGTGCTATCAAGCAGGTCAAAAGTGT

TTAATATTTTCTTTTTGGTCAACTTCATTATGCGTTCTAACATCCATTGT

TTTAGAATTTTGTTACTGTGCTTGTTTAGATGTGTGTATATATATATATA

TAGAATTAAAAACGGACAGAAAGTGCATTGCGAAAAGTAATGATGTTTCC

ATCATAGGACATTACTCTGTCTTTAAAATACCCAGATAGGGTGCATCTTC

TGTTATGCTTTTAGTACACCAATAATACTTCACAAAATAATTGTTGCATC

TTCTTCAGGTTGCTTCAGGACGTTTTGGTGTCACTCCAACATTCTTGGTC

AATGCTGATCAATTGGAGATCAAAATTGCCCAAGGTGCAAAGCCTGGTGA

AGGCGGACAGTTGCCTGGGAAAAAAGTTAGTGCATATATTGCAAGATTAA

GAAATTCTAAGCCTGGGGTTCCCCTTATATCCCCACCTCCACATCATGAT

ATTTATTCTATCGAGGATCTTGCCCAACTGATTTTTGACCTCCATCAGGT

GAAAATTCTGTCATATTGGATCTGATTATTATCATAATTAGAAATGTGAT

TCTAGAAACCATGATAGTAATGATCTCTATGTTCTGATCAATGGACAGGT

CAACCCTAAGGCCAAGGTATCTGTAAAGCTAGTGGCAGAAGCTGGAATAG

GTACTGTTGCTTCTGGGGTTGCAAAGGGTAACGCTGATGTTATACAGGTA

TTATGAGCGGCTTGTCATCTAGAAGTGATGCAATTAAAATTTTTGAAAAT

TTTCCCATGTATATTTTTTTAACAGATTTGTCAATATTGTTAACTTTCAG

GTAATGATGAACTTTCAGAAGTACAAGAAAATTATTTGGTTAAAATAATT

CCATGGTTTGTGTGTACGATGGGGGGATCTGCGTGTGCCATGCGTTGGGC

ATGTCCTCTATGAGGCCTGGACACCATGTTTGTTCGAAAAAAAGAAAGAA

AAAAAAATAGAAAAAGACAAATGGTTGACTGGCAGAGGATGAATAACTCT

CTTTCACATTGTTACTGCATGAGTTTCTTACGAACATAATATATATTTTT

TTGGTTGAGAACATAATGATACGCATCAATGTCTGTAATTAGGCAAATTG

TTTCAATGGATTCTTGTTGCTACATAATTTTAAAAAAAAAATCCATAAAA

ATGGAATATAAAAGTCACAAAGTTTTTGGATAAATTGTTTCTGTGCTTAG

CAATTTGTGCCAGAGCAAATGCAATGTCAGGTGGCTGTAAGGAATTCAAT

AGAATCATAAACCACCTTTTGCTAAAGCTTTTTCACAAAGTTAATTTTTG

TGTAAATTACAAACACGCCCCACCCCTTGACAAAATACATTTAACTTTCT

CAATGTTCTAGAAAATACATCCCACCCCCTGAGGTGTAAATCGCATAACA

AACACCCCCTTCGTTAGTCAACCATTAGGTCATCAATTTTTCTTTGATGT

ATTAGATGATATCTTCTTTGACTTATGATTTATGCATTTTTATGGATGAT

CCATATATTATGGAACTTAAATGCATTTCATTATTTTTCTCGTTGAACTG

GTGTTAATTTCTATTATTTTTGGATTTTATGTTGATATACATAGAAAATA

AAAATATATAATGCTGTATGCTCATTGTGTCTGTATCCTTATTTTTTGAA

ATTCATTGTGTCCCATGTAACCTGTGCCCGTGCCCGTGTTTTCTTAGTTG

TGCATAATATTATAAGTTTGTTTCTTTAATATTTGTAGTTGTGCTAGTAA

GGTGATTTACCTTTTCTTAGGCGGTTGCAAATTTGGATTTATTTGATTAA

CTACAAGGCTACTTAGGTCTCAAGATTTTTATATAGTGTAAATCTTACCA

ATCAAAGCAGAAAATGGGAATATTTGATGGTTGGTAGAGATAACTTGATC

TCAATGTGAACTCGACATTAGAGGGTAAACTTTTCAACTCAAACTTGATA

CATCATAGGCTTTGCCTTTACTTGGTAATTGTGCTCTAAACATGTAGTTC

TTTTTTTTTTCTTTGGAACTTCATTTTCATTTGTTAATGACTTCACATAA

TTAGAACATGTGCAGCTTTTGAACCAATTAAACTCGAAAAGTTTTGTTTA

TTAGATACTGTCATCTACATAACTTCTTCATAACTATATATAGTATCAGT

TGGACATACGAGAACACATCTTGTAAAAGTAACATTTTTTGCTCTCATTC

CAACACAGTAACCAAAATTCTGTGATGTATTAGAGAAGGTAGGAATATTA

TCAATTGACTGATACATGTAATTTGGTTTGCAGATATCAGGGCATGATGG

GGGAACCGGAGCCAGCCCTATAAGTTCCATCAAGCATGCTGGTGGTCCTT

GGGAACTTGGGCTTACAGAAACGCATCAGGTTTGCTTACAACATTAAATA

AATGGTTCAGATTTCTTCCTTTTATTTCTCCTGAATTACTTCAGGTTCTA

AATGTTTAGAGATGACTTCTGTACAGACACTCATTGAAAATGGACTGAGA

GAGAGGGTCATTCTTAGAGTTGACGGAGGCTTCAAAAGTGGTTTTGATGT

CCTAATGGCTGCAGCAATGGGTGCTGATGAGTATGGGTTTGGTTCTGTAG

CAATGATTGCTACTGGATGTGTTATGGCTCGCATCTGTCACACAAATAAT

TGTCCAGTTGGTGTTGCCAGTCAGGTATTAAGGTTTACAACAATATATTG

TGTCCAAATTCCTATGCTCTTTCTAAGATACCTGGATGTCTGCTCATTTC

CCTTGTCTAATGACTAATTGCATATTTCAGAGGGAAGAACTACGAGCTCG

TTTCCCTGGCTTGCCTGGCGATCTTGTAAACTTCTTTCTATATGTTGCTG

AAGAGGTAACTATATGCTGTCTTTTTGGATGTTTGATGGATTTAGCGCTT

CCATGCCTAGAGTCTCCAGTTTGACCTTTTCATCTATTATGTTATATAAA

ACAACACTGGAGGTTGTCTGACCAGCAGTTTGGCATGGGAGAAATTATGG

TCTGACAGGAGGCTAAAAGCCAAATGCTGCCACTTGGAAAAATCTCTTAT

AATGTGTGATAAAAAAAAGCGGCAGAGGTAATGGGGGTATAGCTGCACTT

GAAAAACATTTCATGAACAATGTGCATAACAGATGGCACCTTCCTAGTTT

TATTTTATGTCAATTGACTGTTCACTTGATTTATATAACCAAATTCTTGA

GTTATGTTCAATTTGTGACATTACTAGGTTCTATAAATGGTCCATGGAAT

TGGAATTTGAGTTTTTGGTTGCATTTTAGTTCTAGTTCTACCAGAATAAA

ATTGAAGTAATAATAATTTTTGTTCGAGTTCATTGAAAATTTACCTAATG

TAGAATTTGAAATTTGGGATGATGTTTGGATTATGAAATGATTTGATAAA

TTCCAAACTGTGTTGTCAATAATGTCATGTATAATTGGGTGAACTTTCTA

CATGTGTCTTATATTTTGACACTAGATTCCTGATTAAATATGGACCTGTC

TTTTATTCAAATACACAACATGGCACTGAATAAAAACTCATTGCTGGTGA

TAGACCATATCGCAGTACTCATCCAATAAAGAAACTCTTGGCATTGCAGC

AAATCAAAATTTGTTCATCTCCACTCAAGGTTCATGAAATTCTTTCTAAA

ATGGGATTGGGGCTAAGATTGGGAAAAAGCGAAAAAAGAGAGAGTTTGAT

GGTGATTTAAATTGGATGTTTCAATGTTATATGTACATTAAAAAAAAAAT

AATAATAATAAAAACAAAGATTCCTCACCTGCTAAAGCTAGACAGTACAA

TGGAAACAATAAGGATCAAATCCACTATTTGTAACAAGGATCACCGCATA

AACAATGGGGGCTGGAGAAAGAAATCTAACCAGATGAGGTTCGAGGGTAC

TGGAAAACTAGAATTAATACAAATATAGATGGGTTTGAAATTACTTTCAA

GTTTCCCATATTGCATTATGCAGTTGGTGGAATTTAGGGAAAAAAGTTGC

TTTTGTGGAAAGGGCGAATATAAGTTTGTAAGGTACTGTGAGGATGAGAA

CAGAAGTGCTAAACTTTGGGGGACGTGTTGTTTTTTGTATTACAGAGGAT

CAGCTATCTTAAAAATTCCAAGCATGTAAGATTTTGAATTTTGAAATGTG

GACCGCAAACAATTTGATCTTCATTCTTAATTAAGTGAATCCAATTTTTT

CATAATTGATAATAAAATTCAAATTTTGATTATGATTATGTGTCAAATCC

TAAGATCAATTAATTCACCCCAAAAAAAAAGAGCATCATAAGCCAATTCT

GTTCTTGGCTCAATTTTCCACTCTAATTTTGTGAATCTGTCTAGGCCTGT

AGGTGTGCCGCGTGTTATTCTCTCCACAAATTTTGATATCTTTAGTTTTT

CTCTGTCCACTATCTATTCACCAATGGTCATCGATATGTTTTTGGGTCAG

GTACGGGGCATCTTGGCACAACTGGGCTATGAGAAGCTGGATGACATAAT

TGGCCGAACAGATCTACTAGGACCACGAGATGTATCGTTGATGAAAACAC

AGCATCTTGATCTCAGTTATATTCTCTCTGTATGTATCCAGAAAGCCGTG

TGTTTTCCCTATCATCTCAACATTCACTTGTTCTTGCCTTATTATGTATA

TAAAATATTCCTTTTTACATTTCATTTTTTGACAGTGTGTTGGATTACCA

AAGTGGAGCAGTACTCAAATCAGGCTTCAGGATGTTCATAGTAATGGCCC

CGTTTTGGATGATATTGTACTCTCAGATCCAGAGGTAAACAATTATTTGA

TTACAGTTTGTAATTGAGCAATGAACATAAAAGAATTTCTGCGCTTTCAA

TCAATTTTTTTTTTTTCCTTTTGAATTACAGGGCCATTGCTAAAAAAACT

AATTCTACCAACTAATAAGCTTGTGTGCAACTGGAACTTCTAACTGAAAA

GCTTGATGTGAAGTTCTATCACCGTGATAGCGTGGTTGTTGAATTTTTTT

AATGACTAAGATTAATTTTTAAGAGTTGAAAGTATAAGTAATTGTTGGTG

GCAATAGTTCTTGAAAGTTGTTTTTGAAAGTTTTGCTTCCAAAGTAGTTT

CTTGTTTTGTTAGTTTCTTTTAGCATCATTATTCTATTTTGCTTATTGTG

GTCACTGGTCACAATCTAAGGAAATTTTCATTTTTCATGCAGATATCAGA

TGCAATTGAGAATGAGAAAGTTGTCAATAAAAGCATAAGGATATACAATG

TGGACCGTGCAGTTTGTGGGCGTATAGCAGGTGTAGTTGCAAAGAAATAC

GGTGACACTGGTTTTGCTGGGCAGCTGAATATAACGTAGGTTTTTGCTTT

GTGGTATTCGGTGTGTGGGATTGGCATTGTCTGTTCAGTTTAGTGCAATC

CCATTAATAATCAGAAATTCTCTTATGTATTAGATTCTTAGGGAGTGCTG

GACAGTCATTTGCTTGTTTTCTGACACCTGGAATGAACATTCGGCTGGTG

GGAGAAGCTAATGACTATGTGGGAAAGGTAATCTTATTTCACTCAAAAGT

GGAGTGTTGTTGGGTTGACACTGTATTGTAGTATGAAAATGATCAATCAA

TCAAGTTCAATTCAACTAGTTCATATTGATACAACTATACATTTATGCTA

ATGAAGTGTAAATTTGGTTATTTTCGGTCAAGCTACCTTACCATTTTAGT

GGAGTTTGATTTCTTTAGGTTTGAACGGTCATTACCTTTTCCACCTTCTA

TATCATTTTGTTAAAAGAAAAAAATCATTTTGTAAAATCTGACACTAGCA

GTTAATTTCCAGGGTATGGCTGGAGGGGAATTGGTTGTAACTCCCGTTGA

GAATACTGGGTTCTGCCCCGAGGAAGCCACTATAGTGGGAAACACCTGCT

TATATGGGGCAACAGGTGGCCAAGTCTTTGTCAGAGGGAAAGCCGGGGAG

CGTTTTGCAGTGAGAAACTCACTTGCTGAAGCTGTAGTGGAAGGTACCGG

CGACCATTGTTGTGAATACATGACAGGGGGTTGTGTGGTTGTTCTTGGAA

AGTAAGTATTGTCTCCTCATCTGCCATATTACTTGCTGTTCTTCTACTTC

CACAGAAACCAATCACAGAACTGGACGCAACTCTCTCACATGGTTGTGAG

ACCCACAAAAAGGTGGGATCCACCTCATGTGAGAATTGTGTCATGTTCTG

TGATCGATTTCTGTATTCAGAATTTTCCTATTAATTGACATGCATATTCA

ATGTTCTTTTTGCAGAGTGGGTAGAAATGTTGCTGCTGGTATGACTGGAG

GTTTGGCATACATTCTTGATGACGATGATACTCTTATCCAGAAGGTTTGT

GTCAAATACCTTTCTTACCCCTATGAAACAGTGGAAACTTATTAAATACT

CCTGGAGTAACCACATGGTATTGTATATTTGGTACTTCTGTCAAAAGAAA

AACCACAATCCACACTAGGTGGGTCAACATGTGATTGGTTTGGAGCACCA

CATAGGCAATTCCACATGGTGCTCCTGGACTACTTAAACAGAAGCTAGTA

TACAAAATCTTGATTTTTGTACAACTAGTTTGAGCACTTGCTTTGTCCTC

GCTGCTTTATTTTCCACCAAGTAATATTAGTTTTTAGTCTGTTGATTCTC

TAGTCATGGCTTGTATAATTGTGGACCAGGTAAATAAGGAAATTGTAAAG

ATCCAGAGAGTGGTTGCCCCTGTGGGGCAGATGCAGCTAAAGTGCCTGAT

CGAAGCCCATGTTGTAAGTGTGCTAAACAAACTGCACGATCATGACCACA

CGTACACAACACCAAAATGCAATTACATGCCTTGTAACACATTCAAATTC

TCCATAAAATGCCTTTTACATTGTTTTCTGTAAGTTACTAGGTATTCAAA

AGTAAAAAATGCAAGTTTAATTGCTAAATTATTTATGAAATTCTTGTTAG

GTGTCAGAGCTTTTTAGTTTGTTTTGGATAAACTACCAATTGGTCCTAAT

AGTTAGACTTAATTCTCATCAACTCTTATTGAAAATAAATCCTAGTAAAA

TCGTCGCTAAAAATAATTGTGACAATTTTTTGCGTGTTGCTAAAAATTGA

CAATTAGTGACTTTTTTTTGTCACGGTTTTTATAAATTGTCGCTGAAACT

TACAATTACGGATTTTTTTTGTGACGAATTTTTCAAACTATCGTTAAAAT

TTGAAAATTAGCGACTTTTTGTGATGATTTTTTTAAAATTGTTGCTTAAA

CTTATAATTACTGGCATTTTTTGTGACAATTTTCAAACTGTCGCTGAAAA

TTAACAATTAGCGACATTTTTTAATAGTATTTGATGAAAAACACCTTTAA

TTATCGTAGGACCCATTGAACTATAAGTCCACATATTAGGACCTACACGT

AAGTTCTCCTTTAGTTTTTTGGAAAATATTTGCCATGATCTTGTAATCTT

GTGTTCTTTTACCAAGTTCCAATCTTATGCCTATTGCCTGCAATGAGTAG

AGTTCTAGATTGCCTTTGCGTCGATATTTTTTTGTGTGGCTCCCTCGGAT

TTTAAGATTTTATATATGTTAGTGTAGAAAGAAGCATTTGGTATGTGTAT

TCGTTTCTTAGTTCAATGAACGGCTTGTTTTGCTTTTGATGAAGGAAAAA

ACTGGGAGTGGCATAGGCTCTTCTATTTTGAAGGAGTGGGACAAATATCT

ACCGCTATTCTGGCAGTTGGTTCCACCTAGTGAAGAAGACACCCCCGAGG

CTTGTGCAGAGTATGAGAAAACTACTTCTGGGACGGTGACTTTGCAGTCT

GCATAGAAGAACAGCACCACACAATACACAAAGTGCAGTTATATATAACT

ACGAAGTTTCAACCACATCGATCATGGCCAGAGCAGGGATAGATTCTATG

ATGGAAAATCTTACTCATTAATTGGAGCGGTTTGCAGGTGACTGAAATTT

CGTAATCATCTTATTACTTGGTGACTGAGACTTTGATCACTTCATTGAAA

AGCAATCAAGTGTCCTGTATAGCGATGGTGTGGAAAACTGTAAATTCTAG

GTAGCTGTGATATTGTCAATTTTTAGGCCTGAAGAGGTTTTTTTGTTTTG

TAATACATTTTCATGGCTTGTTTGTATGGTATTTTCTTAAATTTTTCTAC

TTTTATTCAGGATGAGCATAGCCAAATCTGAAAGCCTCGATCTTGATCTC

TAATTTTCTTTCCCTGTCTCTGTTTGTGCGAGTCTTGCACTTGGGTGAAC

TGTGTTGTAATTTATGTATCATAATTGTTATATATACATATAACAAGTTA

CTCTGGAAAATTTTACTTTCACATGAGGTTAGTATTGTTTGCAATTTGTA

AATCCTCTTATGCCTGCCCTATACAAGGGTTTTCGGCATGTTTTTCCTGA

GAAAGGAAATAGTGAGTAAATGTATGATGGACTGAGATGCCAAGAAAATA

AACAACGTTGCCCAATTCTTTAATAGAGAACTGTTAGATTTTACTTTCAA

TAAATACACAATAAGATGAAAGATTATTAATAATACTAATGTACGTTAAA

GAATTTTAAAATCATTAAAAAACAAGAAGATATATTTGGGTAGCGTTTGG

CAGCACTTTTCAAAGTGCTTTCTGGCAATAGAAGGCCTTTTATGGAAATT

TTTGGTGTTTGGTGAAACAAATGTAAAGACCTTTTTTTTCAAAAGTCACA

TTCCTTG

>TEA012255.1 locus=Scaffold5803:453732:456376:+ probable aquaporin TIP1-2

CCAAAAACAAGCAGAGAAAGGGGGTGTTTGCAATTGGAAATAGTCTTTTC

AATTTTTGTCATTTAATTAAAATTTTAAATTTTAATTTTGAAAAATGCCA

ATTTCAAGAATTGCGGTGGGAAGCCCAGCTGAGGCTAGTCAGCCAGATGC

CCTGAAAGCGGCTCTAGCTGAGTTCATTTCCATGCTCATTTTTGTTTTTG

CTGGTGAAGGCGCTGGCATGGCTTTCAGTAAGCATTATTTAAGCTTTTTT

TCTTTTTTCCTTTTTTTCTGATTATGTAATTGCTTTGAAGGGTACAATTT

TTATGAGGTGTGAAGTTTAACTCTTTTCTCGTTTGACAGGCAAACTAACA

GATGGTGGGTCGACAACACCAGCAGGACTTGTGGCCGCATCCTTGGCCCA

TGCCTTTGCACTGTTCGTAGCGGTATCGGTAGGGGCTAACATTTCAGGGG

GGCATGTGAACCCAGCTGTGACATTCGGTGCATTTCTTGGGGGTCATATA

ACATTGTTGAGAAGCATTTTGTATTGGATTGCTCAATGTCTTGGATCAGT

TGTGGCTTGCTTGTTGCTTAAGTTCGCTACTGGGGGACTGGTAAGACAAA

TGGATAAAAATCTAAATTCAACCATAAATTATTAATAAAAAGAGTGAGTG

TAAAATGTACTTAAAAATTATCTACAAAACATACTTATACACAGTTAGTT

AATGGGTTCCACCTTTATTTGTCGTTATATTTATTGTGAGGTGGAGTTCA

GTATTTAATGTGAGTTGTCAAACGTAGTTTTTTTTAAGAATTTTGAGATA

TATTTGTTTGAATTGGTAGCATTAGTCAACTCACAATATGCTAAAATGAT

AAATAAAAAAATAAAATTAAACTACATAAATTATTTTTAAAGCATGTGTT

TTGTTGTATTTTAGTTTCTGCTCTTCTTTTTTTTAATTAAACAGTTTGAT

TCCCAAACTACAAAATGGTGTATTTAAATGATCCATATATAGCAGTATCT

GTCTATCAGTTAACGAGAGTTTGGGATTTTTAGAATTTTACATAAACACT

CAACAAAATCACAAAAACCAAATACACATATGGTGCCAAAATATGTAGTT

GGTTGCATTTTGGTAGCCAATCTTTAAATTTTATTACATTTTATTTATAA

TTTATGAAAATATCACAGTTCCAGTTAATTAAGACAATTATTGGAAAATT

TTAACGGGTCCCTACGTGTAGATCTTAATAGGTGAATTTATTGTCAATAG

ATCATGTGAGAATTAATGGCGTTTTTCATCAAATACTATTAAAAAACATT

ATTAATTATTAATTTTCAGCAACAGTTTGAAAATTTTGTCACAAAAAATA

TCACTAATTATAAGTTTTTGTGACGGTTCAAAAAAATTATCACAAAAAGT

CACCAATTTTCAACTTTCAAAGACAGTTTGAAAAATTCGTTACAAAAAAG

TCACTAATTGTAAGTTTTAGCTACAATTTGTAAAAATATGACAAAAAAGT

GACTGATTATTAATTTTTAGTGATACATGCAAAAAAATATCATAACAAAA

AATTATTTTTACTAAAAAATTTACTACGACTTATTTTTAATAGGATTTGA

TAGGAAGTAAATCCAACTATTAGAACCTATTGATAGTTTATCCAAATTTA

AACTCAGCTTTTACAATTTAACAACTAAAAATAAGAAAATTAGATACTAA

AGTACTAACAAATCATATATTTTAGAAATAATTTGTATAGTTAAATCTAT

CCAAAAATGACAAACAAAACATCACACAAATGGAAAAATTATCGACAAAT

TTTATACAATTGGGTGGTGAATAACAAAAAAAAATGCAGTCCAATAATTT

TTTTTAATTTGACTAATTTGATATTAATACTGCAGGAAACATCAGCATTT

TCACTATCATCCGGTGTGACAGTATGGAACGCACTAGTTTTCGAGATAGT

GATGACCTTTGGTTTGGTTTACACAGTCTACGCTACAGCCGTGGATCCCA

AAAAGGGTGACTTGGGTATCATAGCACCCATCGCAATTGGTTTCATAGTG

GGTGCCAACATCTTAGCCGGTGGTGCCTTCGATGGCGCGTCCATGAACCC

AGCTGTGTCCTTCGGTCCCGCCGTAGTCAGCTGGACTTGGGACAGCCACT

GGGTCTATTGGCTTGGCCCCTTCATCGGCTCCGCAATCGCGGCTTTGGTC

TATGAACTCATCTTCATTTCCCCTAGCACTCATGAGCAGCTCCCCACCAC

AGATTACTAAGAATTATTGATGTCCGTATTGAAAATATAAAAGATTTTCG

CAAAATGAAATACAGATGTGAAATCTAATCTTTTGAATGAGAGGTGAGAT

CTAATATAAGATTCACTTTCTATCTCGCATCTGTGCTTTATTATGTTACT

TTTATGGTTTCAATGCTGTGGGGGGTGAGTGTTGAGTGGTGTTGTTTGCT

TTCATTTTCAGCAGTTGAGAGTTGGTACAGGCCTACTGGCTTTTGTTTCT

TGTGAAATGTGAACTTTGTCTTGTTTTTTTCTGGAATCAATAACAGAAGA

GGTATTTTCATTATTATTTGTCTTCTATATCAAATTTAAAATTTTGTCAT

TTTTTCTGGTACTATTACAAATTTGCCAAAGCAATCCAACTCCCA

>TEA009266.1 locus=Scaffold6323:213747:218165:+ anthocyanidin reductase ((2S)-flavan-3-ol-forming)-like

ATGGCAATGGCAATGGCAATGGCAACAACAACAACAACAACCAAACCGAT

GATCGGAGCGAAGGCGGCGTGTGTGGTTGGCGGCACCGGCTTCGTGGCTG

CCACCCTTGTGAAGATGTTGCTAGAGAGAGGATATTCCGTCAACACCACT

GTCAGGGACCCCGGTCAGTCTCCTCCTTCTCCTCCCTCTAATATTCTATT

CCATTTTTTACTTGTCAACTACTTAAAATTTAGGCTGAATTGGAAATGTA

CCTTCTTAAAATATGAATTATTGATGTAGTTTTAAAAATTATATTTTACT

TTTTAAATTTTTAATTGCATAATAAATAAATTTTTTTATTAATAAATCGT

TAAGTTAATTAATAATTTTTAATGATGTTAGAAATATAATTTAATTAATA

ATTTCGATAATTTTATTATTGTATAAAAAAACAGTTAGGAATTTTATTTT

TTTGTTGATTTTGTTAAATAAATAAAAAATTCTTGTAAGGGAGAATTTGT

AATTTTTAAAGCTATATGAAATAATATCAAGTGTCTATTGGTAATTTATC

CTAATTTTTTTTTTGGTCCTGGTACCCTTGTTTCTTCTACATATTTACAT

TTGTTTTCAGATTTTTTTGGGATTTTTTCACTTATGCACCAACCAAGAAA

TGAGGTGTCTATAGACCATAACATTAAGAGAGACCAACCAGCTGCATCTG

CCACTGGAGTGTGCATAATGGCATTTCCTTCCTTTTGTGTGGACCTAACT

TTCTGAACAAAATAATTTTGATAAATTACACTAAGCTTTCTTATATTTTA

AAAAAATAAAAACAAAAACAAATTCCCATTTTCATCAATGATTCGTTAAT

CATATGAATAGAAAAAATGATGATATCATATATTTTACACTAAAATGTAA

TTTTTTTTCTTATTTATCATTGTGATTTTATATTATATACATTAATTTCG

GCCACTTTAACCTTACTAGAATGTAAATTAACGTAATTATGTCAAAATTA

AAGGACTAATTATATCTTCTCCATTCCTAAAAGAAGGTCAGTGTAATTTA

CCCAATAATTTTTATGAGTAGTGGTGAGTAGGTTCGCGTTAAGAATGTGA

GCTCAACCAAGCCACCCATTAGTGAGGTTTGGAATGTGGTTTTGGTACAT

ATATATAGGGATCAAAGAAAGAAGGGAGAATCATAACACAGTGATTTTTA

CAAAAACAATTTACCCAAAATATGAAAAAATTTATAATTTTAATTACGAA

TATTTATAAAGTTTATCATTTATATGTTATTTTTTTATTTATTTTTGAAA

AACTTTACACAGAATTGTAATTCTGCCAAGAAAGAAAGAAACAATCATCA

CTAAAAGTTAGCCTTAAACAGACTATATTCCCAGTAACATCTATCAATTA

GGTAATGTTGTGGTATGTACTATATGTACCGTCATTACATATTTTTATGT

TATAAATTGTCATAATTCTACAAAAAAAAAAAAATCCCGTAATATTTATC

ACAAAAATACATGGCGTACACTACAAAAATACGTAAAAGGTGCATATGGT

ACAAACATTTTTGGTGGTGTGTACCATATAATTTCCCCTATCAAGAATAA

ATGAATTCATCATAAGAAGTGGAAAATAATCAATCATTTATTTTATTTTT

TTTGTGGCGAAGAACTAATATTGTAAGCTTCATGAACCTAAATCCCGAAC

GAAGCTATGTCTATGGGTAGGTGTGGCCTTGAAGTGAGGTAGGTGGGGGC

AATTGTCACCACTCAACTTTGAGAAATAACAAATCTGTATGACAAACTAT

TTTCAAATTTATTTTGTTTTTGAAATGCATCCCTTTTTTTAAGAAACACA

TTTAAGTTGTTTTATGAATTTTGTTTTGTTTTTTGCACAAAAATGAAAAC

AGGTGATGGTGAACTTTTTATTGTTTTTTTTTTTTTAAAACATGAAAAAC

AAACGAAACATAAAAAGTGAAAATGATACAAAACAGAATGTTTTCTGTTT

ATGTTCGCCCCATTTAAAATGGTGAATTTGCCTATGTTTTTGTTTCTGCC

ATGAAAATATGAGATATCCCATCTCTCTCTCTCTCTCTCTCTCTCTCGGT

AAGTTTTGAAAGAAATGGAAGACATGGAATAATGGTTGCTCTCAATTGTG

TGAGATAAAGAAGACTTTCTTTATATGTAGATCTGTATACAGCAGCAAAT

CAATATACAAAGTGAGAGAATAATACCAATTCTGAATTAGGTGTTTGATG

CAAGGGAAATGCTACAATGTTCAGTTGCTCATTGTAAGAAAAATTACAAT

TTTTGTGATAAAAGTTATAAATGGTAAAGTTACTATGTGCACCTAAAAGT

TATGCATAACTAAGGTTAATACTTTTTGTGATAAAAGTTGGATTTTTTTA

CATAAGGTGCATATCACATGCACCCCAAAAGGGGTGCACACAGTAGTATT

TCCCGATATAAAGTGTCTTATTTTGTTGAATTGCATTCAAATTTAGAGTT

TAAATTAGTAGAAAATGTCAATTGTATTGTAATTTCGAGTACGGTTAAGG

TTTTGCTCAGCAAAAATTGACTCACCCTGACTATATCGGCATATCTCTTT

TTTACTGTTTATGTTTGTTATTTTTTGGACAAATGGTAATGTGCTACAAA

ACAAGGGTATCATAAAACTGAAATAATTCTATAACAATCACACCACAAAT

GTAATAGAGTGGCAAGAGTCTTCTAATACACAATTTATCATGAAAACTCG

AAATATGAATTATCTTGCAAAAATGTTGTATGTCACCAACTTATTGGATG

TTTGTTTTATGTTGAGAAAAAATTTATGTAACAAGAAAAAAAAAAATAAT

CAAAGAGAGTCGGCTCTTCATATTTGTGTAGTGAAACATAACAATGATAA

AAGCCGGTGATATAACACTAAGTTTTTCCTTTGGGTGCAGATAATAAGAA

AAACATCTCTCACCTCGTAGCACTGGAGGGTATGGGAAACTTAAAAATCT

TCCGAGCAGACCTAACTGATGAACAGAGCTTTGATGCCCCCATAGCCGGT

TGTGACCTTGTTTTTGATGTTGCAACCCCAGTTAATTTTGCTTCTGAAGA

TCCAGAGGTATTTCATTACTATTCACATAAAAAATTAAATTTAAACAAAC

TGTCGATTTTTTGACAAAACTGATGATGAATTTTTGAATGTACCTAGAAT

GACATGATAAAGCTGGCAATCCAAGGAGTGCTTAATGTTTTAAAAGCCTG

CGCGAAAGCAGGAACGGTTAAACGTGTCATTTTAACATCATCAGCAGCCT

CTGTAACGATTAATCAGCTTGATGGAACGGGGCTTGTCATGGATGAGAGT

CACTGGTCAGATGTCGAGTTTTTGACTTCTGTGAAGCCACCCACTTGGGT

AACTCCCAAAATTCTTAAATTCATTAAGTTTTTTTATTTTATTTTTATGA

TCCAGAAACGAATAAATATGAATTTGCAGGGGTATCCTGTATCAAAGACA

CTAGCGGAGAAAGCAGCGTGGAAATTTGCTGAAGAAAATAACCTTAACCT

CATAACTGTCGTCCCTACTCTTACAGCTGGTCCTTCTCTTACATCGGAAG

TTCCTAATAGTATTGAACTTGCCATGTCCTTGATCACAGGTACAAAACTA

GACTGAAAAAAAAAAATGTATCGGATAAATTGCTGATGTATTGATTGCAT

TTCAACATTCTGCAGGGAATGAATTCCTCATAGATGGACTGAAAGGTATG

CAGATACTGTCAGGTTCGATCTCAATTACCCACGTAGAGGATGTTTGTGG

TGCGCACATATTTGTGGCGGAGAAAGAATCAGCTTGTGGTCGATACATTT

GTTGTGGTGTCAATTCCAGTGTTCCAGAGCTTGCAAGGTTCTTGAACAAA

AGATACCCCCAGTACAATGTTCCTACCGAGTAAGCCCTTTTTCTTTCTTC

TAAACAATTCACACATCAAGCTTTCTGCATTATAGACCTGCTTGGCACAG

ATTTTTGCAAGATAAAAGTAATAATCTAAATGTATTATCCAAAAACATCC

TTTTCTATCCCCAACTTCCCCAATAATCAGCTTTTTCAACAAGTCATTTT

TTCTCAAAAGTTGTAAGCACACTTTGTGCCAAAAGGGTACAAACATAAGC

ACAAAACAAAAATTTGTCCACACCAAGTGATATAGTATTAACATAATGAA

ATTCTGAAATTTTAACTGTCTGCAAAGTGATATATGGTAATTAATTGAAC

TTAATTGTCGTTGTTCAATAACAGTTTTGGAGATTTGCCGTCCAAAGCCA

AGTTGATCATCTCATCCGAAAAGCTTATCAAAGAAGGATTTAGTTTCAAG

TATGGGATTGAAGAAATTTTTGACCACTCAGTGGCCTACTTAAAGACTAA

GGGGCTTTTGCAGAACTGA

>TEA009696.1 locus=Scaffold10750:159644:163702:+ aquaporin PIP2-4-like

GGGGCGGCAAAAAATAAACTTTAATTAATGTTAAGACATTCCCATAAATA

CCCACATTTGCCTCGTCTCTCAAACAGCCATCAAACTACTCTCATCCCCA

AAACACACACACACACACACACACACACTCTCTCTCTCTCTCTCATACAC

ACAAACACACACTAGCCATGGCAAAGGACATAGAAGTGGGAGGCGGCGAG

TACGCGGCGAAGGACTACCAAGACCCACCACCGGCGCTATTGATAGATGC

AGAGGAGCTGACACTGTGGTCATTGTATAGAGCCACCATTGCAGAGTTCG

TAGCAACACTCTTGTTCCTCTACATTACTGTTTTGACTGTGATTGGTTAC

AAGAGCCAAACTGACCCAACCAAGACTACTGATGCCTGTGGTGGTGTTGG

AATCCTTGGCATTGCTTGGGCCTTCGGTGGAATGATCTTCGTCCTCGTTT

ACTGCACTGCAGGCATCTCTGGTGAGTACTTGTTTGTCGGATGATAATTA

TTCAGTAGTTTCAGAACGCTGGGATTGTTTTTTTTTATTATTTATTAACG

GGGGGGATTCAGAAATTTGTGGGAGGGGATCAATGTAAAATTACTTAGAT

TTTAAGGGGCATAAGTGATATTATGTATTTGGAAGAGAAAATGCTATTTT

TTTTAGGGGTAAGTTATAAATACACCCCTTGAAATTTGTTTAAATTATAG

ATTATTTCATGTAATTTAAAAAATTATAAATACTCATTTTTCAAAAAAAT

ATATTTAACATAATTAACTGAAAAAATGAAATTTCTAACGATTCTCTTAT

ATAACAATAAAATTATCTAAATTATTAAATAAAATATATTTATGATGTTA

TTAAAAATAATTAATTGATTTGATGATTTATTAATAAAATGAACAATTTA

TTATGAAATTAGAAACATCAAAGATGAATGTAATTTCTAAAACTACAGTA

GACAATTCATATTTTAAATAAATCTTAAGGATGCATTTGTAATTTACCTT

CTTTTTAATATATAAACACTCTGAATTGCATGAGATTAATGATACTATTG

TAAAGTTTAAAAGGGTTGAAATAGAATTATTACAAATGCTAACAAGTTTA

ATATTAAACTCATATAAAGTGTTTGGAAATAGTGAAAGTTAAAAATAAAA

TTTTGAAATTAATGAAGTGTTTTGTGTTTCAAGTACACTTTTGAGCAAGA

ATGGGAGGTTAAAAGATTTTTTTTTTTAATTATTTTATAAATATTCAAAA

TTGTACTGGGCTAATGAAAATTTTGTGAAATTTAAGATATCTCGCCCGTC

CTTGCTAATTAGTGATGCCACGCCAAAAATTATTCAGTGTGTTTGGTTTG

AATTTTTTGATTTAAATTTTTTGAAAATTAAATTTTTTTATTTTCGTATT

TTTTTATTTTTAGTGAATATTTTTAAAATTTTAAATATTTTTTTACTTTT

TTAGATACACCTTAGTTAGATATATCTAAAAAGTAAAAAACTTGTGAAAA

AATATTTTAAAAAATTAAAAAATACATAAAAATATAAAAAATATACTAAA

TTCTTGTATTTTTTTGTATTTTTGATATTTAGTGAATTTTTAAAAGATTT

TTTTTACAATTTTTTTTTACTTTTTAGGTGTAGTTATAGTAAGTGTCTAA

AAAGTAAAAAAATTGTTTAAAATATTAAAAAAATTCAGTAAAAGTAAAAA

AATACGGATAATAAGAAAGTAGAATTTTTTGAAAAGTGTTTTTAGAAAAT

TAAAACCAAGCAAGATGATTATTTTTTTTGGATGAGATCGATTTTATCTC

CTCTTTATTGGCTAAAACTTAAATAATCAACATAACTTTTCAACTTATTT

TTGAATTTTTTTTTTTTTTTTTTTGGTGGAACTTTTTAAGTCCATCTCAA

TTAAACTATTCTAAAAATTACAAAATTTGAGTCAATTTTGAAAACAAAAG

TATAATAATGCTAAAATTGAATTATTTCTTTTTTTAATTTTTAAATTAAC

TAGTTAGTCATTATGGATCTGAAAAGTAATGAATTTTGATCCACTAAGTA

AGGTGAATTAAAAAAAACAATATAAAAAAAAATAAGAGTAAGTTATGAAT

ACGTTTTTTGAAATTTATTTAAAATATAAATTACTTCTATGGTTTTAGAA

ATTACATTCCACCCTCTGAAATTCCTAATTTCATAACAAATGACTCATTT

TATCAATAAATCATCAATTCAATTAACAAATTTTAATAATGTCATAAATA

TATTTAAATTCGTAATTCAAATAATTATATTATTATATAAGAAAGCAGTT

AGAAATTTAATTTTTCTAGTTAATTCTGTTAAATAAAAAATTATTACAAA

GAGATATTTGTAATTTTTCAAACTATAGGAGACAATCTTTATTTTAAATA

TATATTAGGGGGTGTTTTTGTAATTGACTCAAAAAAATTAAAAGAAAAAA

AGGCAAGTTAAATTGACTCAAACCTGACTATTGTTGTGTGCTCTAGGACA

CTTGAAATTTTTTTCCAATATTGAAATCACAACATCAATATACGGAGTTC

CATATTTTCTTAGTTTCATATTGAAGTTTCAGGGAAATTAATGGTGGTTT

TTGGTAAACTATATATGCAGGGGGGCACATTAACCCAGCAGTGACATTTG

GTCTGTTGCTGGCCAGGAAGGTGTCTCTGGTCCGAGCCATTCTGTACATT

GTGGCTCAGTGCTTGGGTGCCATATGTGGTTGTGGGTTGGTGAAGGCATT

CCAAAAGGCCTACTACGTCGAGTATGGTGGTGGAGCCAACGAGCTCTCTT

CTGGGTACAGCACCGGCACTGGACTCGCTGCTGAGATTATCGGCACTTTT

GTCCTTGTTTACACGGTCTTCTCTGCTACCGATCCCAAGAGAAATGCCCG

CGATTCTCATGTCCCTGTAAGTATATATTAGTACTAATTCAAGTCTTATT

ATTACGCTATGCAATTTTTATTCTTCTATTCATCTACTTACAAAAAAGAA

AAAGCCAAAAAAAAAAAGGAAGTAGGTAATTTAAGGATTGGCATATAGAT

CCAAGAATATAACTTTGTACTCTATTTTTAAAAATGTTGCAAGATTTCAA

TGGTTGTAAATTAGGATAACAATGATTTTGCCAATTGAGGTTAGGAAATC

AAGCAGCTTCAGTTGAATTGATCGCAAATTGAGCTCAAGTACCTTAGTTT

TTTATATGGTAGAATACTTTAATCTTATATAAACTTGTTGATTTGAGTTA

TTCAAGTCAAGCTAAAAAAATTAATTAGTTTCAAAATTTGTTCAAATTTA

ACTAGTTTGCATAACAAATTAATTTTGAACAGTTAGAGTTTTTTATGACA

TGTTTATCTTTGGAATTAAAGGTTTTGGCACCACTCCCTATTGGATTCGC

TGTGTTCATGGTTCACTTGGCGACCATCCCAATCACCGGCACTGGCATCA

ACCCAGCCCGAAGCTTTGGCGCCACCGTGGTCTACGGCAAACAAAAAGCC

TGGGATGATCAAGTACTTGCCTCCACCCCTCTCTTTTAATTCTATGTTGT

CTTATACATAGTACTTATATTATACCTTAATTTTCATGTTTTAACTTTGT

TTTGTGATGTTAATTATTTCAGTGGATCTTCTGGGTCGGACCATTCATTG

GAGCCGCCATCGCTGCCTTCTATCACCAGTACATCCTGAGAGCCGCCGCA

GTTAAAGCCCTTGGATCATCCAGGAGCAATGCCTAAATAATGTGAATTAG

TTTCAATTTTACATGTTCAGAGGGTATTTTGGCAACCTGGCAAAGTTGTA

AATATGTGCTTGGATCACTTGTTGGCATTGTGGTTTTGGCACTTGTTCTT

ATTGTGCTTATTTGTTGTTTTCTACTCTTTTTTTTTCATTGTTGTTTTGC

TCTTTTTCCTTTGTTGTTGTTGCATCTTGGTGTTCCCAAGTATAATATAT

GAGCAAGTGCTTTTGTGTTTTTGTCTCAATCAACTCTGATTTCTTTAATT

CATGGTCCATTTTCATGGGCTGGTATTGTAAAAAGCAGTATTATAGCTGA

TCACTATGA

>TEA000080.1 locus=Scaffold12649:65156:70562:- protein TRANSPARENT TESTA GLABRA 1

ATAATCCTGTAAATAGGAAATGATGCAAGGAGGAATCCTTGCTCATTGGT

TGGTATGCCATCTTTGAATTGTCTTTTCTCACTTTCTCATACTTCCTCAC

TACCAAGAAAATTAGTTGGAAAGTTTAGCATACGACTGCCGATGCCCAAT

CCATAATTGGAATACTCCTCATTCCTTGCTCATACCGGGAAACTCTCTCT

CGCTCTCTCTCTCTCTCTCTCTCTCTCTCTCTCTGTGTTCAATTCACAAA

CCAAAACCCTAATCTCATACCCTAATTTACTCTCAATCTCTCCACCCCAC

ACCTCGTGGAATCAACAATGGAGAATTCGAGCCAAGATTCCCACCTCAGA

TCCGAAAACTCCGTGACCTACGACTCCCCGTACCCTCTCTACGCCATGGC

CTTCTCTTCCTCCGCCCGATCGCCGCCCCATCACCACCGCATCGCCGTCG

GCAGCTTCATAGAGGAATACAACAACCGTGTCGACATCGTCTCTTTCGAT

GAAGAAACCCTAACCATGAAAACCAACCCCAATCTCTCCTTCGAACACCC

TTACCCTCCCACCAAGCTCATGTTCCACCCCAACCCCTCTGCCTCCCTCC

GCAAATCCTCCGACCTCCTCGCCTCCTCTGGCGACTATCTCCGCCTCTGG

GAAGTCCGCGACAACTCCATCGAATCCATTTCTATCCTCAACAACAGCAA

GACCAGCGAGTTCTGTGCCCCCCTCACTTCATTTGATTGGAACGAGGTCG

ATACTCGCAGAATTGGGACTTCTAGCATCGACACTACTTGTACCATTTGG

GATGTTGAGAGAGGGGTTGTTGAAACCCAGTTGATTGCCCACGATAAAGA

GGTTTACGACATCGCTTGGGGCGAAGCTGGGGTGTTTGCTTCGGTTTCTG

CTGATGGCTCTGTCAGGATTTTCGATTTGAGAGATAAGGAACACTCTACC

ATTATATACGAGAGTCCTCAACCGGACACTCCTTTGCTTAGATTGGCTTG

GAACAAGCAGGATTTGAGGTACATGGCCACAATTTTGATGGACTGTAATA

AAGTTGTGATCTTGGATATTCGATCGCCAACAATGCCGGTTGCAGAGCTG

GAGAGACACCGGGCGAGTGTGAATGCCATCGCTTGGGCTCCCCAGAGTCA

TCGCCATATTTGCTCTGCTGGGGACGATTCACAAGCGCTTATTTGGGAGT

TGCCGACTGTTGCTGGACCCAATGGAATTGACCCAATGTCTATGTACTCT

GCTGGGGCTGAGATTAACCAGCTTCAGTGGTCAGCGGCGCTGCCTGATTG

GATTGCAGTTGCCTTTTCAAACAAAATGCAGCTTCTGAAAGTTTGAGGTA

TGGGAACATTGTTTTCTTGTTAGGTTTTATATTCTGCGATGTTCATTCAT

TCTTGAATTCGGATGCACTGTTATTGAGATTTACAAAGTAGCAACAATTT

GGAATGCTTGAGCTATATATGGGATAAATTTTCAACTGTTTGAGTTGATT

TTTGTACTTGTACTAGTCATTGATTTCTGGATGCTTGTCAATCTGTTGGT

ATCATTGTTGCGGCATATAAAATGTTGTGTTATTTTTTATTATTAAGGGT

AAAATCGAACCACATCTCGTTTCTGATAATGATGGATGCCATGTTTAAGA

ACAAATTCTCAAAATAAATGAGAAGATAGACTCTCCCGTGTGTATTGGTC

CGTGTCCTCTAGAAAACTGGTAACTATATTGTAATCCATAATTTGTATAA

AAACATTTACAACTAAAAAATTCCATCCTTTGGTTAATGTAGTATTACAG

TGACGAAATTTCTTGGAACGAAATGTCCTTTATAGGAAGTTCTAATGCAT

CATTATGTTTTGTGCCTCTTAGATCTCTGGTCCACGAGTCCAGTGATGTG

CAACTGAGCAGCAATTTTGTGGGAAGGTCTACCTGGAAGCATTAGTGTCA

ATGCAAGGAATGAAAGGGGGTGGGGATGGGGGTGAGGGTGGGTTGAGGTT

TCTAATTTTGTTCATGCCTCAGACTCAAACCTCTTTGATGACAATAAAAA

TTGCATTCAAAATTGAAGTGCTAAGTTAGTATCATTGAAAAAACTTCAAA

TCAATCTTAAGATTCTTAACTATATTCCAATAAACTTGTTTCGTGACATG

TGAAAATTAATGTTTTCTAAATGAAATTTCCAAATGCTTTTCAGTGAATG

GTGTACTTTTTTTAAGTGGTAAGGAATTTTGTCTTACCAGTTGCATTTAG

TGAAGAAATTGAAAAACAAAAAAGGCGCTTAAAGGAGGAAATGAGGTTTT

TTCCTTACATGCCTTTCACATTAGGTGAATCTCCTTTATGTCTTTTAAAT

CTTTCTGTTATTCACTACCAAATTTATTAGATTGAGATTTGAACTTTGTT

TTTTTATTTAATCTTTTTGTTTTTACGTGGTATCCATAGGCTTAAAACAA

ACACATATTCTGTATCAGTCGATAAATATGGCAAACAATTACAAAGTCCA

GTTACAAATGCAGTCCACATTCTTAATTGTAGCTCACTTAACTCATATGA

TTTTGCGGCTAGGTTGAGCATGTGATGGCACTATGTGGTGACACCCAAAA

GTGCACAAGAGGTCAAAGGGCCTTAATTATGTGACCCATATTCTTTTGTG

TCGCTATCAGTATGATGTAAGGTTGATTTAGGAGGATTTCCTGTCATTCA

AATGCATTGAAGTCATTAATTAGTTGATTCAGGGTAAATCCAAATTGCTT

TGAGGTTTTGGATTTGAGTACTTATATGCATTGTGTTTGAAACACGTACA

GAAAACATGGGATGTTGTCTATCATTGACCCTCAACTTCAAGTGAAATAG

ATGTTGAAAAACAGAGTAGAAGAAATATAAATTGAAAGAGAGAGTAGGAG

GCAATCACTATCATCTCCATTGCTGCGTAGGATTGAAGTTAGACTTGTTC

TAGGAAGATTTTTATAATGAGAGAGGCCAGCGTGTCATACCGCTATTTTG

AGATTTTGACATAACTTATTCATTGTTTGGTCATCAGTTAAATTAAAACG

GAGCAAAATTTTATACTTCTTCTGTCTTAGGGGAAGATAATTATTAAAAC

CATTACACTTTTTTTTTCCCCTGAGAAGTATTTAGGGTACATTTTGTATG

AATAGTTTTTGAAATACTCATTTCAGCCTCTCATTCACTTTCCCACATTT

GGTATAGTTGAAGGTTGGAAAAATCATTCCATTGGAATGTCCGTTCTCAC

AATTTGTTAGAATTACCATTTCACACCAAAAGGGAGGGGAATGGTTATTC

TATTCCGTTGTGTAATGACCAATGCTAGAGGGAATATATCATATATAAAT

ATAACTTAGGGGAAGTTAATGCGTTGACTGAATCAGTTGAATCCGTACGC

ATAAGATGAATTACCATATTTACCCTTTGCAGCACATTTATGTGGCCACT

CTTTCTCATCCTTTCCCATCTTCTCTTTCTTTCCCTGCCCACTTTCTCTC

TCTCTATCTATCCGCCATTGTTGAATTTGAAGGGGAAAAAATTGATAAAT

ATTACACCAGATTCAGGCAACCTCTCCCTTTATCGATCTACCACCTTCCC

TGATCTCTTTGTCTTCCTTTGTCCATCGCAGACTGGCTACCTTCACTGTT

TTGTCCAGCCCCCCTCCTCCAGTTTATCCCCTCTCACCCGATCTGTCTAT

TGCTTTCCCCATTTTCTCTCACCGACTATCTGCCTCCCTCCCTCCCTCCC

TCCCTCCCTCCCTCACAGCGATGCCCCTTCTGCTAACAAAACCTGATCTT

TCCCTTTTTATCTGTCACGCACTCCGCATAATCAGCAACCACTATCCTAC

TTTCTTCAATACAAGAATACACACTTTCACCCACCCACTTTTGTGGATCG

GACAATGGTGGCACCTTTGGTCTCAACACCAGTGATGGCGAACCTCATTT

GTATTGACAATGATGCATCAACGTTCTCTTCTTATTTAAAATATGGATAA

ATTACACTTTGCCCCCTTTATGTATGATCATTTTCTCATTTTAGCCCTTA

AAGTTTGTTTTTATTTTTTTATTTTTTTTTTTTAACTTTGGCTTTTCATG

TTTTATTTTTTTCCCAATTTACTCCTAGAGCTGAGTTCTATTCAAAATTA

GATAGAAAATGTCACGTTATCTGCCACATAACATAAAAAACATGTTACCA

TATTGAGGATTGGGTTTGTGTCAGAGGTATAAGTTAAATATAATAATATT

TTTATGCTATGTAGTTGTTAATGTGTCATTTTTTGTCTAATTTTGGACAC

AAATTAGCTTTTAAAGTAAATTGAAAAAACTTGAAACATAAAGTGTCAAA

GTGGGGAAAAAATAAACTTTAAGGGCCAAAATGAGAAAATGGCTAAACAT

AAAAGGGGCAAAGTGTAATTTACCCTTAAATTCACTTTCTACAGGTTGTG

AGTAGGGTTGACCACAGTGAAGGAAATTTGATTGTGTTGTGAGAAGAAAA

ACTGAGTGTGGAGTACATGTTGAAAGTGAAGTAGAGAAAGAAAAAGAAGA

GTCTCAGAAAAATAAATTAAAAAAGAAAGGAAGAACAGAGGAACTGGTGA

GATCTGGAGCCGCAGAGAGTGGATAAAAAAGGGTTGGCCACTTGACAAAG

GGTAAATACAGGAGTTTACAATAATGTGCGTGGAACTGTTTTTTTCAGTT

GTGGCATTAGTGCTACCCTATAGATTATATGTTGTATCTTCAATATTTAG

AAATATAATACATAATATATTTATGTGTATTTATATATATCAGTTTATTT

AATATTTTTATGTAGTGCTAGTTTTGTTTAGAATTTTCATCCCAGTCTGA

ATGTGTAAATATGCATGGGAATGAGAATAGTTATTCTTTGTCATGTGAAC

CAGAAGAATTTTCATCCCAGTCTGAACACTAAACATGGGAGTGAGAATTG

TTATTCTTTGTCACGTGAACCAAATAGTGGAATAGGAACGAGTATTCAAT

TCCATTCCCACTCTCATTTTGTTGTCATCTCATTCCATTATTGCGTACCA

AACATAGCCTGAAATCTTTTCACATCATATCATGTTCTGCTAAACTTTTC

TATATTAAATTTTTCTATCATGCTATGTATTATACTGGATAACATTTTCT

TATTCTCCATGCAGATCCCCAGATGATGGGTCATGCAGGCAACGTTTACC

GAAGCGCCCAGTTAAAGTTGTATTGTTAATTTACTTAGACAAACACGAAC

TAATCCTCAAATGTTGATGAGAATTGTGTTGATATGTAATTCAAATTGTG

GGTGCAGGTATCTTGTATTTTTCTGTTTCAGTCATATTGTAACACAAACT

CCGATCAGTTGGTTAGAATCCCCCCTCATGTGTTTGGTGCAGAAGAAAAT

CAAAAGA
